# Complex origins of chloroplast membranes with photosynthetic machineries: multiple transfers of genes from divergent organisms at different times or a single endosymbiotic event?

**DOI:** 10.1007/s10265-019-01157-z

**Published:** 2019-12-06

**Authors:** Naoki Sato

**Affiliations:** Department of Life Sciences, Graduate School of Arts and Sciences, University of Tokyo, Tokyo, 153-8902 Japan

**Keywords:** Glycolipid biosynthesis, Hidden cyanobacterial lineage, Host-directed chloroplast formation, Membrane origin, Primary endosymbiosis

## Abstract

The paradigm “cyanobacterial origin of chloroplasts” is currently viewed as an established fact. However, we may have to re-consider the origin of chloroplast membranes, because membranes are not replicated by their own. It is the genes for lipid biosynthetic enzymes that are inherited. In the current understandings, these enzymes became encoded by the nuclear genome as a result of endosymbiotic gene transfer from the endosymbiont. However, we previously showed that many enzymes involved in the synthesis of chloroplast peptidoglycan and glycolipids did not originate from cyanobacteria. Here I present results of comprehensive phylogenetic analysis of chloroplast enzymes involved in fatty acid and lipid biosynthesis, as well as additional chloroplast components related to photosynthesis and gene expression. Four types of phylogenetic relationship between chloroplast enzymes (encoded by the chloroplast and nuclear genomes) and cyanobacterial counterparts were found: type 1, chloroplast enzymes diverged from inside of cyanobacterial clade; type 2, chloroplast and cyanobacterial enzymes are sister groups; type 3, chloroplast enzymes originated from homologs of bacteria other than cyanobacteria; type 4, chloroplast enzymes diverged from eukaryotic homologs. Estimation of evolutionary distances suggested that the acquisition times of chloroplast enzymes were diverse, indicating that multiple gene transfers accounted for the chloroplast enzymes analyzed. Based on the results, I try to relax the tight logic of the endosymbiotic origin of chloroplasts involving a single endosymbiotic event by proposing alternative hypotheses. The hypothesis of host-directed chloroplast formation proposes that glycolipid synthesis ability had been acquired by the eukaryotic host before the acquisition of chloroplast ribosomes. Chloroplast membrane system could have been provided by the host, whereas cyanobacteria contributed to the genes for the genetic and photosynthesis systems, at various times, either before or after the formation of chloroplast membranes. The origin(s) of chloroplasts seems to be more complicated than the single event of primary endosymbiosis.

**Electronic supplementary material:**

The online version of this article (10.1007/s10265-019-01157-z) contains supplementary material, which is available to authorized users.

## Introduction

“The endosymbiotic theory” of the origin of chloroplasts and mitochondria explains that chloroplasts originated from an ancient endosymbiotic cyanobacterial cell, and that mitochondria from an ancient endosymbiotic α-proteobacterial cell (for reviews, see Archibald 2015; Löffelhardt 2014). The theory was first proposed in a clear form by Mereschkowsky (1905) for chloroplast origin, but remained a mysterious, unsupported hypothesis during the first half of the 20th century. The similarity of oxygenic photosynthesis involving chlorophylls, the presence of internal membranes later called thylakoid membranes (Stanier and van Niel 1962), the presence of peptidoglycan in some cyanelles (see Stanier et al. 1957/1976, p. 758), and later the presence of DNA in chloroplasts (Ris and Plaut 1962) constituted the initial basis of the endosymbiotic origin of chloroplasts (Echlin 1966; Sagan 1967). The results of phylogenetic analysis of ribosomal RNA, ribosomal proteins, and various other proteins encoded by the chloroplast genome clearly showed the close relationship between chloroplasts and cyanobacteria, and, after critical examination, were taken as good evidence for the endosymbiotic origin of chloroplasts (Gray and Doolittle 1982). In addition, syntenic gene clusters encoding ribosomal proteins, ATPase subunits, and RNA polymerase subunits were a good support that the plastid genomes originated from cyanobacterial genomes (Ohta et al. 1997; Stoebe and Kowallik 1999), which is difficult to explain by convergent evolution. Monophyletic origin of Viridiplantae (green algae and plants), red algae, and glaucophytes has been established by phylogenetic analysis of proteins encoded by both chloroplast (Nelissen et al. 1995; Turner et al. 1999) and nuclear genomes (Nozaki 2005; Rodríguez-Ezpeleta et al. 2005), and these photosynthetic organisms are called Archaeplastida, meaning that they are descendants of an ancestral proto-alga, resulting from a single endosymbiosis (Adl et al. 2005). At the same time, all chloroplasts are believed to originate from a cyanobacterium near the root of cyanobacterial diversification. *Gloeomargarita lithophora* is a recently identified, possible candidate of the closest relative of chloroplast (Ponce-Toledo et al. 2017), among others. Endosymbiotic origin of mitochondria was also supported by the presence of DNA (Nass and Nass 1963), as well as phylogenetic analysis of ribosomal RNA, ribosomal proteins and various mitochondrial proteins (Gray 1992; Gray and Doolittle 1982). In the present study, we shall mainly focus on the origin of chloroplasts.

Curiously, many researchers believe that everything in the chloroplast originated from cyanobacteria, once the endosymbiotic origin of chloroplast was accepted. Some exceptions might be explained by horizontal gene transfers, but the general view remains the same, even though rigorous phylogenetic analysis has not been performed for all chloroplast proteins. The same is true in textbooks. See, for example, the best-seller textbook *Campbell Biology* that presents a simplistic diagram showing the serial endosymbioses of a proteobacterial cell and a cyanobacterial cell to form a mitochondrion and a chloroplast, respectively (Figures 7–16 in Campbell et al. 2014). This type of illustration dates from Goksøyr (1967) and Sagan (1967). To focus on the chloroplasts, the current illustrations of this kind invariably show two limiting membranes and internal thylakoid membranes that are inherited from a cyanobacterial endosymbiont down to the present-day chloroplasts. Illustration helps comprehension for students, but at the same time, gives a serious misunderstanding to non-specialists. It should be emphasized that membranes are not replicated by themselves. Obviously, it is the genes encoding the enzymes for the lipid biosynthesis but not the membranes themselves that are inherited. We can imagine various situations: if the enzymes involved in the biosynthesis of chloroplast membrane lipids are encoded by the chloroplast genome, then we might be able to say that the chloroplast membranes are inherited from the ancestral endosymbiont membranes. If the enzymes are encoded by the host genome, we have two possibilities: If the enzymes originated from the endosymbiont (endosymbiotic gene transfer or EGT), then, we may still say that the chloroplast membranes are inherited from the ancestral endosymbiont membranes. If, however, the enzymes are indigenous to the host or if they originated from organisms other than the endosymbiont, then, we cannot say that the chloroplast membranes are inherited from the ancestral endosymbiont. The problem of membrane heredity must be understood in the context of gene phylogeny.

Similar illustrations are also abundant in review articles (see, e.g., Archibald 2015, and many others). The tradition of using membrane topology in explaining the primary and secondary endosymbioses of chloroplasts dates from Whatley (1981) and Cavalier-Smith (1982), and membrane topology has been repeatedly used as a visually comprehensible explanation of the endosymbiotic origin of chloroplasts. All these authors must have been aware of the fact that the membranes are not inherited by themselves, but many biologists implicitly believed that the enzymes for the synthesis of chloroplast membranes must originate from cyanobacteria and their genes are now transferred to the nucleus by EGT. In this sense, the membranes have always been believed products of the endosymbiont but not the host.

Another aspect of the problem is related to the following two notions that are not usually distinguished: the endosymbiotic origin of chloroplasts and the monophyletic origin of Archaeplastida. The latter has been demonstrated by many phylogenomic analyses. The similarity of protein import machinery provides another line of evidence (Matsuzaki et al. 2004). This is not the problem to analyze in the present study. As demonstrated by many phylogenetic analyses, I admit the monophyletic origin of the three lineages of Archaeplastida, which share most of gene acquisitions that I will show in this study. Rather, I try to question whether the chloroplasts were acquired by the ancient eukaryotic host by a single event of endosymbiosis. Namely, I try to know whether all the numerous common traits of cyanobacteria and chloroplasts are good evidence for the endosymbiotic origin of chloroplasts. Similarity of membrane lipids, photosynthetic mechanisms, and gene expression systems in cyanobacteria and chloroplasts, are they evidence for the continuity of cyanobacteria and chloroplasts?

The chloroplast is the sole site of fatty acid synthesis in plants and algal cells (Mori et al. 2016; Ohlrogge et al. 1979; Somerville and Browse 1991), and this was considered a result of cyanobacterial endosymbiosis. Indeed, the *accD* gene encoding a subunit of prokaryotic acetyl-CoA carboxylase is encoded by the chloroplast genome of many plants and algae. The chloroplast genomes of various algae also encode additional genes for fatty acid synthesis, such as *acpP* (encoding acyl carrier protein, ACP) and *fabH* (encoding KAS III, the first condensing enzyme in fatty acid synthesis). Based on these findings, lipid biologists believe that the fatty acid synthesis system of chloroplast originates from cyanobacteria.

Plant lipid biochemists traditionally use the terms “prokaryotic pathway” and “eukaryotic pathway” of chloroplast lipid biosynthesis (originally discussed by Roughan and Slack 1982, Somerville and Browse 1991; but still used by Hori et al. 2015, Petroutsos et al. 2014, among others). The former is a pathway completed within the chloroplast, whereas the latter is a pathway involving cooperative actions of endoplasmic reticulum and chloroplasts. The endosymbiotic origin of chloroplast was a basis for the nomenclature “prokaryotic pathway” in a pioneering paper (Zepke et al. 1978), which cited Mereschkowsky (1905) to emphasize the similarity of cyanobacteria and chloroplasts.

After the initial use of “prokaryotic” in lipid research field, various enzymes have been identified in the lipid biosynthesis in the chloroplast without seriously discussing the relationship between the chloroplast and cyanobacterial systems, although we have repeatedly shown that galactolipids are synthesized by different systems in chloroplasts and cyanobacteria (Awai et al. 2014; Sato 2015; Sato and Awai 2016; Sato and Murata 1982). In a recent paper, Sato and Awai (2017) showed that chloroplasts and cyanobacteria have phylogenetically unrelated pathways of diacylglycerol synthesis involving the two acyltransferases and phosphatidate phosphatase, based on detailed and comprehensive phylogenetic analyses, using a comparative genomic database Gclust (Sato 2009), presenting homolog clusters including all protein sequences of representative prokaryotic and eukaryotic organisms. This technical advance resolved the problem in previous works that constructed phylogenetic tree with only cyanobacterial and chloroplast enzymes, because inclusion of homologs of many other organisms was necessary to test cyanobacterial origin of chloroplast enzymes.

In the present study, I extended phylogenetic analysis to most major enzymes involved in the lipid biosynthesis in the chloroplasts, as well as some additional chloroplast enzymes involved in photosynthesis, gene expression, and division. The results revealed complex origins of chloroplast enzymes, suggesting that the visually understandable membrane heredity relationship associated with the endosymbiosis diagram should be re-considered. As an alternative, I present a hypothesis of host-directed chloroplast formation to explain the complex phylogenetic relationships. Note that “chloroplast” rather than “plastid” is consistently used in the present study to focus on the photosynthetic function and thylakoid membranes of cyanobacteria and chloroplasts.

## Materials and methods

### Sequence data

Essentially, the data sources were identical to those described in previous reports (Sato and Awai 2017; Sato and Takano 2017). The protein cluster database Gclust (Sato 2009) as available at the web site http://gclust.c.u-tokyo.ac.jp (Gclust server) was used to retrieve the sequences of various enzyme families for phylogenetic analysis. Gclust software does not use a simple E-value cut-off in clustering proteins, but automatically determines an appropriate threshold for each protein family by estimating the entropy of distribution and the count of organisms. The validity of homology clustering by Gclust and the quality of the resultant clusters were evaluated in previous studies (Sato 2009; Sato and Awai 2017). I used Dataset Gclust2012_42 to obtain core sequences. In addition, Dataset Gclust2017R6 was used to add protein family members. For analysis of chloroplast-encoded proteins, Datasets CPBACT10 and CyanoClust 4 (Sasaki and Sato 2010), also available from the Gclust server, were used. For some enzymes of lipid biosynthesis that have homologs only in cyanobacteria and/or plants/algae within the Gclust database, additional homologs were searched using the BLAST service of the National Center for Biotechnology Information (NCBI) and retrieved therefrom.

The names of enzymes/genes analyzed in the present study are listed in Online Resource 1, with some additional information including enzyme functions.

### Phylogenetic analysis

The methods of sequence alignment and phylogenetic reconstruction were essentially identical to those used in previous papers (Sato and Awai 2017; Sato and Takano 2017). Briefly, homologous protein sequences were aligned by the software Muscle version 3.8.31 (Edgar 2004). The alignment was examined to remove distant sequences, and ill-aligned *N*- and *C*-termini were trimmed. Only sites having gaps in less than 20% of the total sequences were used for the calculation. Sequence manipulation was performed by the SISEQ software version 159.44 (Sato 2000), now available from the author’s laboratory web site (http://nsato4.sakura.ne.jp/).

Initial phylogenetic tree was constructed by the maximum likelihood (ML) method using the software PhyML version 3.1 (Guindon et al. 2010) (options were: -d aa –m LG –s BEST –b -5). Then, removal of distant sequences, trimming of both ends, re-alignment, and ML calculation were repeated to obtain a reasonably reproducible tree. Baysian Inference (BI) analysis was also performed using the software MrBayes version 3.2.6 (Ronquist et al. 2012). Another BI analysis (PB) was performed using PhyloBayes version 4.1 (Lartillot and Philippe 2004) with CAT-GTR model in very rare difficult cases (RpoB and RpoC) to check the results of ML and BI. CAT-GTR model was recommended as a default model in the PhyloBayes software development team, and could be a good control of the calculation by ML and BI. The command “bpcomp” was used to check convergence in PB. In most analysis, LG model was used in both PhyML and MrBayes calculations. LG model was selected by model selection software in our previous studies (Sato and Awai 2017; Sato and Takano 2017) and many recent studies. To make the calculation consistent, the same model was used throughout the present study. Other parameters in MrBayes were: rates = invgamma, ratepr = variable, ngen = 2,000,000 (up to 50,000,000). The parameters, samplefreq and burnin, were appropriately set depending on the value of ngen. The posterior probability of splitting was obtained by the default settings. Convergence was confirmed by the average standard deviation of split frequencies, which must be less than 0.01 in general. The high values of posterior probabilities were also a sign of convergence. I present phylogenetic trees in which major branching pattern was consistent in both ML and BI methods, especially with respect to the positions of cyanobacteria and chloroplasts. This is the criterion of a reliable tree in the present study. A composite result with ML and BI on BI tree is presented as a default, but in some cases (such as AccB and Tic20), only an ML tree is shown because BI calculation did not converge even after very long runs of calculation.

In other cases (such as ACP), in which phylogenetic calculation with many taxa did not give consistent results with BI and ML, and even with different runs of ML, a minimal set of taxa containing chloroplast proteins with the most related proteins was used for calculation. The position of the root was determined by setting a clearly identified outgroup (such as bacteria), or conveniently estimated using the R script of the MAD software (Tria et al. 2017). Graphical representation of the final trees was prepared by the software FigTree version 1.4.3 (http://tree.bio.ed.ac.uk/software/figtree/), followed by decoration by Adobe Illustrator version CS6. In the main text, only a summary of tree types is shown. All phylogenetic trees are presented in Online Resources 2–7.

### Distance analysis

As an index of gene acquisition time, relative stem length was estimated essentially according to the method of Pittis and Gabaldón (2016) for the analysis of acquisition times of mitochondrial proteins. The length of the stem (from the origin of the clade to the first diversification of the clade) and the length of the leaf (the median length of the clade) were measured in each of the phylogenetic trees for the green and red lineages, respectively, and named VS and VL for the green lineage, and RS and RL for the red lineage. The ratios VS/VL and RS/RL were used as relative stem length of the two lineages, respectively. The position of divergence in the green lineage (the last Viridiplantae common ancestor, or LVCA) corresponds to the divergence of green algal clade and streptophyte clade. The position of divergence in the red lineage (the last Rhodophyta common ancestor, or LRCA) corresponds to the divergence of Cyanidiophytina clade and Rhodophytina clade. Therefore, the measurement of the stems and leaves do not depend on selection of taxa, and provided robust measures as far as the phylogenetic analysis was valid. As a reference of evolutionary rate, the median branch length of a neighboring cyanobacterial clade (CL) was measured (if applicable), and the value CL/VL was evaluated. Since the branch lengths of *Prochlorococcus* and marine *Synechococcus* were very long, these species were excluded in calculating CL values. If both CL/VL and VS/VL are very small, then this could be a result of unusually large VL, or a high rate of evolution in the green lineage. A corresponding value CL/RL was not used, because estimation of RL was not as robust as estimation of VL, because of a small number of red algal taxa.

## Results

### Comprehensive phylogenetic analysis of chloroplast enzymes

**Fig. 1:**
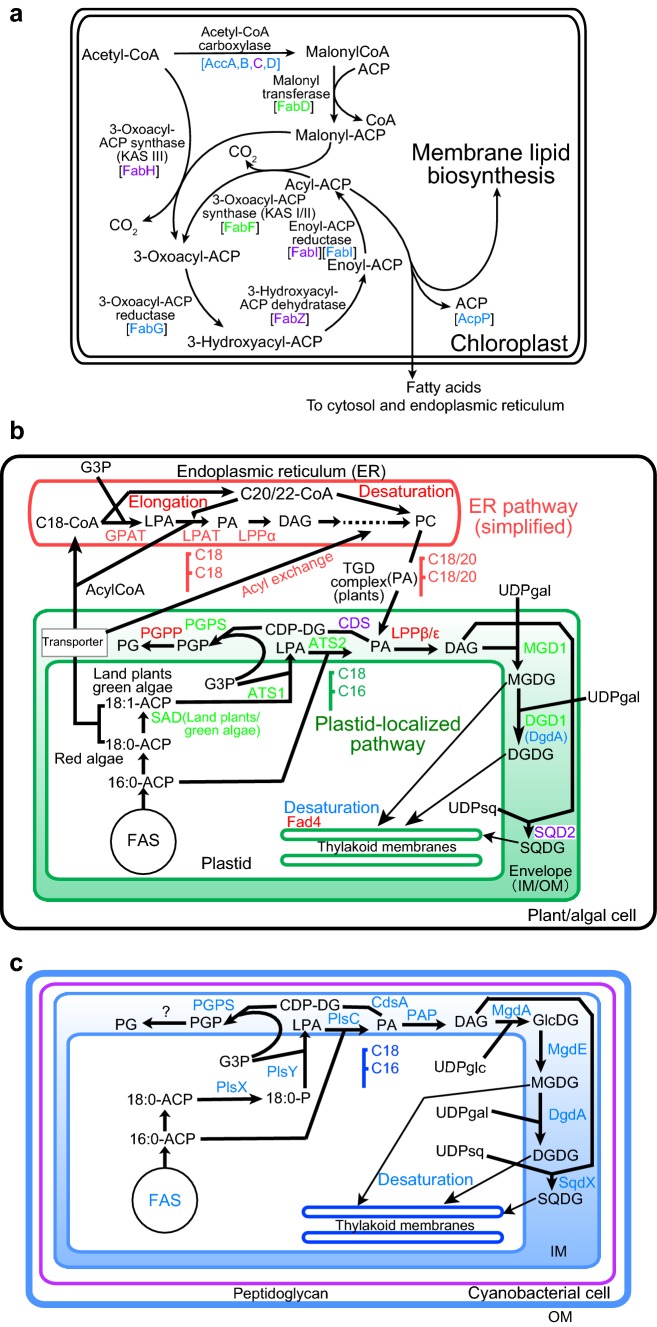
Diverse origins of enzymes in the pathways of fatty acid and lipid biosynthesis in chloroplasts and cyanobacteria. **a** Fatty acid synthesis in plants and algae. Gene-based enzyme names are color-coded according to the phylogenetic types defined in the text and Fig. 2. **b** Lipid biosynthesis in plants and algae emphasizing the localization within the cell. Gene-based enzyme names are color-coded according to the phylogenetic types defined in the text and Fig. 2. *ACP* acyl carrier protein, *G3P* glycerol 3-phosphate, *LPA* lysophosphatidic acid, *PA* phosphatidic acid, *DAG* diacylglycerol, *MGDG* monogalactosyl diacylglycerol, *DGDG* digalactosyl diacylglycerol, *UDPgal* UDP galactose, *UDPsq* UDP sulfoquinovose, *SQDG* sulfoquinovosyl diacylglycerol, *CDP-DG* CDP diacylglycerol, *PGP* phosphatidylglycerol phosphate, *PG* phosphatidylglycerol, *FAS* fatty acid synthetase, *CoA* coenzyme A, *PC* phosphatidylcholine, *IM* inner membrane, *OM* outer membrane. **c** Lipid biosynthesis in cyanobacteria. All enzyme names are colored blue. *UDPglc* UDP glucose, *GlcDG* monoglucosyl diacylglycerol

**Fig. 2:**
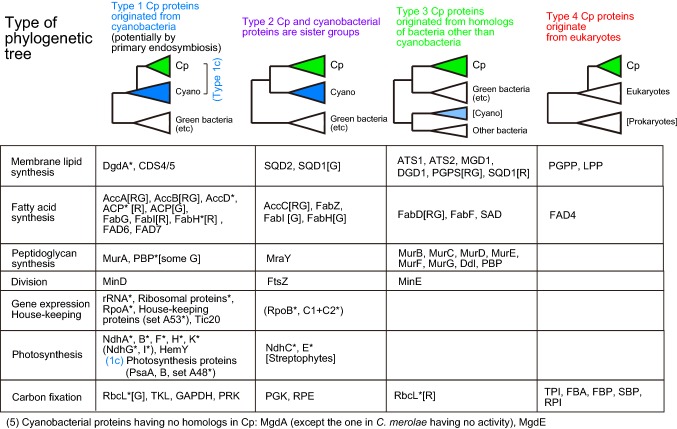
Four types of phylogenetic trees of chloroplast proteins. The color-codes of the four tree types are defined as shown and used throughout the present study. [G] and [R] indicate, respectively, green lineage (land plants + green algae) and red lineage (red algae, cryptophytes, stramenopiles). [RG] indicates that the two lineages are separated, but both present an identical tree type. Asterisk (*) indicates that the protein is encoded by the chloroplast genome. Proteins in parenthesis need special remarks on the tree topology, as follows: NdhG and I, overall tree topology of cyanobacteria is unusual. RpoB and C, both type 1 and type 2 topologies are found depending on methods. *Cp* chloroplast, *Cyano* cyanobacteria

I performed many phylogenetic analyses of chloroplast enzymes involved in fatty acid and lipid biosynthesis. The results are presented in color-codes indicating the origin of enzymes (as explained later) in a simplified pathway of fatty acid synthesis (Fig. 1a), as well as lipid biosynthetic pathways in plants/algae and cyanobacteria in Fig. 1b and c, respectively. Phylogenetic analysis was also performed with many chloroplast enzymes involved in photosynthesis, gene expression, and other functions. All phylogenetic trees obtained in the present study are presented in Online Resources 2–7, and a summary of phylogenetic analysis is presented in Fig. 2 to show phylogenetic tree type and Online Resource 1. The results of ATS1, ATS2, and LPP (Sato and Awai 2017), enzymes of peptidoglycan synthesis (Sato and Takano 2017), fatty acid desaturases FAD6 and FAD7 (Sato and Moriyama 2007), and protoporphyrin IX oxidase HemY (Kobayashi et al. 2014) are also included in Fig. 2. All other data are new in the present study. Four major types of phylogenetic trees were found:

- Type 1: Chloroplast enzymes diverged from inside of cyanobacterial clade.
- Type 2: Chloroplast and cyanobacterial enzymes are sister groups.
- Type 3: Chloroplast enzymes originated from homologs of bacteria other than cyanobacteria.
- Type 4: Chloroplast enzymes diverged from eukaryotic homologs.

Additionally, another type (type 5) is defined for cyanobacterial enzymes that have no homologs in chloroplasts. In all illustrations in the present study, a color-coding scheme is used consistently for the four types of phylogenetic trees: namely, blue for type 1, purple for type 2, green for type 3, and red for type 4.

### Lipid-biosynthesis enzymes

We begin by examining phylogenetic pattern of lipid-related enzymes. For some previously reported phylogenetic results, phylogenetic type is now assigned. Type 3 phylogeny was estimated for chloroplast acyltransferases, ATS1 and ATS2. Chloroplast phosphatidate phosphatase LPP was found to be a eukaryotic enzyme (type 4). I explain every phylogenetic tree in Online Resource 2 one by one. MGD1 originated from green bacteria. The presence of diatom sequences outside Archaeplastida clade is found in various phylogenetic trees in the present study. This is not easily explained by the secondary endosymbiosis. However, I prefer to limit the current analysis to the problem of primary endosymbiosis. The problem of diatoms will be described at the end of “Discussion”. I also find a proteobacterial (*Aromatoleum*) sequence in the Archaeplastida clade, but such occurrence of sporadic bacterial sequences is considered a result of horizontal gene transfer, and excluded from the main discussion in the present study.

The eukaryotic enzyme for the synthesis of digalactosyl diacylglycerol (DGDG), DGD1, is now known to have prokaryotic homologs in some proteobacteria, which were detected by the BLAST search in the current public database (but not in the genome sets in the Gclust database). Nevertheless, it is not clear if these bacterial enzymes were the origin of eukaryotic DGD1, or vice versa, because the tree is not rooted by an unambiguous criterion. DGD1 is tentatively assigned to type 3 phylogeny. MgdA also has some homologs in some bacteria, but it is still difficult to find the origin. A similar situation is found for MgdE, which was hitherto believed to be present in only cyanobacteria. Cyanobacterial monogalactosyl diacylglycerol (MGDG) synthesis enzymes, MdgA and MgdE, are classified to type 5 (no homologs in eukaryotes). DgdA, a chloroplast-encoded galactosyltransferase for DGDG synthesis in *Cyanidioschyzon merolae* and *Galdieria sulphuraria*, showed type 1 phylogeny. Note that other red algae have a DGD1, a plant/algal type of DGDG synthesis enzyme but not DgdA.

CDP diacylglycerol synthases of the chloroplast, CDS4/5 (but not CDS1/2 in the endoplasmic reticulum), originate from cyanobacteria, although exact phylogenetic positions of red algal enzymes and enzymes of the green lineage changed with calculation methods. Obviously, a phylogenetic tree in which the two lineages are associated (BI and ML trees) is more likely. Enzymes involved in phosphatidylglycerol (PG) synthesis, PGPS and PGPP, did not originate from cyanobacteria (types 3 and 4, respectively). Note that eukaryotic cardiolipin synthase (CLS) is related to prokaryotic PGPS (PgsA), but not to prokaryotic CLS (not shown). This is interesting in considering mitochondrial origin, but I do not argue about it any further in the present study on the chloroplast origin. Sulfoquinovosyl diacylglycerol (SQDG) synthase SQD2/SqdX presented a type 2 tree. The phylogenetic tree of UDPsulfoquinovose synthesis enzyme SQD1/SqdB was complex. Cyanobacterial enzymes were split into two major clades with an additional small group. The enzymes of the green lineage and β-cyanobacteria originate from green bacterial enzymes (type 2), whereas the enzymes of the red algae and α-proteobacteria attached with the enzymes of α-cyanobacteria. The tree of red algal SQD1 is tentatively classified as type 3.

### Fatty acid synthesis

Phylogenetic trees of individual enzymes in fatty acid synthesis are presented in Online Resource 3. Subunits of acetyl-CoA carboxylase, AccA, AccB, and AccD, presented phylogenetic relationship type 1, although exact tree shape of AccB was dependent on analytical methods. In AccA and AccB, the green and red lineages diverged from different points within the cyanobacterial clade. This is indicated as [RG] in Fig. 2. AccC showed type 2 phylogeny. Acyl carrier protein (ACP) of chloroplasts unambiguously originated from cyanobacteria, but red algal chloroplast-encoded ACP and ACP of the green lineage were split within the cyanobacterial clade in both full and simplified trees (Online Resource 3, pages 6–8). Mitochondrial ACP is related to α-proteobacteria.

FabF (KAS I and II: note that FabB is a variant of FabF present in some bacteria), originated from green bacteria (type 3). Two isozymes, KAS I and KAS II, known in green algae and land plants diverged at the root of green lineage after its separation from the red lineage. Mitochondrial KAS members are closely associated with α-proteobacterial FabF. Chloroplast FabD is likely to originate from some bacteria other than cyanobacteria (type 3), but it is difficult to identify exact phylum of the origin. FabG presented phylogenetic relationship type 1. FabI in the green and red lineages split into two clades: the red lineage enzymes within cyanobacterial clade (type 1) and the green lineage enzymes outside cyanobacteria (type 2), respectively. The FabZ tree was type 2. In the FabH (KAS III) tree, chloroplast-encoded enzymes in the red lineage originated from cyanobacteria (type 1), whereas the homologs of the green lineage were a sister group of cyanobacteria (type 2).

Fatty acid desaturase involved in the desaturation of *sn*2-palmitoyl group of phosphatidylglycerol (FAD4) is found only in photosynthetic eukaryotes, and related to ubiquitin-ligating enzyme E2 (type 4). Stearyol ACP desaturase (SAD), a stromal enzyme involved in the synthesis of oleoyl ACP, is present in the green lineage. Bacterial homologs are found in mainly Bacteroidetes group. Curiously, homologs in diatoms are found within the clade of Bacteroidetes. SAD is likely to belong to type 3 phylogeny. Previous results showing that chloroplast delta 12 desaturase FAD6 originated from cyanobacterial DesA and that chloroplast delta 15 desaturase FAD7 originated from cyanobacterial DesB (Supplementary Fig. 13 in Sato and Moriyama 2007) were confirmed by re-calculation in Online Resource 3, page 17.

### Phylogeny of enzymes encoded by the chloroplast genome

We now characterize phylogenetic relationships of other chloroplast enzymes to compare with lipid-related enzymes. In this section, we examine enzymes and rRNA encoded by the chloroplast genome (Online Resource 4). Type 1 tree was typically found in the phylogenetic analysis of ribosomal RNA (rRNA) (including both 16S and 23S rRNA), and this is exactly expected from the endosymbiotic theory. Photosynthesis-related proteins encoded by the chloroplast genome (green and red lineages) were analyzed as concatenated sequences (set A48, including 23 proteins in 48 organisms). A type 1 tree was found, although no bacterial outgroup is available. For this reason, it was placed in the category type 1c. The trees of PsaA and PsaB can be rooted, however, by using either of them as an outgroup as shown in Online Resource 4 (pages 3–5). Another set of photosynthesis-related proteins conserved in red algal chloroplast genomes (set B37, including 24 proteins in 37 organisms) presented also a type 1c tree, when analyzed as concatenated sequences.

Conserved house-keeping proteins in the chloroplast genomes of green and red lineages were also analyzed as concatenated sequences (set A53, including 23 proteins in 53 organisms). A type 1 tree was obtained with bacterial proteins as an outgroup. House-keeping proteins conserved in red algal chloroplast genomes (set B42, including 53 proteins in 42 organisms) showed a type 1 tree, when analyzed as concatenated sequences. Individual ribosomal proteins showed various aberrant trees (not shown), maybe because their short sequence lengths do not contain enough phylogenetic signals. However, when nine ribosomal proteins that showed aberrant trees in individual trees were concatenated, a type 1 tree was found (set A53 selected or A53-2). A similar tree, obtained with the 33 proteins (Tajima et al. 2016: hereafter nicknamed “33P”), was re-analyzed with the LG model instead of WAG model. Conserved proteins in red algae (5 and 3 species, respectively) and cyanobacteria (set B37 and set F35) also showed type 1c trees.

Many of these type 1 trees showed a deep origin of chloroplast clade in the cyanobacterial phylogeny, either before or after the separation of the Yellowstone strains of *Synechococcus*. In the type 1 trees of PsaA and PsaB, however, chloroplast clade diverged later, having the *Synechococcus*–*Prochlorococcus* clade (α-cyanobacteria) as a sister group.

The Ndh proteins (Online Resource 5) that are conserved in the chloroplast genomes of certain green algae and land plants (Streptophyta) presented a type 1 tree, when analyzed as concatenated sequences (Ndh_all, page 4). NdhD and NdhJ were not included in this analysis, because NdhD has many paralogs, and NdhJ is not present in *Nephroselmis*. Individual trees of these proteins (both type 1) are presented at the end of Online Resource 5 (pages 2, 3, 5, and 6). NdhC and NdhE showed type 2 trees. NdhG and NdhI also showed strange trees with unusual cyanobacterial phylogeny when analyzed individually. Nevertheless, these data are not strong enough to reject that Ndh proteins originate from inside of cyanobacterial clade (type 1). More studies are necessary on the phylogeny of Ndh proteins.

Chlorophyll biosynthesis enzymes, ChlB, ChlL, and ChlN (set E48 in Online Resource 4, page 14), were analyzed as concatenated sequences. Cyanobacterial proteins are split into two major clades of α-cyanobacteria and β-cyanobacteria. Chloroplast-encoded proteins originated from β-cyanobacteria (type 1). Another enzyme involved in tetrapyrrole synthesis, HemY, also showed a type 1 phylogeny, although the majority of cyanobacteria possess an unrelated, isofunctional enzyme HemJ.

### Phylogeny of chloroplast enzymes involved in other functions

Two nuclear-encoded photosystem proteins, PsaD and PsaE, were analyzed (Online Resource 4, pages 8 and 9) to compare with PsaA and PsaB. PsaD showed a type 1c tree, whereas, in the PsaE tree, red algal proteins and green lineage proteins diverged separately within the β-cyanobacterial clade. This anomaly could be due to the small size of PsaE proteins.

Phylogenetic trees of chloroplast-encoded RNA polymerase subunits, RpoB and RpoC (note that bacterial RpoC is split into two separate parts C1 and C2 in cyanobacteria and chloroplasts) are shown in Online Resource 4, pages 17–20. The results depended on analytical methods: type 1 trees in some analyses (RpoB by BI and PB, RpoC by PB), and type 2 trees in others (RpoB by ML, RpoC by ML and BI). RpoC was analyzed with or without land plant sequences, but the results were not different. RpoA presented a type 1 tree in all analyses (Online Resource 4, page 16). Curiously, in the RpoA tree, chloroplast clade diverged late from nitrogen-fixing β-cyanobacteria.

**Fig. 3:**
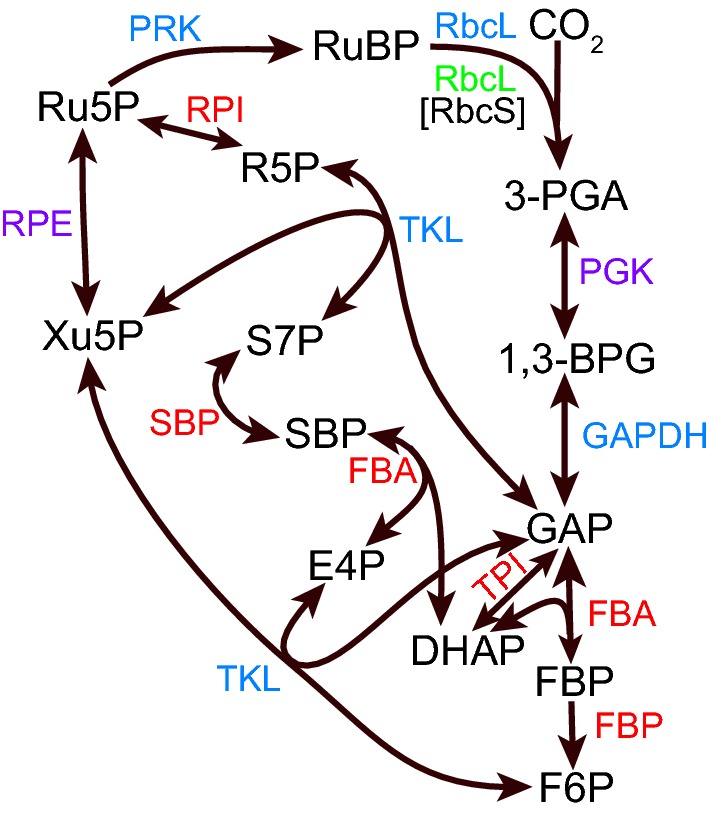
Diverse origins of Calvin-Benson cycle enzymes. Gene-based enzyme names are color-coded according to the phylogenetic types defined in the text and Fig. 2, except RbcS, which was not analyzed in the present study. Substrate names: *RuBP* ribulose 1,5-bisphosphate, *3-PGA* 3-phosphoglycerate, *1,3-BPG* 1,3-bisphosphoglycerate, *GAP* glyceraldehyde 3-phosphate, *DHAP* dihydroxyacetone phosphate, *FBP* fructose 1,6-bisphosphate, *F6P* fructose 6-phosphate, *E4P* erythrose 4-phosphate, *SBP* sedoheptulose 1,7-bisphosphate, *S7P* sedoheptulose 7-phosphate, *Xu5P* xylulose 5-phosphate, *R5P* ribose 5-phosphate, *Ru5P* ribulose 5-phosphate. Enzyme names: *RbcL and RbcS* ribulose 1,5-bisphosphate carboxylase/oxygenase large and small subunits, respectively; *PGK* phosphoglycerate kinase, *GAPDH* glyceraldehyde 3-phosphate dehydrogenase, *FBA* fructose 1,6-bisphosphate aldolase (also acting with sedoheptulose bisphosphate), *FBP* fructose 1,6-bisphosphatase, *TPI* triose 3-phosphate isomerase, *TKL* transketolase, *SBP* sedoheptulose 1,7-bisphosphatase, *RPI* ribulose 5-phosphate isomerase, *RPE* ribulose 5-phosphate epimerase, *PRK* phosphoribulokinase

Calvin-Benson Cycle enzymes (Fig. 3) have been known to originate from different sources (Martin and Schnarrenberger 1997; Tabita 1999; Matsuzaki et al. 2004). Re-analysis of phylogenetic trees of these enzymes indicated that chloroplast-encoded RbcL (Online Resource 4, page 15) in the green lineage, as well as nuclear-encoded GAPDH, PRK, and TKL, showed type 1 trees, whereas PGK and RPE showed type 2 trees (Online Resource 6). RbcL in the red lineage (Online Resource 4, page 15) originate from proteobacteria (type 3 tree) as already known. It is known in a previous study (Matsuzaki et al. 2004) that TPI, FBA, FBP, SBP, and RPI are of the eukaryotic origin (type 4 tree). Note, however, that, in the PRK tree, α-cyanobacteria and β-cyanobacteria formed distinct clades, and that the chloroplast clade diverged from the β-cyanobacterial clade. RbcL in the green lineage also originated from β-cyanobacteria. The origin of chloroplast PRK and RbcL is, therefore, different from that of other enzymes of cyanobacterial origin.

Peptidoglycan synthesis enzymes were already analyzed in Sato and Takano (2017). Most of them presented type 3 trees, whereas a type 2 tree was inferred for MraY, and a type 1 tree for MurA. Chloroplasts also have prokaryotic division proteins, FtsZ, MinD, and MinE (Online Resource 7). The FtsZ tree constructed in the present study was essentially similar to the one presented in Grosche and Rensing (2017), confirming a type 2 phylogeny. MinD showed a type 1 tree, while the putative partner MinE showed a type 2 or type 3 tree. Curiously, chloroplast MinD proteins were sister to the α-cyanobacterial clade.

Translocon components were also analyzed. As already described (Matsuzaki et al. 2004), only limited core components of the translocon have bacterial homologs. Tic20 showed a type 1 tree, although Archaeal homologs are present between its two isozymes. Tic21, Tic22, and Toc75 also presented type 1 trees in re-analysis (not shown), and this is consistent with the previous results (Matsuzaki et al. 2004).

The results of phylogenetic analysis for lipid-related enzymes and Calvin–Benson Cycle enzymes are shown in Figs. 1 and 3, respectively, with color-codings.

### Acquisition times of chloroplast proteins

**Fig. 4:**
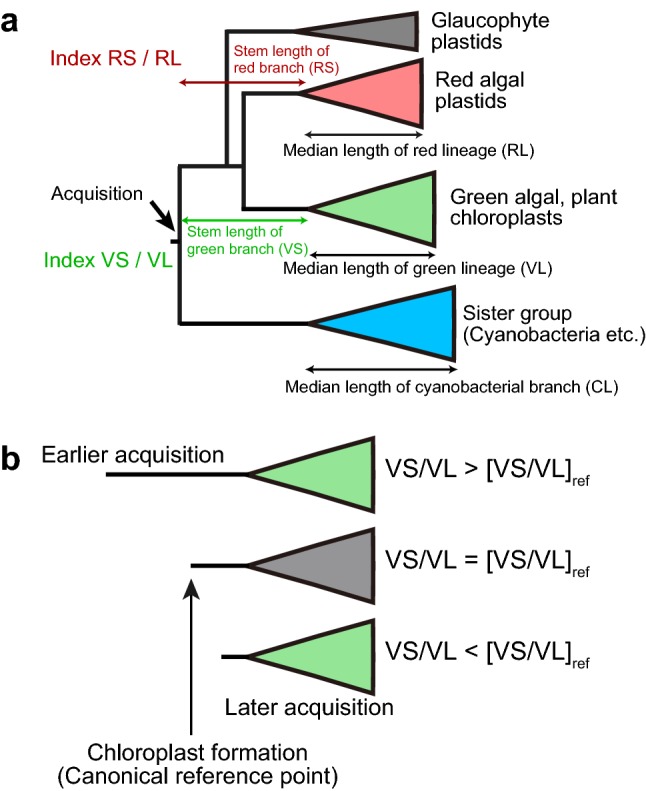
Estimation of relative stem length. **a** Measurement of the stem and leaf lengths of the red and green lineages. The median length of the green lineage was taken as the leaf length of the green lineage (VL, for Viridiplantae length), whereas the corresponding length of the red lineage was taken as RL (for Rhodophyta length). *VS* Viridiplantae stem, *RS* Rhodophyta stem, *CL* cyanobacterial branch length. **b** The ratio VS/VL was taken as a measure for gene acquisition time in the green lineage. An enzyme that was acquired earlier than the canonical reference point presents a larger value of VS/VL than [VS/VL]_ref_. A similar relationship is applied for the red lineage with RS/RL

I tried to estimate acquisition time of individual proteins (or rRNA) according to the method of Pittis and Gabaldón (2016) used for mitochondrial proteins (Fig. 4a). Acquisition time was separately estimated for the enzymes in the red and green lineages, partly because inclusion of *Cyanophora*, a single taxon currently available in the Glaucophyta, was not always possible, and partly because the origins of enzymes in the two lineages could be different, as known for RbcL. Assuming a constant rate of evolution, the acquisition time of an enzyme before the diversification of the green or red lineage can be estimated by a ratio of the stem length (VS or RS) and the leaf length (VL or RL) of the phylogenetic tree (V and R stand for Viridiplantae and Rhodophyta, respectively). The leaf length was measured as a median of various taxa as described in the original method. The geometric mean of the values obtained with ML and BI trees was used for each protein in further analysis.

If the acquisition time of an enzyme is earlier than that of a reference enzyme, then the value VS/VL or RS/RL will be larger than the reference value (Fig. 4b). The values VS/VL and RS/RL must be correlated, if the origin of the enzyme is common in the two lineages and the two lineages diverged from a common ancestor.

**Fig. 5:**
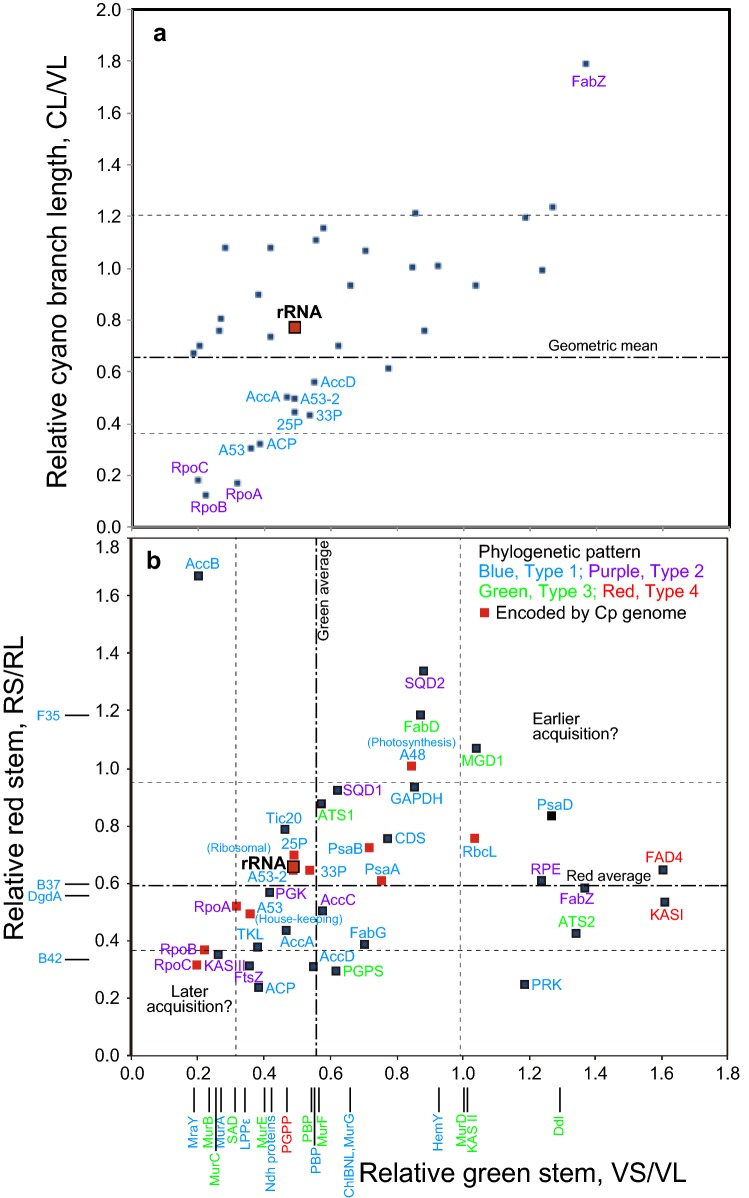
Distribution of relative stem lengths of chloroplast proteins and rRNA in the green and red lineages. **a** Distribution of CL/VL versus VS/VL values. **b** Distribution of RS/RL versus VS/VL values. The horizontal axis shows relative stem length of the green lineage (VS/VL), whereas the vertical axis shows relative cyanobacterial branch length (CL/VL) in (**a**), and relative stem length of the red lineage (RS/RL) in (**b**). In **a**, CL/VL is expected to be near 1.0 if evolutionary rate is similar in the cyanobacterial sister group and the green lineage. Protein names are marked for only outliers. In **b**, data only in the green lineage are shown near the horizontal axis, whereas data only in the red algal enzymes are shown near the vertical axis. All enzyme names (or dataset names) are color-coded according to the four types of phylogenetic relationship as defined in Fig. 2. Data symbols for the enzymes encoded by the chloroplast genome are shown in red. Geometric means of all data in the two axes are shown as “Green average” and “Red average”. The dotted lines show average plus/minus standard deviation

The results are summarized in Fig. 5b, in which VS/VL values are plotted in the horizontal axis, and RS/RL values in the vertical axis. The values of enzymes that are only present in one of the red and green lineages are plotted along the respective axes. As a general trend, the values VS/VL and RS/RL are correlated and many data points are arranged along or near the diagonal. The original data are available in Online Resource 8.

I tried to determine a reference point that represents a canonical origin of chloroplast genes (Table 1). Several measurements of the rRNA data with different taxa and methods yielded VS/VL and RS/RL values about 0.49 and 0.66, respectively. Because these are ratios, geometric means were calculated. Curiously, two published rRNA trees (Marin et al. 2005; Ponce-Toledo et al. 2017) gave larger values, maybe because phylogenetic methods were not identical to that used here. Nevertheless, ribosomal protein tree in A53-2 (Online Resource 4), as well as the tree published in our previous paper (data 33P, Tajima et al. 2016), and the tree shown in Shih et al. 2013 (data 25P), gave values comparable with those obtained with rRNA as above. Unfortunately, again, another protein tree gave larger values (Ponce-Toledo et al. 2017). In addition, geometric means of all indices (including overlapping data) obtained in the present study were 0.546 and 0.594 for VS/VL and RS/RL, respectively, which were similar to the respective values obtained above for rRNA. This estimate of average might not be very accurate, because data are not completely independent, and the weight of each value is not identical. However, approximate coincidence of these values obtained by fundamentally different methods suggest that the values about 0.5 and 0.6 for VS/VL and RS/RL, respectively, can be regarded as representative values for the origin of canonical genes and proteins in the chloroplast, either encoded by the chloroplast or nuclear genomes.

**Table 1:** Estimation of a reference point of chloroplast gene acquisition

Sequences	References	Methods	VS/VL	RS/RL	CL/VL
Cp rRNA (16S + 23S)	This study	BI (pair, 64 taxa, v3.1.2)	0.489	0.654	0.744
		BI (pair, 73 taxa, v3.1.2)	0.455	0.584	0.830
		BI (pair, 73 taxa, v3.2.6)	0.467	0.619	0.764
		ML (GTR, 73 taxa)	0.549	0.793	0.745
		Geometric mean	0.489	0.658	0.770
	Marin et al. (2005)	ML (GTR)	0.646	1.086	0.780
	Ponce-Toledo et al. (2017)	ML (GTR)	1.048	1.984	N/A
Cp proteins	This study (A53sel)	BI, ML (LG, v3.2.6)	0.488	0.643	0.499
	Tajima et al. (2016) (33P)	BI (v3.2.6, recalculated with LG model)	0.529	0.646	0.430
	Shih et al. (2013) (25P)	ML (LG)	0.488	0.697	0.445
	Ponce-Toledo et al. (2017)	PB (CAT-GTR)	0.959	0.846	0.325
All indices	This study	Geometric mean	0.546	0.594	0.656

*Cp* chloroplast, *BI* Bayesian Inference (v3.1.2 and v3.2.6 are versions of MrBayes), *ML* maximum likelihood, *GTR* general time-reversible, *PB* Bayesian Inference with PhyloBayes

With these canonical values as a reference, it is surprising to find wide variation in the values of VS/VL and RS/RL in Fig. 5b. In this figure, data labels are color-coded to show phylogenetic origin (types 1, 2, 3, or 4). In addition, each symbol of chloroplast-encoded protein is highlighted in red. Even the chloroplast-encoded proteins or proteins of cyanobacterial origin (type 1 or type 2) gave highly scattered distribution of VS/VL and RS/RL. As a general trend, enzymes involved in fatty acid and lipid biosynthesis (SQD1, SQD2, MGD1, ATS1, ATS2, CDS, FabD, FabZ, FAD4, AccB, KAS I, and KAS II) and those involved in photosynthesis (RbcL, PsaD, PRK, RPE, GAPDH, PsaA, PsaB, and set A48) gave larger values for both parameters or at least one of them. HemY, which is involved in chlorophyll synthesis in the green lineage showed a larger value of VS/VL. In contrast, RpoB, RpoC, KAS III and FtsZ gave significantly smaller values for both parameters. Subunits of peptidoglycan synthesis that are conserved in the green lineage (and *Cyanophora*, which is not a target of the present analysis) showed diverse values of VS/VL. Interestingly, MurA, which originate from cyanobacteria, and MraY, which is sister to cyanobacterial homologs, showed very small values of VS/VL, suggesting that they were acquired later than rRNA. The enzymes with large values of VS/VL and RS/RL could be acquired earlier than the acquisition of rRNA.

Variation in VS/VL and RS/RL values could result from uneven rate of evolution. Relative evolutionary rate of the cyanobacterial branch (CL/VL) is taken as a reference (Fig. 5a). Most values of CL/VL distributed between 0.6 and 1.2 without correlation with VS/VL values. Nevertheless, the data points for RpoA, RpoB, and RpoC were clearly outliers: both CL/VL and VS/VL were very small for these enzymes, indicating that the evolutionary rate was higher in the branch of green lineage (VL). The fact that RS/RL was also small for RpoB and RpoC (Fig. 5b) suggests that evolutionary rate was also high in the branch of red lineage. AccA, AccD, ACP, as well as some concatenated data, presented also small values of CL/VL. The relative stem values for these could also be affected by the rapid evolution in the chloroplasts, although the effect might not be large.

## Discussion

### Diverse origins of chloroplast enzymes

The present study provided two different criteria for assessing the origins of chloroplast enzymes involved in membrane lipid synthesis, photosynthesis, gene expression, among others. The first criterion is phylogenetic branching, which has also been used to show chloroplast enzymes of cyanobacterial origin before. However, many proteins involved in photosynthesis are conserved in only cyanobacteria and chloroplasts, and it has been theoretically difficult to determine exactly whether the chloroplast enzymes originate from cyanobacteria, or chloroplast and cyanobacterial enzymes are sister groups (type 1c). The combined tree of PsaA and PsaB showed unambiguously that a *Gloeobacter* homolog is at the base of each lineage. Based on this result, we can safely, but tentatively, set the root with *Gloeobacter* as the first branching species for type 1c trees. Figure 2 shows four major types of phylogenetic relationship of chloroplast proteins with cyanobacterial homologs. As described in “Results”, some enzymes such as RpoB, RpoC, and Ndh proteins showed inconsistent results depending on taxon selection and phylogenetic methods, but most proteins analyzed in the present study showed consistent branching pattern with both ML and BI methods. In this respect, the classification of enzyme origins in Fig. 2 is reliable, and provides the basis of further consideration on evolutionary history. Type 1 phylogeny could be further classified according to the origin within the cyanobacterial clade. Many type 1 enzymes had a deep origin in the cyanobacterial clade after the Yellowstone strains, but, as described above, PsaA, PsaB, and MinD originated from α-cyanobacteria, whereas RbcL[G], PRK, RpoA, and ChlBLN originated from β-cyanobacteria. This suggests that even the enzymes of cyanobacterial origin could have different origins within cyanobacteria (see below).

Another criterion is relative stem length as defined in Fig. 4. I used a method previously applied to mitochondrial enzymes (Pittis and Gabaldón 2016), but stem length was estimated for each of the green and red lineages. This is justified by two reasons. First, to estimate the stem length of Archaeplastida as a whole, we need sequences in all three lineages, namely, the green, red, and glaucophyte lineages. In many phylogenetic trees in which a glaucophyte sequence is available, glaucophyte diverges first. In this respect, the estimate of the stem length depends on the presence of glaucophyte sequence. In addition, *Cyanophora paradoxa* was the only species of glaucophyte whose genomic data were available until recently. The estimation of stem length of all Archaeplastida is not always possible with the original *Cyanophora* genome data published in 2012 from Rutgars University. A new data published recently (Price et al. 2019) might be useful in future studies.

The second reason to estimate stem length for the green and red lineages was the fact that not all enzymes are perfectly conserved in both green and red lineages. As stated above, Ndh proteins, enzymes involved in peptidoglycan synthesis, and chlorophyll biosynthesis enzymes are present in the green lineage, but not in the red lineage. In contrast, many proteins in the dataset B37, B42, and F35 are conserved in the red lineage but not in the green lineage. I therefore prefer to estimate the stem length for each of the green and red lineages in all enzymes or datasets. Nevertheless, the high correlation of the two values in Fig. 5b indicates that the analysis was successful in general. Extreme values were found for some small proteins, but we can use the distribution of data points in further discussion.

These two criteria, namely, branching pattern and relative stem length, suggested that chloroplast proteins, whether they are encoded by the chloroplast or nuclear genomes, are phylogenetically highly diverse.

### Diversity in gene acquisition times

I have to admit that there are possible pitfalls in the estimation of relative stem length. First, the estimate could be liable to tree shape. If tree shape is not unambiguously fixed, it is difficult to determine stem lengths. In most of my analyses, the branching pattern of the chloroplast clades was supported by both ML and BI methods as described above. I made many more trees that are not presented in Online Resources during the process of the present study, and confirmed the validity of the presented trees unless otherwise stated. Curiously, however, different trees of RpoB and RpoC gave similar values of VS/VL, RS/RL, and CL/VL. Second, selection of taxa can affect estimation of relative stem length, especially in determining leaf lengths, VL and RL. As described above, these values were determined as a median of branch lengths in the respective subtree. In the case of green lineage, I was able to use enough taxa to obtain a stable value of median, namely, removing or adding a taxon does not significantly affect the value of VL. In the case of red lineage, however, the number of available taxa was limited to five or six, depending on enzymes. Addition or removal of a taxon can affect RL value significantly in some enzymes. This could result in lower or higher estimate of RS/RL values as illustrated in Fig. 5b, such as AccB, F35, and B42. Third, relative stem length might not reflect actual acquisition time in the evolutionary history. If evolutionary rate was not identical for the time of diversification (VL or RL) and for the time from the acquisition to the common ancestor (VS or RS), the ratio VS/VL or RS/RL does not reflect the ratio of the two times.

I have two ideas to overcome this problem of evolutionary rate. First, the length of cyanobacterial sister group could provide a measure of changes in evolutionary rate. According to this criterion, the small values of VS/VL and RS/RL of RNA polymerase subunits were interpreted as a result of higher rate of evolution in the chloroplast branch. Second, we can assume that the selection pressure of functionally related proteins is similar. This might be true for the groups of enzymes involved in fatty acid synthesis, lipid biosynthesis, photosynthesis, protein synthesis, transcription, and carbon fixation. In this respect, highly diversified values of relative stem lengths obtained with functionally related proteins in each functional group could represent real differences in acquisition time of the enzymes.

Acquisition of chloroplast rRNA and house-keeping proteins (including ribosomal components and ATPase subunits, and possibly RNA polymerase subunits) (Table 1) may be taken as a canonical time of acquisition of chloroplast genes. The fact that these house-keeping proteins form discrete gene clusters within the chloroplast genome (Stoebe and Kowallik 1999) suggests that the clusters were introduced into the chloroplast in formation as a single unit. In my data, it is not clear whether RNA polymerase was acquired with ribosome. The argument on evolutionary rate as described above might not be sufficient for proving that RNA polymerase was acquired with ribosome, because the phylogenetic origin of RpoB and RpoC is not clearly resolved (either type 1 or type 2). Another concern is that, even in type 1 trees, the exact origins of RpoA, RpoB, and RpoC within the cyanobacterial clade were diverse. Further work is needed to solve the origin of RNA polymerase.

### Potentially early acquisition of glycolipid synthesis enzymes

Acquisition of lipid biosynthetic ability by the chloroplast is divergent in origin and time. Origins of enzymes involved in glycolipid and phosphatidylglycerol synthesis are quite diverse, as shown by type 3 and type 4 phylogenies. The enzymes were potentially acquired earlier than the acquisition of chloroplast ribosome as shown by longer relative stem lengths. These include fatty acid synthase, KAS I/II, malonyltransferase, FabD, the second acyltransferase, ATS2, eukaryotic MGDG synthase, MGD1, and palmitoyl desaturase of phosphatidylglycerol, FAD4. Some type 2 enzymes also presented longer relative stem lengths. These include dehydratase FabZ, the two enzymes in SQDG synthesis, SQD1/SqdB and SQD2/SqdX. Type 3 enzymes also include the first acyltransferase, ATS1, and the second galactosylation enzyme, DGD1. Acquisition of all these components of lipid biosynthetic machinery by chloroplast must be different from the origin of chloroplast ribosomes, in both time and source. According to the larger values of relative stem length, it is quite possible that many of the lipid biosynthetic enzymes had been acquired before the acquisition of ribosomes. By contrast, DgdA, a chloroplast-encoded galactosyltransferase to produce DGDG in Cyanidiales red algae, originate from cyanobacteria (type 1) and presented a comparable value of RS/RL with rRNA, suggesting that it is acquired from cyanobacteria with ribosomal components. It is unlikely that DGD1 was acquired independently in the other red algae and the green lineage. Therefore, DGD1 in the red algae other than Cyanidiales must be acquired before the separation of the red and green lineages, suggesting that DGD1 and DgdA co-existed in early red algae, and possibly in early Archaeplastida.

Early acquisition of SQDG and MGDG synthesis activities could provide advantages for a proto-algal cell, because phosphorus is a limiting element in aquatic environments. Phosphorus is an important element for all organisms, but its abundance is limited in the Earth crust, and much more limited in the seawater. Photosynthetic membranes of plants and algae are made of glycolipids and the content of phosphatidylglycerol is limited to about 10%. If the membranes should be made entirely of phospholipids, phosphorus requirement of plants and algae would be much higher. In many green algae, such as *Chlamydomonas reinhardtii*, phosphatidylcholine (PC) is replaced by diacylglyceryltrimethylhomoserine (DGTS) (Sato and Furuya 1985). Phosphate limitation is known to reduce phosphatidylcholine content and to increase DGTS content in some fungi (Senik et al. 2015). The use of glycolipids instead of phospholipids must be an adaptation of photosynthetic organisms to phosphorus-limited environments. The same argument must also be valid for early microorganisms living in phosphorus-limited water. The acquisition of SQDG and MGDG synthesis activities (possibly, DGDG synthesis, as well) could have provided selective advantage in early microorganisms, even before the acquisition of photosynthetic activity. As an interesting supporting evidence, phosphate limitation in *Arabidopsis thaliana* causes accumulation of DGDG (normally a chloroplast lipid synthesized in the envelope membranes) to the extraplastidic compartment to replace phospholipids (Härtel et al. 2000). This could be reminiscent of the ancient situation in the host cell before the chloroplast formation.

### Acquisition of photosynthetic machinery

**Fig. 6:**
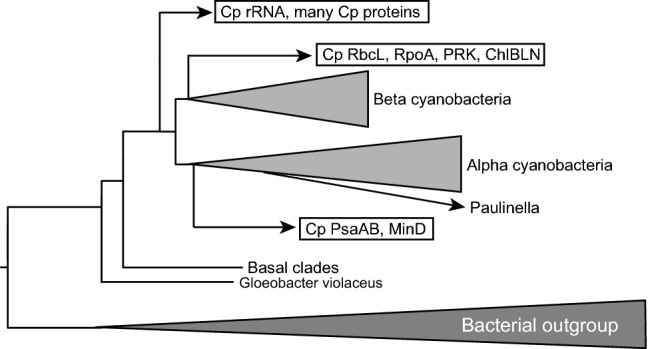
Multiple origins of chloroplast genes/enzymes within the cyanobacteria. The phylogenetic tree of rRNA (Online Resource 3, page 2) is shown schematically as a basic framework. The results of phylogenetic analysis as summarized in Online Resource 1 indicate that chloroplast genes/enzymes that are known to originate from cyanobacteria have three distinct origins within the cyanobacteria. This is evidence for multiple acquisitions of chloroplast genes from cyanobacteria

The origin(s) of photosynthetic machinery might be more complicated than through a single endosymbiotic event. Most of the photosynthesis-related components analyzed in the present study originated from cyanobacteria (type 1). Nevertheless, as described above, the exact origins of individual components are not identical. As already known, most chloroplast-encoded proteins have a deep origin within the cyanobacterial clade: namely, chloroplast proteins diverged early after the diversification of some basal groups (Yellowstone strains of *Synechococcus*, and *Pseudanabaena*) as shown in Online Resource 4, although the exact branch point is different in different studies (Ponce-Toledo et al. 2017; Shih et al. 2013). However, PsaA and PsaB are sister to α-cyanobacteria (*Prochlorococcus, Synechococcus*), whereas ChlBLN, RbcL [green lineage], RPE, and PRK originate from β-cyanobacteria (*Nostoc*, *Cyanothece*, *Synechocystis*, etc.). In addition, relative stem lengths of RbcL, PsaD, and the set A48 (23 photosynthetic proteins encoded by the chloroplast genome) are larger than that of rRNA. These results suggest that photosynthesis-related proteins are diverse in origins (Fig. 6). Analysis of individual proteins in the set A48 will shed more light on the origin of photosynthetic machinery, although this is not the main focus of the present study.

### Possible explanations

The finding of the present study that the origin of chloroplast enzymes, especially those involved in the synthesis of chloroplast membranes, is quite diverse in both phylogeny and time of acquisition is not consistent with the currently accepted endosymbiotic theory on the origin of chloroplasts, involving a single event of endosymbiosis. Several ideas can resolve this conflict. First, an easy solution is to deny the validity of the phylogenetic analysis, especially, the estimation of acquisition times of enzymes. Second, the two hypotheses can be merged, namely, accepting both single event of endosymbiosis and many gene transfers to chloroplasts in addition. The first attitude is not justified, because the endosymbiotic theory has never been rigorously tested by phylogenetic analysis with massive genomic data, which became available after the acceptance of the endosymbiotic theory in the late 20th century (see next section). In the second possibility, people are willing to admit that many gene transfers occurred *after* the establishment of chloroplasts. The present study revealed, however, that many enzymes related to photosynthesis and membrane lipid synthesis could have been acquired *before* the acquisition of chloroplast ribosome. We can imagine another scenario, in which an initial chloroplast was acquired by endosymbiosis with the enzymes of photosynthesis and lipid biosynthesis. Then, the ribosome was replaced by a new one from another endosymbiont. This could be a good idea. But then, we have to ask what were endosymbioses and what were gene transfers. Bacterial contribution to the plastid proteome has been repeatedly reported (Qiu et al. 2013), and an entirely different scenario was presented by Martin’s group (Ku et al. 2015), in which various bacterial enzymes had been transferred to a special lineage of cyanobacteria, which then became an endosymbiont in the primary endosymbiosis that engendered chloroplasts. A problem in this hypothesis is that such ancestral endosymbiont must have possessed two redundant pathways of glycolipid synthesis (MDG1–DGD1 for chloroplasts, and MgdA–MgdE–DgdA for cyanobacteria). I consider more plausible to suppose that the chloroplast lipid biosynthesis system was established before the acquisition of chloroplast ribosomes, because glycolipids must have provided adaptive advantage even before the formation of chloroplasts.

### Historical considerations on the origin of endosymbiotic theory

It is helpful to review the origin of endosymbiotic theory itself. Historically, the endosymbiotic hypothesis was initially conceived by Mereschkowsky (1905), but had no impact on biology in the first half of the 20th century. The endosymbiotic theory was revived in the 1960s and 1970s by various scientists as a possibility to explain a phylogenetic inconsistency, namely, the similarity of cyanobacteria (called blue-green algae, then) and chloroplasts, and the similarity in cell mechanisms of eukaryotic organisms, including plants, algae, protists, and animals (for reviews of the time, see Stanier 1970, 1974). See my previous publication (Sato 2017) for historical overview on the endosymbiotic theory with a re-evaluation of the work of Lynn Margulis. At that time, gene transfer was known only in bacteria and viruses. No one considered a possibility of gene transfer from a prokaryote to a eukaryote. Various serious doubts were cast on the endosymbiotic theory until the mid 1970s, but an early phylogenetic analysis using rRNA and some chloroplast genes provided qualitative evidence for the cyanobacterial origin of chloroplasts (Gray and Doolittle 1982; Schwartz and Dayhoff 1978). Once the endosymbiotic origin was accepted, various discussions and doubts about it were forgotten, and a visual image of endosymbiosis became a standalone model that does not require further rigorous verification. People no longer paid attention to the concept that membranes are not inherited by themselves. Various visual explanations of the endosymbiotic origin of primary and secondary chloroplasts were presented in top journals in the 1980s and 1990s (see for example, Cavalier-Smith 2000), but no one doubted the reality of the images, forgetting to explain the origin of chloroplast membranes in reasonable ways.

From the early days, we have been showing that cyanobacteria and chloroplasts have different pathways of glycolipid synthesis (Sato and Murata 1982). Later identification of glycolipid synthesis genes in plants and cyanobacteria clearly established fundamental differences in the two systems (for a short review, see Sato and Awai 2016). In this respect, the chloroplast membranes are not heritages of cyanobacteria. Some people might still try to consider that enzymes in membrane lipid synthesis must be replaced after the primary endosymbiosis. The present study clearly showed that the acquisition times of the enzymes were diverse, and some enzymes could have been acquired before the acquisition of chloroplast ribosomes. Certainly, it is no longer possible to keep the simple endosymbiotic explanation. Rather, we have to assume many repeated endosymbiotic events with cyanobacteria, chlamydiae, green bacteria, and others, if we still use endosymbiotic explanation. There are discussions about the role of chlamydiae, working with cyanobacteria to establish chloroplasts (Cenci et al. 2017; Domman et al. 2015), but the problem might not be limited to chlamydiae. Do we need to keep the endosymbiotic explanation? We only need many gene transfers. Some transfers might be triggered by endosymbiosis, but as stated above, the endosymbiont membranes were never inherited by themselves. We cannot keep the visual image of endosymbiosis any more, because chloroplast membranes are quite different from cyanobacterial membranes.

### Philosophical considerations on the endosymbiotic theory

Many readers might question whether I try to deny the endosymbiotic explanation as a whole or many gene transfers that I suppose above could still be explained within the framework of the endosymbiotic theory. It is pertinent to present a logical or philosophical analysis of the theory to answer the question or doubt.

The current general belief of the endosymbiotic notion comes from the following inference:

- First premise: many chloroplast genes (or most parts of the chloroplast genome) originate from cyanobacteria.
- Second premise: an organelle as a whole has a high evolutionary fitness, whereas its components do not.
- Conclusion: chloroplast must be acquired as a whole from a cyanobacterial ancestor.

This is a very strong type of inference, which is hardly falsified by any experimental evidence. The notion “endosymbiotic origin” is a hard-core theory, which is supported by this type of very strong inference, and this is the point that I try to criticize. Can we relax this inference to present a more reasonable argument from the scientific viewpoint? As I argued, acquisition of glycolipids could have been beneficial for the ancient host cell (future algal cell) in phosphate-limited environments. Photosynthetic genes and gene expression machinery could be acquired independently, because they have different functions. We can relax the second premise by replacing it with functionality of smaller units. Some metabolic enzymes could be acquired alone. We might be able to hypothesize a neutral evolution for some components. In other words, we should not be too much constrained by the benefit that a component affords to the organelle. The “shopping bag model” could be an interesting hypothesis in this regard (Howe et al. 2008). Exaptation or pre-adaptation could explain acquisition of some enzymes of an entire pathway such as peptidoglycan synthesis. For example, the fact that the Arabidopsis MurE acts as a regulator in chloroplast development (Lin et al. 2017) could suggest that the initial acquisition of this enzyme could be uncoupled with other components of peptidoglycan synthesis. We might be able to relax the hard-core theory of endosymbiotic origin of chloroplasts by accumulation of various efforts like this. At this moment, I am not sure if the notion “endosymbiosis” is necessary to explain the origin of chloroplasts (or mitochondria). This should be demonstrated experimentally but not logically.

This discussion should be limited to the primary endosymbiosis of Archaeplastida, but not the secondary endosymbiosis. The secondary endosymbiosis is supported by cytological and genetic evidence, and we can trace the pathways of endosymbiosis by identifying intermediates having a nucleomorph. Initial formation of chloroplasts might not be a simple process.

### Alternative hypotheses

As an alternative to the current, simplistic notion of endosymbiotic origin of chloroplasts, I present two hypotheses: hidden cyanobacterial lineage and host-directed chloroplasts formation. The first hypothesis is introduced to explain type 2 phylogeny of chloroplast proteins. This type of phylogenetic trees implicate that chloroplast proteins originate from an ancestor of cyanobacteria. This could be achieved by assuming either that gene transfer occurred from a cyanobacterial ancestor to a eukaryotic host before chloroplast formation, or that another, hidden cyanobacterial lineage diverging before the diversification of extant cyanobacteria provided genes to chloroplasts. If the relative stem length is long for all proteins showing type 2 phylogeny, then we can choose the first alternative, namely, ancient gene transfer from a cyanobacterial ancestor to a eukaryote. But MraY, FtsZ, NdhC, and NdhE had short stems. It is difficult to imagine transfer of the genes encoding these proteins from a cyanobacterial ancestor to chloroplasts. That is why I suppose that another, hidden lineage of cyanobacteria might have been kept in a dormant state (with a low evolutionary rate) and provided genes to chloroplasts at later times.

Type 2 phylogeny was consistently obtained for SQD1, SQD2, FtsZ, and other proteins listed in Fig. 2. However, we cannot completely exclude a possibility that type 2 trees could be artefact of phylogenetic reconstruction. Inconsistent phylogenetic trees (type 1 or type 2) obtained with RpoB, RpoC, and some Ndh proteins (see “Results”) could cast doubt about the reality of type 2 phylogeny. We cannot answer this in the current state of knowledge and technique. It is best to keep this question to eventually answer it in the future.

Host-directed chloroplast formation is an attractive alternative to the simplistic view on the endosymbiotic origin of chloroplasts. The results of the present study showed that the genes encoding the enzymes involved in glycolipid biosynthesis and photosynthesis-related proteins could have been present in a eukaryote (future host) before the formation of chloroplasts. This is similar to the premitochondrion hypothesis (Gray 2014) and the “pre-mitochondrial symbioses” hypothesis (Gabaldón 2018) for the origin of mitochondrial proteins, which tried to explain early acquisition of many mitochondrial proteins before the mitochondrial formation. As already explained, the glycolipids have advantage in adaptation to low phosphate environments. We can imagine a modified autogenous scenario, in which a eukaryotic future host acquired ability to form membranes consisting of glycolipids, then incorporated photosynthetic machinery from cyanobacteria into the membranes, and finally acquired ribosomes again from cyanobacteria. It is tempting to assume parallel scenarios for the origin of mitochondria and chloroplasts. However, falsification of endosymbiotic origin of chloroplasts could have a stronger impact than that of mitochondria, because chloroplasts and cyanobacteria share a clearly identified function, photosynthesis. The origin of eukaryotic cells and organelles could be far more complicated than imagined before.

An additional hypothesis is the fatty acid hypothesis. Early acquisition of membrane forming ability was also important from the point of view of fatty acid synthesis. Archaea do not use acyl lipids and have no ability of fatty acid synthesis (for a review, see Caforio and Driessen 2016). Early eukaryotes were obliged to make their membranes by obtaining fatty acids from bacterial preys. Acquisition of ability of fatty acid synthesis is preferable for eukaryotes, but this required a large reducing power. Photosynthetic production of a large reducing power was beneficial to fatty acid synthesis as well as carbon fixation. Early acquisition of fatty acid synthesis ability guaranteed ability to produce numerous photosynthetic membranes in a eukaryotic ancestor. Photosynthesis, fatty acid synthesis, and glycolipid synthesis were a tightly related trio that promoted evolution of photosynthetic eukaryotes, and this does not require endosymbiosis.

### Remaining problems and concluding remarks

All our discussions are related to the primary endosymbiosis, namely, the formation of chloroplasts in the initial Archaeplastida. The monophyly of Archaeplastida is supported by phylogenetic analyses cited in Introduction, and the conservation of translocon components (Tic20, Tic21, Tic22, and Toc75). Another line of evidence for monophyly might be the overall similarity in phylogenetic types of various chloroplast proteins (with some exceptions) in the three lineages of Archaeplastida.

We have to analyze the secondary endosymbiosis in future studies. Data on diatoms are included in the phylogenetic trees in the present study, but the phylogenetic relationship of chloroplast enzymes of diatoms with other algae and plants was variable, either within the plant/algal clade, or outside it. Both the red algal enzymes and the enzymes of the secondary host could contribute to the proteome of the diatom chloroplasts. Nevertheless, the shape of phylogenetic tree is sometimes strange for the diatom clade, and we will have to analyze carefully the phylogenetic relationship of diatom chloroplasts with other chloroplasts.

*Paulinella chromatophora* and its related protists are interesting organisms that originate from a recent (probably about 100 million years ago) endosymbiosis of an α-cyanobacterium (Lhee et al. 2017; Nowack et al. 2008). I have included one or two *Paulinella* species in all the phylogenetic trees in the present study. All enzymes in the biosynthesis of lipids and fatty acids in the chromatophore are entirely encoded by the chromatophore genome, and originate from cyanobacteria. The same is true for all Calvin-Benson Cycle enzymes, photosynthetic proteins, and division proteins that were analyzed in the present study. These provided good control data that my phylogenetic analyses were properly performed. The only chromatophore enzyme currently known to originate from proteobacteria is MurF involved in peptidoglycan synthesis (Nowack et al. 2016; Sato and Takano 2017). In this respect, the chromatophores of *Paulinella chromatophora* and related photosynthetic *Paulinella* are semiautonomous endosymbionts/organelles, and cannot survive outside the host cell. Supporting evidence for semi-autonomy was obtained by lipid analysis and lipid biosynthetic studies recently performed in our laboratory. The situation is fundamentally different from that of chloroplasts of plants and algae. *Paulinella* could be a good model of endosymbiosis, but we have to distinguish between chromatophores and chloroplasts.

On the other hand, we can recognize an interesting similarity of chromatophores and chloroplasts. Singer et al. (2017) demonstrated that about 450 nuclear-encoded proteins are targeted to chromatophores, and that most of them probably originated from the ancestral eukaryotic host. They suggested re-targeting of these proteins. In other words, the host cell had prepared these re-targetable protein genes before the chromatophore endosymbiosis. This is in line with my hypothesis of host-directed chloroplast formation. Therefore, despite apparent differences in integration of the endosymbiont/organelle within the host cell, we can assume a similar scenario of formation of the endosymbiont/organelle, in which the host had already genes for the proteins to be used in the endosymbiont/organelle.

Diverse origins of chloroplast DNA replication machineries (Moriyama and Sato 2014) and nucleoid components (Sato 2001) conform to the complex history of chloroplast formation described above. DNA polymerase of viral origin was first used in mitochondria and then also used in chloroplasts. Various components related to DNA replication were replaced during the evolution of chloroplasts mainly in the green lineage, but some components were also replaced in the red lineage. All these processes are supposed to occur after the establishment of chloroplasts.

The origins of chloroplast enzymes are diverse, in both donor organisms and acquisition times. Especially, most enzymes involved in chloroplast membrane lipids did not originate from cyanobacteria, but rather from various bacteria and eukaryotes. We cannot adhere to the visual image of the endosymbiotic origin of chloroplasts, explicitly showing membrane heredity. In addition, some of the photosynthesis-related proteins and enzymes that originated from cyanobacteria could be acquired at diverse times, either before or after the acquisition of ribosomal components. The origin of chloroplasts might not be as simple as we hypothesize with the visual image of cyanobacterial endosymbiosis, which occurred once at a fixed time during the evolution of eukaryotes.

## Electronic supplementary material

Below is the link to the electronic supplementary material.

Supplementary material 1 (XLSX 26 kb)

Supplementary material 2 (PDF 1310 kb)

Supplementary material 3 (PDF 3645 kb)

Supplementary material 4 (PDF 3118 kb)

Supplementary material 5 (PDF 186 kb)

Supplementary material 6 (PDF 1105 kb)

Supplementary material 7 (PDF 324 kb)

Supplementary material 8 (XLSX 19 kb)
