# Supplementary material for: Complex origins of chloroplast membranes with photosynthetic machineries: multiple transfers of genes from divergent organisms at different times or a single endosymbiotic event?
Source: J Plant Res. 2019 Dec 6;133(1):15–33. doi: 10.1007/s10265-019-01157-z (PMC6946739; doi:10.1007/s10265-019-01157-z)
Supplement: Supplementary file 2 — Supplementary material 2 (PDF 1310 kb) [file 10265_2019_1157_MOESM2_ESM.pdf]

Journal of Plant Research

Online Resources

Complex origins of chloroplast membranes with photosynthetic machineries: Multiple transfers of genes from divergent organisms at different times or a single endosymbiotic event?

Naoki Sato

University of Tokyo

naokisat@bio.c.u-tokyo.ac.jp

Phylogenetic trees are grouped in 6 PDF files (Online Resources 2 – 7).

Online Resource 2. Phylogenetic trees of enzymes involved in chloroplast lipid biosynthesis.

This PDF file includes phylogenetic trees of the following enzymes: MDG1, monogalactosyl diacylglycerol synthase in plants/algae; DGD1, digalactosyl diacylglycerol synthase in plants/algae; MgdA, monoglucosyl diacylglycerol synthase in cyanobacteria; monoglucosyl diacylglycerol epimerase in cyanobacteria; DgdA, digalactosyl diacylglycerol synthase in cyanobacteria; CDS, CDP diacylglycerol synthase; PGPS, phosphatidylglycerol phosphate synthase (including eukaryotic CLS, cardiolipin synthase); PGPP1, phosphatidylglycerol phosphate phosphatase; SqdB/SQD1, UDP sulfoquinovose synthase; SqdX/SQD2, sulfoquinovosyl diacylglycerol synthase. Locate the enzymes in the biosynthetic pathway in Fig. 1 panels B and C.

Three types of phylogenetic methods were tested depending on enzymes: BI, Bayesian Inference method by MrBayes version 3.2.6 with LG model; PB, Bayesian Inference method by PhyloBayes version 4.1 with CAT model; ML, Maximum Likelihood method by PhyML version 4 with LG model. A combination of numbers on each major branch indicates branch support values. For simplicity, support values are not shown for minor branches. "1.00" was shown as "1" for short. Taxa are consistently color-coded according to the color scheme in the FigTree software throughout this paper (the original RGB intensities are shown in each parenthesis): Green (or Spring) (0, 255, 0), land plants; Sea Foam (0, 255, 128), green algae and *Paulinella*; Lime(128, 255, 0), *Cyanophora*; Salmon (255, 102, 102), red algae; Cantaloupe (255, 204, 102), diatoms; Red (or Maraschino) (255, 0, 0), α-proteobacteria; Tangerine (255, 128, 0), β-proteobacteria; Grape (128, 0, 255), γ-proteobacteria; Cyan (or Turquoise) (0, 255, 255), cyanobacteria; Clover (0, 128, 0), green bacteria; Midnight (0, 0, 128), Firmicutes; Aluminum (153, 153, 153), Archaea; Mocha (128, 64, 0), actinobacteria; Steel (102, 102, 102), protists; Black (0, 0, 0), other bacteria and animals. This color code is used in all subsequent phylogenetic trees in Online Resources.

MGD1 (BI/ML)

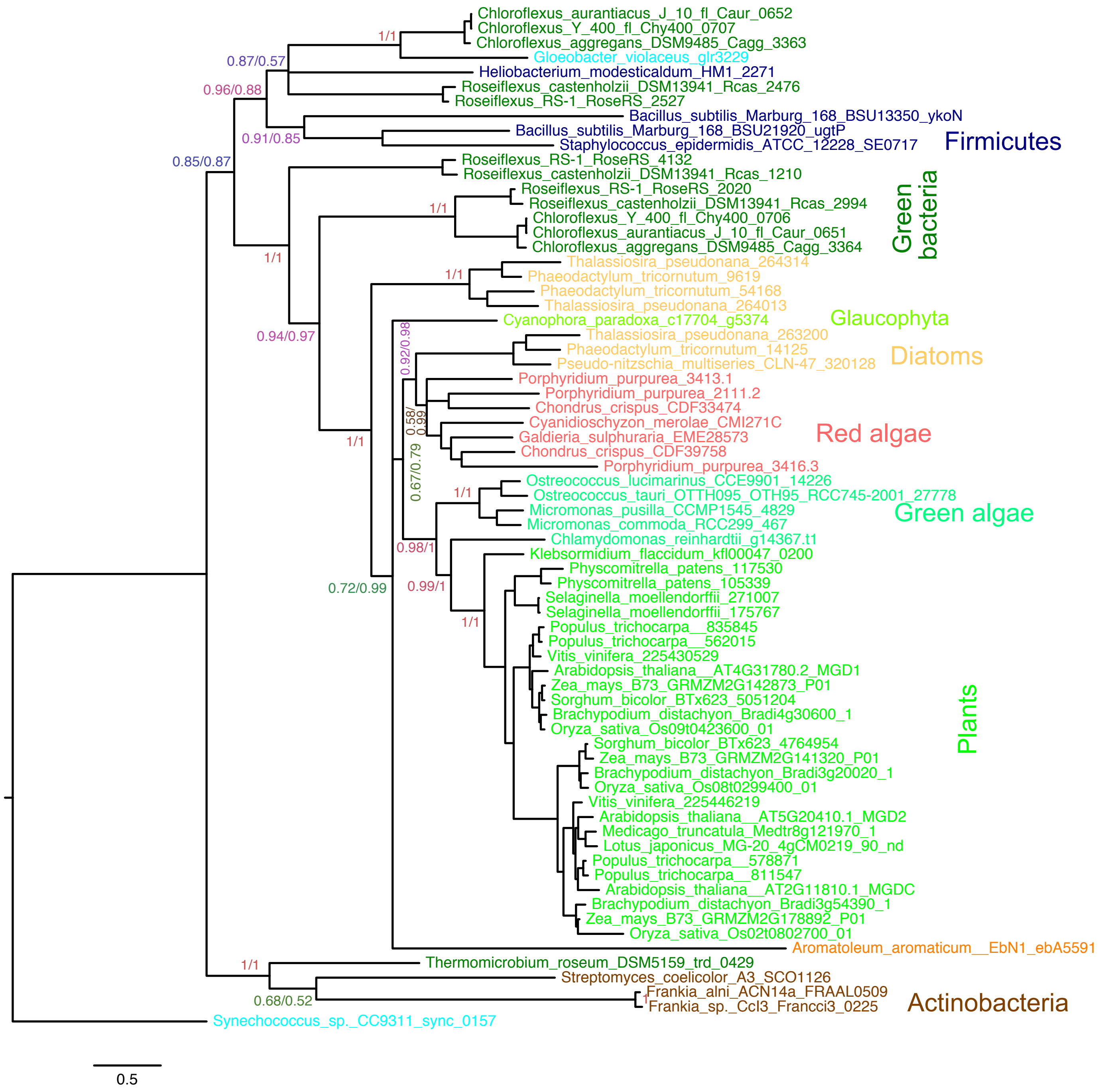
Chloroflexus_aurantiacus_J_10_fl_Caur_0652
Chloroflexus_Y_400_fl_Chy400_0707
Chloroflexus_aggregans_DSM9485_Cagg_3363
Gloeobacter_violaceus_glr3229
Heliobacterium_modesticaldum_HM1_2271
Roseiflexus_castenholzii_DSM13941_Rcas_2476
Roseiflexus_RS-1_RoseRS_2527
Bacillus_subtilis_Marburg_168_BSU13350_ykoN
Bacillus_subtilis_Marburg_168_BSU21920_ugtP
Staphylococcus_epidermidis_ATCC_12228_SE0717
Firmicutes
Roseiflexus_RS-1_RoseRS_4132
Roseiflexus_castenholzii_DSM13941_Rcas_1210
Roseiflexus_RS-1_RoseRS_2020
Roseiflexus_castenholzii_DSM13941_Rcas_2994
Chloroflexus_Y_400_fl_Chy400_0706
Chloroflexus_aurantiacus_J_10_fl_Caur_0651
Chloroflexus_aggregans_DSM9485_Cagg_3364
Green bacteria
Thalassiosira_pseudonana_264314
Phaeodactylum_tricornutum_9619
Phaeodactylum_tricornutum_54168
Thalassiosira_pseudonana_264013
Cyanophora_paradoxa_c17704_g5374
Glaucophyta
Thalassiosira_pseudonana_263200
Phaeodactylum_tricornutum_14125
Pseudo-nitzschia_multiseries_CLN-47_320128
Diatoms
Porphyridium_purpurea_3413.1
Porphyridium_purpurea_2111.2
Chondrus_crispus_CDF33474
Cyanidioschyzon_merolae_CMI271C
Galdieria_sulphuraria_EME28573
Chondrus_crispus_CDF39758
Porphyridium_purpurea_3416.3
Red algae
Ostreococcus_lucimarinus_CCE9901_14226
Ostreococcus_tauri_OTTH095_OTH95_RCC745-2001_27778
Micromonas_pusilla_CCMP1545_4829
Micromonas_commoda_RCC299_467
Green algae
Chlamydomonas_reinhardtii_g14367.t1
Klebsormidium_flaccidum_kfl00047_0200
Physcomitrella_patens_117530
Physcomitrella_patens_105339
Selaginella_moellendorffii_271007
Selaginella_moellendorffii_175767
Populus_trichocarpa__835845
Populus_trichocarpa__562015
Vitis_vinifera_225430529
Arabidopsis_thaliana__AT4G31780.2_MGD1
Zea_mays_B73_GRMZM2G142873_P01
Sorghum_bicolor_BTx623_5051204
Brachypodium_distachyon_Bradi4g30600_1
Oryza_sativa_Os09t0423600_01
Sorghum_bicolor_BTx623_4764954
Zea_mays_B73_GRMZM2G141320_P01
Brachypodium_distachyon_Bradi3g20020_1
Oryza_sativa_Os08t0299400_01
Vitis_vinifera_225446219
Arabidopsis_thaliana__AT5G20410.1_MGD2
Medicago_truncatula_Medtr8g121970_1
Lotus_japonicus_MG-20_4gCM0219_90_nd
Populus_trichocarpa__578871
Populus_trichocarpa__811547
Arabidopsis_thaliana__AT2G11810.1_MGDC
Brachypodium_distachyon_Bradi3g54390_1
Zea_mays_B73_GRMZM2G178892_P01
Oryza_sativa_Os02t0802700_01
Plants
Aromatoleum_aromaticum__EbN1_ebA5591
Thermomicrobium_roseum_DSM5159_trd_0429
Streptomyces_coelicolor_A3_SCO1126
Frankia_alni_ACN14a_FRAAL0509
Frankia_sp._CcI3_Francci3_0225
Actinobacteria
Synechococcus_sp._CC9311_sync_0157
1/1
0.87/0.57
0.96/0.88
0.91/0.85
0.85/0.87
1/1
1/1
0.94/0.97
1/1
0.92/0.98
0.58/0.99
0.67/0.79
1/1
1/1
0.98/1
0.72/0.99
0.99/1
1/1
1/1
0.68/0.52
1
0.5

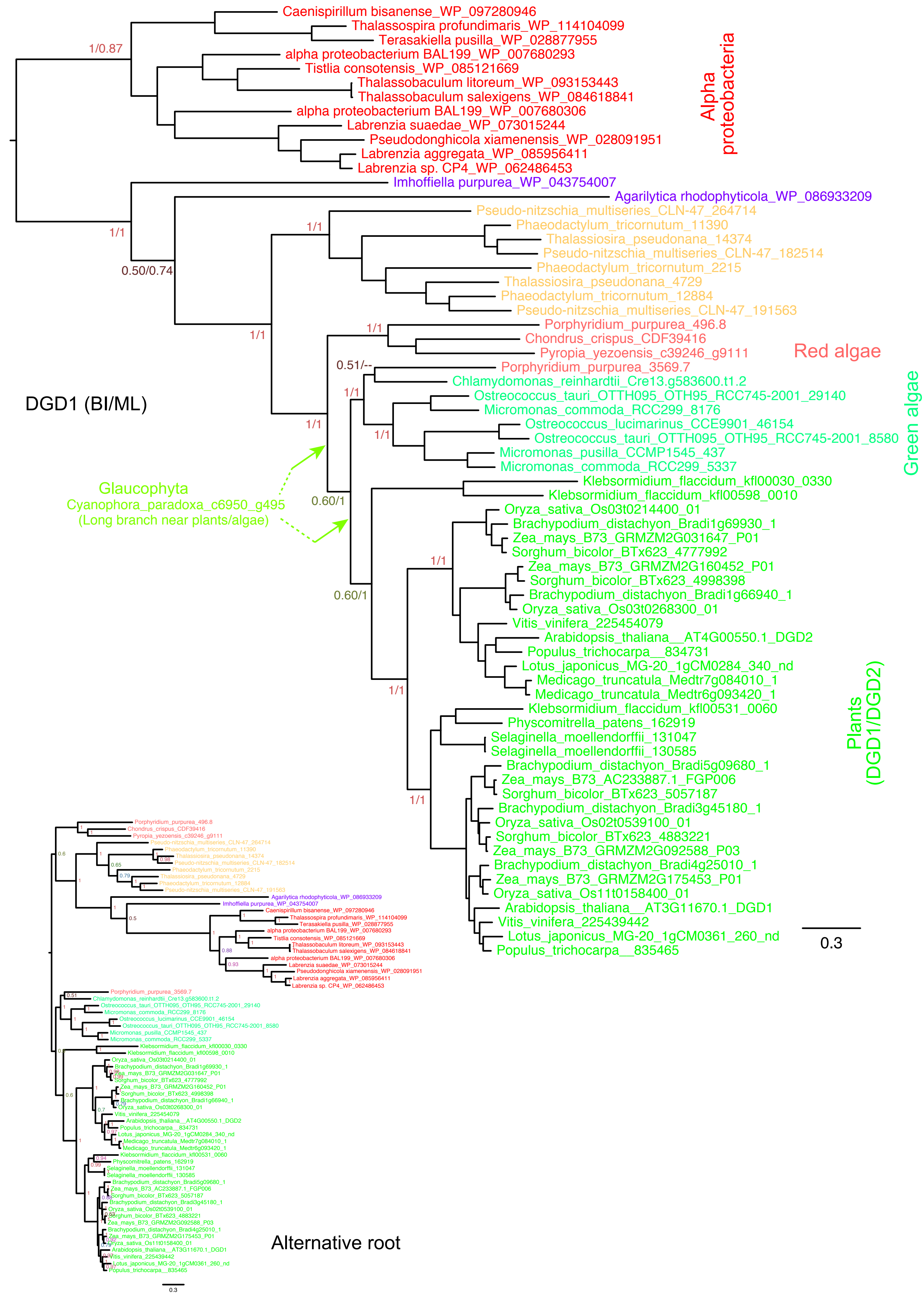

Caenispirillum bisanense_WP_097280946
Thalassospira profundimaris_WP_114104099
Terasakiella pusilla_WP_028877955
alpha proteobacterium BAL199_WP_007680293
Tistlia consotensis_WP_085121669
Thalassobaculum litoreum_WP_093153443
Thalassobaculum salexigens_WP_084618841
alpha proteobacterium BAL199_WP_007680306
Labrenzia suaedae_WP_073015244
Pseudodonghicola xiamenensis_WP_028091951
Labrenzia aggregata_WP_085956411
Labrenzia sp. CP4_WP_062486453
Alpha proteobacteria
1/0.87
Imhoffiella purpurea_WP_043754007
Agarilytica rhodophyticola_WP_086933209
1/1
0.50/0.74
Pseudo-nitzschia_multiseries_CLN-47_264714
Phaeodactylum_tricornutum_11390
Thalassiosira_pseudonana_14374
Pseudo-nitzschia_multiseries_CLN-47_182514
Phaeodactylum_tricornutum_2215
Thalassiosira_pseudonana_4729
Phaeodactylum_tricornutum_12884
Pseudo-nitzschia_multiseries_CLN-47_191563
1/1
1/1
Porphyridium_purpurea_496.8
Chondrus_crispus_CDF39416
Pyropia_yezoensis_c39246_g9111
Red algae
1/1
0.51/--
Porphyridium_purpurea_3569.7
Chlamydomonas_reinhardtii_Cre13.g583600.t1.2
Ostreococcus_tauri_OTTH095_OTH95_RCC745-2001_29140
Micromonas_commoda_RCC299_8176
Ostreococcus_lucimarinus_CCE9901_46154
Ostreococcus_tauri_OTTH095_OTH95_RCC745-2001_8580
Micromonas_pusilla_CCMP1545_437
Micromonas_commoda_RCC299_5337
Green algae
1/1
1/1
1/1
DGD1 (BI/ML)
Glaucophyta
Cyanophora_paradoxa_c6950_g495
(Long branch near plants/algae)
0.60/1
0.60/1
Klebsormidium_flaccidum_kfl00030_0330
Klebsormidium_flaccidum_kfl00598_0010
Oryza_sativa_Os03t0214400_01
Brachypodium_distachyon_Bradi1g69930_1
Zea_mays_B73_GRMZM2G031647_P01
Sorghum_bicolor_BTx623_4777992
Zea_mays_B73_GRMZM2G160452_P01
Sorghum_bicolor_BTx623_4998398
Brachypodium_distachyon_Bradi1g66940_1
Oryza_sativa_Os03t0268300_01
Vitis_vinifera_225454079
Arabidopsis_thaliana__AT4G00550.1_DGD2
Populus_trichocarpa__834731
Lotus_japonicus_MG-20_1gCM0284_340_nd
Medicago_truncatula_Medtr7g084010_1
Medicago_truncatula_Medtr6g093420_1
1/1
1/1
Klebsormidium_flaccidum_kfl00531_0060
Physcomitrella_patens_162919
Selaginella_moellendorffii_131047
Selaginella_moellendorffii_130585
Brachypodium_distachyon_Bradi5g09680_1
Zea_mays_B73_AC233887.1_FGP006
Sorghum_bicolor_BTx623_5057187
Brachypodium_distachyon_Bradi3g45180_1
Oryza_sativa_Os02t0539100_01
Sorghum_bicolor_BTx623_4883221
Zea_mays_B73_GRMZM2G092588_P03
Brachypodium_distachyon_Bradi4g25010_1
Zea_mays_B73_GRMZM2G175453_P01
Oryza_sativa_Os11t0158400_01
Arabidopsis_thaliana__AT3G11670.1_DGD1
Vitis_vinifera_225439442
Lotus_japonicus_MG-20_1gCM0361_260_nd
Populus_trichocarpa__835465
1/1
Plants (DGD1/DGD2)
0.3
Alternative root
0.3

MgdA (BI/ML)

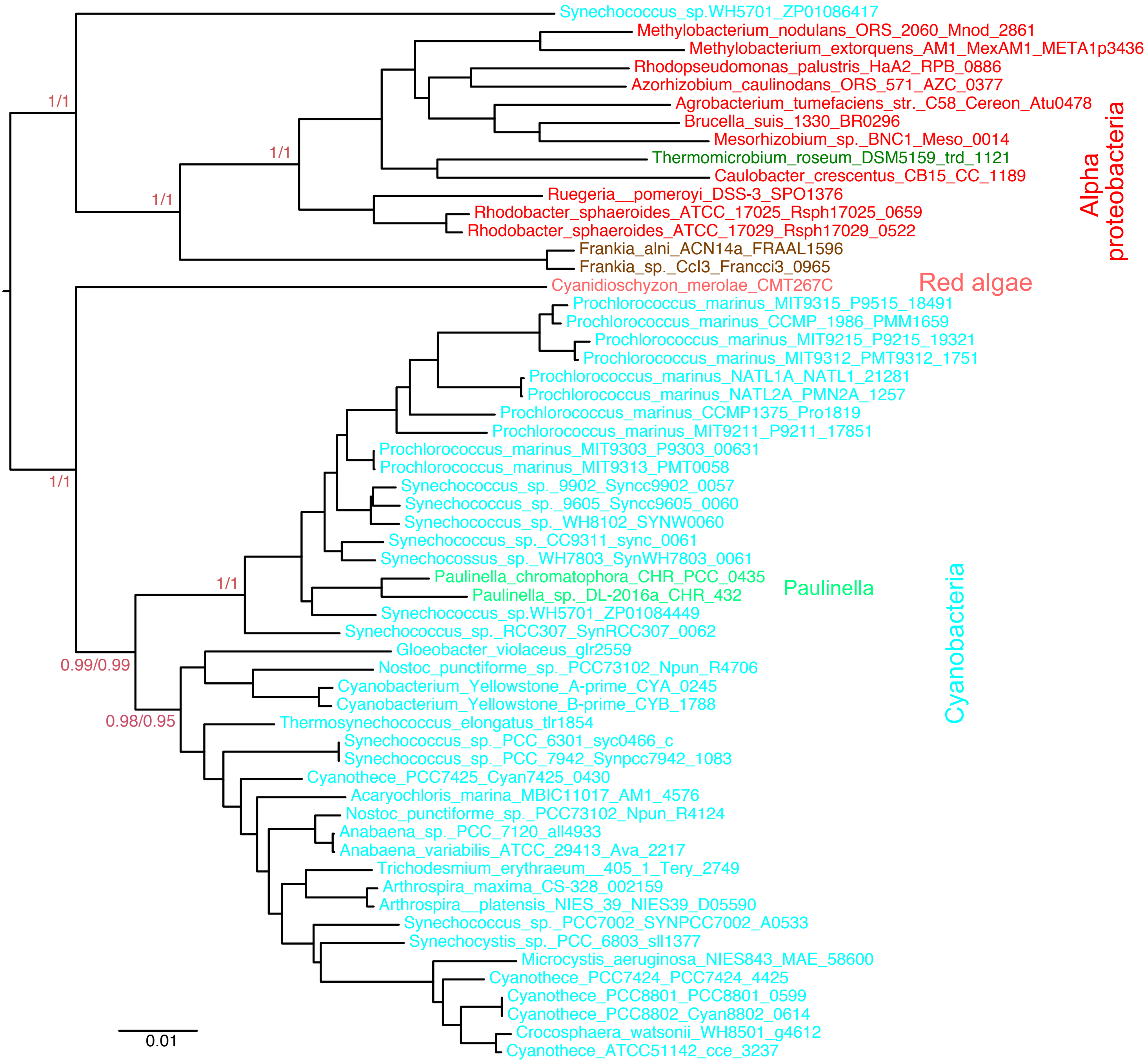
Synechococcus_sp.WH5701_ZP01086417
Methylobacterium_nodulans_ORS_2060_Mnod_2861
Methylobacterium_extorquens_AM1_MexAM1_META1p3436
Rhodopseudomonas_palustris_HaA2_RPB_0886
Azorhizobium_caulinodans_ORS_571_AZC_0377
Agrobacterium_tumefaciens_str._C58_Cereon_Atu0478
Brucella_suis_1330_BR0296
Mesorhizobium_sp._BNC1_Meso_0014
Thermomicrobium_roseum_DSM5159_trd_1121
Caulobacter_crescentus_CB15_CC_1189
Ruegeria__pomeroyi_DSS-3_SPO1376
Rhodobacter_sphaeroides_ATCC_17025_Rsph17025_0659
Rhodobacter_sphaeroides_ATCC_17029_Rsph17029_0522
Frankia_alni_ACN14a_FRAAL1596
Frankia_sp._CcI3_Francci3_0965
Cyanidioschyzon_merolae_CMT267C
Prochlorococcus_marinus_MIT9315_P9515_18491
Prochlorococcus_marinus_CCMP_1986_PMM1659
Prochlorococcus_marinus_MIT9215_P9215_19321
Prochlorococcus_marinus_MIT9312_PMT9312_1751
Prochlorococcus_marinus_NATL1A_NATL1_21281
Prochlorococcus_marinus_NATL2A_PMN2A_1257
Prochlorococcus_marinus_CCMP1375_Pro1819
Prochlorococcus_marinus_MIT9211_P9211_17851
Prochlorococcus_marinus_MIT9303_P9303_00631
Prochlorococcus_marinus_MIT9313_PMT0058
Synechococcus_sp._9902_Syncc9902_0057
Synechococcus_sp._9605_Syncc9605_0060
Synechococcus_sp._WH8102_SYNW0060
Synechococcus_sp._CC9311_sync_0061
Synechocossus_sp._WH7803_SynWH7803_0061
Paulinella_chromatophora_CHR_PCC_0435
Paulinella_sp._DL-2016a_CHR_432
Synechococcus_sp.WH5701_ZP01084449
Synechococcus_sp._RCC307_SynRCC307_0062
Gloeobacter_violaceus_glr2559
Nostoc_punctiforme_sp._PCC73102_Npun_R4706
Cyanobacterium_Yellowstone_A-prime_CYA_0245
Cyanobacterium_Yellowstone_B-prime_CYB_1788
Thermosynechococcus_elongatus_tlr1854
Synechococcus_sp._PCC_6301_syc0466_c
Synechococcus_sp._PCC_7942_Synpcc7942_1083
Cyanothece_PCC7425_Cyan7425_0430
Acaryochloris_marina_MBIC11017_AM1_4576
Nostoc_punctiforme_sp._PCC73102_Npun_R4124
Anabaena_sp._PCC_7120_all4933
Anabaena_variabilis_ATCC_29413_Ava_2217
Trichodesmium_erythraeum__405_1_Tery_2749
Arthrospira_maxima_CS-328_002159
Arthrospira__platensis_NIES_39_NIES39_D05590
Synechococcus_sp._PCC7002_SYNPCC7002_A0533
Synechocystis_sp._PCC_6803_sll1377
Microcystis_aeruginosa_NIES843_MAE_58600
Cyanothece_PCC7424_PCC7424_4425
Cyanothece_PCC8801_PCC8801_0599
Cyanothece_PCC8802_Cyan8802_0614
Crocosphaera_watsonii_WH8501_g4612
Cyanothece_ATCC51142_cce_3237
1/1
1/1
1/1
1/1
1/1
0.99/0.99
0.98/0.95
0.01
Alpha proteobacteria
Red algae
Paulinella
Cyanobacteria

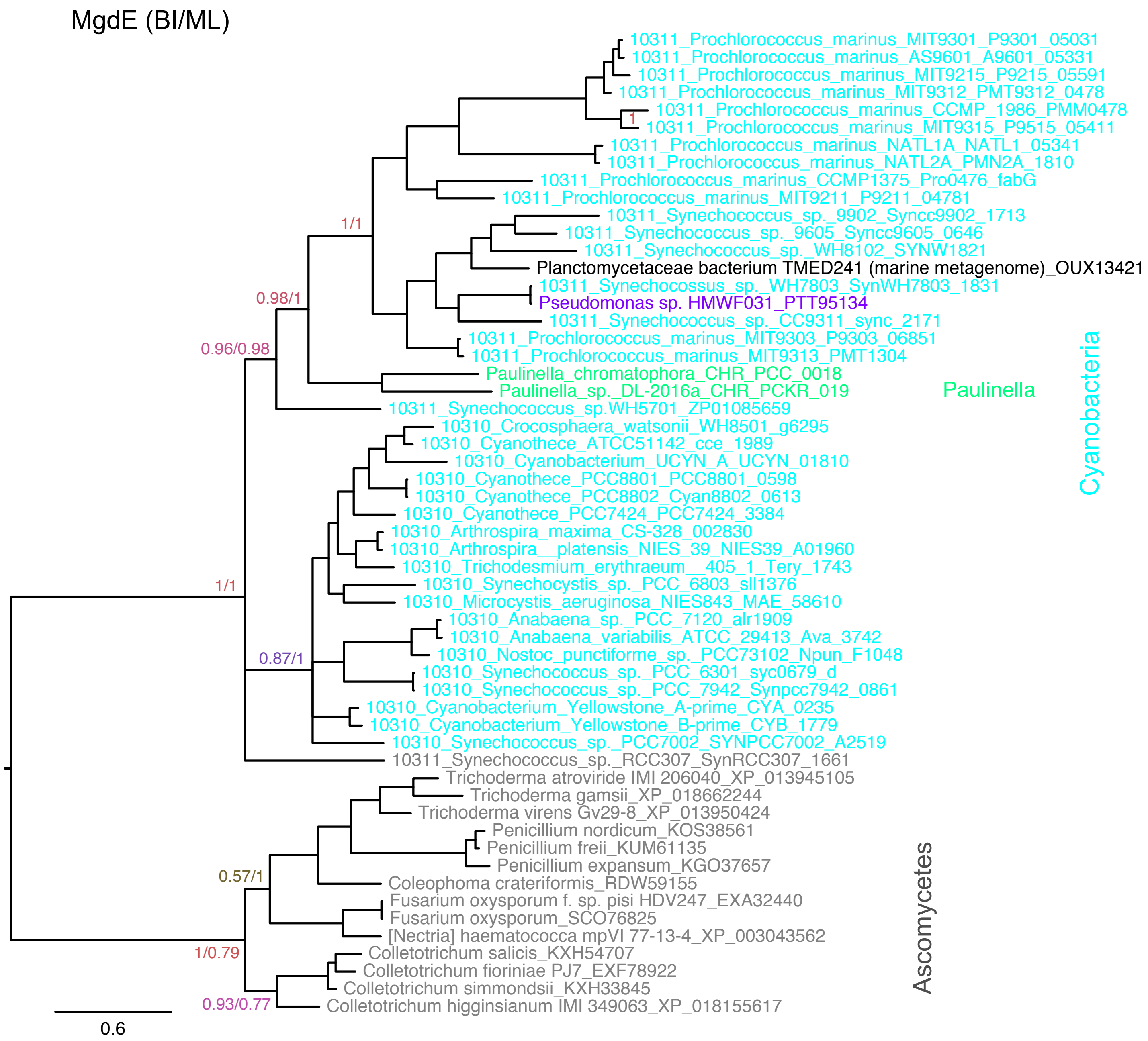

MgdE (BI/ML)
10311_Prochlorococcus_marinus_MIT9301_P9301_05031
10311_Prochlorococcus_marinus_AS9601_A9601_05331
10311_Prochlorococcus_marinus_MIT9215_P9215_05591
10311_Prochlorococcus_marinus_MIT9312_PMT9312_0478
10311_Prochlorococcus_marinus_CCMP_1986_PMM0478
1
10311_Prochlorococcus_marinus_MIT9315_P9515_05411
10311_Prochlorococcus_marinus_NATL1A_NATL1_05341
10311_Prochlorococcus_marinus_NATL2A_PMN2A_1810
10311_Prochlorococcus_marinus_CCMP1375_Pro0476_fabG
10311_Prochlorococcus_marinus_MIT9211_P9211_04781
1/1
10311_Synechococcus_sp._9902_Syncc9902_1713
10311_Synechococcus_sp._9605_Syncc9605_0646
10311_Synechococcus_sp._WH8102_SYNW1821
Planctomycetaceae bacterium TMED241 (marine metagenome)_OUX13421
10311_Synechocossus_sp._WH7803_SynWH7803_1831
Pseudomonas sp. HMWF031_PTT95134
0.98/1
10311_Synechococcus_sp._CC9311_sync_2171
0.96/0.98
10311_Prochlorococcus_marinus_MIT9303_P9303_06851
10311_Prochlorococcus_marinus_MIT9313_PMT1304
Paulinella_chromatophora_CHR_PCC_0018
Paulinella_sp._DL-2016a_CHR_PCKR_019
Paulinella
10311_Synechococcus_sp.WH5701_ZP01085659
10310_Crocosphaera_watsonii_WH8501_g6295
10310_Cyanothece_ATCC51142_cce_1989
10310_Cyanobacterium_UCYN_A_UCYN_01810
10310_Cyanothece_PCC8801_PCC8801_0598
10310_Cyanothece_PCC8802_Cyan8802_0613
10310_Cyanothece_PCC7424_PCC7424_3384
10310_Arthrospira_maxima_CS-328_002830
10310_Arthrospira__platensis_NIES_39_NIES39_A01960
10310_Trichodesmium_erythraeum__405_1_Tery_1743
1/1
10310_Synechocystis_sp._PCC_6803_sll1376
10310_Microcystis_aeruginosa_NIES843_MAE_58610
10310_Anabaena_sp._PCC_7120_alr1909
10310_Anabaena_variabilis_ATCC_29413_Ava_3742
0.87/1
10310_Nostoc_punctiforme_sp._PCC73102_Npun_F1048
10310_Synechococcus_sp._PCC_6301_syc0679_d
10310_Synechococcus_sp._PCC_7942_Synpcc7942_0861
10310_Cyanobacterium_Yellowstone_A-prime_CYA_0235
10310_Cyanobacterium_Yellowstone_B-prime_CYB_1779
10310_Synechococcus_sp._PCC7002_SYNPCC7002_A2519
10311_Synechococcus_sp._RCC307_SynRCC307_1661
Cyanobacteria
Trichoderma atroviride IMI 206040_XP_013945105
Trichoderma gamsii_XP_018662244
Trichoderma virens Gv29-8_XP_013950424
Penicillium nordicum_KOS38561
Penicillium freii_KUM61135
Penicillium expansum_KGO37657
0.57/1
Coleophoma crateriformis_RDW59155
Fusarium oxysporum f. sp. pisi HDV247_EXA32440
Fusarium oxysporum_SCO76825
[Nectria] haematococca mpVI 77-13-4_XP_003043562
1/0.79
Colletotrichum salicis_KXH54707
Colletotrichum fioriniae PJ7_EXF78922
Colletotrichum simmondsii_KXH33845
0.93/0.77
Colletotrichum higginsianum IMI 349063_XP_018155617
Ascomycetes
0.6

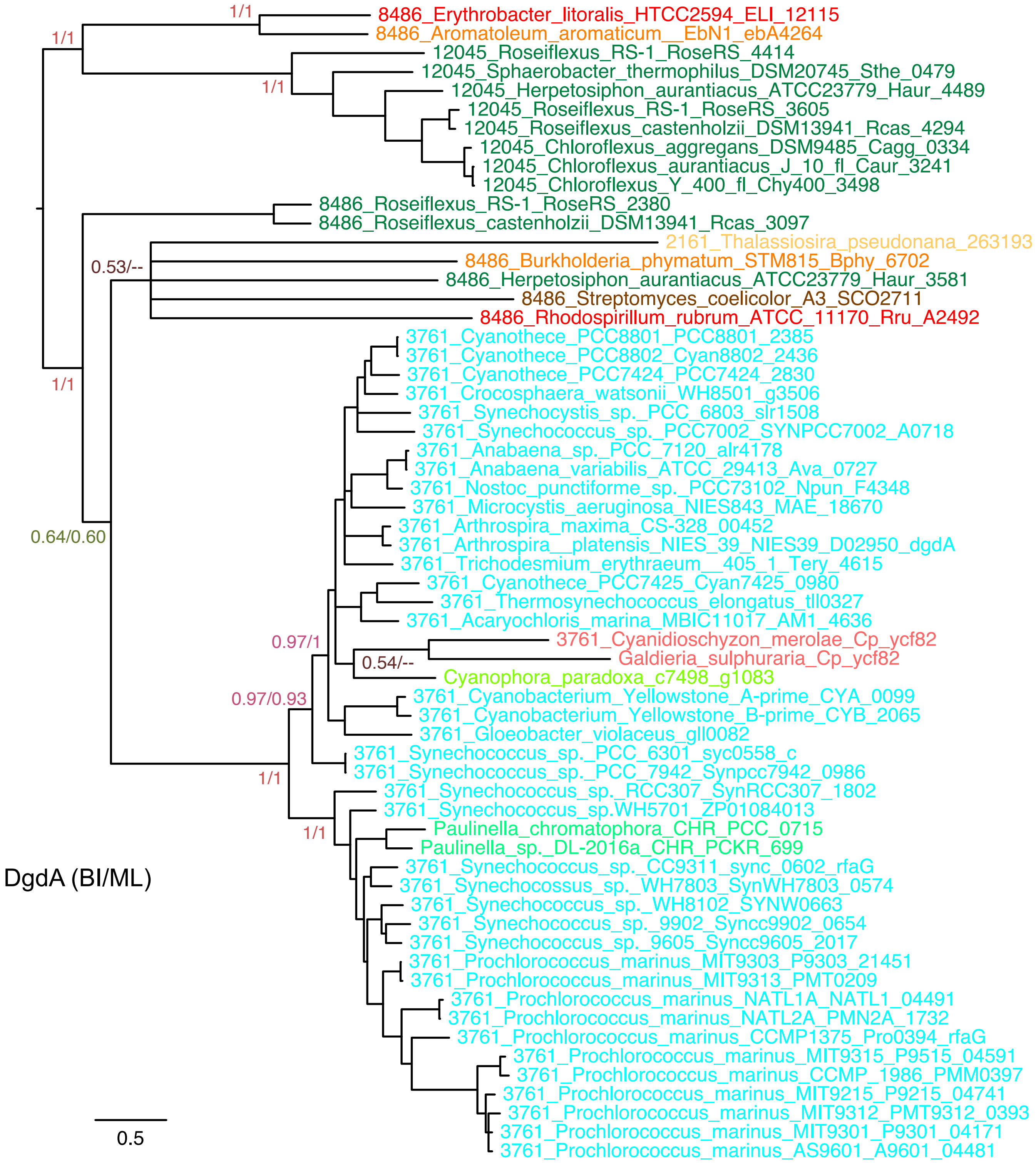
8486_Erythrobacter_litoralis_HTCC2594_ELI_12115
8486_Aromatoleum_aromaticum__EbN1_ebA4264
12045_Roseiflexus_RS-1_RoseRS_4414
12045_Sphaerobacter_thermophilus_DSM20745_Sthe_0479
12045_Herpetosiphon_aurantiacus_ATCC23779_Haur_4489
12045_Roseiflexus_RS-1_RoseRS_3605
12045_Roseiflexus_castenholzii_DSM13941_Rcas_4294
12045_Chloroflexus_aggregans_DSM9485_Cagg_0334
12045_Chloroflexus_aurantiacus_J_10_fl_Caur_3241
12045_Chloroflexus_Y_400_fl_Chy400_3498
8486_Roseiflexus_RS-1_RoseRS_2380
8486_Roseiflexus_castenholzii_DSM13941_Rcas_3097
2161_Thalassiosira_pseudonana_263193
8486_Burkholderia_phymatum_STM815_Bphy_6702
8486_Herpetosiphon_aurantiacus_ATCC23779_Haur_3581
8486_Streptomyces_coelicolor_A3_SCO2711
8486_Rhodospirillum_rubrum_ATCC_11170_Rru_A2492
3761_Cyanothece_PCC8801_PCC8801_2385
3761_Cyanothece_PCC8802_Cyan8802_2436
3761_Cyanothece_PCC7424_PCC7424_2830
3761_Crocosphaera_watsonii_WH8501_g3506
3761_Synechocystis_sp._PCC_6803_slr1508
3761_Synechococcus_sp._PCC7002_SYNPCC7002_A0718
3761_Anabaena_sp._PCC_7120_alr4178
3761_Anabaena_variabilis_ATCC_29413_Ava_0727
3761_Nostoc_punctiforme_sp._PCC73102_Npun_F4348
3761_Microcystis_aeruginosa_NIES843_MAE_18670
3761_Arthrospira_maxima_CS-328_00452
3761_Arthrospira__platensis_NIES_39_NIES39_D02950_dgdA
3761_Trichodesmium_erythraeum__405_1_Tery_4615
3761_Cyanothece_PCC7425_Cyan7425_0980
3761_Thermosynechococcus_elongatus_tll0327
3761_Acaryochloris_marina_MBIC11017_AM1_4636
3761_Cyanidioschyzon_merolae_Cp_ycf82
Galdieria_sulphuraria_Cp_ycf82
Cyanophora_paradoxa_c7498_g1083
3761_Cyanobacterium_Yellowstone_A-prime_CYA_0099
3761_Cyanobacterium_Yellowstone_B-prime_CYB_2065
3761_Gloeobacter_violaceus_gll0082
3761_Synechococcus_sp._PCC_6301_syc0558_c
3761_Synechococcus_sp._PCC_7942_Synpcc7942_0986
3761_Synechococcus_sp._RCC307_SynRCC307_1802
3761_Synechococcus_sp.WH5701_ZP01084013
Paulinella_chromatophora_CHR_PCC_0715
Paulinella_sp._DL-2016a_CHR_PCKR_699
3761_Synechococcus_sp._CC9311_sync_0602_rfaG
3761_Synechocossus_sp._WH7803_SynWH7803_0574
3761_Synechococcus_sp._WH8102_SYNW0663
3761_Synechococcus_sp._9902_Syncc9902_0654
3761_Synechococcus_sp._9605_Syncc9605_2017
3761_Prochlorococcus_marinus_MIT9303_P9303_21451
3761_Prochlorococcus_marinus_MIT9313_PMT0209
3761_Prochlorococcus_marinus_NATL1A_NATL1_04491
3761_Prochlorococcus_marinus_NATL2A_PMN2A_1732
3761_Prochlorococcus_marinus_CCMP1375_Pro0394_rfaG
3761_Prochlorococcus_marinus_MIT9315_P9515_04591
3761_Prochlorococcus_marinus_CCMP_1986_PMM0397
3761_Prochlorococcus_marinus_MIT9215_P9215_04741
3761_Prochlorococcus_marinus_MIT9312_PMT9312_0393
3761_Prochlorococcus_marinus_MIT9301_P9301_04171
3761_Prochlorococcus_marinus_AS9601_A9601_04481
1/1
1/1
1/1
0.53/--
1/1
0.64/0.60
0.97/1
0.54/--
0.97/0.93
1/1
1/1
DgdA (BI/ML)
0.5

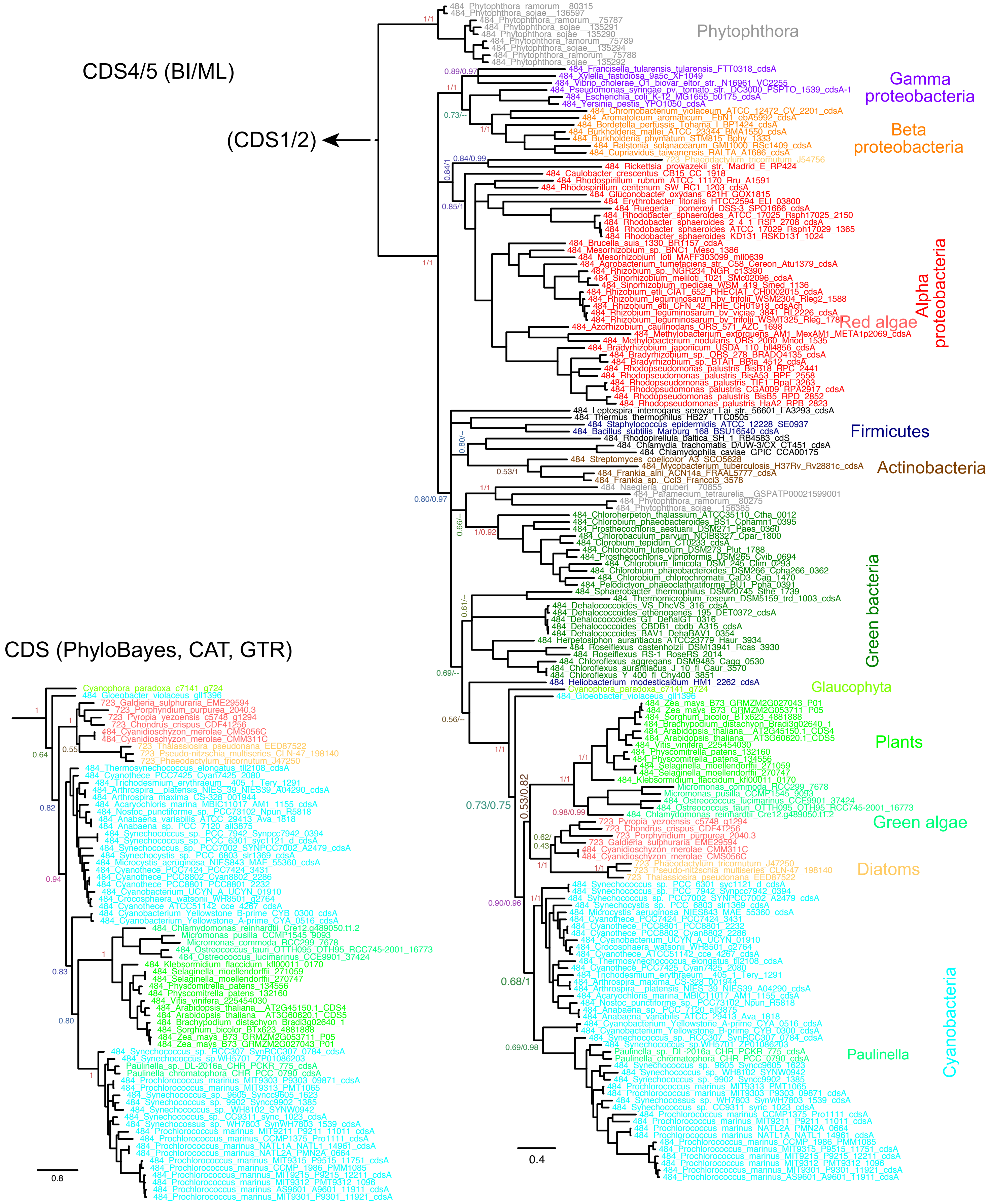

CDS4/5 (BI/ML)
(CDS1/2)
Phytophthora
Gamma proteobacteria
Beta proteobacteria
Alpha proteobacteria
Red algae
Firmicutes
Actinobacteria
Green bacteria
Glaucophyta
Plants
Green algae
Diatoms
Paulinella
Cyanobacteria
0.4
CDS (PhyloBayes, CAT, GTR)
0.8

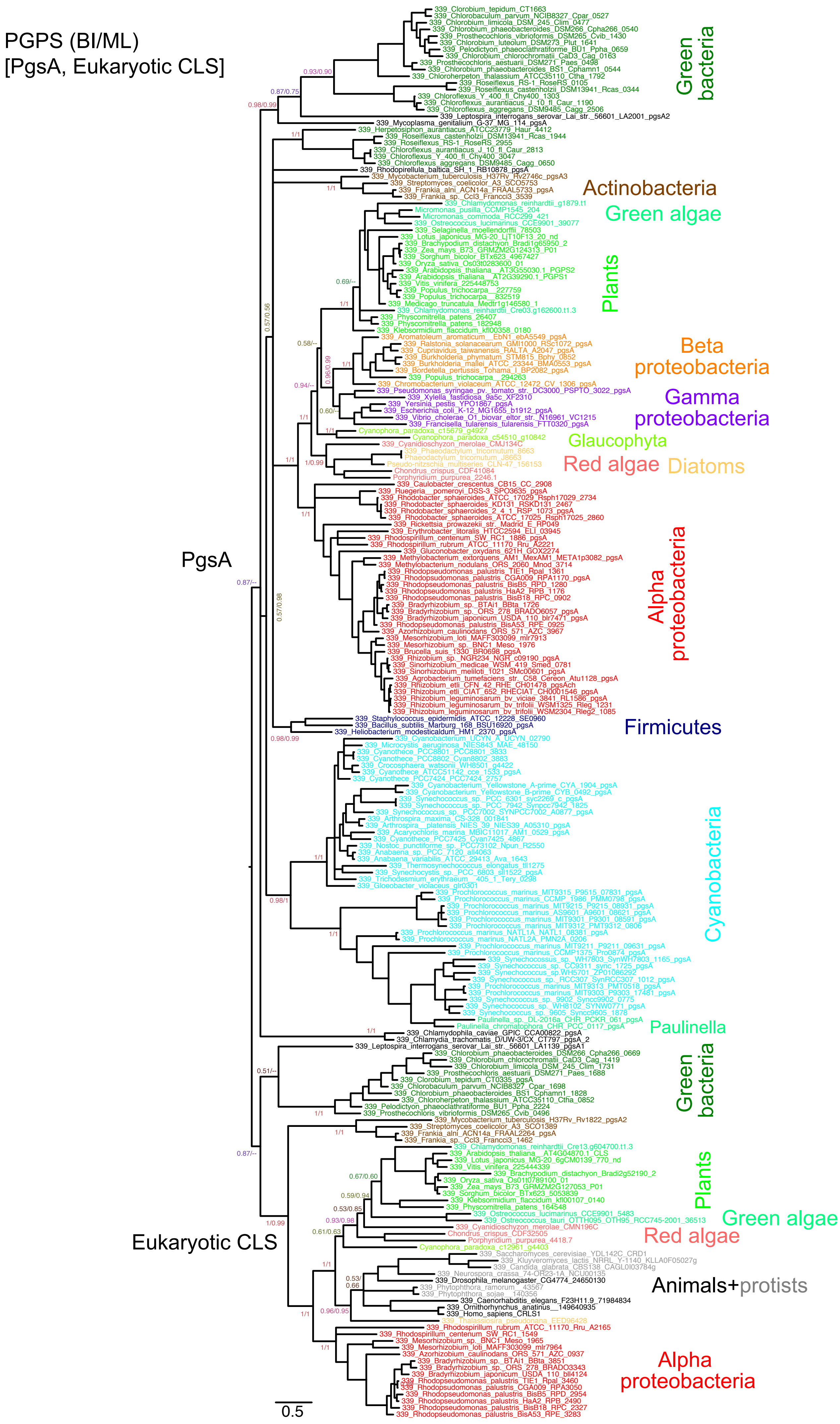

PGPS (BI/ML)
[PgsA, Eukaryotic CLS]
PgsA
Eukaryotic CLS
Green bacteria
Actinobacteria
Green algae
Plants
Beta proteobacteria
Gamma proteobacteria
Glaucophyta
Red algae
Diatoms
Alpha proteobacteria
Firmicutes
Cyanobacteria
Paulinella
Green bacteria
Plants
Green algae
Red algae
Animals+protists
Alpha proteobacteria
0.5

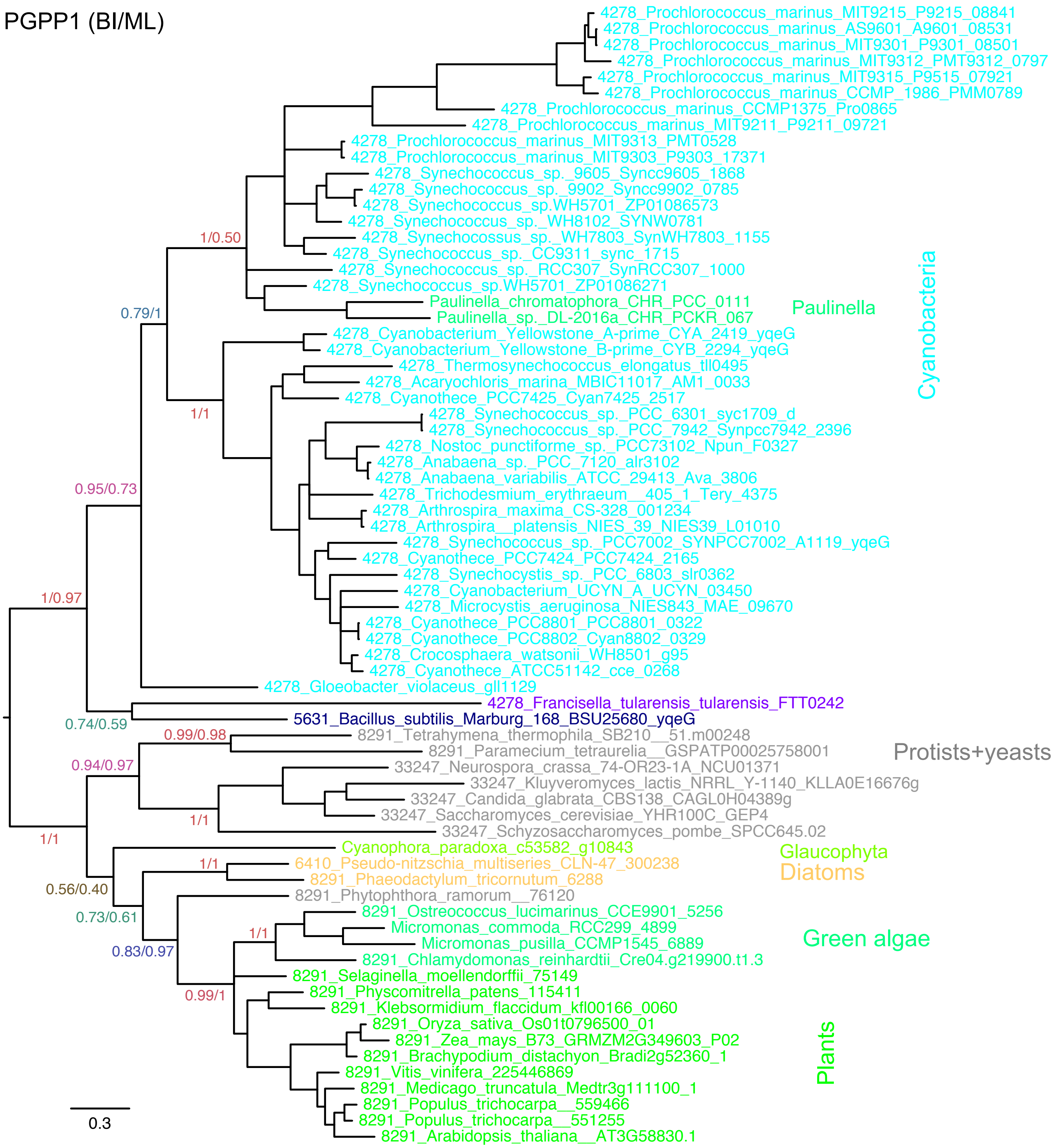
PGPP1 (BI/ML)
4278_Prochlorococcus_marinus_MIT9215_P9215_08841
4278_Prochlorococcus_marinus_AS9601_A9601_08531
4278_Prochlorococcus_marinus_MIT9301_P9301_08501
4278_Prochlorococcus_marinus_MIT9312_PMT9312_0797
4278_Prochlorococcus_marinus_MIT9315_P9515_07921
4278_Prochlorococcus_marinus_CCMP_1986_PMM0789
4278_Prochlorococcus_marinus_CCMP1375_Pro0865
4278_Prochlorococcus_marinus_MIT9211_P9211_09721
4278_Prochlorococcus_marinus_MIT9313_PMT0528
4278_Prochlorococcus_marinus_MIT9303_P9303_17371
4278_Synechococcus_sp._9605_Syncc9605_1868
4278_Synechococcus_sp._9902_Syncc9902_0785
4278_Synechococcus_sp.WH5701_ZP01086573
4278_Synechococcus_sp._WH8102_SYNW0781
4278_Synechocossus_sp._WH7803_SynWH7803_1155
4278_Synechococcus_sp._CC9311_sync_1715
4278_Synechococcus_sp._RCC307_SynRCC307_1000
4278_Synechococcus_sp.WH5701_ZP01086271
Paulinella_chromatophora_CHR_PCC_0111
Paulinella_sp._DL-2016a_CHR_PCKR_067
4278_Cyanobacterium_Yellowstone_A-prime_CYA_2419_yqeG
4278_Cyanobacterium_Yellowstone_B-prime_CYB_2294_yqeG
4278_Thermosynechococcus_elongatus_tll0495
4278_Acaryochloris_marina_MBIC11017_AM1_0033
4278_Cyanothece_PCC7425_Cyan7425_2517
4278_Synechococcus_sp._PCC_6301_syc1709_d
4278_Synechococcus_sp._PCC_7942_Synpcc7942_2396
4278_Nostoc_punctiforme_sp._PCC73102_Npun_F0327
4278_Anabaena_sp._PCC_7120_alr3102
4278_Anabaena_variabilis_ATCC_29413_Ava_3806
4278_Trichodesmium_erythraeum__405_1_Tery_4375
4278_Arthrospira_maxima_CS-328_001234
4278_Arthrospira__platensis_NIES_39_NIES39_L01010
4278_Synechococcus_sp._PCC7002_SYNPCC7002_A1119_yqeG
4278_Cyanothece_PCC7424_PCC7424_2165
4278_Synechocystis_sp._PCC_6803_slr0362
4278_Cyanobacterium_UCYN_A_UCYN_03450
4278_Microcystis_aeruginosa_NIES843_MAE_09670
4278_Cyanothece_PCC8801_PCC8801_0322
4278_Cyanothece_PCC8802_Cyan8802_0329
4278_Crocosphaera_watsonii_WH8501_g95
4278_Cyanothece_ATCC51142_cce_0268
4278_Gloeobacter_violaceus_gll1129
4278_Francisella_tularensis_tularensis_FTT0242
5631_Bacillus_subtilis_Marburg_168_BSU25680_yqeG
8291_Tetrahymena_thermophila_SB210__51.m00248
8291_Paramecium_tetraurelia__GSPATP00025758001
33247_Neurospora_crassa_74-OR23-1A_NCU01371
33247_Kluyveromyces_lactis_NRRL_Y-1140_KLLA0E16676g
33247_Candida_glabrata_CBS138_CAGL0H04389g
33247_Saccharomyces_cerevisiae_YHR100C_GEP4
33247_Schyzosaccharomyces_pombe_SPCC645.02
Cyanophora_paradoxa_c53582_g10843
6410_Pseudo-nitzschia_multiseries_CLN-47_300238
8291_Phaeodactylum_tricornutum_6288
8291_Phytophthora_ramorum__76120
8291_Ostreococcus_lucimarinus_CCE9901_5256
Micromonas_commoda_RCC299_4899
Micromonas_pusilla_CCMP1545_6889
8291_Chlamydomonas_reinhardtii_Cre04.g219900.t1.3
8291_Selaginella_moellendorffii_75149
8291_Physcomitrella_patens_115411
8291_Klebsormidium_flaccidum_kfl00166_0060
8291_Oryza_sativa_Os01t0796500_01
8291_Zea_mays_B73_GRMZM2G349603_P02
8291_Brachypodium_distachyon_Bradi2g52360_1
8291_Vitis_vinifera_225446869
8291_Medicago_truncatula_Medtr3g111100_1
8291_Populus_trichocarpa__559466
8291_Populus_trichocarpa__551255
8291_Arabidopsis_thaliana__AT3G58830.1
1/0.50
0.79/1
1/1
0.95/0.73
1/0.97
0.74/0.59
0.99/0.98
0.94/0.97
1/1
1/1
1/1
0.56/0.40
0.73/0.61
0.83/0.97
1/1
0.99/1
Paulinella
Cyanobacteria
Protists+yeasts
Glaucophyta
Diatoms
Green algae
Plants
0.3

SQD1/SqdB (BI/ML)

Similar splitting of green and red lineages in PhyloBayes, CAT, GTR

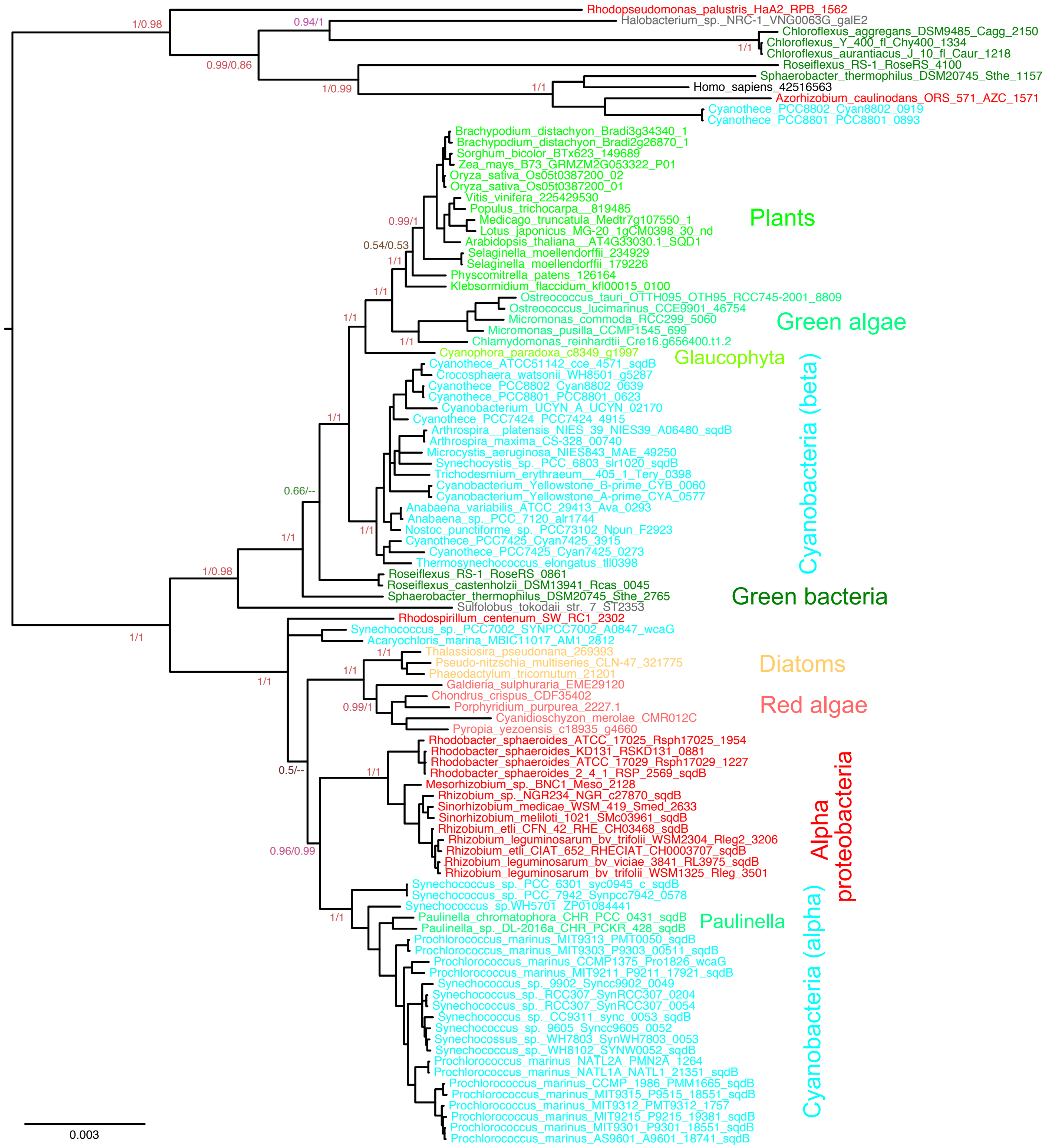

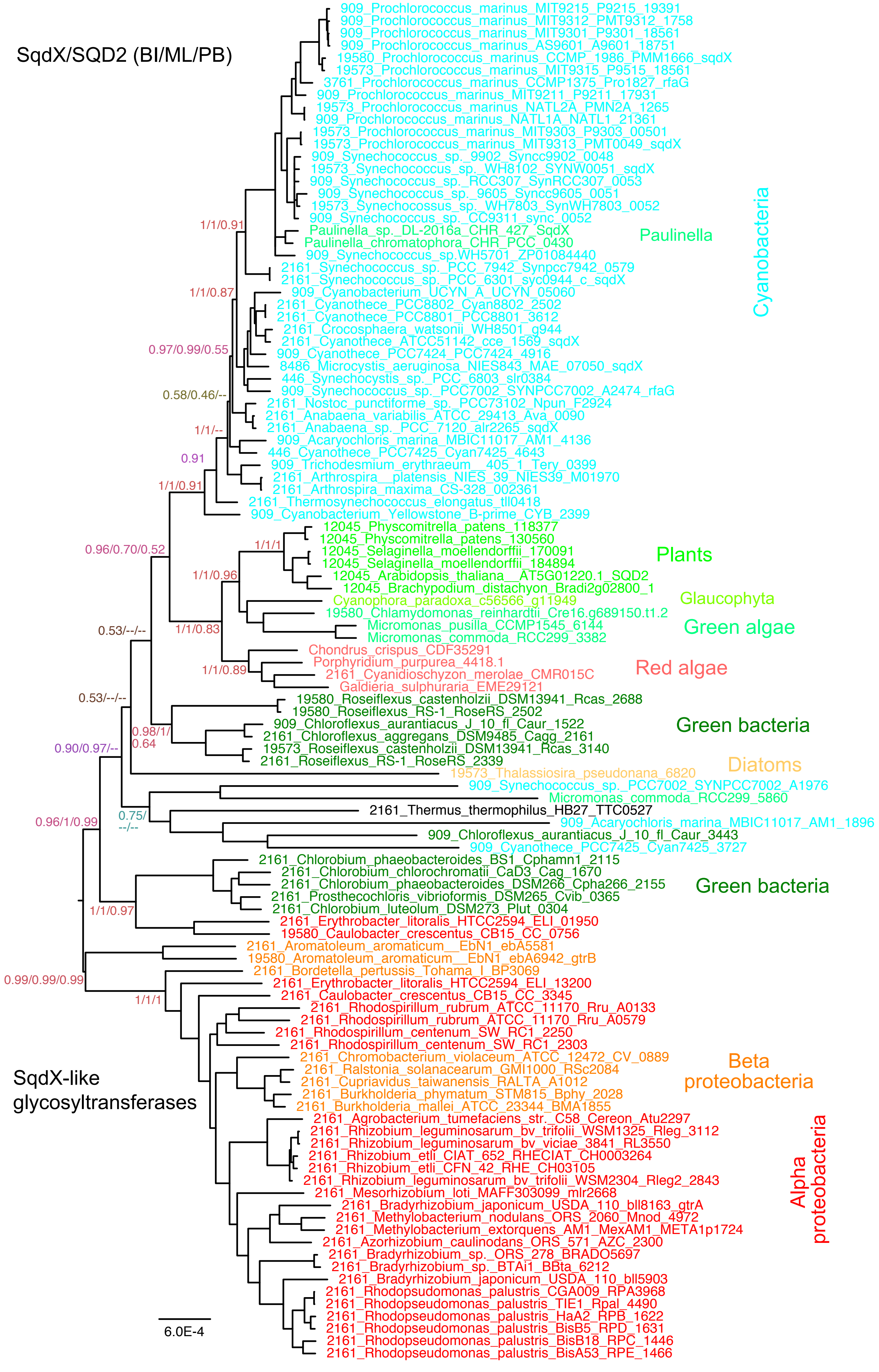
SqdX/SQD2 (BI/ML/PB)
909_Prochlorococcus_marinus_MIT9215_P9215_19391
909_Prochlorococcus_marinus_MIT9312_PMT9312_1758
909_Prochlorococcus_marinus_MIT9301_P9301_18561
909_Prochlorococcus_marinus_AS9601_A9601_18751
19580_Prochlorococcus_marinus_CCMP_1986_PMM1666_sqdX
19573_Prochlorococcus_marinus_MIT9315_P9515_18561
3761_Prochlorococcus_marinus_CCMP1375_Pro1827_rfaG
909_Prochlorococcus_marinus_MIT9211_P9211_17931
19573_Prochlorococcus_marinus_NATL2A_PMN2A_1265
909_Prochlorococcus_marinus_NATL1A_NATL1_21361
19573_Prochlorococcus_marinus_MIT9303_P9303_00501
19573_Prochlorococcus_marinus_MIT9313_PMT0049_sqdX
909_Synechococcus_sp._9902_Syncc9902_0048
19573_Synechococcus_sp._WH8102_SYNW0051_sqdX
909_Synechococcus_sp._RCC307_SynRCC307_0053
909_Synechococcus_sp._9605_Syncc9605_0051
19573_Synechocossus_sp._WH7803_SynWH7803_0052
909_Synechococcus_sp._CC9311_sync_0052
Paulinella_sp._DL-2016a_CHR_427_SqdX
Paulinella_chromatophora_CHR_PCC_0430
909_Synechococcus_sp.WH5701_ZP01084440
2161_Synechococcus_sp._PCC_7942_Synpcc7942_0579
2161_Synechococcus_sp._PCC_6301_syc0944_c_sqdX
909_Cyanobacterium_UCYN_A_UCYN_05060
2161_Cyanothece_PCC8802_Cyan8802_2502
2161_Cyanothece_PCC8801_PCC8801_3612
2161_Crocosphaera_watsonii_WH8501_g944
2161_Cyanothece_ATCC51142_cce_1569_sqdX
909_Cyanothece_PCC7424_PCC7424_4916
8486_Microcystis_aeruginosa_NIES843_MAE_07050_sqdX
446_Synechocystis_sp._PCC_6803_slr0384
909_Synechococcus_sp._PCC7002_SYNPCC7002_A2474_rfaG
2161_Nostoc_punctiforme_sp._PCC73102_Npun_F2924
2161_Anabaena_variabilis_ATCC_29413_Ava_0090
2161_Anabaena_sp._PCC_7120_alr2265_sqdX
909_Acaryochloris_marina_MBIC11017_AM1_4136
446_Cyanothece_PCC7425_Cyan7425_4643
909_Trichodesmium_erythraeum__405_1_Tery_0399
2161_Arthrospira__platensis_NIES_39_NIES39_M01970
2161_Arthrospira_maxima_CS-328_002361
2161_Thermosynechococcus_elongatus_tll0418
909_Cyanobacterium_Yellowstone_B-prime_CYB_2399
12045_Physcomitrella_patens_118377
12045_Physcomitrella_patens_130560
12045_Selaginella_moellendorffii_170091
12045_Selaginella_moellendorffii_184894
12045_Arabidopsis_thaliana__AT5G01220.1_SQD2
12045_Brachypodium_distachyon_Bradi2g02800_1
Cyanophora_paradoxa_c56566_g11949
19580_Chlamydomonas_reinhardtii_Cre16.g689150.t1.2
Micromonas_pusilla_CCMP1545_6144
Micromonas_commoda_RCC299_3382
Chondrus_crispus_CDF35291
Porphyridium_purpurea_4418.1
2161_Cyanidioschyzon_merolae_CMR015C
Galdieria_sulphuraria_EME29121
19580_Roseiflexus_castenholzii_DSM13941_Rcas_2688
19580_Roseiflexus_RS-1_RoseRS_2502
909_Chloroflexus_aurantiacus_J_10_fl_Caur_1522
2161_Chloroflexus_aggregans_DSM9485_Cagg_2161
19573_Roseiflexus_castenholzii_DSM13941_Rcas_3140
2161_Roseiflexus_RS-1_RoseRS_2339
19573_Thalassiosira_pseudonana_6820
909_Synechococcus_sp._PCC7002_SYNPCC7002_A1976
Micromonas_commoda_RCC299_5860
2161_Thermus_thermophilus_HB27_TTC0527
909_Acaryochloris_marina_MBIC11017_AM1_1896
909_Chloroflexus_aurantiacus_J_10_fl_Caur_3443
909_Cyanothece_PCC7425_Cyan7425_3727
2161_Chlorobium_phaeobacteroides_BS1_Cphamn1_2115
2161_Chlorobium_chlorochromatii_CaD3_Cag_1670
2161_Chlorobium_phaeobacteroides_DSM266_Cpha266_2155
2161_Prosthecochloris_vibrioformis_DSM265_Cvib_0365
2161_Chlorobium_luteolum_DSM273_Plut_0304
2161_Erythrobacter_litoralis_HTCC2594_ELI_01950
19580_Caulobacter_crescentus_CB15_CC_0756
2161_Aromatoleum_aromaticum__EbN1_ebA5581
19580_Aromatoleum_aromaticum__EbN1_ebA6942_gtrB
2161_Bordetella_pertussis_Tohama_I_BP3069
2161_Erythrobacter_litoralis_HTCC2594_ELI_13200
2161_Caulobacter_crescentus_CB15_CC_3345
2161_Rhodospirillum_rubrum_ATCC_11170_Rru_A0133
2161_Rhodospirillum_rubrum_ATCC_11170_Rru_A0579
2161_Rhodospirillum_centenum_SW_RC1_2250
2161_Rhodospirillum_centenum_SW_RC1_2303
2161_Chromobacterium_violaceum_ATCC_12472_CV_0889
2161_Ralstonia_solanacearum_GMI1000_RSc2084
2161_Cupriavidus_taiwanensis_RALTA_A1012
2161_Burkholderia_phymatum_STM815_Bphy_2028
2161_Burkholderia_mallei_ATCC_23344_BMA1855
2161_Agrobacterium_tumefaciens_str._C58_Cereon_Atu2297
2161_Rhizobium_leguminosarum_bv_trifolii_WSM1325_Rleg_3112
2161_Rhizobium_leguminosarum_bv_viciae_3841_RL3550
2161_Rhizobium_etli_CIAT_652_RHECIAT_CH0003264
2161_Rhizobium_etli_CFN_42_RHE_CH03105
2161_Rhizobium_leguminosarum_bv_trifolii_WSM2304_Rleg2_2843
2161_Mesorhizobium_loti_MAFF303099_mlr2668
2161_Bradyrhizobium_japonicum_USDA_110_bll8163_gtrA
2161_Methylobacterium_nodulans_ORS_2060_Mnod_4972
2161_Methylobacterium_extorquens_AM1_MexAM1_META1p1724
2161_Azorhizobium_caulinodans_ORS_571_AZC_2300
2161_Bradyrhizobium_sp._ORS_278_BRADO5697
2161_Bradyrhizobium_sp._BTAi1_BBta_6212
2161_Bradyrhizobium_japonicum_USDA_110_bll5903
2161_Rhodopsudomonas_palustris_CGA009_RPA3968
2161_Rhodopseudomonas_palustris_TIE1_Rpal_4490
2161_Rhodopseudomonas_palustris_HaA2_RPB_1622
2161_Rhodopseudomonas_palustris_BisB5_RPD_1631
2161_Rhodopseudomonas_palustris_BisB18_RPC_1446
2161_Rhodopseudomonas_palustris_BisA53_RPE_1466
Paulinella
Cyanobacteria
Plants
Glaucophyta
Green algae
Red algae
Green bacteria
Diatoms
Green bacteria
Beta proteobacteria
Alpha proteobacteria
SqdX-like glycosyltransferases
1/1/0.91
1/1/0.87
0.97/0.99/0.55
0.58/0.46/--
1/1/--
0.91
1/1/0.91
0.96/0.70/0.52
1/1/1
1/1/0.96
1/1/0.83
0.53/--/--
1/1/0.89
0.53/--/--
0.98/1/0.64
0.90/0.97/--
0.75/--/--
0.96/1/0.99
1/1/0.97
0.99/0.99/0.99
1/1/1
6.0E-4
