# Supplementary material for: Complex origins of chloroplast membranes with photosynthetic machineries: multiple transfers of genes from divergent organisms at different times or a single endosymbiotic event?
Source: J Plant Res. 2019 Dec 6;133(1):15–33. doi: 10.1007/s10265-019-01157-z (PMC6946739; doi:10.1007/s10265-019-01157-z)
Supplement: Supplementary file 3 — Supplementary material 3 (PDF 3645 kb) [file 10265_2019_1157_MOESM3_ESM.pdf]

Journal of Plant Research

Online Resources

Complex origins of chloroplast membranes with photosynthetic machineries: Multiple transfers of genes from divergent organisms at different times or a single endosymbiotic event?

Naoki Sato

University of Tokyo

naokisat@bio.c.u-tokyo.ac.jp

Online Resource 3. Phylogenetic trees of enzymes involved in chloroplast fatty acid synthesis.

AccA, B, C, and D, subunits of acetyl-CoA carboxylase; ACP, acyl carrier protein; FabBF, 3-oxoacyl-ACP synthase KAS I/II; FabD, malonyl transferase; FabG, 3-oxoacyl-ACP reductase; FabI, enoyl-ACP reductase; FabZ, 3-hydroxylacyl-ACP dehydratase; FadA, palmitoyl phosphatidylglycerol 3-*trans* desaturase; KAS III, 3-oxoacyl-ACP synthase acting in the initial step; SAD, stearoyl-ACP desaturase. An additional tree of desaturases, which was reconstructed using the data in Sato and Moriyama (2007), is also presented at the end of file. Locate the enzymes in the biosynthetic pathway in Fig. 1 panels A and B.

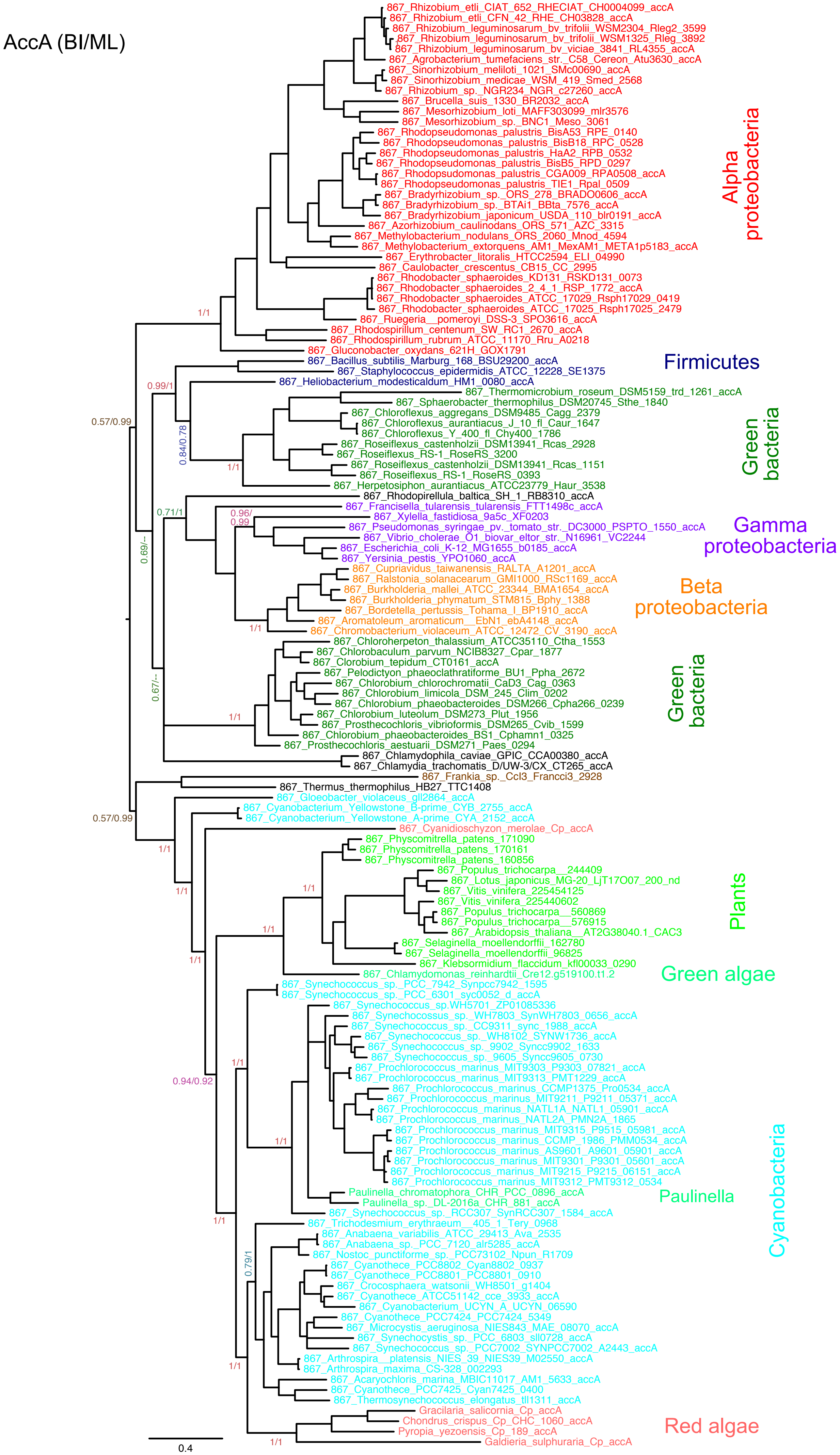
AccA (BI/ML)
867_Rhizobium_etli_CIAT_652_RHECIAT_CH0004099_accA
867_Rhizobium_etli_CFN_42_RHE_CH03828_accA
867_Rhizobium_leguminosarum_bv_trifolii_WSM2304_Rleg2_3599
867_Rhizobium_leguminosarum_bv_trifolii_WSM1325_Rleg_3892
867_Rhizobium_leguminosarum_bv_viciae_3841_RL4355_accA
867_Agrobacterium_tumefaciens_str._C58_Cereon_Atu3630_accA
867_Sinorhizobium_meliloti_1021_SMc00690_accA
867_Sinorhizobium_medicae_WSM_419_Smed_2568
867_Rhizobium_sp._NGR234_NGR_c27260_accA
867_Brucella_suis_1330_BR2032_accA
867_Mesorhizobium_loti_MAFF303099_mlr3576
867_Mesorhizobium_sp._BNC1_Meso_3061
867_Rhodopseudomonas_palustris_BisA53_RPE_0140
867_Rhodopseudomonas_palustris_BisB18_RPC_0528
867_Rhodopseudomonas_palustris_HaA2_RPB_0532
867_Rhodopseudomonas_palustris_BisB5_RPD_0297
867_Rhodopsudomonas_palustris_CGA009_RPA0508_accA
867_Rhodopseudomonas_palustris_TIE1_Rpal_0509
867_Bradyrhizobium_sp._ORS_278_BRADO0606_accA
867_Bradyrhizobium_sp._BTAi1_BBta_7576_accA
867_Bradyrhizobium_japonicum_USDA_110_blr0191_accA
867_Azorhizobium_caulinodans_ORS_571_AZC_3315
867_Methylobacterium_nodulans_ORS_2060_Mnod_4594
867_Methylobacterium_extorquens_AM1_MexAM1_META1p5183_accA
867_Erythrobacter_litoralis_HTCC2594_ELI_04990
867_Caulobacter_crescentus_CB15_CC_2995
867_Rhodobacter_sphaeroides_KD131_RSKD131_0073
867_Rhodobacter_sphaeroides_2_4_1_RSP_1772_accA
867_Rhodobacter_sphaeroides_ATCC_17029_Rsph17029_0419
867_Rhodobacter_sphaeroides_ATCC_17025_Rsph17025_2479
867_Ruegeria__pomeroyi_DSS-3_SPO3616_accA
867_Rhodospirillum_centenum_SW_RC1_2670_accA
867_Rhodospirillum_rubrum_ATCC_11170_Rru_A0218
867_Gluconobacter_oxydans_621H_GOX1791
Alpha proteobacteria
1/1
867_Bacillus_subtilis_Marburg_168_BSU29200_accA
867_Staphylococcus_epidermidis_ATCC_12228_SE1375
867_Heliobacterium_modesticaldum_HM1_0080_accA
Firmicutes
0.99/1
0.57/0.99
0.84/0.78
867_Thermomicrobium_roseum_DSM5159_trd_1261_accA
867_Sphaerobacter_thermophilus_DSM20745_Sthe_1840
867_Chloroflexus_aggregans_DSM9485_Cagg_2379
867_Chloroflexus_aurantiacus_J_10_fl_Caur_1647
867_Chloroflexus_Y_400_fl_Chy400_1786
867_Roseiflexus_castenholzii_DSM13941_Rcas_2928
867_Roseiflexus_RS-1_RoseRS_3200
867_Roseiflexus_castenholzii_DSM13941_Rcas_1151
867_Roseiflexus_RS-1_RoseRS_0393
867_Herpetosiphon_aurantiacus_ATCC23779_Haur_3538
1/1
Green bacteria
867_Rhodopirellula_baltica_SH_1_RB8310_accA
867_Francisella_tularensis_tularensis_FTT1498c_accA
867_Xylella_fastidiosa_9a5c_XF0203
867_Pseudomonas_syringae_pv._tomato_str._DC3000_PSPTO_1550_accA
867_Vibrio_cholerae_O1_biovar_eltor_str._N16961_VC2244
867_Escherichia_coli_K-12_MG1655_b0185_accA
867_Yersinia_pestis_YPO1060_accA
0.71/1
0.96/
0.99
0.69/--
Gamma proteobacteria
867_Cupriavidus_taiwanensis_RALTA_A1201_accA
867_Ralstonia_solanacearum_GMI1000_RSc1169_accA
867_Burkholderia_mallei_ATCC_23344_BMA1654_accA
867_Burkholderia_phymatum_STM815_Bphy_1388
867_Bordetella_pertussis_Tohama_I_BP1910_accA
867_Aromatoleum_aromaticum__EbN1_ebA4148_accA
867_Chromobacterium_violaceum_ATCC_12472_CV_3190_accA
1/1
Beta proteobacteria
867_Chloroherpeton_thalassium_ATCC35110_Ctha_1553
867_Chlorobaculum_parvum_NCIB8327_Cpar_1877
867_Clorobium_tepidum_CT0161_accA
867_Pelodictyon_phaeoclathratiforme_BU1_Ppha_2672
867_Chlorobium_chlorochromatii_CaD3_Cag_0363
867_Chlorobium_limicola_DSM_245_Clim_0202
867_Chlorobium_phaeobacteroides_DSM266_Cpha266_0239
867_Chlorobium_luteolum_DSM273_Plut_1956
867_Prosthecochloris_vibrioformis_DSM265_Cvib_1599
867_Chlorobium_phaeobacteroides_BS1_Cphamn1_0325
867_Prosthecochloris_aestuarii_DSM271_Paes_0294
0.67/--
1/1
Green bacteria
867_Chlamydophila_caviae_GPIC_CCA00380_accA
867_Chlamydia_trachomatis_D/UW-3/CX_CT265_accA
867_Frankia_sp._CcI3_Francci3_2928
867_Thermus_thermophilus_HB27_TTC1408
867_Gloeobacter_violaceus_gll2864_accA
867_Cyanobacterium_Yellowstone_B-prime_CYB_2755_accA
867_Cyanobacterium_Yellowstone_A-prime_CYA_2152_accA
867_Cyanidioschyzon_merolae_Cp_accA
0.57/0.99
1/1
867_Physcomitrella_patens_171090
867_Physcomitrella_patens_170161
867_Physcomitrella_patens_160856
867_Populus_trichocarpa__244409
867_Lotus_japonicus_MG-20_LjT17O07_200_nd
867_Vitis_vinifera_225454125
867_Vitis_vinifera_225440602
867_Populus_trichocarpa__560869
867_Populus_trichocarpa__576915
867_Arabidopsis_thaliana__AT2G38040.1_CAC3
867_Selaginella_moellendorffii_162780
867_Selaginella_moellendorffii_96825
867_Klebsormidium_flaccidum_kfl00033_0290
1/1
1/1
1/1
1/1
Plants
867_Chlamydomonas_reinhardtii_Cre12.g519100.t1.2
Green algae
867_Synechococcus_sp._PCC_7942_Synpcc7942_1595
867_Synechococcus_sp._PCC_6301_syc0052_d_accA
867_Synechococcus_sp.WH5701_ZP01085336
867_Synechocossus_sp._WH7803_SynWH7803_0656_accA
867_Synechococcus_sp._CC9311_sync_1988_accA
867_Synechococcus_sp._WH8102_SYNW1736_accA
867_Synechococcus_sp._9902_Syncc9902_1633
867_Synechococcus_sp._9605_Syncc9605_0730
867_Prochlorococcus_marinus_MIT9303_P9303_07821_accA
867_Prochlorococcus_marinus_MIT9313_PMT1229_accA
867_Prochlorococcus_marinus_CCMP1375_Pro0534_accA
867_Prochlorococcus_marinus_MIT9211_P9211_05371_accA
867_Prochlorococcus_marinus_NATL1A_NATL1_05901_accA
867_Prochlorococcus_marinus_NATL2A_PMN2A_1865
867_Prochlorococcus_marinus_MIT9315_P9515_05981_accA
867_Prochlorococcus_marinus_CCMP_1986_PMM0534_accA
867_Prochlorococcus_marinus_AS9601_A9601_05901_accA
867_Prochlorococcus_marinus_MIT9301_P9301_05601_accA
867_Prochlorococcus_marinus_MIT9215_P9215_06151_accA
867_Prochlorococcus_marinus_MIT9312_PMT9312_0534
0.94/0.92
1/1
1/1
Paulinella_chromatophora_CHR_PCC_0896_accA
Paulinella_sp._DL-2016a_CHR_881_accA
Paulinella
867_Synechococcus_sp._RCC307_SynRCC307_1584_accA
867_Trichodesmium_erythraeum__405_1_Tery_0968
1/1
867_Anabaena_variabilis_ATCC_29413_Ava_2535
867_Anabaena_sp._PCC_7120_alr5285_accA
867_Nostoc_punctiforme_sp._PCC73102_Npun_R1709
867_Cyanothece_PCC8802_Cyan8802_0937
867_Cyanothece_PCC8801_PCC8801_0910
867_Crocosphaera_watsonii_WH8501_g1404
867_Cyanothece_ATCC51142_cce_3933_accA
867_Cyanobacterium_UCYN_A_UCYN_06590
867_Cyanothece_PCC7424_PCC7424_5349
867_Microcystis_aeruginosa_NIES843_MAE_08070_accA
867_Synechocystis_sp._PCC_6803_sll0728_accA
867_Synechococcus_sp._PCC7002_SYNPCC7002_A2443_accA
867_Arthrospira__platensis_NIES_39_NIES39_M02550_accA
867_Arthrospira_maxima_CS-328_002293
867_Acaryochloris_marina_MBIC11017_AM1_5633_accA
867_Cyanothece_PCC7425_Cyan7425_0400
867_Thermosynechococcus_elongatus_tll1311_accA
0.79/1
1/1
Cyanobacteria
Gracilaria_salicornia_Cp_accA
Chondrus_crispus_Cp_CHC_1060_accA
Pyropia_yezoensis_Cp_189_accA
Galdieria_sulphuraria_Cp_accA
1/1
Red algae
0.4

Representative topologies of AccB (ML)

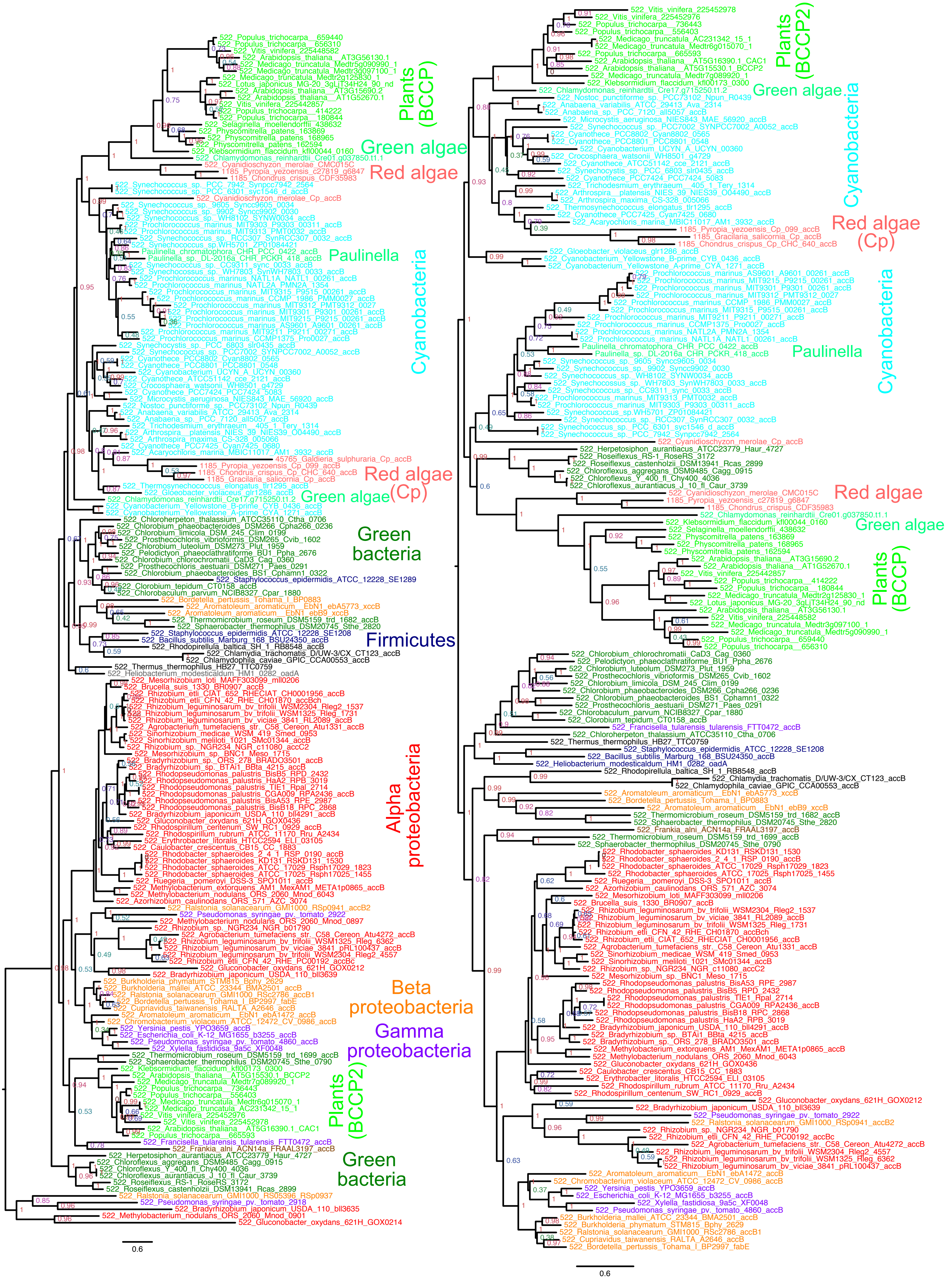

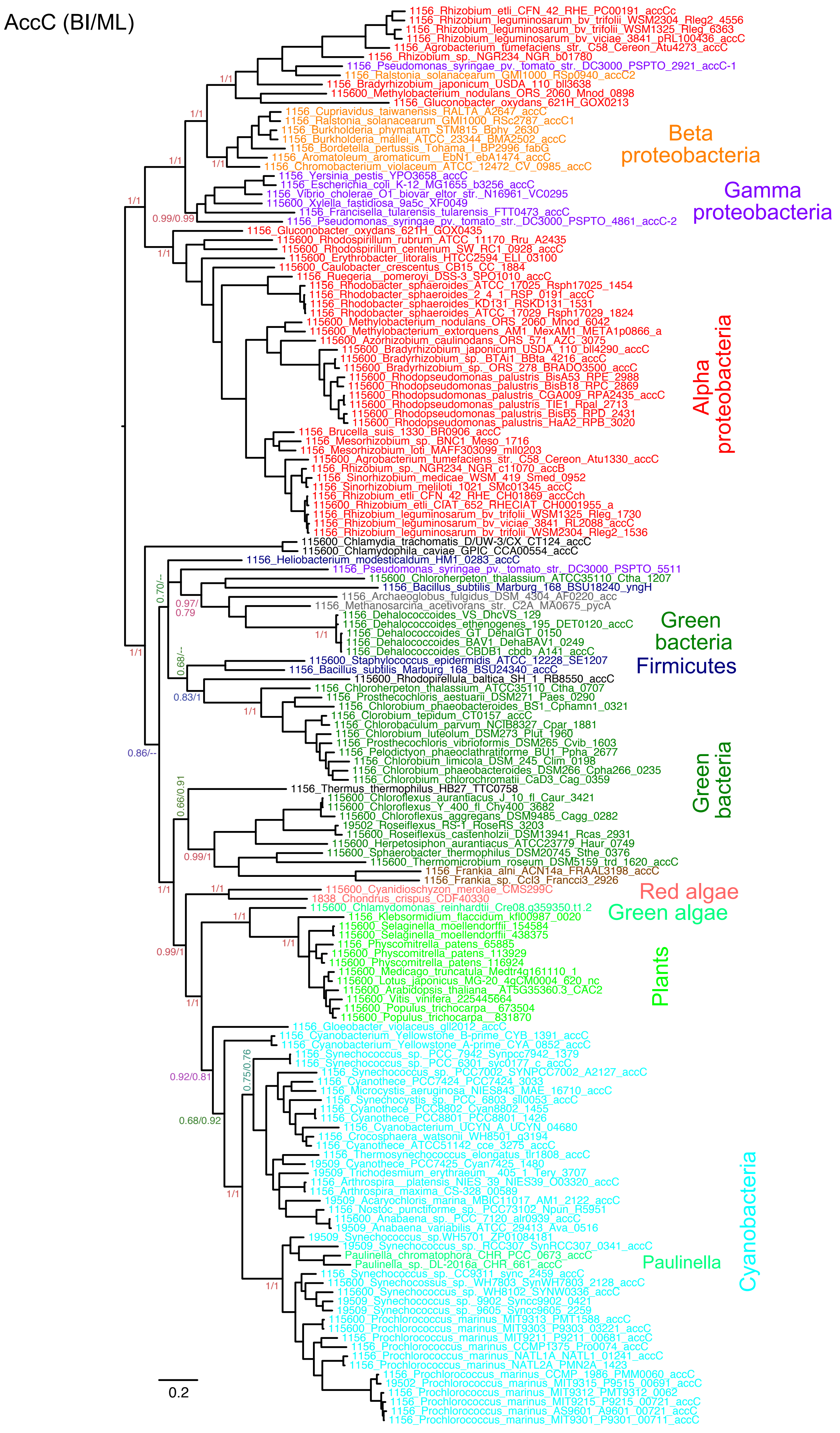
AccC (BI/ML)
1156_Rhizobium_etli_CFN_42_RHE_PC00191_accCc
1156_Rhizobium_leguminosarum_bv_trifolii_WSM2304_Rleg2_4556
1156_Rhizobium_leguminosarum_bv_trifolii_WSM1325_Rleg_6363
1156_Rhizobium_leguminosarum_bv_viciae_3841_pRL100436_accC
1156_Agrobacterium_tumefaciens_str._C58_Cereon_Atu4273_accC
1156_Rhizobium_sp._NGR234_NGR_b01780
1156_Pseudomonas_syringae_pv._tomato_str._DC3000_PSPTO_2921_accC-1
1156_Ralstonia_solanacearum_GMI1000_RSp0940_accC2
1156_Bradyrhizobium_japonicum_USDA_110_bll3638
115600_Methylobacterium_nodulans_ORS_2060_Mnod_0898
1156_Gluconobacter_oxydans_621H_GOX0213
1156_Cupriavidus_taiwanensis_RALTA_A2647_accC
1156_Ralstonia_solanacearum_GMI1000_RSc2787_accC1
1156_Burkholderia_phymatum_STM815_Bphy_2630
1156_Burkholderia_mallei_ATCC_23344_BMA2502_accC
1156_Bordetella_pertussis_Tohama_I_BP2996_fabG
1156_Aromatoleum_aromaticum_EbN1_ebA1474_accC
1156_Chromobacterium_violaceum_ATCC_12472_CV_0985_accC
Beta proteobacteria
1156_Yersinia_pestis_YPO3658_accC
1156_Escherichia_coli_K-12_MG1655_b3256_accC
1156_Vibrio_cholerae_O1_biovar_eltor_str._N16961_VC0295
115600_Xylella_fastidiosa_9a5c_XF0049
1156_Francisella_tularensis_tularensis_FTT0473_accC
1156_Pseudomonas_syringae_pv._tomato_str._DC3000_PSPTO_4861_accC-2
Gamma proteobacteria
1156_Gluconobacter_oxydans_621H_GOX0435
115600_Rhodospirillum_rubrum_ATCC_11170_Rru_A2435
115600_Rhodospirillum_centenum_SW_RC1_0928_accC
115600_Erythrobacter_litoralis_HTCC2594_ELI_03100
115600_Caulobacter_crescentus_CB15_CC_1884
1156_Ruegeria_pomeroyi_DSS-3_SPO1010_accC
1156_Rhodobacter_sphaeroides_ATCC_17025_Rsph17025_1454
1156_Rhodobacter_sphaeroides_2_4_1_RSP_0191_accC
1156_Rhodobacter_sphaeroides_KD131_RSKD131_1531
1156_Rhodobacter_sphaeroides_ATCC_17029_Rsph17029_1824
115600_Methylobacterium_nodulans_ORS_2060_Mnod_6042
115600_Methylobacterium_extorquens_AM1_MexAM1_META1p0866_a
115600_Azorhizobium_caulinodans_ORS_571_AZC_3075
115600_Bradyrhizobium_japonicum_USDA_110_bll4290_accC
115600_Bradyrhizobium_sp._BTAi1_BBta_4216_accC
115600_Bradyrhizobium_sp._ORS_278_BRADO3500_accC
115600_Rhodopseudomonas_palustris_BisA53_RPE_2988
115600_Rhodopseudomonas_palustris_BisB18_RPC_2869
115600_Rhodopsudomonas_palustris_CGA009_RPA2435_accC
115600_Rhodopseudomonas_palustris_TIE1_Rpal_2713
115600_Rhodopseudomonas_palustris_BisB5_RPD_2431
115600_Rhodopseudomonas_palustris_HaA2_RPB_3020
Alpha proteobacteria
1156_Brucella_suis_1330_BR0906_accC
1156_Mesorhizobium_sp._BNC1_Meso_1716
1156_Mesorhizobium_loti_MAFF303099_mll0203
115600_Agrobacterium_tumefaciens_str._C58_Cereon_Atu1330_accC
1156_Rhizobium_sp._NGR234_NGR_c11070_accB
1156_Sinorhizobium_medicae_WSM_419_Smed_0952
1156_Sinorhizobium_meliloti_1021_SMc01345_accC
1156_Rhizobium_etli_CFN_42_RHE_CH01869_accCch
115600_Rhizobium_etli_CIAT_652_RHECIAT_CH0001955_a
1156_Rhizobium_leguminosarum_bv_trifolii_WSM1325_Rleg_1730
1156_Rhizobium_leguminosarum_bv_viciae_3841_RL2088_accC
1156_Rhizobium_leguminosarum_bv_trifolii_WSM2304_Rleg2_1536
115600_Chlamydia_trachomatis_D/UW-3/CX_CT124_accC
115600_Chlamydophila_caviae_GPIC_CCA00554_accC
1156_Heliobacterium_modesticaldum_HM1_0283_accC
1156_Pseudomonas_syringae_pv._tomato_str._DC3000_PSPTO_5511
115600_Chloroherpeton_thalassium_ATCC35110_Ctha_1207
1156_Bacillus_subtilis_Marburg_168_BSU18240_yngH
1156_Archaeoglobus_fulgidus_DSM_4304_AF0220_acc
1156_Methanosarcina_acetivorans_str._C2A_MA0675_pycA
1156_Dehalococcoides_VS_DhcVS_129
1156_Dehalococcoides_ethenogenes_195_DET0120_accC
1156_Dehalococcoides_GT_DehalGT_0150
1156_Dehalococcoides_BAV1_DehaBAV1_0249
1156_Dehalococcoides_CBDB1_cbdb_A141_accC
Green bacteria
115600_Staphylococcus_epidermidis_ATCC_12228_SE1207
1156_Bacillus_subtilis_Marburg_168_BSU24340_accC
Firmicutes
115600_Rhodopirellula_baltica_SH_1_RB8550_accC
1156_Chloroherpeton_thalassium_ATCC35110_Ctha_0707
1156_Prosthecochloris_aestuarii_DSM271_Paes_0290
1156_Chlorobium_phaeobacteroides_BS1_Cphamn1_0321
1156_Clorobium_tepidum_CT0157_accC
1156_Chlorobaculum_parvum_NCIB8327_Cpar_1881
1156_Chlorobium_luteolum_DSM273_Plut_1960
1156_Prosthecochloris_vibrioformis_DSM265_Cvib_1603
1156_Pelodictyon_phaeoclathratiforme_BU1_Ppha_2677
1156_Chlorobium_limicola_DSM_245_Clim_0198
1156_Chlorobium_phaeobacteroides_DSM266_Cpha266_0235
1156_Chlorobium_chlorochromatii_CaD3_Cag_0359
Green bacteria
1156_Thermus_thermophilus_HB27_TTC0758
115600_Chloroflexus_aurantiacus_J_10_fl_Caur_3421
115600_Chloroflexus_Y_400_fl_Chy400_3682
115600_Chloroflexus_aggregans_DSM9485_Cagg_0282
19502_Roseiflexus_RS-1_RoseRS_3203
115600_Roseiflexus_castenholzii_DSM13941_Rcas_2931
115600_Herpetosiphon_aurantiacus_ATCC23779_Haur_0749
115600_Sphaerobacter_thermophilus_DSM20745_Sthe_0376
115600_Thermomicrobium_roseum_DSM5159_trd_1620_accC
1156_Frankia_alni_ACN14a_FRAAL3198_accC
1156_Frankia_sp._CcI3_Francci3_2926
115600_Cyanidioschyzon_merolae_CMS299C
1838_Chondrus_crispus_CDF40330
Red algae
115600_Chlamydomonas_reinhardtii_Cre08.g359350.t1.2
1156_Klebsormidium_flaccidum_kfl00987_0020
Green algae
115600_Selaginella_moellendorffii_154584
115600_Selaginella_moellendorffii_438375
1156_Physcomitrella_patens_65885
115600_Physcomitrella_patens_113929
115600_Physcomitrella_patens_116924
115600_Medicago_truncatula_Medtr4g161110_1
115600_Lotus_japonicus_MG-20_4gCM0004_620_nc
115600_Arabidopsis_thaliana_AT5G35360.3_CAC2
115600_Vitis_vinifera_225445664
115600_Populus_trichocarpa_673504
115600_Populus_trichocarpa_831870
Plants
1156_Gloeobacter_violaceus_gll2012_accC
1156_Cyanobacterium_Yellowstone_B-prime_CYB_1391_accC
1156_Cyanobacterium_Yellowstone_A-prime_CYA_0852_accC
1156_Synechococcus_sp._PCC_7942_Synpcc7942_1379
1156_Synechococcus_sp._PCC_6301_syc0177_c_accC
1156_Synechococcus_sp._PCC7002_SYNPCC7002_A2127_accC
1156_Cyanothece_PCC7424_PCC7424_3033
1156_Microcystis_aeruginosa_NIES843_MAE_16710_accC
1156_Synechocystis_sp._PCC_6803_sll0053_accC
1156_Cyanothece_PCC8802_Cyan8802_1455
1156_Cyanothece_PCC8801_PCC8801_1426
1156_Cyanobacterium_UCYN_A_UCYN_04680
1156_Crocosphaera_watsonii_WH8501_g3194
1156_Cyanothece_ATCC51142_cce_3275_accC
1156_Thermosynechococcus_elongatus_tlr1808_accC
19509_Cyanothece_PCC7425_Cyan7425_1480
19509_Trichodesmium_erythraeum_405_1_Tery_3707
1156_Arthrospira_platensis_NIES_39_NIES39_O03320_accC
1156_Arthrospira_maxima_CS-328_00589
19509_Acaryochloris_marina_MBIC11017_AM1_2122_accC
1156_Nostoc_punctiforme_sp._PCC73102_Npun_R5951
115600_Anabaena_sp._PCC_7120_alr0939_accC
19509_Anabaena_variabilis_ATCC_29413_Ava_0516
19509_Synechococcus_sp.WH5701_ZP01084181
19509_Synechococcus_sp._RCC307_SynRCC307_0341_accC
Paulinella_chromatophora_CHR_PCC_0673_accC
Paulinella_sp._DL-2016a_CHR_661_accC
Paulinella
1156_Synechococcus_sp._CC9311_sync_2459_accC
115600_Synechocossus_sp._WH7803_SynWH7803_2128_accC
115600_Synechococcus_sp._WH8102_SYNW0336_accC
19509_Synechococcus_sp._9902_Syncc9902_0421
19509_Synechococcus_sp._9605_Syncc9605_2259
115600_Prochlorococcus_marinus_MIT9313_PMT1588_accC
115600_Prochlorococcus_marinus_MIT9303_P9303_03221_accC
1156_Prochlorococcus_marinus_MIT9211_P9211_00681_accC
1156_Prochlorococcus_marinus_CCMP1375_Pro0074_accC
1156_Prochlorococcus_marinus_NATL1A_NATL1_01241_accC
1156_Prochlorococcus_marinus_NATL2A_PMN2A_1423
1156_Prochlorococcus_marinus_CCMP_1986_PMM0060_accC
19502_Prochlorococcus_marinus_MIT9315_P9515_00691_accC
1156_Prochlorococcus_marinus_MIT9312_PMT9312_0062
1156_Prochlorococcus_marinus_MIT9215_P9215_00721_accC
1156_Prochlorococcus_marinus_AS9601_A9601_00721_accC
1156_Prochlorococcus_marinus_MIT9301_P9301_00711_accC
Cyanobacteria
0.2

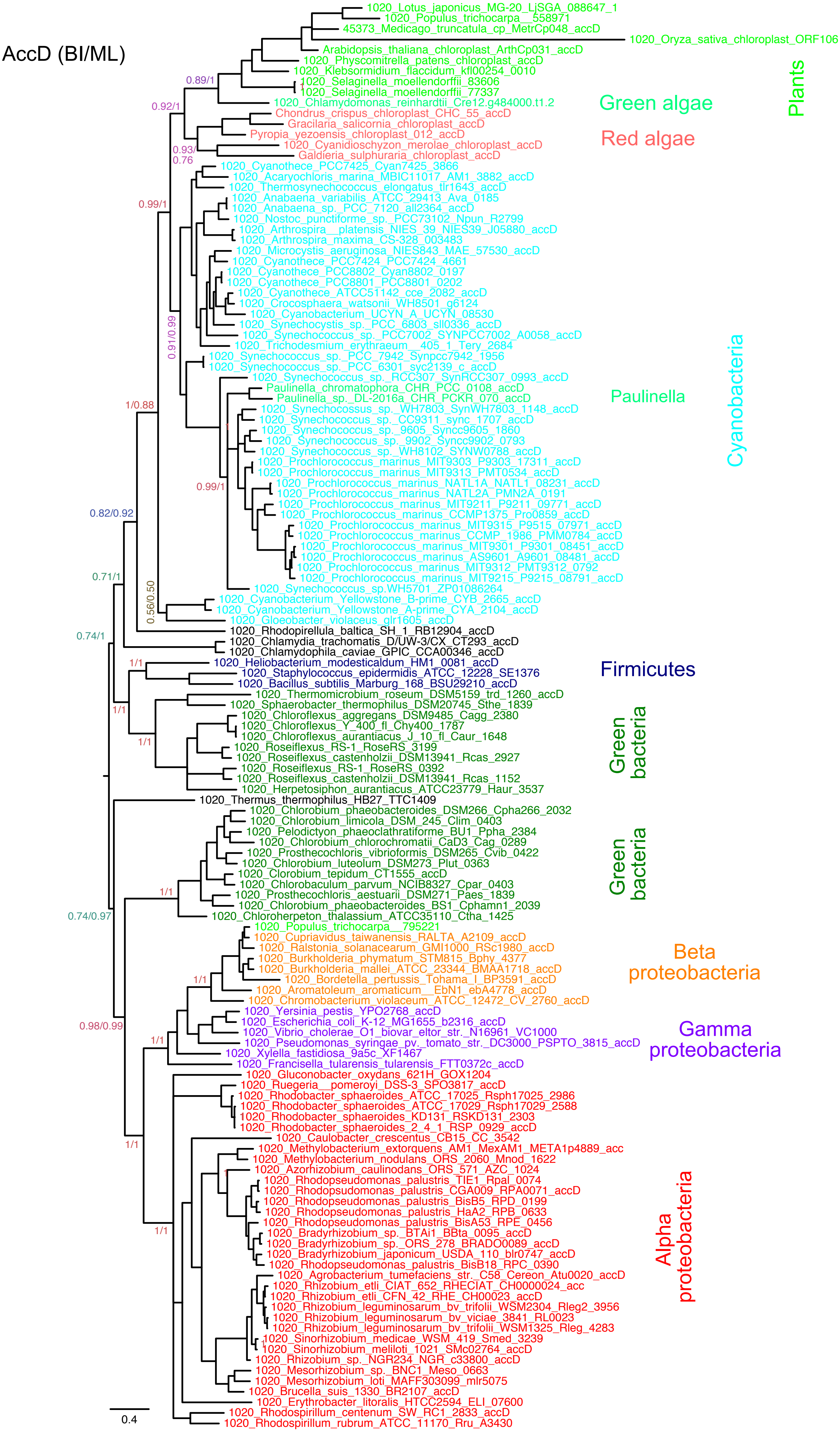

AccD (BI/ML)
1020_Lotus_japonicus_MG-20_LjSGA_088647_1
1020_Populus_trichocarpa__558971
45373_Medicago_truncatula_cp_MetrCp048_accD
1020_Oryza_sativa_chloroplast_ORF106
Arabidopsis_thaliana_chloroplast_ArthCp031_accD
1020_Physcomitrella_patens_chloroplast_accD
1020_Klebsormidium_flaccidum_kfl00254_0010
1020_Selaginella_moellendorffii_83606
1020_Selaginella_moellendorffii_77337
1020_Chlamydomonas_reinhardtii_Cre12.g484000.t1.2
Chondrus_crispus_chloroplast_CHC_55_accD
Gracilaria_salicornia_chloroplast_accD
Pyropia_yezoensis_chloroplast_012_accD
1020_Cyanidioschyzon_merolae_chloroplast_accD
Galdieria_sulphuraria_chloroplast_accD
1020_Cyanothece_PCC7425_Cyan7425_3866
1020_Acaryochloris_marina_MBIC11017_AM1_3882_accD
1020_Thermosynechococcus_elongatus_tlr1643_accD
1020_Anabaena_variabilis_ATCC_29413_Ava_0185
1020_Anabaena_sp._PCC_7120_all2364_accD
1020_Nostoc_punctiforme_sp._PCC73102_Npun_R2799
1020_Arthrospira__platensis_NIES_39_NIES39_J05880_accD
1020_Arthrospira_maxima_CS-328_003483
1020_Microcystis_aeruginosa_NIES843_MAE_57530_accD
1020_Cyanothece_PCC7424_PCC7424_4661
1020_Cyanothece_PCC8802_Cyan8802_0197
1020_Cyanothece_PCC8801_PCC8801_0202
1020_Cyanothece_ATCC51142_cce_2082_accD
1020_Crocosphaera_watsonii_WH8501_g6124
1020_Cyanobacterium_UCYN_A_UCYN_08530
1020_Synechocystis_sp._PCC_6803_sll0336_accD
1020_Synechococcus_sp._PCC7002_SYNPCC7002_A0058_accD
1020_Trichodesmium_erythraeum__405_1_Tery_2684
1020_Synechococcus_sp._PCC_7942_Synpcc7942_1956
1020_Synechococcus_sp._PCC_6301_syc2139_c_accD
1020_Synechococcus_sp._RCC307_SynRCC307_0993_accD
Paulinella_chromatophora_CHR_PCC_0108_accD
Paulinella_sp._DL-2016a_CHR_PCKR_070_accD
1020_Synechocossus_sp._WH7803_SynWH7803_1148_accD
1020_Synechococcus_sp._CC9311_sync_1707_accD
1020_Synechococcus_sp._9605_Syncc9605_1860
1020_Synechococcus_sp._9902_Syncc9902_0793
1020_Synechococcus_sp._WH8102_SYNW0788_accD
1020_Prochlorococcus_marinus_MIT9303_P9303_17311_accD
1020_Prochlorococcus_marinus_MIT9313_PMT0534_accD
1020_Prochlorococcus_marinus_NATL1A_NATL1_08231_accD
1020_Prochlorococcus_marinus_NATL2A_PMN2A_0191
1020_Prochlorococcus_marinus_MIT9211_P9211_09771_accD
1020_Prochlorococcus_marinus_CCMP1375_Pro0859_accD
1020_Prochlorococcus_marinus_MIT9315_P9515_07971_accD
1020_Prochlorococcus_marinus_CCMP_1986_PMM0784_accD
1020_Prochlorococcus_marinus_MIT9301_P9301_08451_accD
1020_Prochlorococcus_marinus_AS9601_A9601_08481_accD
1020_Prochlorococcus_marinus_MIT9312_PMT9312_0792
1020_Prochlorococcus_marinus_MIT9215_P9215_08791_accD
1020_Synechococcus_sp.WH5701_ZP01086264
1020_Cyanobacterium_Yellowstone_B-prime_CYB_2665_accD
1020_Cyanobacterium_Yellowstone_A-prime_CYA_2104_accD
1020_Gloeobacter_violaceus_glr1605_accD
1020_Rhodopirellula_baltica_SH_1_RB12904_accD
1020_Chlamydia_trachomatis_D/UW-3/CX_CT293_accD
1020_Chlamydophila_caviae_GPIC_CCA00346_accD
1020_Heliobacterium_modesticaldum_HM1_0081_accD
1020_Staphylococcus_epidermidis_ATCC_12228_SE1376
1020_Bacillus_subtilis_Marburg_168_BSU29210_accD
1020_Thermomicrobium_roseum_DSM5159_trd_1260_accD
1020_Sphaerobacter_thermophilus_DSM20745_Sthe_1839
1020_Chloroflexus_aggregans_DSM9485_Cagg_2380
1020_Chloroflexus_Y_400_fl_Chy400_1787
1020_Chloroflexus_aurantiacus_J_10_fl_Caur_1648
1020_Roseiflexus_RS-1_RoseRS_3199
1020_Roseiflexus_castenholzii_DSM13941_Rcas_2927
1020_Roseiflexus_RS-1_RoseRS_0392
1020_Roseiflexus_castenholzii_DSM13941_Rcas_1152
1020_Herpetosiphon_aurantiacus_ATCC23779_Haur_3537
1020_Thermus_thermophilus_HB27_TTC1409
1020_Chlorobium_phaeobacteroides_DSM266_Cpha266_2032
1020_Chlorobium_limicola_DSM_245_Clim_0403
1020_Pelodictyon_phaeoclathratiforme_BU1_Ppha_2384
1020_Chlorobium_chlorochromatii_CaD3_Cag_0289
1020_Prosthecochloris_vibrioformis_DSM265_Cvib_0422
1020_Chlorobium_luteolum_DSM273_Plut_0363
1020_Clorobium_tepidum_CT1555_accD
1020_Chlorobaculum_parvum_NCIB8327_Cpar_0403
1020_Prosthecochloris_aestuarii_DSM271_Paes_1839
1020_Chlorobium_phaeobacteroides_BS1_Cphamn1_2039
1020_Chloroherpeton_thalassium_ATCC35110_Ctha_1425
1020_Populus_trichocarpa__795221
1020_Cupriavidus_taiwanensis_RALTA_A2109_accD
1020_Ralstonia_solanacearum_GMI1000_RSc1980_accD
1020_Burkholderia_phymatum_STM815_Bphy_4377
1020_Burkholderia_mallei_ATCC_23344_BMAA1718_accD
1020_Bordetella_pertussis_Tohama_I_BP3591_accD
1020_Aromatoleum_aromaticum__EbN1_ebA4778_accD
1020_Chromobacterium_violaceum_ATCC_12472_CV_2760_accD
1020_Yersinia_pestis_YPO2768_accD
1020_Escherichia_coli_K-12_MG1655_b2316_accD
1020_Vibrio_cholerae_O1_biovar_eltor_str._N16961_VC1000
1020_Pseudomonas_syringae_pv._tomato_str._DC3000_PSPTO_3815_accD
1020_Xylella_fastidiosa_9a5c_XF1467
1020_Francisella_tularensis_tularensis_FTT0372c_accD
1020_Gluconobacter_oxydans_621H_GOX1204
1020_Ruegeria__pomeroyi_DSS-3_SPO3817_accD
1020_Rhodobacter_sphaeroides_ATCC_17025_Rsph17025_2986
1020_Rhodobacter_sphaeroides_ATCC_17029_Rsph17029_2588
1020_Rhodobacter_sphaeroides_KD131_RSKD131_2303
1020_Rhodobacter_sphaeroides_2_4_1_RSP_0929_accD
1020_Caulobacter_crescentus_CB15_CC_3542
1020_Methylobacterium_extorquens_AM1_MexAM1_META1p4889_acc
1020_Methylobacterium_nodulans_ORS_2060_Mnod_1622
1020_Azorhizobium_caulinodans_ORS_571_AZC_1024
1020_Rhodopseudomonas_palustris_TIE1_Rpal_0074
1020_Rhodopseudomonas_palustris_CGA009_RPA0071_accD
1020_Rhodopseudomonas_palustris_BisB5_RPD_0199
1020_Rhodopseudomonas_palustris_HaA2_RPB_0633
1020_Rhodopseudomonas_palustris_BisA53_RPE_0456
1020_Bradyrhizobium_sp._BTAi1_BBta_0095_accD
1020_Bradyrhizobium_sp._ORS_278_BRADO0089_accD
1020_Bradyrhizobium_japonicum_USDA_110_blr0747_accD
1020_Rhodopseudomonas_palustris_BisB18_RPC_0390
1020_Agrobacterium_tumefaciens_str._C58_Cereon_Atu0020_accD
1020_Rhizobium_etli_CIAT_652_RHECIAT_CH0000024_acc
1020_Rhizobium_etli_CFN_42_RHE_CH00023_accD
1020_Rhizobium_leguminosarum_bv_trifolii_WSM2304_Rleg2_3956
1020_Rhizobium_leguminosarum_bv_viciae_3841_RL0023
1020_Rhizobium_leguminosarum_bv_trifolii_WSM1325_Rleg_4283
1020_Sinorhizobium_medicae_WSM_419_Smed_3239
1020_Sinorhizobium_meliloti_1021_SMc02764_accD
1020_Rhizobium_sp._NGR234_NGR_c33800_accD
1020_Mesorhizobium_sp._BNC1_Meso_0663
1020_Mesorhizobium_loti_MAFF303099_mlr5075
1020_Brucella_suis_1330_BR2107_accD
1020_Erythrobacter_litoralis_HTCC2594_ELI_07600
1020_Rhodospirillum_centenum_SW_RC1_2833_accD
1020_Rhodospirillum_rubrum_ATCC_11170_Rru_A3430
0.89/1
0.92/1
0.93/
0.76
0.99/1
0.91/0.99
1/0.88
0.99/1
0.82/0.92
0.71/1
0.56/0.50
0.74/1
1/1
1/1
1/1
0.74/0.97
1/1
1/1
0.98/0.99
1/1
1/1
1/1
0.4
Plants
Green algae
Red algae
Cyanobacteria
Paulinella
Firmicutes
Green bacteria
Green bacteria
Beta proteobacteria
Gamma proteobacteria
Alpha proteobacteria

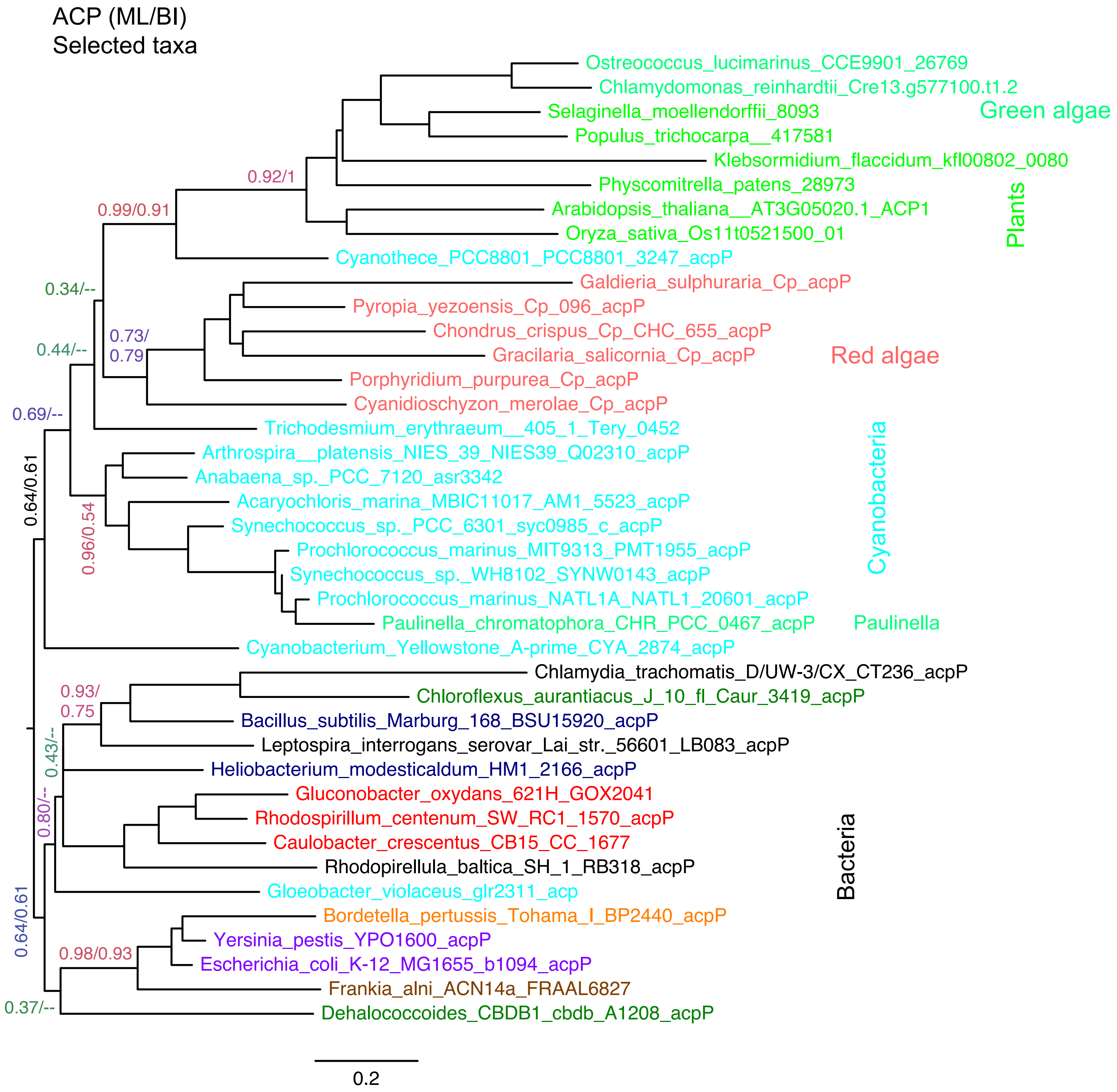
ACP (ML/BI)
Selected taxa
Ostreococcus_lucimarinus_CCE9901_26769
Chlamydomonas_reinhardtii_Cre13.g577100.t1.2
Selaginella_moellendorffii_8093
Populus_trichocarpa__417581
Klebsormidium_flaccidum_kfl00802_0080
Physcomitrella_patens_28973
Arabidopsis_thaliana__AT3G05020.1_ACP1
Oryza_sativa_Os11t0521500_01
Cyanothece_PCC8801_PCC8801_3247_acpP
Galdieria_sulphuraria_Cp_acpP
Pyropia_yezoensis_Cp_096_acpP
Chondrus_crispus_Cp_CHC_655_acpP
Gracilaria_salicornia_Cp_acpP
Porphyridium_purpurea_Cp_acpP
Cyanidioschyzon_merolae_Cp_acpP
Trichodesmium_erythraeum__405_1_Tery_0452
Arthrospira__platensis_NIES_39_NIES39_Q02310_acpP
Anabaena_sp._PCC_7120_asr3342
Acaryochloris_marina_MBIC11017_AM1_5523_acpP
Synechococcus_sp._PCC_6301_syc0985_c_acpP
Prochlorococcus_marinus_MIT9313_PMT1955_acpP
Synechococcus_sp._WH8102_SYNW0143_acpP
Prochlorococcus_marinus_NATL1A_NATL1_20601_acpP
Paulinella_chromatophora_CHR_PCC_0467_acpP
Cyanobacterium_Yellowstone_A-prime_CYA_2874_acpP
Chlamydia_trachomatis_D/UW-3/CX_CT236_acpP
Chloroflexus_aurantiacus_J_10_fl_Caur_3419_acpP
Bacillus_subtilis_Marburg_168_BSU15920_acpP
Leptospira_interrogans_serovar_Lai_str._56601_LB083_acpP
Heliobacterium_modesticaldum_HM1_2166_acpP
Gluconobacter_oxydans_621H_GOX2041
Rhodospirillum_centenum_SW_RC1_1570_acpP
Caulobacter_crescentus_CB15_CC_1677
Rhodopirellula_baltica_SH_1_RB318_acpP
Gloeobacter_violaceus_glr2311_acp
Bordetella_pertussis_Tohama_I_BP2440_acpP
Yersinia_pestis_YPO1600_acpP
Escherichia_coli_K-12_MG1655_b1094_acpP
Frankia_alni_ACN14a_FRAAL6827
Dehalococcoides_CBDB1_cbdb_A1208_acpP
Green algae
Plants
Red algae
Cyanobacteria
Paulinella
Bacteria
0.92/1
0.99/0.91
0.34/--
0.73/
0.79
0.44/--
0.69/--
0.64/0.61
0.96/0.54
0.93/
0.75
0.43/--
0.80/--
0.64/0.61
0.98/0.93
0.37/--
0.2

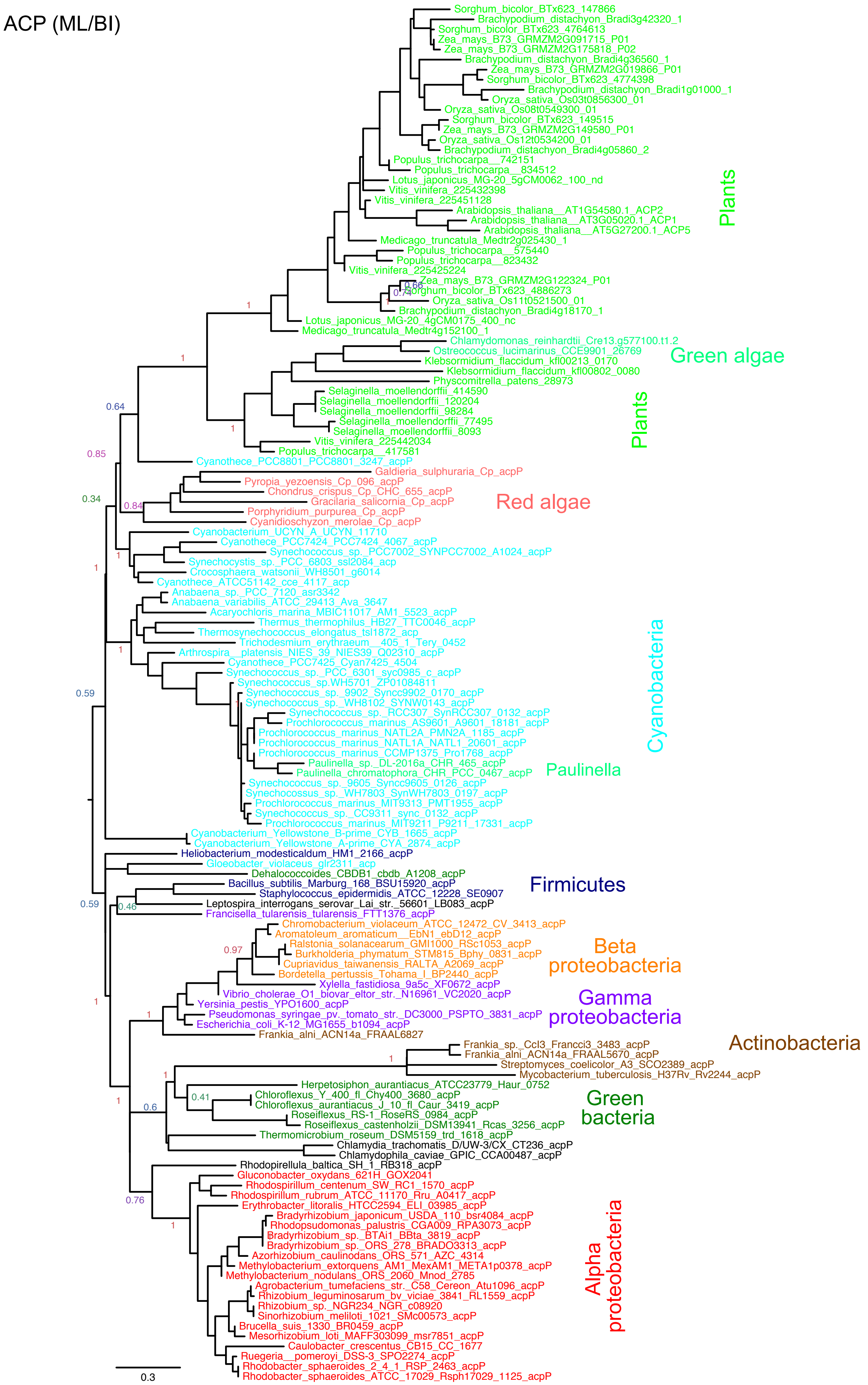

ACP (ML/BI)
Sorghum_bicolor_BTx623_147866
Brachypodium_distachyon_Bradi3g42320_1
Sorghum_bicolor_BTx623_4764613
Zea_mays_B73_GRMZM2G091715_P01
Zea_mays_B73_GRMZM2G175818_P02
Brachypodium_distachyon_Bradi4g36560_1
Zea_mays_B73_GRMZM2G019866_P01
Sorghum_bicolor_BTx623_4774398
Brachypodium_distachyon_Bradi1g01000_1
Oryza_sativa_Os03t0856300_01
Oryza_sativa_Os08t0549300_01
Sorghum_bicolor_BTx623_149515
Zea_mays_B73_GRMZM2G149580_P01
Oryza_sativa_Os12t0534200_01
Brachypodium_distachyon_Bradi4g05860_2
Populus_trichocarpa__742151
Populus_trichocarpa__834512
Lotus_japonicus_MG-20_5gCM0062_100_nd
Vitis_vinifera_225432398
Vitis_vinifera_225451128
Arabidopsis_thaliana__AT1G54580.1_ACP2
Arabidopsis_thaliana__AT3G05020.1_ACP1
Arabidopsis_thaliana__AT5G27200.1_ACP5
Medicago_truncatula_Medtr2g025430_1
Populus_trichocarpa__575440
Populus_trichocarpa__823432
Vitis_vinifera_225425224
Zea_mays_B73_GRMZM2G122324_P01
0.66
Sorghum_bicolor_BTx623_4886273
0.74
Oryza_sativa_Os11t0521500_01
1
Brachypodium_distachyon_Bradi4g18170_1
1
Lotus_japonicus_MG-20_4gCM0175_400_nc
Medicago_truncatula_Medtr4g152100_1
Plants
Chlamydomonas_reinhardtii_Cre13.g577100.t1.2
Ostreococcus_lucimarinus_CCE9901_26769
Klebsormidium_flaccidum_kfl00213_0170
Klebsormidium_flaccidum_kfl00802_0080
Physcomitrella_patens_28973
Green algae
1
Selaginella_moellendorffii_414590
Selaginella_moellendorffii_120204
Selaginella_moellendorffii_98284
Selaginella_moellendorffii_77495
Selaginella_moellendorffii_8093
1
Vitis_vinifera_225442034
Populus_trichocarpa__417581
Plants
0.64
0.85
Cyanothece_PCC8801_PCC8801_3247_acpP
Galdieria_sulphuraria_Cp_acpP
Pyropia_yezoensis_Cp_096_acpP
Chondrus_crispus_Cp_CHC_655_acpP
Gracilaria_salicornia_Cp_acpP
0.34
0.84
Porphyridium_purpurea_Cp_acpP
Cyanidioschyzon_merolae_Cp_acpP
Red algae
Cyanobacterium_UCYN_A_UCYN_11710
Cyanothece_PCC7424_PCC7424_4067_acpP
Synechococcus_sp._PCC7002_SYNPCC7002_A1024_acpP
1
Synechocystis_sp._PCC_6803_ssl2084_acp
1
Crocosphaera_watsonii_WH8501_g6014
Cyanothece_ATCC51142_cce_4117_acp
Anabaena_sp._PCC_7120_asr3342
Anabaena_variabilis_ATCC_29413_Ava_3647
Acaryochloris_marina_MBIC11017_AM1_5523_acpP
Thermus_thermophilus_HB27_TTC0046_acpP
Thermosynechococcus_elongatus_tsl1872_acp
Trichodesmium_erythraeum__405_1_Tery_0452
1
Arthrospira__platensis_NIES_39_NIES39_Q02310_acpP
Cyanothece_PCC7425_Cyan7425_4504
Synechococcus_sp._PCC_6301_syc0985_c_acpP
Synechococcus_sp.WH5701_ZP01084811
0.59
Synechococcus_sp._9902_Syncc9902_0170_acpP
Synechococcus_sp._WH8102_SYNW0143_acpP
1
Synechococcus_sp._RCC307_SynRCC307_0132_acpP
Prochlorococcus_marinus_AS9601_A9601_18181_acpP
Prochlorococcus_marinus_NATL2A_PMN2A_1185_acpP
Prochlorococcus_marinus_NATL1A_NATL1_20601_acpP
Prochlorococcus_marinus_CCMP1375_Pro1768_acpP
Paulinella_sp._DL-2016a_CHR_465_acpP
Paulinella_chromatophora_CHR_PCC_0467_acpP
Paulinella
Synechococcus_sp._9605_Syncc9605_0126_acpP
Synechocossus_sp._WH7803_SynWH7803_0197_acpP
Prochlorococcus_marinus_MIT9313_PMT1955_acpP
Synechococcus_sp._CC9311_sync_0132_acpP
Prochlorococcus_marinus_MIT9211_P9211_17331_acpP
Cyanobacteria
Cyanobacterium_Yellowstone_B-prime_CYB_1665_acpP
Cyanobacterium_Yellowstone_A-prime_CYA_2874_acpP
Heliobacterium_modesticaldum_HM1_2166_acpP
Gloeobacter_violaceus_glr2311_acp
Dehalococcoides_CBDB1_cbdb_A1208_acpP
Bacillus_subtilis_Marburg_168_BSU15920_acpP
Staphylococcus_epidermidis_ATCC_12228_SE0907
Firmicutes
0.59
0.46
Leptospira_interrogans_serovar_Lai_str._56601_LB083_acpP
Francisella_tularensis_tularensis_FTT1376_acpP
Chromobacterium_violaceum_ATCC_12472_CV_3413_acpP
Aromatoleum_aromaticum__EbN1_ebD12_acpP
Ralstonia_solanacearum_GMI1000_RSc1053_acpP
0.97
Burkholderia_phymatum_STM815_Bphy_0831_acpP
Cupriavidus_taiwanensis_RALTA_A2069_acpP
Bordetella_pertussis_Tohama_I_BP2440_acpP
Beta
proteobacteria
Xylella_fastidiosa_9a5c_XF0672_acpP
Vibrio_cholerae_O1_biovar_eltor_str._N16961_VC2020_acpP
1
Yersinia_pestis_YPO1600_acpP
1
Pseudomonas_syringae_pv._tomato_str._DC3000_PSPTO_3831_acpP
Escherichia_coli_K-12_MG1655_b1094_acpP
Gamma
proteobacteria
Frankia_alni_ACN14a_FRAAL6827
Frankia_sp._CcI3_Francci3_3483_acpP
Frankia_alni_ACN14a_FRAAL5670_acpP
1
Streptomyces_coelicolor_A3_SCO2389_acpP
Mycobacterium_tuberculosis_H37Rv_Rv2244_acpP
Actinobacteria
1
Herpetosiphon_aurantiacus_ATCC23779_Haur_0752
0.41
Chloroflexus_Y_400_fl_Chy400_3680_acpP
Chloroflexus_aurantiacus_J_10_fl_Caur_3419_acpP
1
0.6
Roseiflexus_RS-1_RoseRS_0984_acpP
Roseiflexus_castenholzii_DSM13941_Rcas_3256_acpP
Green
bacteria
Thermomicrobium_roseum_DSM5159_trd_1618_acpP
Chlamydia_trachomatis_D/UW-3/CX_CT236_acpP
Chlamydophila_caviae_GPIC_CCA00487_acpP
Rhodopirellula_baltica_SH_1_RB318_acpP
Gluconobacter_oxydans_621H_GOX2041
Rhodospirillum_centenum_SW_RC1_1570_acpP
0.76
Rhodospirillum_rubrum_ATCC_11170_Rru_A0417_acpP
Erythrobacter_litoralis_HTCC2594_ELI_03985_acpP
1
Bradyrhizobium_japonicum_USDA_110_bsr4084_acpP
Rhodopsudomonas_palustris_CGA009_RPA3073_acpP
Bradyrhizobium_sp._BTAi1_BBta_3819_acpP
Bradyrhizobium_sp._ORS_278_BRADO3313_acpP
Azorhizobium_caulinodans_ORS_571_AZC_4314
Methylobacterium_extorquens_AM1_MexAM1_META1p0378_acpP
Methylobacterium_nodulans_ORS_2060_Mnod_2785
Agrobacterium_tumefaciens_str._C58_Cereon_Atu1096_acpP
Rhizobium_leguminosarum_bv_viciae_3841_RL1559_acpP
Rhizobium_sp._NGR234_NGR_c08920
Sinorhizobium_meliloti_1021_SMc00573_acpP
Brucella_suis_1330_BR0459_acpP
Mesorhizobium_loti_MAFF303099_msr7851_acpP
Caulobacter_crescentus_CB15_CC_1677
Ruegeria_pomeroyi_DSS-3_SPO2274_acpP
Rhodobacter_sphaeroides_2_4_1_RSP_2463_acpP
Rhodobacter_sphaeroides_ATCC_17029_Rsph17029_1125_acpP
Alpha
proteobacteria
0.3

ACP (ML)
Large data

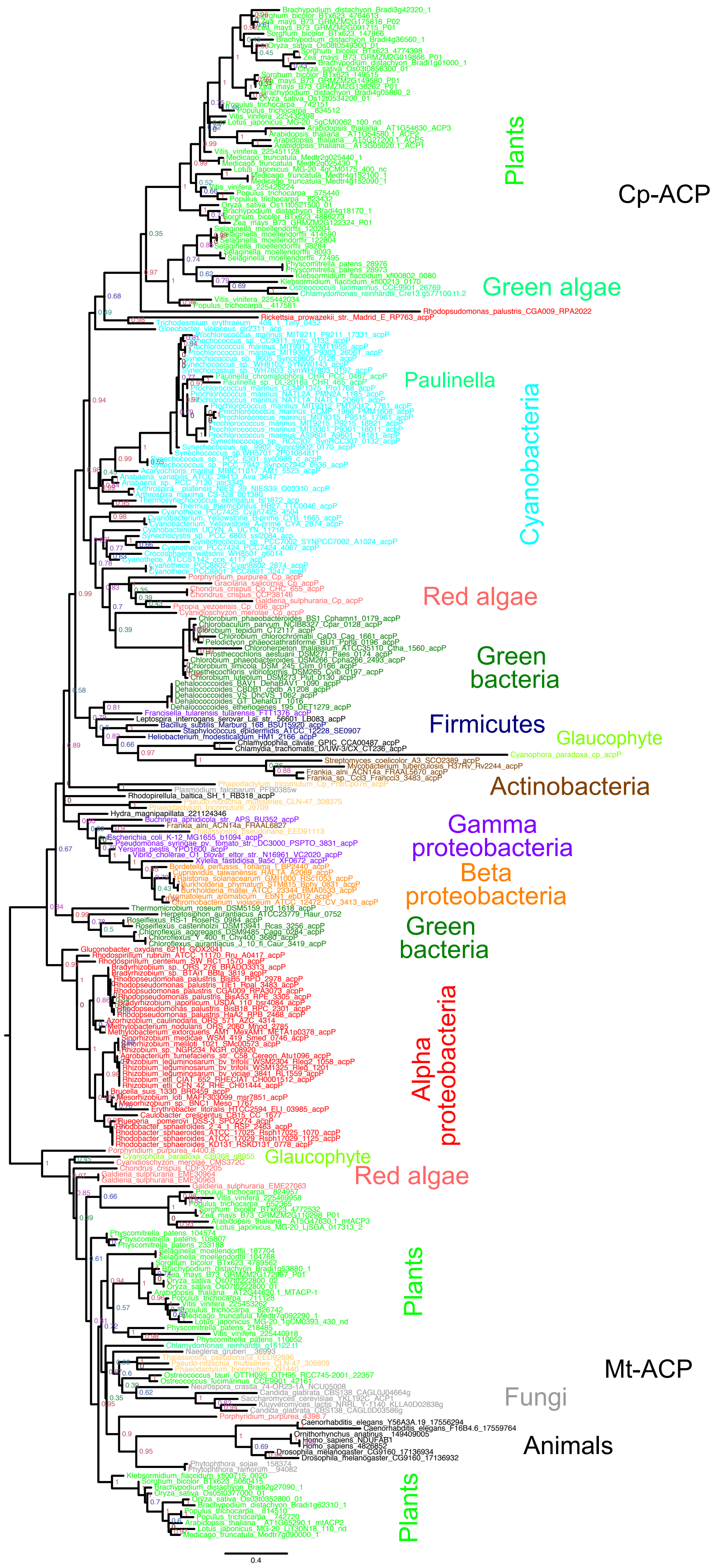

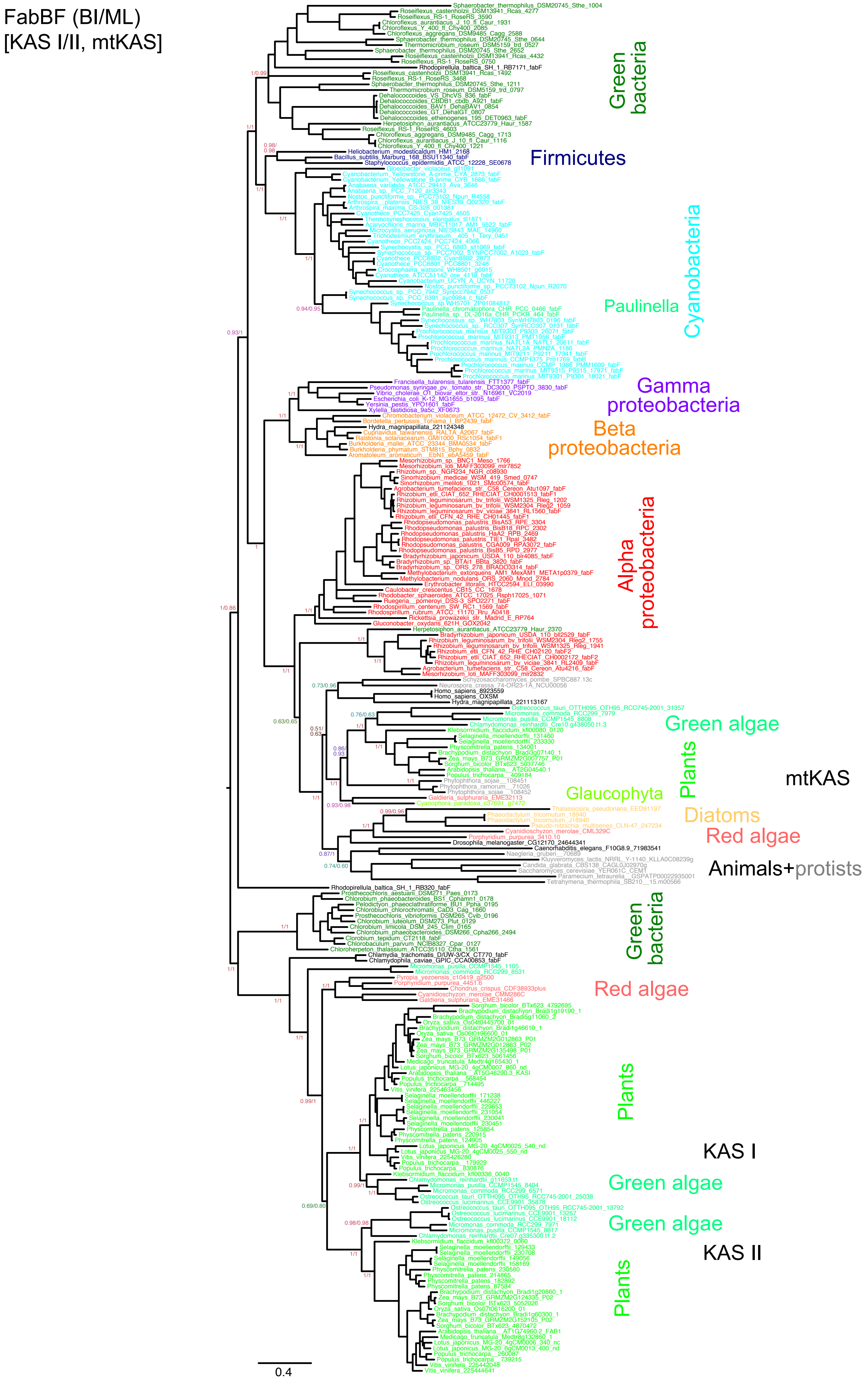
FabBF (BI/ML)
[KAS I/II, mtKAS]
Green bacteria
Firmicutes
Cyanobacteria
Paulinella
Gamma proteobacteria
Beta proteobacteria
Alpha proteobacteria
Green algae
Plants
mtKAS
Glaucophyta
Diatoms
Red algae
Animals+protists
Green bacteria
Red algae
Plants
KAS I
Green algae
Green algae
KAS II
Plants
0.4

FabD (BI/ML)

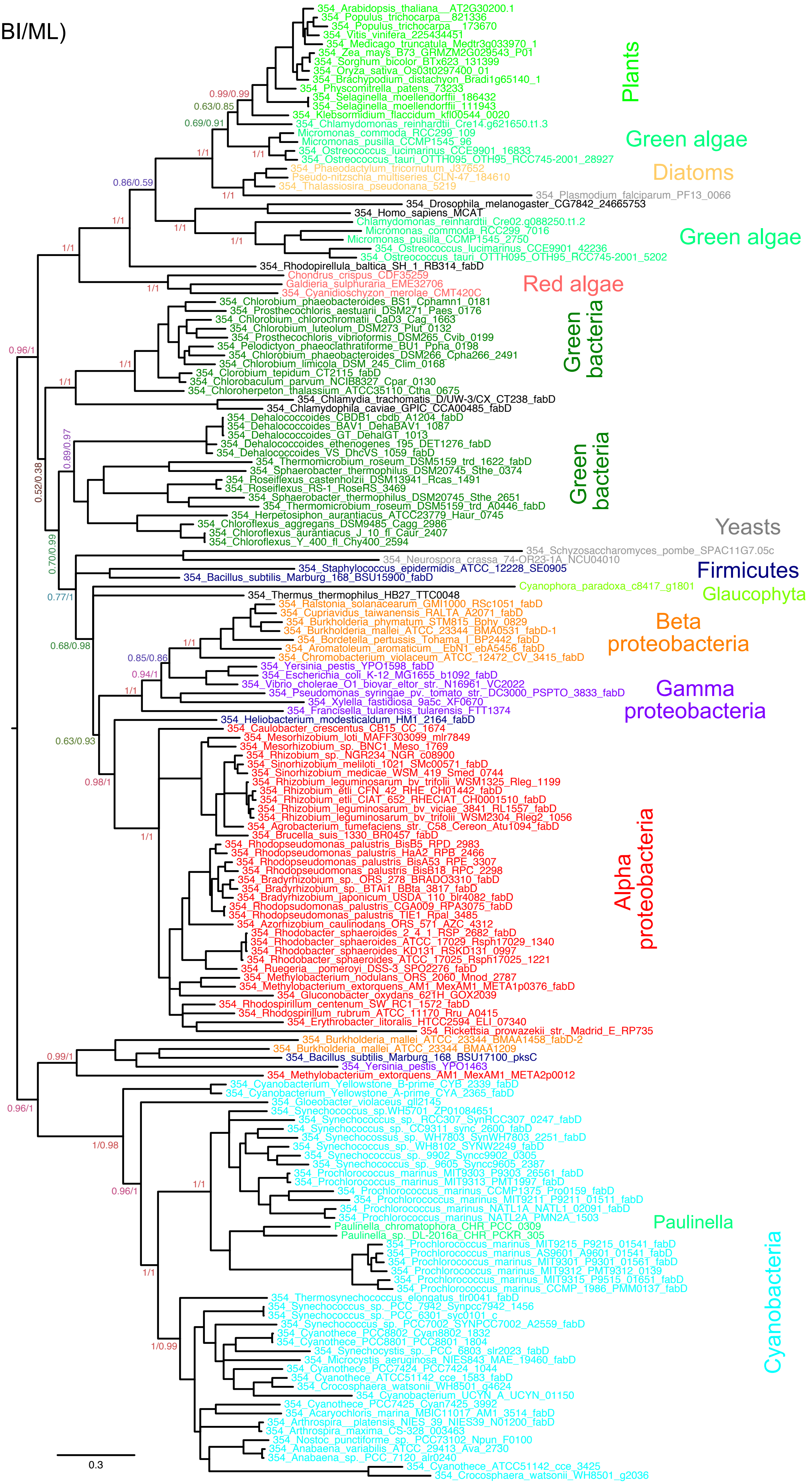
354_Arabidopsis_thaliana__AT2G30200.1
354_Populus_trichocarpa__821336
354_Populus_trichocarpa__173670
354_Vitis_vinifera_225434451
354_Medicago_truncatula_Medtr3g033970_1
354_Zea_mays_B73_GRMZM2G029543_P01
354_Sorghum_bicolor_BTx623_131399
354_Oryza_sativa_Os03t0297400_01
354_Brachypodium_distachyon_Bradi1g65140_1
354_Physcomitrella_patens_73233
354_Selaginella_moellendorffii_186432
354_Selaginella_moellendorffii_111943
354_Klebsormidium_flaccidum_kfl00544_0020
354_Chlamydomonas_reinhardtii_Cre14.g621650.t1.3
Micromonas_commoda_RCC299_109
Micromonas_pusilla_CCMP1545_96
354_Ostreococcus_lucimarinus_CCE9901_16833
354_Ostreococcus_tauri_OTTH095_OTH95_RCC745-2001_28927
354_Phaeodactylum_tricornutum_J37652
Pseudo-nitzschia_multiseries_CLN-47_184610
354_Thalassiosira_pseudonana_5219
354_Plasmodium_falciparum_PF13_0066
354_Drosophila_melanogaster_CG7842_24665753
354_Homo_sapiens_MCAT
Chlamydomonas_reinhardtii_Cre02.g088250.t1.2
Micromonas_commoda_RCC299_7016
Micromonas_pusilla_CCMP1545_2750
354_Ostreococcus_lucimarinus_CCE9901_42236
354_Ostreococcus_tauri_OTTH095_OTH95_RCC745-2001_5202
354_Rhodopirellula_baltica_SH_1_RB314_fabD
Chondrus_crispus_CDF35259
Galdieria_sulphuraria_EME32706
354_Cyanidioschyzon_merolae_CMT420C
354_Chlorobium_phaeobacteroides_BS1_Cphamn1_0181
354_Prosthecochloris_aestuarii_DSM271_Paes_0176
354_Chlorobium_chlorochromatii_CaD3_Cag_1663
354_Chlorobium_luteolum_DSM273_Plut_0132
354_Prosthecochloris_vibrioformis_DSM265_Cvib_0199
354_Pelodictyon_phaeoclathratiforme_BU1_Ppha_0198
354_Chlorobium_phaeobacteroides_DSM266_Cpha266_2491
354_Chlorobium_limicola_DSM_245_Clim_0168
354_Clorobium_tepidum_CT2115_fabD
354_Chlorobaculum_parvum_NCIB8327_Cpar_0130
354_Chloroherpeton_thalassium_ATCC35110_Ctha_0675
354_Chlamydia_trachomatis_D/UW-3/CX_CT238_fabD
354_Chlamydophila_caviae_GPIC_CCA00485_fabD
354_Dehalococcoides_CBDB1_cbdb_A1204_fabD
354_Dehalococcoides_BAV1_DehaBAV1_1087
354_Dehalococcoides_GT_DehalGT_1013
354_Dehalococcoides_ethenogenes_195_DET1276_fabD
354_Dehalococcoides_VS_DhcVS_1059_fabD
354_Thermomicrobium_roseum_DSM5159_trd_1622_fabD
354_Sphaerobacter_thermophilus_DSM20745_Sthe_0374
354_Roseiflexus_castenholzii_DSM13941_Rcas_1491
354_Roseiflexus_RS-1_RoseRS_3469
354_Sphaerobacter_thermophilus_DSM20745_Sthe_2651
354_Thermomicrobium_roseum_DSM5159_trd_A0446_fabD
354_Herpetosiphon_aurantiacus_ATCC23779_Haur_0745
354_Chloroflexus_aggregans_DSM9485_Cagg_2986
354_Chloroflexus_aurantiacus_J_10_fl_Caur_2407
354_Chloroflexus_Y_400_fl_Chy400_2594
354_Schyzosaccharomyces_pombe_SPAC11G7.05c
354_Neurospora_crassa_74-OR23-1A_NCU04010
354_Staphylococcus_epidermidis_ATCC_12228_SE0905
354_Bacillus_subtilis_Marburg_168_BSU15900_fabD
Cyanophora_paradoxa_c8417_g1801
354_Thermus_thermophilus_HB27_TTC0048
354_Ralstonia_solanacearum_GMI1000_RSc1051_fabD
354_Cupriavidus_taiwanensis_RALTA_A2071_fabD
354_Burkholderia_phymatum_STM815_Bphy_0829
354_Burkholderia_mallei_ATCC_23344_BMA0531_fabD-1
354_Bordetella_pertussis_Tohama_I_BP2442_fabD
354_Aromatoleum_aromaticum__EbN1_ebA5456_fabD
354_Chromobacterium_violaceum_ATCC_12472_CV_3415_fabD
354_Yersinia_pestis_YPO1598_fabD
354_Escherichia_coli_K-12_MG1655_b1092_fabD
354_Vibrio_cholerae_O1_biovar_eltor_str._N16961_VC2022
354_Pseudomonas_syringae_pv._tomato_str._DC3000_PSPTO_3833_fabD
354_Xylella_fastidiosa_9a5c_XF0670
354_Francisella_tularensis_tularensis_FTT1374
354_Heliobacterium_modesticaldum_HM1_2164_fabD
354_Caulobacter_crescentus_CB15_CC_1674
354_Mesorhizobium_loti_MAFF303099_mlr7849
354_Mesorhizobium_sp._BNC1_Meso_1769
354_Rhizobium_sp._NGR234_NGR_c08900
354_Sinorhizobium_meliloti_1021_SMc00571_fabD
354_Sinorhizobium_medicae_WSM_419_Smed_0744
354_Rhizobium_leguminosarum_bv_trifolii_WSM1325_Rleg_1199
354_Rhizobium_etli_CFN_42_RHE_CH01442_fabD
354_Rhizobium_etli_CIAT_652_RHECIAT_CH0001510_fabD
354_Rhizobium_leguminosarum_bv_viciae_3841_RL1557_fabD
354_Rhizobium_leguminosarum_bv_trifolii_WSM2304_Rleg2_1056
354_Agrobacterium_tumefaciens_str._C58_Cereon_Atu1094_fabD
354_Brucella_suis_1330_BR0457_fabD
354_Rhodopseudomonas_palustris_BisB5_RPD_2983
354_Rhodopseudomonas_palustris_HaA2_RPB_2466
354_Rhodopseudomonas_palustris_BisA53_RPE_3307
354_Rhodopseudomonas_palustris_BisB18_RPC_2298
354_Bradyrhizobium_sp._ORS_278_BRADO3310_fabD
354_Bradyrhizobium_sp._BTAi1_BBta_3817_fabD
354_Bradyrhizobium_japonicum_USDA_110_blr4082_fabD
354_Rhodopseudomonas_palustris_CGA009_RPA3075_fabD
354_Rhodopseudomonas_palustris_TIE1_Rpal_3485
354_Azorhizobium_caulinodans_ORS_571_AZC_4312
354_Rhodobacter_sphaeroides_2_4_1_RSP_2682_fabD
354_Rhodobacter_sphaeroides_ATCC_17029_Rsph17029_1340
354_Rhodobacter_sphaeroides_KD131_RSKD131_0997
354_Rhodobacter_sphaeroides_ATCC_17025_Rsph17025_1221
354_Ruegeria__pomeroyi_DSS-3_SPO2276_fabD
354_Methylobacterium_nodulans_ORS_2060_Mnod_2787
354_Methylobacterium_extorquens_AM1_MexAM1_META1p0376_fabD
354_Gluconobacter_oxydans_621H_GOX2039
354_Rhodospirillum_centenum_SW_RC1_1572_fabD
354_Rhodospirillum_rubrum_ATCC_11170_Rru_A0415
354_Erythrobacter_litoralis_HTCC2594_ELI_07340
354_Rickettsia_prowazekii_str._Madrid_E_RP735
354_Burkholderia_mallei_ATCC_23344_BMAA1458_fabD-2
354_Burkholderia_mallei_ATCC_23344_BMAA1209
354_Bacillus_subtilis_Marburg_168_BSU17100_pksC
354_Yersinia_pestis_YPO1463
354_Methylobacterium_extorquens_AM1_MexAM1_META2p0012
354_Cyanobacterium_Yellowstone_B-prime_CYB_2339_fabD
354_Cyanobacterium_Yellowstone_A-prime_CYA_2365_fabD
354_Gloeobacter_violaceus_gll2145
354_Synechococcus_sp.WH5701_ZP01084651
354_Synechococcus_sp._RCC307_SynRCC307_0247_fabD
354_Synechococcus_sp._CC9311_sync_2600_fabD
354_Synechococcus_sp._WH7803_SynWH7803_2251_fabD
354_Synechococcus_sp._WH8102_SYNW2249_fabD
354_Synechococcus_sp._9902_Syncc9902_0305
354_Synechococcus_sp._9605_Syncc9605_2387
354_Prochlorococcus_marinus_MIT9303_P9303_26561_fabD
354_Prochlorococcus_marinus_MIT9313_PMT1997_fabD
354_Prochlorococcus_marinus_CCMP1375_Pro0159_fabD
354_Prochlorococcus_marinus_MIT9211_P9211_01511_fabD
354_Prochlorococcus_marinus_NATL1A_NATL1_02091_fabD
354_Prochlorococcus_marinus_NATL2A_PMN2A_1503
Paulinella_chromatophora_CHR_PCC_0309
Paulinella_sp._DL-2016a_CHR_PCKR_305
354_Prochlorococcus_marinus_MIT9215_P9215_01541_fabD
354_Prochlorococcus_marinus_AS9601_A9601_01541_fabD
354_Prochlorococcus_marinus_MIT9301_P9301_01561_fabD
354_Prochlorococcus_marinus_MIT9312_PMT9312_0139
354_Prochlorococcus_marinus_MIT9315_P9515_01651_fabD
354_Prochlorococcus_marinus_CCMP_1986_PMM0137_fabD
354_Thermosynechococcus_elongatus_tlr0041_fabD
354_Synechococcus_sp._PCC_7942_Synpcc7942_1456
354_Synechococcus_sp._PCC_6301_syc0101_c
354_Synechococcus_sp._PCC7002_SYNPCC7002_A2559_fabD
354_Cyanothece_PCC8802_Cyan8802_1832
354_Cyanothece_PCC8801_PCC8801_1804
354_Synechocystis_sp._PCC_6803_slr2023_fabD
354_Microcystis_aeruginosa_NIES843_MAE_19460_fabD
354_Cyanothece_PCC7424_PCC7424_1044
354_Cyanothece_ATCC51142_cce_1583_fabD
354_Crocosphaera_watsonii_WH8501_g4624
354_Cyanobacterium_UCYN_A_UCYN_01150
354_Cyanothece_PCC7425_Cyan7425_3992
354_Acaryochloris_marina_MBIC11017_AM1_3514_fabD
354_Arthrospira__platensis_NIES_39_NIES39_N01200_fabD
354_Arthrospira_maxima_CS-328_003463
354_Nostoc_punctiforme_sp._PCC73102_Npun_F0100
354_Anabaena_variabilis_ATCC_29413_Ava_2730
354_Anabaena_sp._PCC_7120_alr0240
354_Cyanothece_ATCC51142_cce_3425
354_Crocosphaera_watsonii_WH8501_g2036
Plants
Green algae
Diatoms
Green algae
Red algae
Green bacteria
Green bacteria
Yeasts
Firmicutes
Glaucophyta
Beta proteobacteria
Gamma proteobacteria
Alpha proteobacteria
Paulinella
Cyanobacteria
0.99/0.99
0.63/0.85
0.69/0.91
1/1
1/1
0.86/0.59
1/1
1/1
1/1
1/1
1/1
0.96/1
1/1
1/1
0.89/0.97
0.52/0.38
0.70/0.99
0.77/1
0.68/0.98
1/1
0.85/0.86
0.94/1
1/1
0.63/0.93
0.98/1
1/1
0.99/1
0.96/1
1/0.98
0.96/1
1/1
1/1
1/0.99
0.3

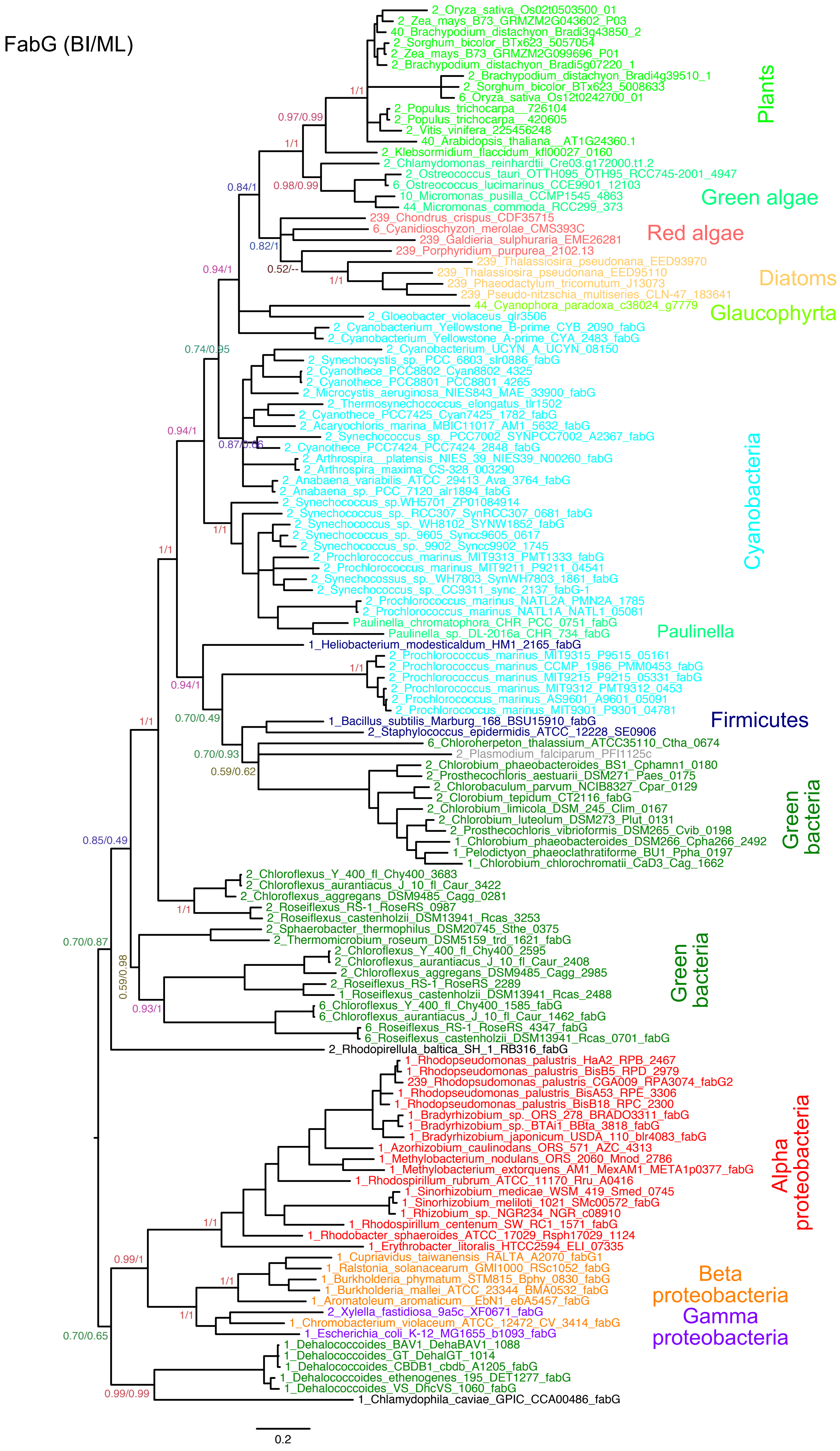

FabG (BI/ML)
2_Oryza_sativa_Os02t0503500_01
2_Zea_mays_B73_GRMZM2G043602_P03
40_Brachypodium_distachyon_Bradi3g43850_2
2_Sorghum_bicolor_BTx623_5057054
2_Zea_mays_B73_GRMZM2G099696_P01
2_Brachypodium_distachyon_Bradi5g07220_1
2_Brachypodium_distachyon_Bradi4g39510_1
2_Sorghum_bicolor_BTx623_5008633
6_Oryza_sativa_Os12t0242700_01
2_Populus_trichocarpa__726104
2_Populus_trichocarpa__420605
2_Vitis_vinifera_225456248
40_Arabidopsis_thaliana__AT1G24360.1
2_Klebsormidium_flaccidum_kfl00027_0160
2_Chlamydomonas_reinhardtii_Cre03.g172000.t1.2
2_Ostreococcus_tauri_OTTH095_OTH95_RCC745-2001_4947
6_Ostreococcus_lucimarinus_CCE9901_12103
10_Micromonas_pusilla_CCMP1545_4863
44_Micromonas_commoda_RCC299_373
239_Chondrus_crispus_CDF35715
6_Cyanidioschyzon_merolae_CMS393C
239_Galdieria_sulphuraria_EME26281
239_Porphyridium_purpurea_2102.13
239_Thalassiosira_pseudonana_EED93970
239_Thalassiosira_pseudonana_EED95110
239_Phaeodactylum_tricornutum_J13073
239_Pseudo-nitzschia_multiseries_CLN-47_183641
44_Cyanophora_paradoxa_c38024_g7779
2_Gloeobacter_violaceus_glr3506
2_Cyanobacterium_Yellowstone_B-prime_CYB_2090_fabG
2_Cyanobacterium_Yellowstone_A-prime_CYA_2483_fabG
2_Cyanobacterium_UCYN_A_UCYN_08150
2_Synechocystis_sp._PCC_6803_slr0886_fabG
2_Cyanothece_PCC8802_Cyan8802_4325
2_Cyanothece_PCC8801_PCC8801_4265
2_Microcystis_aeruginosa_NIES843_MAE_33900_fabG
2_Thermosynechococcus_elongatus_tlr1502
2_Cyanothece_PCC7425_Cyan7425_1782_fabG
2_Acaryochloris_marina_MBIC11017_AM1_5632_fabG
2_Synechococcus_sp._PCC7002_SYNPCC7002_A2367_fabG
2_Cyanothece_PCC7424_PCC7424_2848_fabG
2_Arthrospira__platensis_NIES_39_NIES39_N00260_fabG
2_Arthrospira_maxima_CS-328_003290
2_Anabaena_variabilis_ATCC_29413_Ava_3764_fabG
2_Anabaena_sp._PCC_7120_alr1894_fabG
2_Synechococcus_sp.WH5701_ZP01084914
2_Synechococcus_sp._RCC307_SynRCC307_0681_fabG
2_Synechococcus_sp._WH8102_SYNW1852_fabG
2_Synechococcus_sp._9605_Syncc9605_0617
2_Synechococcus_sp._9902_Syncc9902_1745
2_Prochlorococcus_marinus_MIT9313_PMT1333_fabG
2_Prochlorococcus_marinus_MIT9211_P9211_04541
2_Synechocossus_sp._WH7803_SynWH7803_1861_fabG
2_Synechococcus_sp._CC9311_sync_2137_fabG-1
2_Prochlorococcus_marinus_NATL2A_PMN2A_1785
2_Prochlorococcus_marinus_NATL1A_NATL1_05081
Paulinella_chromatophora_CHR_PCC_0751_fabG
Paulinella_sp._DL-2016a_CHR_734_fabG
1_Heliobacterium_modesticaldum_HM1_2165_fabG
2_Prochlorococcus_marinus_MIT9315_P9515_05161
2_Prochlorococcus_marinus_CCMP_1986_PMM0453_fabG
2_Prochlorococcus_marinus_MIT9215_P9215_05331_fabG
2_Prochlorococcus_marinus_MIT9312_PMT9312_0453
2_Prochlorococcus_marinus_AS9601_A9601_05091
2_Prochlorococcus_marinus_MIT9301_P9301_04781
1_Bacillus_subtilis_Marburg_168_BSU15910_fabG
2_Staphylococcus_epidermidis_ATCC_12228_SE0906
6_Chloroherpeton_thalassium_ATCC35110_Ctha_0674
2_Plasmodium_falciparum_PFI1125c
2_Chlorobium_phaeobacteroides_BS1_Cphamn1_0180
2_Prosthecochloris_aestuarii_DSM271_Paes_0175
2_Chlorobaculum_parvum_NCIB8327_Cpar_0129
2_Clorobium_tepidum_CT2116_fabG
2_Chlorobium_limicola_DSM_245_Clim_0167
2_Chlorobium_luteolum_DSM273_Plut_0131
2_Prosthecochloris_vibrioformis_DSM265_Cvib_0198
1_Chlorobium_phaeobacteroides_DSM266_Cpha266_2492
1_Pelodictyon_phaeoclathratiforme_BU1_Ppha_0197
1_Chlorobium_chlorochromatii_CaD3_Cag_1662
2_Chloroflexus_Y_400_fl_Chy400_3683
2_Chloroflexus_aurantiacus_J_10_fl_Caur_3422
2_Chloroflexus_aggregans_DSM9485_Cagg_0281
2_Roseiflexus_RS-1_RoseRS_0987
2_Roseiflexus_castenholzii_DSM13941_Rcas_3253
2_Sphaerobacter_thermophilus_DSM20745_Sthe_0375
2_Thermomicrobium_roseum_DSM5159_trd_1621_fabG
2_Chloroflexus_Y_400_fl_Chy400_2595
2_Chloroflexus_aurantiacus_J_10_fl_Caur_2408
2_Chloroflexus_aggregans_DSM9485_Cagg_2985
2_Roseiflexus_RS-1_RoseRS_2289
1_Roseiflexus_castenholzii_DSM13941_Rcas_2488
6_Chloroflexus_Y_400_fl_Chy400_1585_fabG
6_Chloroflexus_aurantiacus_J_10_fl_Caur_1462_fabG
6_Roseiflexus_RS-1_RoseRS_4347_fabG
6_Roseiflexus_castenholzii_DSM13941_Rcas_0701_fabG
2_Rhodopirellula_baltica_SH_1_RB316_fabG
1_Rhodopseudomonas_palustris_HaA2_RPB_2467
1_Rhodopseudomonas_palustris_BisB5_RPD_2979
239_Rhodopsudomonas_palustris_CGA009_RPA3074_fabG2
1_Rhodopseudomonas_palustris_BisA53_RPE_3306
1_Rhodopseudomonas_palustris_BisB18_RPC_2300
1_Bradyrhizobium_sp._ORS_278_BRADO3311_fabG
1_Bradyrhizobium_sp._BTAi1_BBta_3818_fabG
1_Bradyrhizobium_japonicum_USDA_110_blr4083_fabG
1_Azorhizobium_caulinodans_ORS_571_AZC_4313
1_Methylobacterium_nodulans_ORS_2060_Mnod_2786
1_Methylobacterium_extorquens_AM1_MexAM1_META1p0377_fabG
1_Rhodospirillum_rubrum_ATCC_11170_Rru_A0416
1_Sinorhizobium_medicae_WSM_419_Smed_0745
1_Sinorhizobium_meliloti_1021_SMc00572_fabG
1_Rhizobium_sp._NGR234_NGR_c08910
1_Rhodospirillum_centenum_SW_RC1_1571_fabG
1_Rhodobacter_sphaeroides_ATCC_17029_Rsph17029_1124
1_Erythrobacter_litoralis_HTCC2594_ELI_07335
1_Cupriavidus_taiwanensis_RALTA_A2070_fabG1
1_Ralstonia_solanacearum_GMI1000_RSc1052_fabG
1_Burkholderia_phymatum_STM815_Bphy_0830_fabG
1_Burkholderia_mallei_ATCC_23344_BMA0532_fabG
1_Aromatoleum_aromaticum__EbN1_ebA5457_fabG
2_Xylella_fastidiosa_9a5c_XF0671_fabG
1_Chromobacterium_violaceum_ATCC_12472_CV_3414_fabG
1_Escherichia_coli_K-12_MG1655_b1093_fabG
1_Dehalococcoides_BAV1_DehaBAV1_1088
1_Dehalococcoides_GT_DehalGT_1014
1_Dehalococcoides_CBDB1_cbdb_A1205_fabG
1_Dehalococcoides_ethenogenes_195_DET1277_fabG
1_Dehalococcoides_VS_DhcVS_1060_fabG
1_Chlamydophila_caviae_GPIC_CCA00486_fabG
Plants
Green algae
Red algae
Diatoms
Glaucophyrta
Cyanobacteria
Paulinella
Firmicutes
Green bacteria
Green bacteria
Alpha proteobacteria
Beta proteobacteria
Gamma proteobacteria
0.2

FabI (BI/ML)

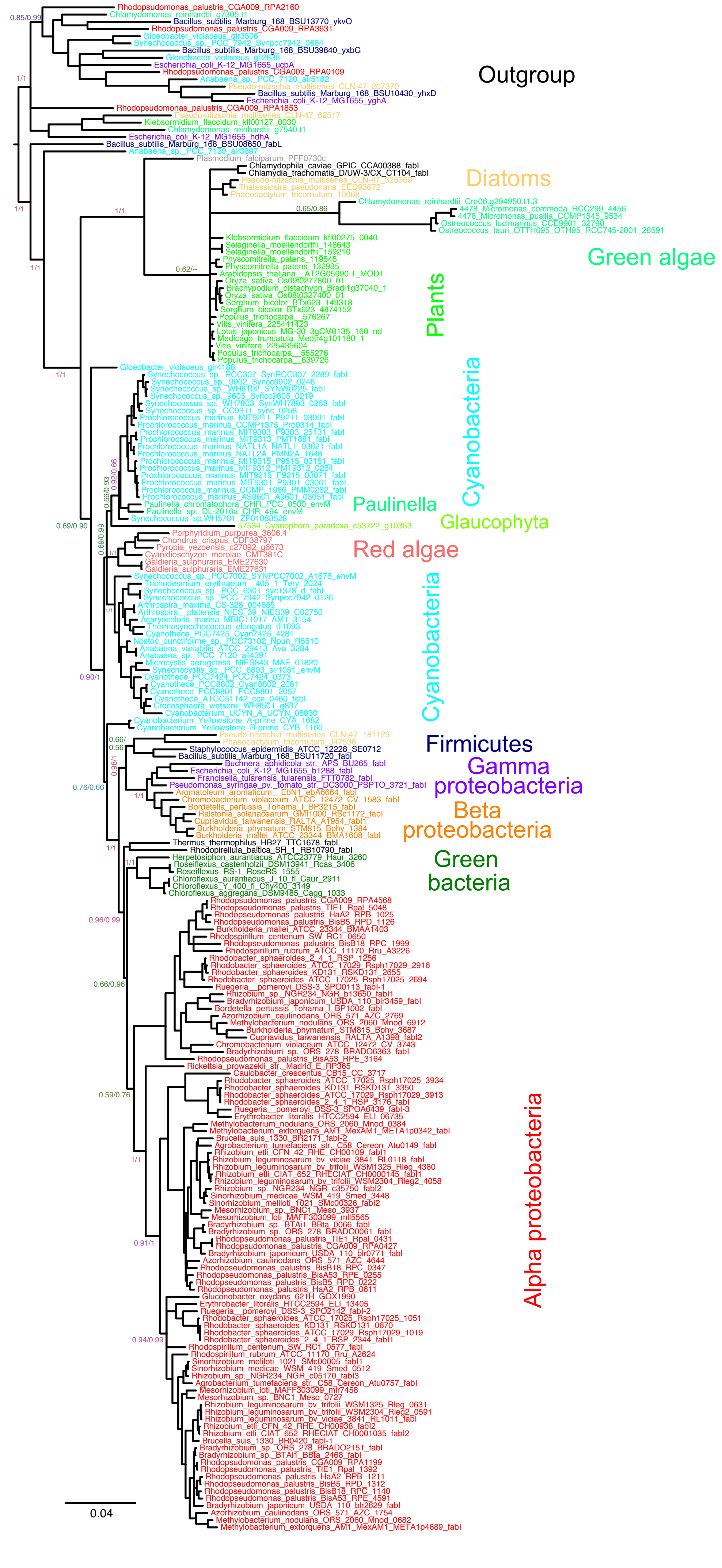

Outgroup
Diatoms
Green algae
Plants
Cyanobacteria
Paulinella
Glaucophyta
Red algae
Cyanobacteria
Firmicutes
Gamma proteobacteria
Beta proteobacteria
Green bacteria
Alpha proteobacteria
0.04

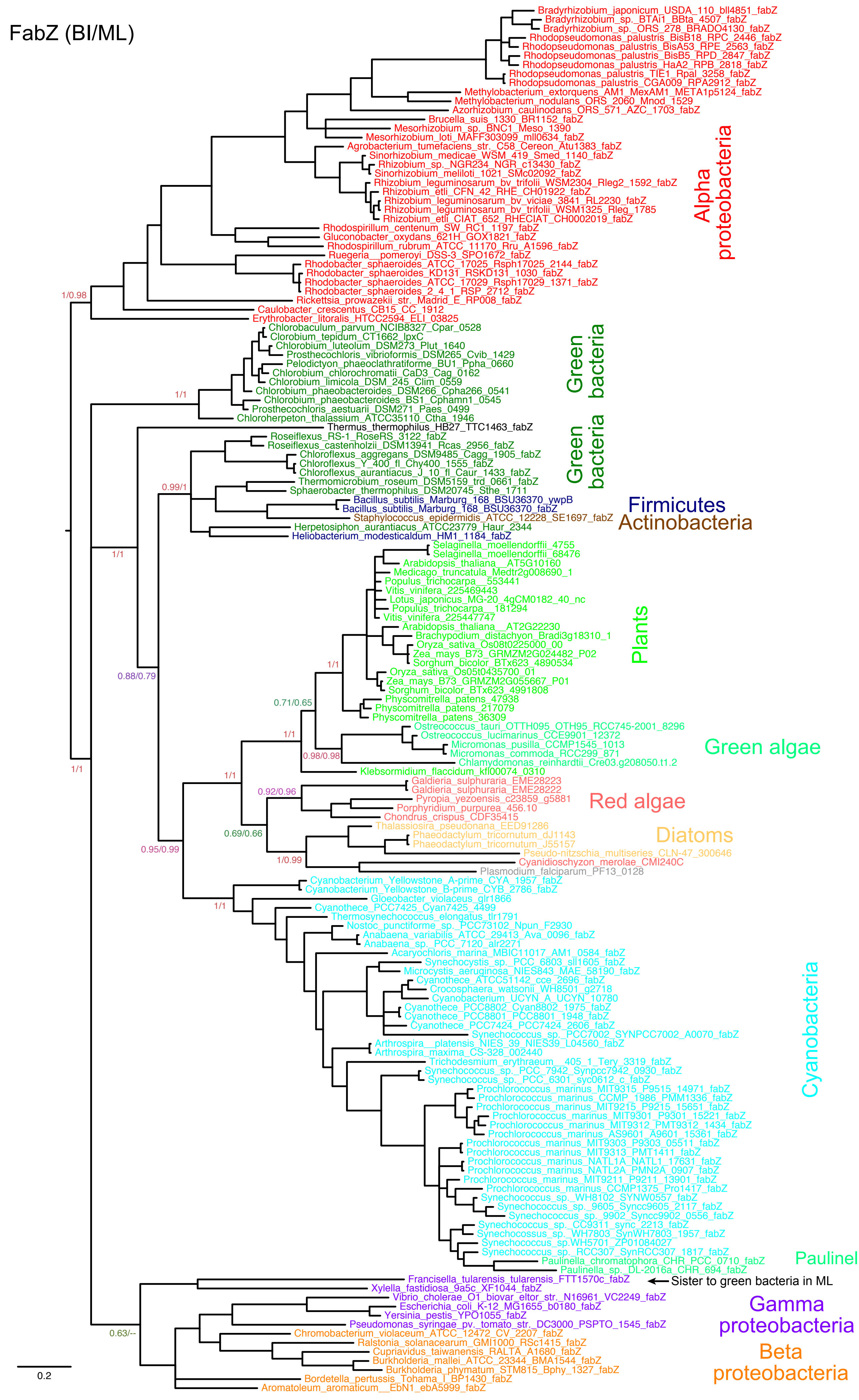
FabZ (BI/ML)
Bradyrhizobium_japonicum_USDA_110_bll4851_fabZ
Bradyrhizobium_sp._BTAi1_BBta_4507_fabZ
Bradyrhizobium_sp._ORS_278_BRADO4130_fabZ
Rhodopseudomonas_palustris_BisB18_RPC_2446_fabZ
Rhodopseudomonas_palustris_BisA53_RPE_2563_fabZ
Rhodopseudomonas_palustris_BisB5_RPD_2847_fabZ
Rhodopseudomonas_palustris_HaA2_RPB_2818_fabZ
Rhodopseudomonas_palustris_TIE1_Rpal_3258_fabZ
Rhodopsudomonas_palustris_CGA009_RPA2912_fabZ
Methylobacterium_extorquens_AM1_MexAM1_META1p5124_fabZ
Methylobacterium_nodulans_ORS_2060_Mnod_1529
Azorhizobium_caulinodans_ORS_571_AZC_1703_fabZ
Brucella_suis_1330_BR1152_fabZ
Mesorhizobium_sp._BNC1_Meso_1390
Mesorhizobium_loti_MAFF303099_mll0634_fabZ
Agrobacterium_tumefaciens_str._C58_Cereon_Atu1383_fabZ
Sinorhizobium_medicae_WSM_419_Smed_1140_fabZ
Rhizobium_sp._NGR234_NGR_c13430_fabZ
Sinorhizobium_meliloti_1021_SMc02092_fabZ
Rhizobium_leguminosarum_bv_trifolii_WSM2304_Rleg2_1592_fabZ
Rhizobium_etli_CFN_42_RHE_CH01922_fabZ
Rhizobium_leguminosarum_bv_viciae_3841_RL2230_fabZ
Rhizobium_leguminosarum_bv_trifolii_WSM1325_Rleg_1785
Rhizobium_etli_CIAT_652_RHECIAT_CH0002019_fabZ
Rhodospirillum_centenum_SW_RC1_1197_fabZ
Gluconobacter_oxydans_621H_GOX1821_fabZ
Rhodospirillum_rubrum_ATCC_11170_Rru_A1596_fabZ
Ruegeria__pomeroyi_DSS-3_SPO1672_fabZ
Rhodobacter_sphaeroides_ATCC_17025_Rsph17025_2144_fabZ
Rhodobacter_sphaeroides_KD131_RSKD131_1030_fabZ
Rhodobacter_sphaeroides_ATCC_17029_Rsph17029_1371_fabZ
Rhodobacter_sphaeroides_2_4_1_RSP_2712_fabZ
Rickettsia_prowazekii_str._Madrid_E_RP008_fabZ
Caulobacter_crescentus_CB15_CC_1912
Erythrobacter_litoralis_HTCC2594_ELI_03825
Alpha proteobacteria
1/0.98
Chlorobaculum_parvum_NCIB8327_Cpar_0528
Clorobium_tepidum_CT1662_lpxC
Chlorobium_luteolum_DSM273_Plut_1640
Prosthecochloris_vibrioformis_DSM265_Cvib_1429
Pelodictyon_phaeoclathratiforme_BU1_Ppha_0660
Chlorobium_chlorochromatii_CaD3_Cag_0162
Chlorobium_limicola_DSM_245_Clim_0559
Chlorobium_phaeobacteroides_DSM266_Cpha266_0541
Chlorobium_phaeobacteroides_BS1_Cphamn1_0545
Prosthecochloris_aestuarii_DSM271_Paes_0499
Chloroherpeton_thalassium_ATCC35110_Ctha_1946
Green bacteria
1/1
Thermus_thermophilus_HB27_TTC1463_fabZ
Roseiflexus_RS-1_RoseRS_3122_fabZ
Roseiflexus_castenholzii_DSM13941_Rcas_2956_fabZ
Chloroflexus_aggregans_DSM9485_Cagg_1905_fabZ
Chloroflexus_Y_400_fl_Chy400_1555_fabZ
Chloroflexus_aurantiacus_J_10_fl_Caur_1433_fabZ
Thermomicrobium_roseum_DSM5159_trd_0661_fabZ
Sphaerobacter_thermophilus_DSM20745_Sthe_1711
Green bacteria
0.99/1
Bacillus_subtilis_Marburg_168_BSU36370_ywpB
Bacillus_subtilis_Marburg_168_BSU36370_fabZ
Staphylococcus_epidermidis_ATCC_12228_SE1697_fabZ
Firmicutes
Actinobacteria
Herpetosiphon_aurantiacus_ATCC23779_Haur_2344
Heliobacterium_modesticaldum_HM1_1184_fabZ
1/1
Selaginella_moellendorffii_4755
Selaginella_moellendorffii_68476
Arabidopsis_thaliana__AT5G10160
Medicago_truncatula_Medtr2g008690_1
Populus_trichocarpa__553441
Vitis_vinifera_225469443
Lotus_japonicus_MG-20_4gCM0182_40_nc
Populus_trichocarpa__181294
Vitis_vinifera_225447747
Arabidopsis_thaliana__AT2G22230
Brachypodium_distachyon_Bradi3g18310_1
Oryza_sativa_Os08t0225000_00
Zea_mays_B73_GRMZM2G024482_P02
Sorghum_bicolor_BTx623_4890534
Plants
Oryza_sativa_Os05t0435700_01
Zea_mays_B73_GRMZM2G055667_P01
Sorghum_bicolor_BTx623_4991808
1/1
Physcomitrella_patens_47938
Physcomitrella_patens_217079
Physcomitrella_patens_36309
0.88/0.79
0.71/0.65
Ostreococcus_tauri_OTTH095_OTH95_RCC745-2001_8296
Ostreococcus_lucimarinus_CCE9901_12372
Micromonas_pusilla_CCMP1545_1013
Micromonas_commoda_RCC299_871
Chlamydomonas_reinhardtii_Cre03.g208050.t1.2
Green algae
1/1
0.98/0.98
Klebsormidium_flaccidum_kfl00074_0310
1/1
Galdieria_sulphuraria_EME28223
Galdieria_sulphuraria_EME28222
0.92/0.96
Pyropia_yezoensis_c23859_g5881
Porphyridium_purpurea_456.10
Chondrus_crispus_CDF35415
Red algae
Thalassiosira_pseudonana_EED91286
0.69/0.66
Phaeodactylum_tricornutum_dJ1143
Phaeodactylum_tricornutum_J55157
Diatoms
0.95/0.99
Pseudo-nitzschia_multiseries_CLN-47_300646
1/0.99
Cyanidioschyzon_merolae_CMI240C
Plasmodium_falciparum_PF13_0128
Cyanobacterium_Yellowstone_A-prime_CYA_1957_fabZ
Cyanobacterium_Yellowstone_B-prime_CYB_2786_fabZ
Gloeobacter_violaceus_glr1866
1/1
1/1
Cyanothece_PCC7425_Cyan7425_4499
Thermosynechococcus_elongatus_tlr1791
Nostoc_punctiforme_sp._PCC73102_Npun_F2930
Anabaena_variabilis_ATCC_29413_Ava_0096_fabZ
Anabaena_sp._PCC_7120_alr2271
Acaryochloris_marina_MBIC11017_AM1_0584_fabZ
Synechocystis_sp._PCC_6803_sll1605_fabZ
Microcystis_aeruginosa_NIES843_MAE_58190_fabZ
Cyanothece_ATCC51142_cce_2696_fabZ
Crocosphaera_watsonii_WH8501_g2718
Cyanobacterium_UCYN_A_UCYN_10780
Cyanothece_PCC8802_Cyan8802_1975_fabZ
Cyanothece_PCC8801_PCC8801_1948_fabZ
Cyanothece_PCC7424_PCC7424_2606_fabZ
Synechococcus_sp._PCC7002_SYNPCC7002_A0070_fabZ
Arthrospira__platensis_NIES_39_NIES39_L04560_fabZ
Arthrospira_maxima_CS-328_002440
Trichodesmium_erythraeum__405_1_Tery_3319_fabZ
Synechococcus_sp._PCC_7942_Synpcc7942_0930_fabZ
Synechococcus_sp._PCC_6301_syc0612_c_fabZ
Prochlorococcus_marinus_MIT9315_P9515_14971_fabZ
Prochlorococcus_marinus_CCMP_1986_PMM1336_fabZ
Prochlorococcus_marinus_MIT9215_P9215_15651_fabZ
Prochlorococcus_marinus_MIT9301_P9301_15221_fabZ
Prochlorococcus_marinus_MIT9312_PMT9312_1434_fabZ
Prochlorococcus_marinus_AS9601_A9601_15361_fabZ
Prochlorococcus_marinus_MIT9303_P9303_05511_fabZ
Prochlorococcus_marinus_MIT9313_PMT1411_fabZ
Prochlorococcus_marinus_NATL1A_NATL1_17631_fabZ
Prochlorococcus_marinus_NATL2A_PMN2A_0907_fabZ
Prochlorococcus_marinus_MIT9211_P9211_13901_fabZ
Prochlorococcus_marinus_CCMP1375_Pro1417_fabZ
Synechococcus_sp._WH8102_SYNW0557_fabZ
Synechococcus_sp._9605_Syncc9605_2117_fabZ
Synechococcus_sp._9902_Syncc9902_0556_fabZ
Synechococcus_sp._CC9311_sync_2213_fabZ
Synechocossus_sp._WH7803_SynWH7803_1957_fabZ
Synechococcus_sp.WH5701_ZP01084027
Synechococcus_sp._RCC307_SynRCC307_1817_fabZ
Cyanobacteria
Paulinella_chromatophora_CHR_PCC_0710_fabZ
Paulinella_sp._DL-2016a_CHR_694_fabZ
Paulinella
1/1
Francisella_tularensis_tularensis_FTT1570c_fabZ
Sister to green bacteria in ML
Xylella_fastidiosa_9a5c_XF1044_fabZ
Vibrio_cholerae_O1_biovar_eltor_str._N16961_VC2249_fabZ
Escherichia_coli_K-12_MG1655_b0180_fabZ
Yersinia_pestis_YPO1055_fabZ
Pseudomonas_syringae_pv._tomato_str._DC3000_PSPTO_1545_fabZ
Gamma proteobacteria
0.63/--
Chromobacterium_violaceum_ATCC_12472_CV_2207_fabZ
Ralstonia_solanacearum_GMI1000_RSc1415_fabZ
Cupriavidus_taiwanensis_RALTA_A1680_fabZ
Burkholderia_mallei_ATCC_23344_BMA1544_fabZ
Burkholderia_phymatum_STM815_Bphy_1327_fabZ
Bordetella_pertussis_Tohama_I_BP1430_fabZ
Aromatoleum_aromaticum__EbN1_ebA5999_fabZ
Beta proteobacteria
0.2

KAS III / FabH (BI/ML)

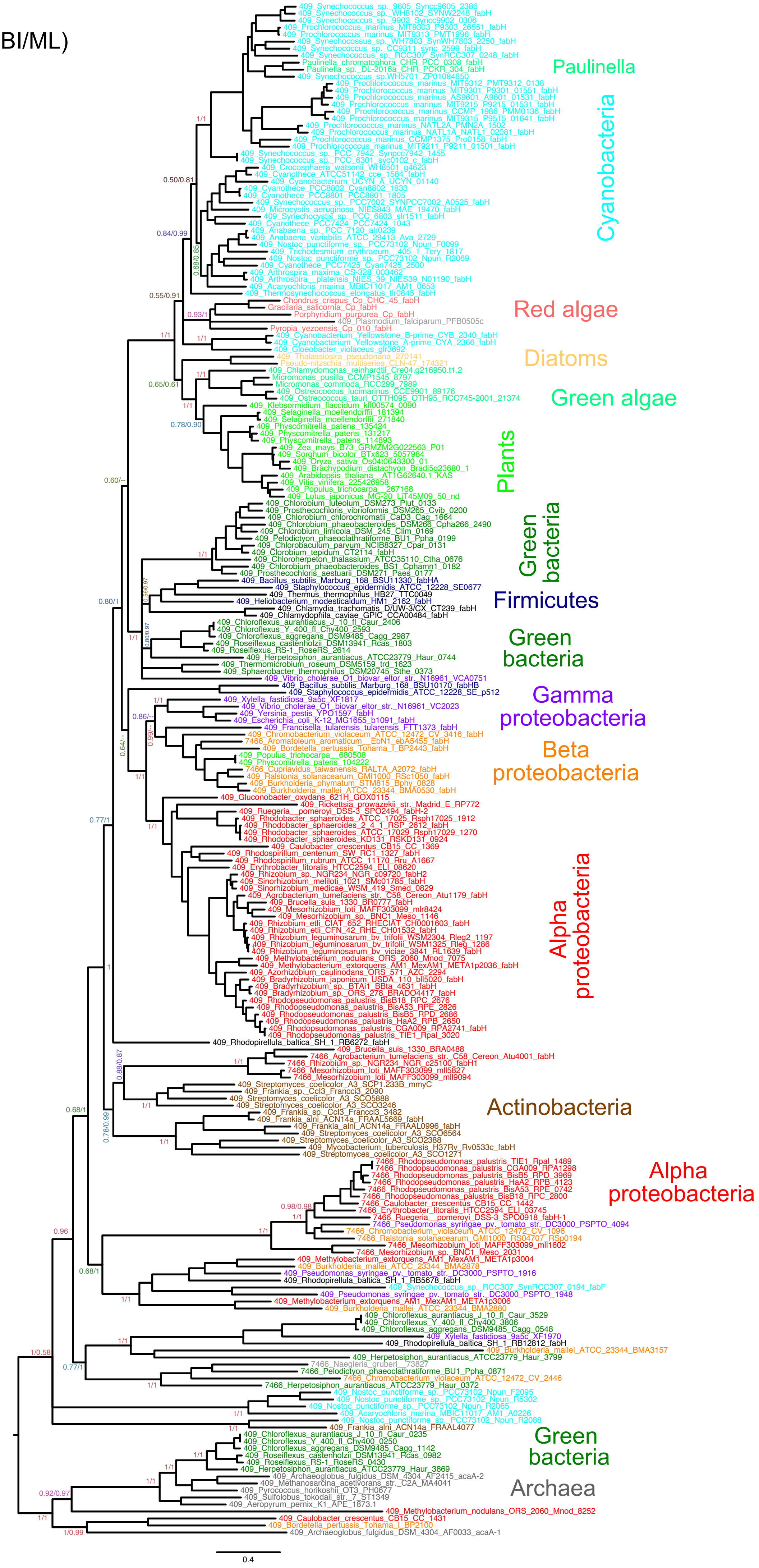

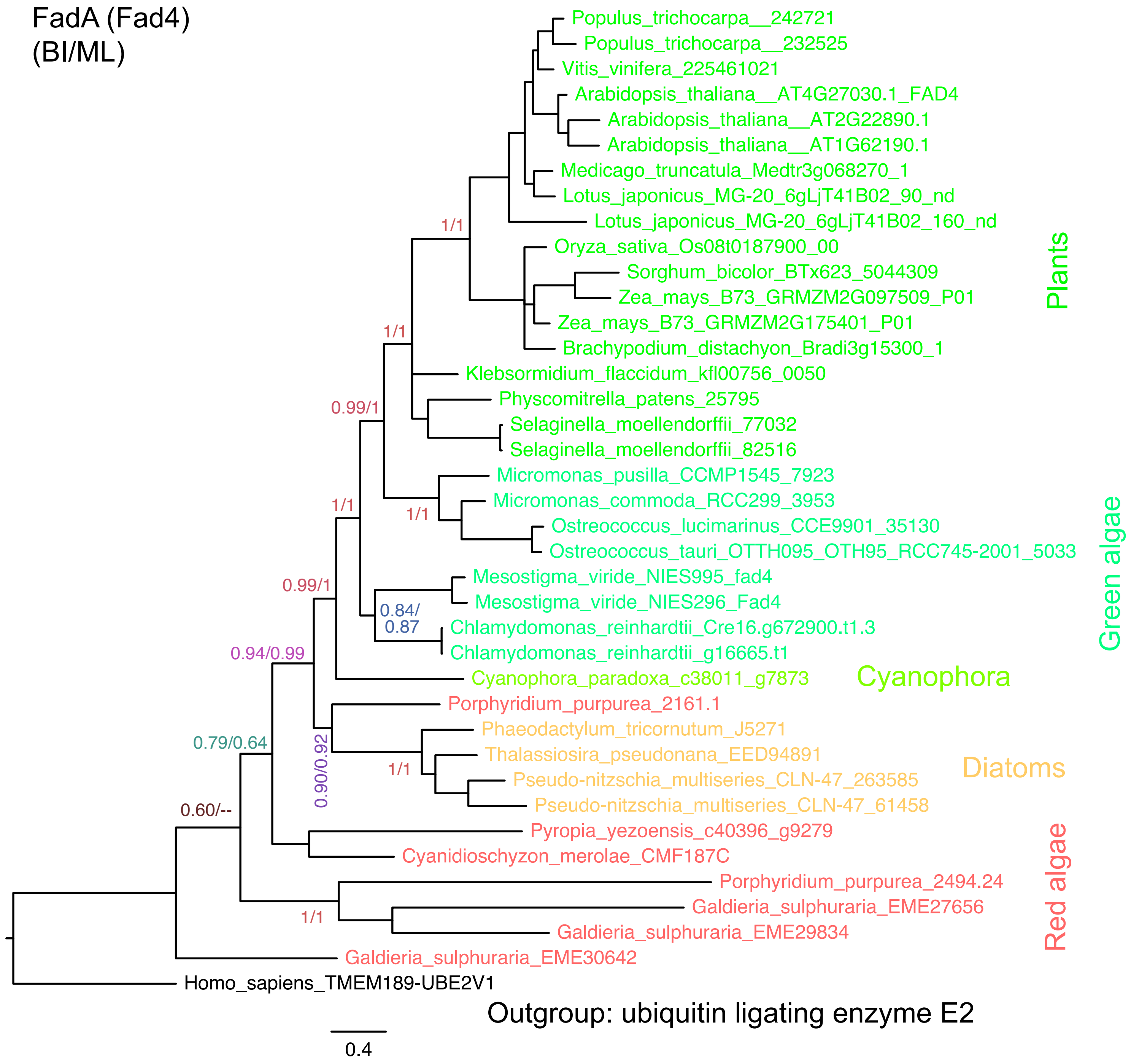
FadA (Fad4)
(BI/ML)
Populus_trichocarpa__242721
Populus_trichocarpa__232525
Vitis_vinifera_225461021
Arabidopsis_thaliana__AT4G27030.1_FAD4
Arabidopsis_thaliana__AT2G22890.1
Arabidopsis_thaliana__AT1G62190.1
Medicago_truncatula_Medtr3g068270_1
Lotus_japonicus_MG-20_6gLjT41B02_90_nd
Lotus_japonicus_MG-20_6gLjT41B02_160_nd
Oryza_sativa_Os08t0187900_00
Sorghum_bicolor_BTx623_5044309
Zea_mays_B73_GRMZM2G097509_P01
Zea_mays_B73_GRMZM2G175401_P01
Brachypodium_distachyon_Bradi3g15300_1
Klebsormidium_flaccidum_kfl00756_0050
Physcomitrella_patens_25795
Selaginella_moellendorffii_77032
Selaginella_moellendorffii_82516
Micromonas_pusilla_CCMP1545_7923
Micromonas_commoda_RCC299_3953
Ostreococcus_lucimarinus_CCE9901_35130
Ostreococcus_tauri_OTTH095_OTH95_RCC745-2001_5033
Mesostigma_viride_NIES995_fad4
Mesostigma_viride_NIES296_Fad4
Chlamydomonas_reinhardtii_Cre16.g672900.t1.3
Chlamydomonas_reinhardtii_g16665.t1
Cyanophora_paradoxa_c38011_g7873
Porphyridium_purpurea_2161.1
Phaeodactylum_tricornutum_J5271
Thalassiosira_pseudonana_EED94891
Pseudo-nitzschia_multiseries_CLN-47_263585
Pseudo-nitzschia_multiseries_CLN-47_61458
Pyropia_yezoensis_c40396_g9279
Cyanidioschyzon_merolae_CMF187C
Porphyridium_purpurea_2494.24
Galdieria_sulphuraria_EME27656
Galdieria_sulphuraria_EME29834
Galdieria_sulphuraria_EME30642
Homo_sapiens_TMEM189-UBE2V1
1/1
1/1
0.99/1
1/1
1/1
0.99/1
0.84/
0.87
0.94/0.99
0.79/0.64
0.90/0.92
1/1
0.60/--
1/1
Plants
Green algae
Cyanophora
Diatoms
Red algae
Outgroup: ubiquitin ligating enzyme E2
0.4

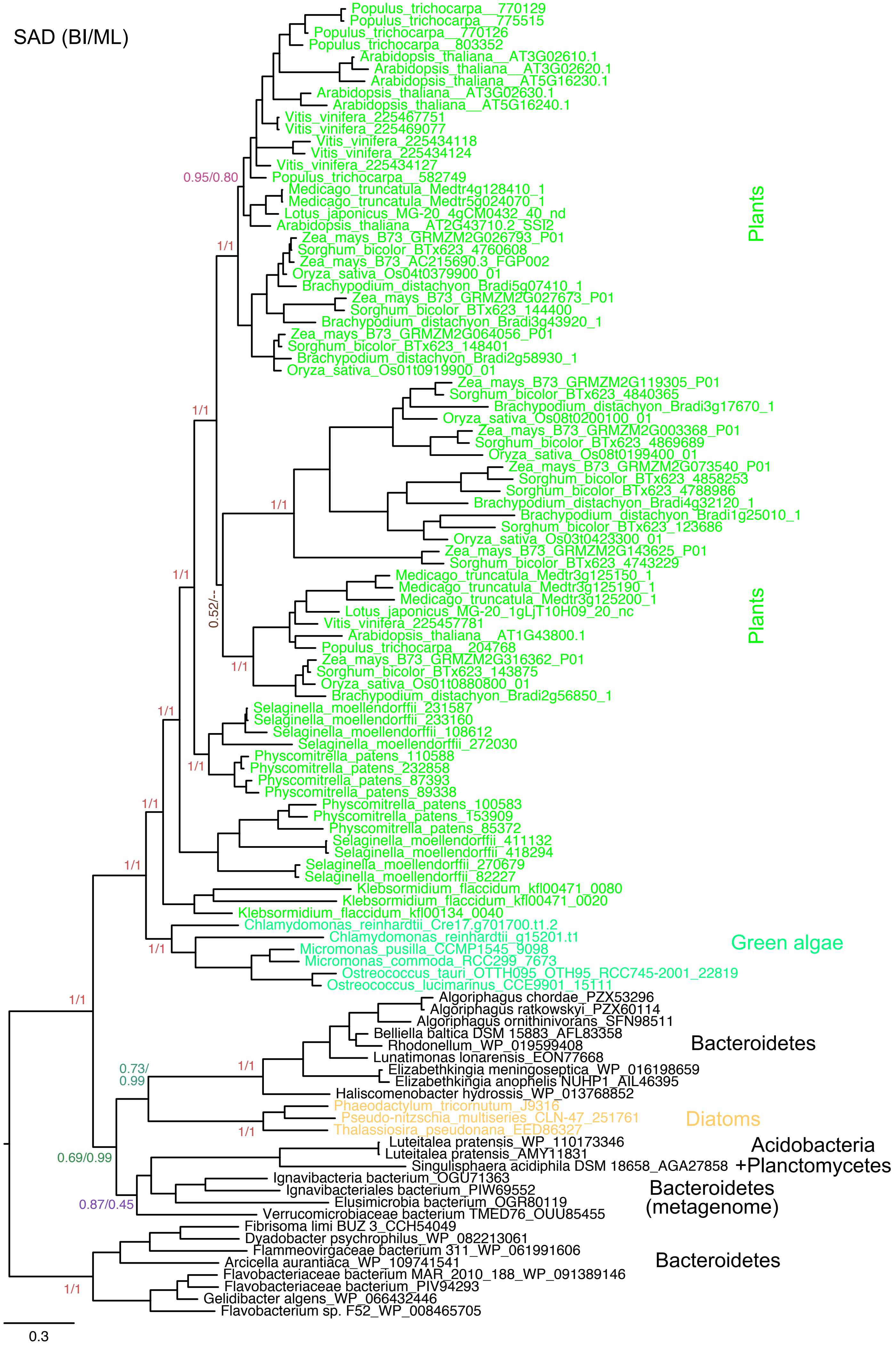
SAD (BI/ML)
Populus_trichocarpa__770129
Populus_trichocarpa__775515
Populus_trichocarpa__770126
Populus_trichocarpa__803352
Arabidopsis_thaliana__AT3G02610.1
Arabidopsis_thaliana__AT3G02620.1
Arabidopsis_thaliana__AT5G16230.1
Arabidopsis_thaliana__AT3G02630.1
Arabidopsis_thaliana__AT5G16240.1
Vitis_vinifera_225467751
Vitis_vinifera_225469077
Vitis_vinifera_225434118
Vitis_vinifera_225434124
Vitis_vinifera_225434127
0.95/0.80
Populus_trichocarpa__582749
Medicago_truncatula_Medtr4g128410_1
Medicago_truncatula_Medtr5g024070_1
Lotus_japonicus_MG-20_4gCM0432_40_nd
Arabidopsis_thaliana__AT2G43710.2_SSI2
1/1
Zea_mays_B73_GRMZM2G026793_P01
Sorghum_bicolor_BTx623_4760608
Zea_mays_B73_AC215690.3_FGP002
Oryza_sativa_Os04t0379900_01
Brachypodium_distachyon_Bradi5g07410_1
Zea_mays_B73_GRMZM2G027673_P01
Sorghum_bicolor_BTx623_144400
Brachypodium_distachyon_Bradi3g43920_1
Zea_mays_B73_GRMZM2G064056_P01
Sorghum_bicolor_BTx623_148401
Brachypodium_distachyon_Bradi2g58930_1
Oryza_sativa_Os01t0919900_01
Plants
1/1
Zea_mays_B73_GRMZM2G119305_P01
Sorghum_bicolor_BTx623_4840365
Brachypodium_distachyon_Bradi3g17670_1
Oryza_sativa_Os08t0200100_01
Zea_mays_B73_GRMZM2G003368_P01
Sorghum_bicolor_BTx623_4869689
Oryza_sativa_Os08t0199400_01
Zea_mays_B73_GRMZM2G073540_P01
Sorghum_bicolor_BTx623_4858253
Sorghum_bicolor_BTx623_4788986
1/1
Brachypodium_distachyon_Bradi4g32120_1
Brachypodium_distachyon_Bradi1g25010_1
Sorghum_bicolor_BTx623_123686
Oryza_sativa_Os03t0423300_01
Zea_mays_B73_GRMZM2G143625_P01
Sorghum_bicolor_BTx623_4743229
1/1
Medicago_truncatula_Medtr3g125150_1
Medicago_truncatula_Medtr3g125190_1
Medicago_truncatula_Medtr3g125200_1
0.52/--
Lotus_japonicus_MG-20_1gLjT10H09_20_nc
Vitis_vinifera_225457781
Arabidopsis_thaliana__AT1G43800.1
Populus_trichocarpa__204768
Plants
1/1
Zea_mays_B73_GRMZM2G316362_P01
Sorghum_bicolor_BTx623_143875
Oryza_sativa_Os01t0880800_01
Brachypodium_distachyon_Bradi2g56850_1
1/1
Selaginella_moellendorffii_231587
Selaginella_moellendorffii_233160
Selaginella_moellendorffii_108612
Selaginella_moellendorffii_272030
Physcomitrella_patens_110588
1/1
Physcomitrella_patens_232858
Physcomitrella_patens_87393
Physcomitrella_patens_89338
1/1
Physcomitrella_patens_100583
Physcomitrella_patens_153909
Physcomitrella_patens_85372
Selaginella_moellendorffii_411132
Selaginella_moellendorffii_418294
1/1
Selaginella_moellendorffii_270679
Selaginella_moellendorffii_82227
Klebsormidium_flaccidum_kfl00471_0080
Klebsormidium_flaccidum_kfl00471_0020
Klebsormidium_flaccidum_kfl00134_0040
Chlamydomonas_reinhardtii_Cre17.g701700.t1.2
Chlamydomonas_reinhardtii_g15201.t1
1/1
Green algae
Micromonas_pusilla_CCMP1545_9098
Micromonas_commoda_RCC299_7673
Ostreococcus_tauri_OTTH095_OTH95_RCC745-2001_22819
Ostreococcus_lucimarinus_CCE9901_15111
1/1
Algoriphagus chordae_PZX53296
Algoriphagus ratkowskyi_PZX60114
Algoriphagus ornithinivorans_SFN98511
Belliella baltica DSM 15883_AFL83358
Rhodonellum_WP_019599408
Bacteroidetes
Lunatimonas lonarensis_EON77668
0.73/
0.99
1/1
Elizabethkingia meningoseptica_WP_016198659
Elizabethkingia anophelis NUHP1_AIL46395
Haliscomenobacter hydrossis_WP_013768852
Phaeodactylum_tricornutum_J9316
Pseudo-nitzschia_multiseries_CLN-47_251761
Diatoms
1/1
Thalassiosira_pseudonana_EED86327
Luteitalea pratensis_WP_110173346
Acidobacteria
Luteitalea pratensis_AMY11831
0.69/0.99
Singulisphaera acidiphila DSM 18658_AGA27858
+Planctomycetes
Ignavibacteria bacterium_OGU71363
Bacteroidetes
Ignavibacteriales bacterium_PIW69552
0.87/0.45
Elusimicrobia bacterium_OGR80119
(metagenome)
Verrucomicrobiaceae bacterium TMED76_OUU85455
Fibrisoma limi BUZ 3_CCH54049
Dyadobacter psychrophilus_WP_082213061
Flammeovirgaceae bacterium 311_WP_061991606
Arcicella aurantiaca_WP_109741541
Bacteroidetes
Flavobacteriaceae bacterium MAR_2010_188_WP_091389146
1/1
Flavobacteriaceae bacterium_PIV94293
Gelidibacter algens_WP_066432446
Flavobacterium sp. F52_WP_008465705
0.3

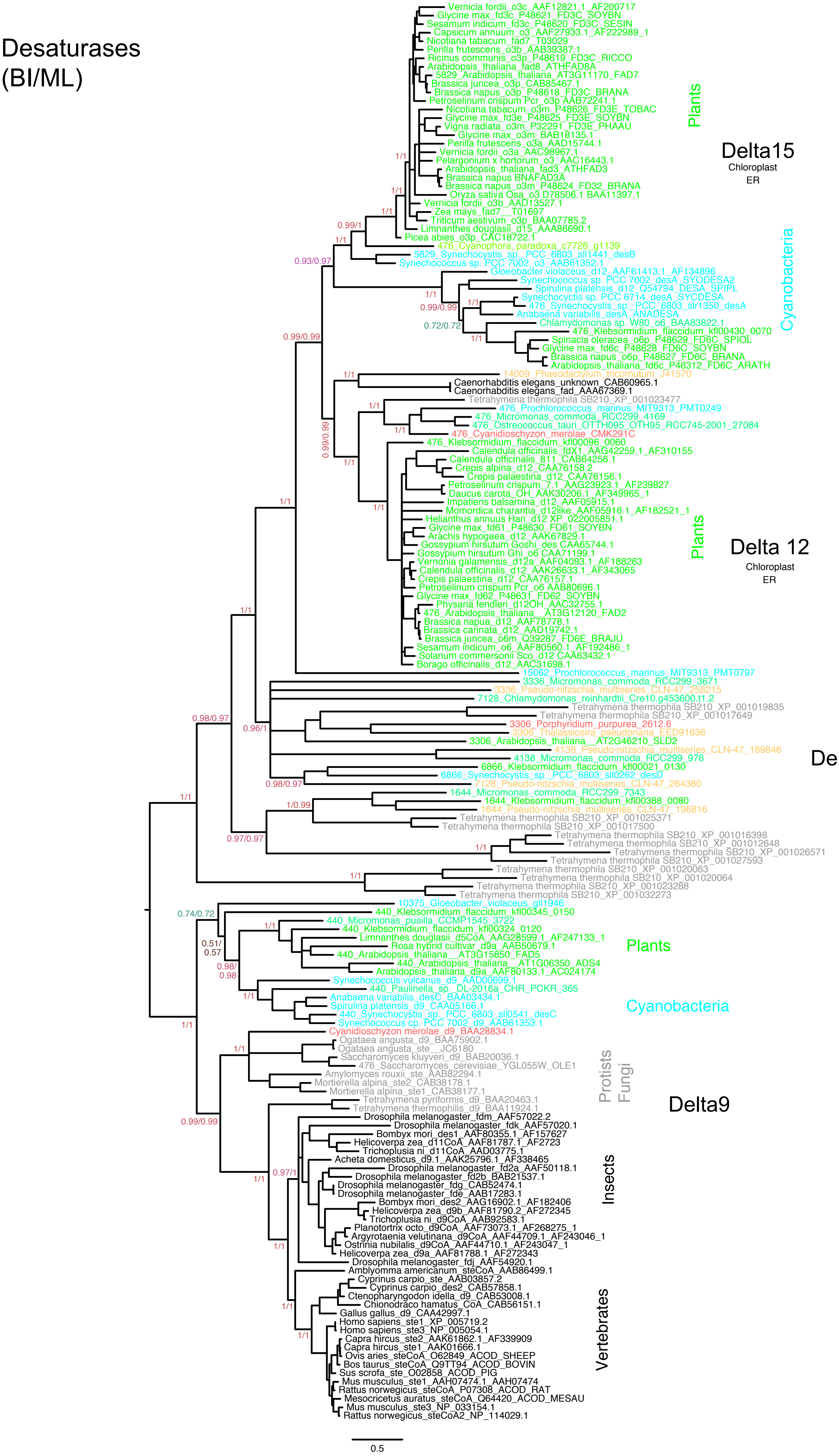

Desaturases
(BI/ML)
Vernicia fordii_o3c_AAF12821.1_AF200717
Glycine max_fd3c_P48621_FD3C_SOYBN
Sesamum indicum_fd3c_P48620_FD3C_SESIN
Capsicum annuum_o3_AAF27933.1_AF222989_1
Nicotiana tabacum_fad7_T03029
Perilla frutescens_o3b_AAB39387.1
Ricinus communis_o3p_P48619_FD3C_RICCO
Arabidopsis_thaliana_fad8_ATHFAD8A
5829_Arabidopsis_thaliana_AT3G11170_FAD7
Brassica juncea_o3p_CAB85467.1
Brassica napus_o3p_P48618_FD3C_BRANA
Petroselinum crispum Pcr_o3p AAB72241.1
Nicotiana tabacum_o3m_P48626_FD3E_TOBAC
Glycine max_fd3e_P48625_FD3E_SOYBN
Vigna radiata_o3m_P32291_FD3E_PHAAU
Glycine max_o3m_BAB18135.1
Perilla frutescens_o3a_AAD15744.1
Vernicia fordii_o3a_AAC98967.1
Pelargonium x hortorum_o3_AAC16443.1
Arabidopsis_thaliana_fad3_ATHFAD3
Brassica napus BNAFAD3A
Brassica napus_o3m_P48624_FD32_BRANA
Oryza sativa Osa_o3 D78506.1 BAA11397.1
Vernicia fordii_o3b_AAD13527.1
Zea mays_fad7__T01697
Triticum aestivum_o3p_BAA07785.2
Limnanthes douglasii_d15_AAA86690.1
Picea abies_o3p_CAC18722.1
476_Cyanophora_paradoxa_c7726_g1139
5829_Synechocystis_sp._PCC_6803_sll1441_desB
Synechococcus sp. PCC 7002_o3_AAB61352.1
Gloeobacter violaceus_d12_AAF61413.1_AF134896
Synechococcus sp. PCC 7002_desA_SYODESA2
Spirulina platensis_d12_Q54794_DESA_SPIPL
Synechocystis sp. PCC 6714_desA_SYCDESA
476_Synechocystis_sp._PCC_6803_slr1350_desA
Anabaena variabilis_desA_ANADESA
Chlamydomonas sp. W80_o6_BAA83822.1
476_Klebsormidium_flaccidum_kfl00430_0070
Spinacia oleracea_o6p_P48629_FD6C_SPIOL
Glycine max_fd6c_P48628_FD6C_SOYBN
Brassica napus_o6p_P48627_FD6C_BRANA
Arabidopsis_thaliana_fd6c_P46312_FD6C_ARATH
14009_Phaeodactylum_tricornutum_J41570
Caenorhabditis elegans_unknown_CAB60965.1
Caenorhabditis elegans_fad_AAA67369.1
Tetrahymena thermophila SB210_XP_001023477
476_Prochlorococcus_marinus_MIT9313_PMT0249
476_Micromonas_commoda_RCC299_4169
476_Ostreococcus_tauri_OTTH095_OTH95_RCC745-2001_27084
476_Cyanidioschyzon_merolae_CMK291C
476_Klebsormidium_flaccidum_kfl00096_0060
Calendula officinalis_fdX1_AAG42259.1_AF310155
Calendula officinalis_811_CAB64256.1
Crepis alpina_d12_CAA76158.2
Crepis palaestina_d12_CAA76156.1
Petroselinum crispum_7.1_AAG23923.1_AF239827
Daucus carota_OH_AAK30206.1_AF349965_1
Impatiens balsamina_d12_AAF05915.1
Momordica charantia_d12like_AAF05916.1_AF182521_1
Helianthus annuus Han_d12 XP_022005851.1
Glycine max_fd61_P48630_FD61_SOYBN
Arachis hypogaea_d12_AAK67829.1
Gossypium hirsutum Goshi_des CAA65744.1
Gossypium hirsutum Ghi_o6 CAA71199.1
Vernonia galamensis_d12a_AAF04093.1_AF188263
Calendula officinalis_d12_AAK26633.1_AF343065
Crepis palaestina_d12_CAA76157.1
Petroselinum crispum Pcr_o6 AAB80696.1
Glycine max_fd62_P48631_FD62_SOYBN
Physaria fendleri_d12OH_AAC32755.1
476_Arabidopsis_thaliana__AT3G12120_FAD2
Brassica napus_d12_AAF78778.1
Brassica carinata_d12_AAD19742.1
Brassica juncea_o6m_Q39287_FD6E_BRAJU
Sesamum indicum_o6_AAF80560.1_AF192486_1
Solanum commersonii Sco_d12 CAA63432.1
Borago officinalis_d12_AAC31698.1
15062_Prochlorococcus_marinus_MIT9313_PMT0797
3336_Micromonas_commoda_RCC299_3671
3336_Pseudo-nitzschia_multiseries_CLN-47_258215
7128_Chlamydomonas_reinhardtii_Cre10.g453600.t1.2
Tetrahymena thermophila SB210_XP_001019835
Tetrahymena thermophila SB210_XP_001017649
3306_Porphyridium_purpurea_2612.6
3306_Thalassiosira_pseudonana_EED91636
3306_Arabidopsis_thaliana__AT2G46210_SLD2
4138_Pseudo-nitzschia_multiseries_CLN-47_189846
4138_Micromonas_commoda_RCC299_976
6866_Klebsormidium_flaccidum_kfl00021_0130
6866_Synechocystis_sp._PCC_6803_sll0262_desD
7128_Pseudo-nitzschia_multiseries_CLN-47_264380
1644_Micromonas_commoda_RCC299_7343
1644_Klebsormidium_flaccidum_kfl00388_0080
1644_Pseudo-nitzschia_multiseries_CLN-47_196816
Tetrahymena thermophila SB210_XP_001025371
Tetrahymena thermophila SB210_XP_001017500
Tetrahymena thermophila SB210_XP_001016398
Tetrahymena thermophila SB210_XP_001012648
Tetrahymena thermophila SB210_XP_001026571
Tetrahymena thermophila SB210_XP_001027593
Tetrahymena thermophila SB210_XP_001020063
Tetrahymena thermophila SB210_XP_001020064
Tetrahymena thermophila SB210_XP_001023288
Tetrahymena thermophila SB210_XP_001032273
10375_Gloeobacter_violaceus_gll1946
440_Klebsormidium_flaccidum_kfl00345_0150
440_Micromonas_pusilla_CCMP1545_3722
440_Klebsormidium_flaccidum_kfl00324_0120
Limnanthes douglasii_d5CoA_AAG28599.1_AF247133_1
Rosa hybrid cultivar_d9a_AAB50679.1
440_Arabidopsis_thaliana__AT3G15850_FAD5
440_Arabidopsis_thaliana__AT1G06350_ADS4
Arabidopsis_thaliana_d9a_AAF80133.1_AC024174
Synechococcus vulcanus_d9_AAD00699.1
440_Paulinella_sp._DL-2016a_CHR_PCKR_365
Anabaena variabilis_desC_BAA03434.1
Spirulina platensis_d9_CAA05166.1
440_Synechocystis_sp._PCC_6803_sll0541_desC
Synechococcus cp. PCC 7002_d9_AAB61353.1
Cyanidioschyzon merolae_d9_BAA28834.1
Ogataea angusta_d9_BAA75902.1
Ogataea angusta_ste__JC6180
Saccharomyces kluyveri_d9_BAB20036.1
476_Saccharomyces_cerevisiae_YGL055W_OLE1
Amylomyces rouxii_ste_AAB82294.1
Mortierella alpina_ste2_CAB38178.1
Mortierella alpina_ste1_CAB38177.1
Tetrahymena pyriformis_d9_BAA20463.1
Tetrahymena thermophilis_d9_BAA11924.1
Drosophila melanogaster_fdm_AAF57022.2
Drosophila melanogaster_fdk_AAF57020.1
Bombyx mori_des1_AAF80355.1_AF157627
Helicoverpa zea_d11CoA_AAF81787.1_AF2723
Trichoplusia ni_d11CoA_AAD03775.1
Acheta domesticus_d9.1_AAK25796.1_AF338465
Drosophila melanogaster_fd2a_AAF50118.1
Drosophila melanogaster_fd2b_BAB21537.1
Drosophila melanogaster_fdg_CAB52474.1
Drosophila melanogaster_fde_AAB17283.1
Bombyx mori_des2_AAG16902.1_AF182406
Helicoverpa zea_d9b_AAF81790.2_AF272345
Trichoplusia ni_d9CoA_AAB92583.1
Planotortrix octo_d9CoA_AAF73073.1_AF268275_1
Argyrotaenia velutinana_d9CoA_AAF44709.1_AF243046_1
Ostrinia nubilalis_d9CoA_AAF44710.1_AF243047_1
Helicoverpa zea_d9a_AAF81788.1_AF272343
Drosophila melanogaster_fdj_AAF54920.1
Amblyomma americanum_steCoA_AAB86499.1
Cyprinus carpio_ste_AAB03857.2
Cyprinus carpio_des2_CAB57858.1
Ctenopharyngodon idella_d9_CAB53008.1
Chionodraco hamatus_CoA_CAB56151.1
Gallus gallus_d9_CAA42997.1
Homo sapiens_ste1_XP_005719.2
Homo sapiens_ste3_NP_005054.1
Capra hircus_ste2_AAK61862.1_AF339909
Capra hircus_ste1_AAK01666.1
Ovis aries_steCoA_O62849_ACOD_SHEEP
Bos taurus_steCoA_Q9TT94_ACOD_BOVIN
Sus scrofa_ste_O02858_ACOD_PIG
Mus musculus_ste1_AAH07474.1_AAH07474
Rattus norvegicus_steCoA_P07308_ACOD_RAT
Mesocricetus auratus_steCoA_Q64420_ACOD_MESAU
Mus musculus_ste3_NP_033154.1
Rattus norvegicus_steCoA2_NP_114029.1
Plants
Delta15
Chloroplast
ER
Cyanobacteria
Plants
Delta 12
Chloroplast
ER
Delta6
Protists
Plants
Cyanobacteria
Protists
Fungi
Delta9
Insects
Vertebrates
1/1
0.99/1
0.93/0.97
0.99/0.99
0.72/0.72
0.99/0.99
0.98/0.97
0.96/1
0.98/0.97
1/0.99
0.97/0.97
0.74/0.72
0.51/
0.57
0.98/
0.98
0.97/1
0.99/0.99
0.5
