# Supplementary material for: Complex origins of chloroplast membranes with photosynthetic machineries: multiple transfers of genes from divergent organisms at different times or a single endosymbiotic event?
Source: J Plant Res. 2019 Dec 6;133(1):15–33. doi: 10.1007/s10265-019-01157-z (PMC6946739; doi:10.1007/s10265-019-01157-z)

Journal of Plant Research

Online Resources

Complex origins of chloroplast membranes with photosynthetic machineries: Multiple transfers of genes from divergent organisms at different times or a single endosymbiotic event?

Naoki Sato

University of Tokyo

naokisat@bio.c.u-tokyo.ac.jp

Online Resource 4. Phylogenetic trees of chloroplast-encoded rRNA and proteins.

16S-23S rRNA, a combined tree of 16S rRNA and 23S rRNA; 33 proteins (33P in Fig. 5), a combined tree with 33 chloroplast-encoded proteins in Tajima et al. 2016, re-analyzed with the LG model; PsaA, Photosystem I reaction center protein; PsaB, Photosystem I core protein; PsaAB, a tree including selected PsaA and PsaB; PsaD and PsaE, nuclear-encoded Photosystem I proteins; Set A48, a combined tree of 23 chloroplast-encoded photosynthesis-related proteins conserved in 48 organisms (in the Gclust dataset CPBACT10); Set A53, a combined tree of 23 chloroplast-encoded house-keeping proteins conserved in 53 organisms; Set A53 selected (A53-2), a combined tree of 9 selected proteins in the set A53; Set B42, 53 chloroplast-encoded proteins conserved in red algae and bacteria (42 species); Set F35, 20 chloroplast-encoded proteins conserved in macrophytic red algae (35 species); Set B37, 24 chloroplast-encoded proteins conserved in red algae and cyanobacteria (37 species); Set E48, three chloroplast-encoded chlorophyll synthesis enzymes conserved in 48 organisms; RbcL, ribulose-1,5-bisphosphate carboxylase/oxygenase large subunit; RpoA, RNA polymerase subunit α; RpoB (two pages), RNA polymerase subunit β; RpoC (two pages), RNA polymerase subunit β' in bacteria, and subunits β' and β'' that are split in cyanobacteria and chloroplasts.

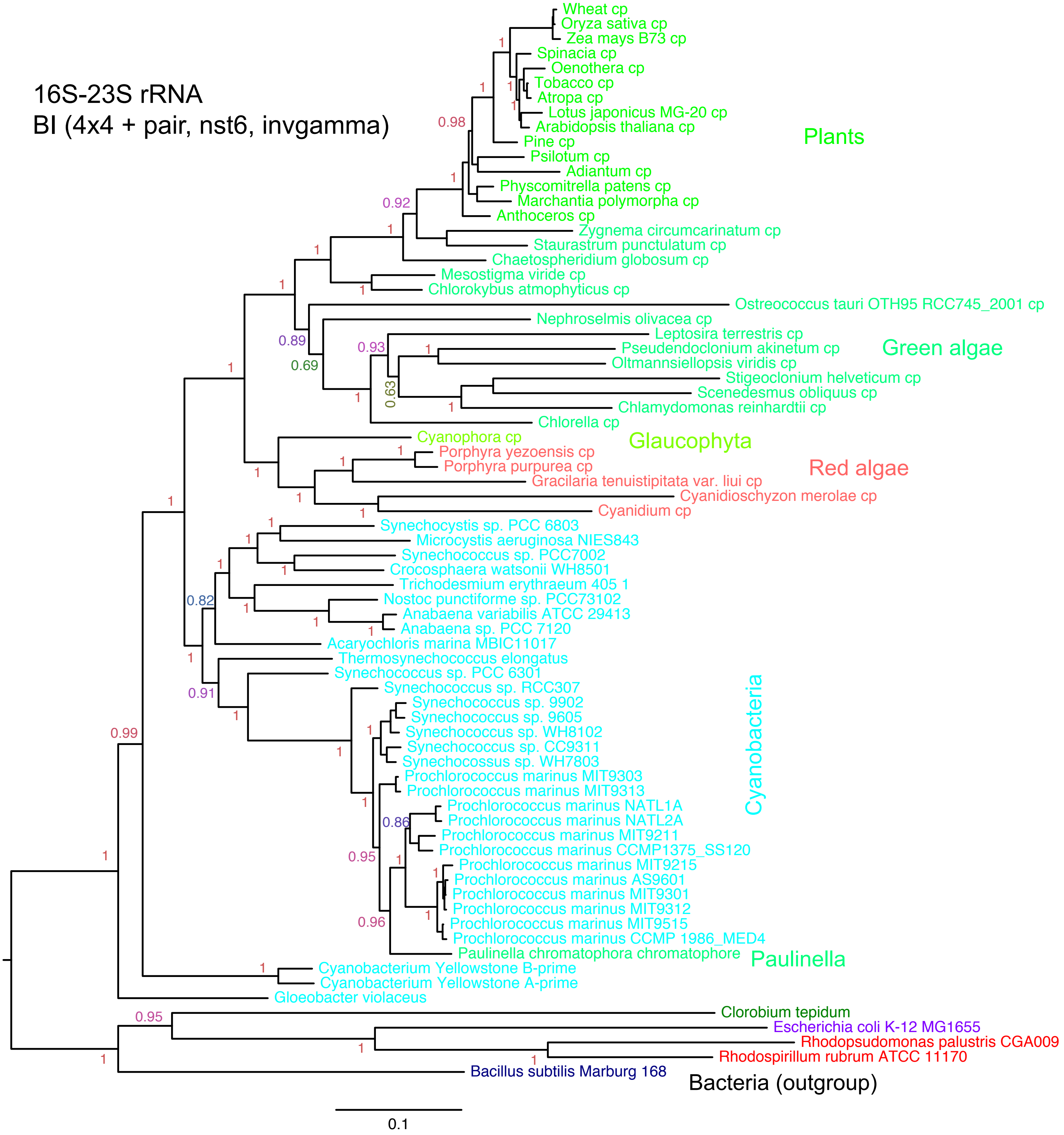
16S-23S rRNA
BI (4x4 + pair, nst6, invgamma)
Wheat cp
Oryza sativa cp
Zea mays B73 cp
Spinacia cp
Oenothera cp
Tobacco cp
Atropa cp
Lotus japonicus MG-20 cp
Arabidopsis thaliana cp
Pine cp
Psilotum cp
Adiantum cp
Physcomitrella patens cp
Marchantia polymorpha cp
Anthoceros cp
Zygnema circumcarinatum cp
Staurastrum punctulatum cp
Chaetospheridium globosum cp
Mesostigma viride cp
Chlorokybus atmophyticus cp
Ostreococcus tauri OTH95 RCC745_2001 cp
Nephroselmis olivacea cp
Leptosira terrestris cp
Pseudendoclonium akinetum cp
Oltmannsiellopsis viridis cp
Stigeoclonium helveticum cp
Scenedesmus obliquus cp
Chlamydomonas reinhardtii cp
Chlorella cp
Cyanophora cp
Porphyra yezoensis cp
Porphyra purpurea cp
Gracilaria tenuistipitata var. liui cp
Cyanidioschyzon merolae cp
Cyanidium cp
Synechocystis sp. PCC 6803
Microcystis aeruginosa NIES843
Synechococcus sp. PCC7002
Crocosphaera watsonii WH8501
Trichodesmium erythraeum 405 1
Nostoc punctiforme sp. PCC73102
Anabaena variabilis ATCC 29413
Anabaena sp. PCC 7120
Acaryochloris marina MBIC11017
Thermosynechococcus elongatus
Synechococcus sp. PCC 6301
Synechococcus sp. RCC307
Synechococcus sp. 9902
Synechococcus sp. 9605
Synechococcus sp. WH8102
Synechococcus sp. CC9311
Synechocossus sp. WH7803
Prochlorococcus marinus MIT9303
Prochlorococcus marinus MIT9313
Prochlorococcus marinus NATL1A
Prochlorococcus marinus NATL2A
Prochlorococcus marinus MIT9211
Prochlorococcus marinus CCMP1375_SS120
Prochlorococcus marinus MIT9215
Prochlorococcus marinus AS9601
Prochlorococcus marinus MIT9301
Prochlorococcus marinus MIT9312
Prochlorococcus marinus MIT9515
Prochlorococcus marinus CCMP 1986_MED4
Paulinella chromatophora chromatophore
Cyanobacterium Yellowstone B-prime
Cyanobacterium Yellowstone A-prime
Gloeobacter violaceus
Clorobium tepidum
Escherichia coli K-12 MG1655
Rhodopsudomonas palustris CGA009
Rhodospirillum rubrum ATCC 11170
Bacillus subtilis Marburg 168
Plants
Green algae
Glaucophyta
Red algae
Cyanobacteria
Paulinella
Bacteria (outgroup)
0.98
0.92
0.89
0.69
0.93
0.63
0.82
0.91
0.99
0.86
0.95
0.96
0.95
0.1

Set A48
(23 proteins of photosynthesis conserved in 48 organisms)
(BI)

PsaA, PsaB, PsaC, PsbA, PsbB, PsbC, PsbD, PsbE, PsbF, PsbH, PsbI, PsbJ, PsbK, PsbL, PsbN, PetA, PetB, PetD, PetG, Ycf3, Ycf4, CcsA, AtpF

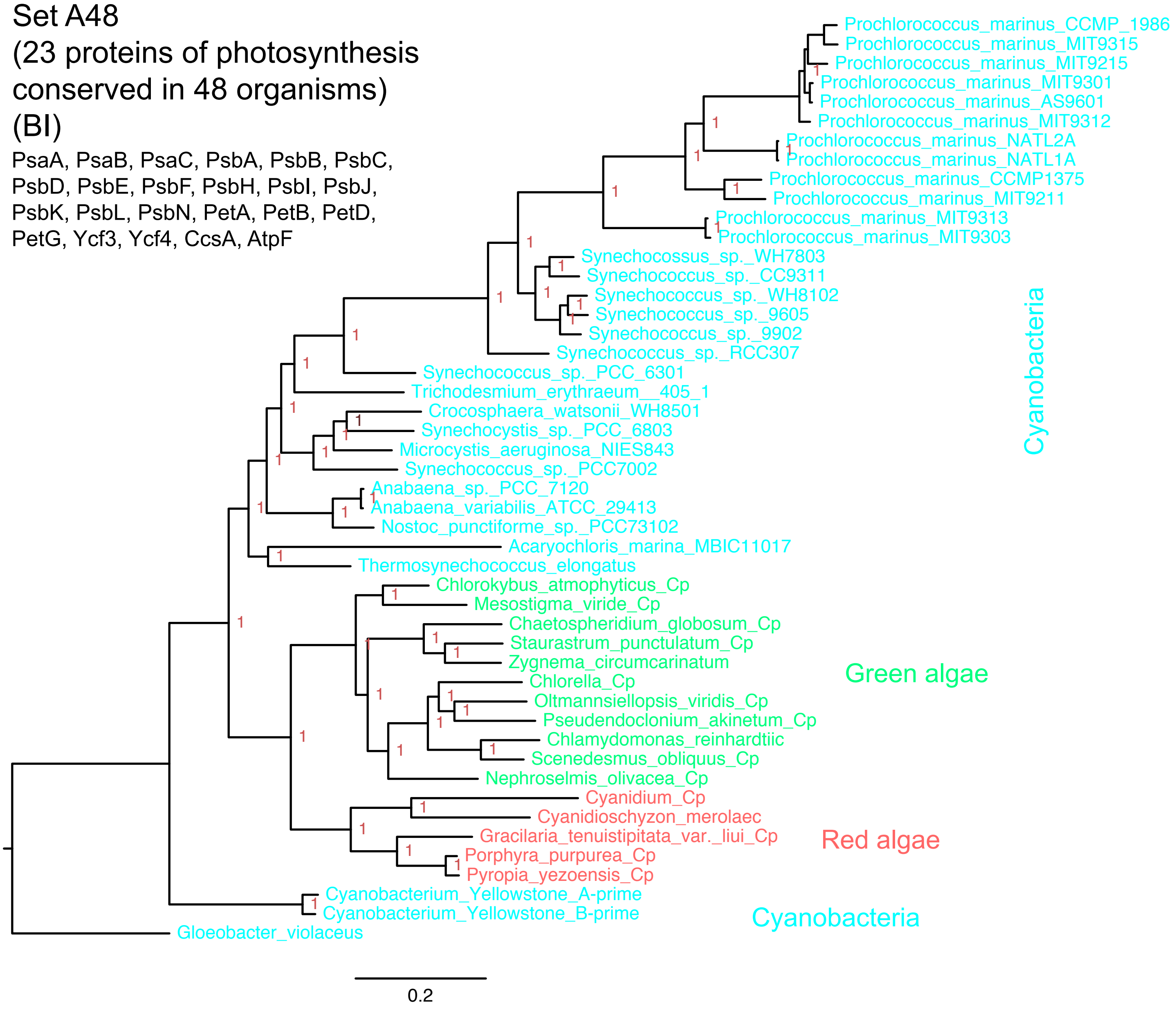

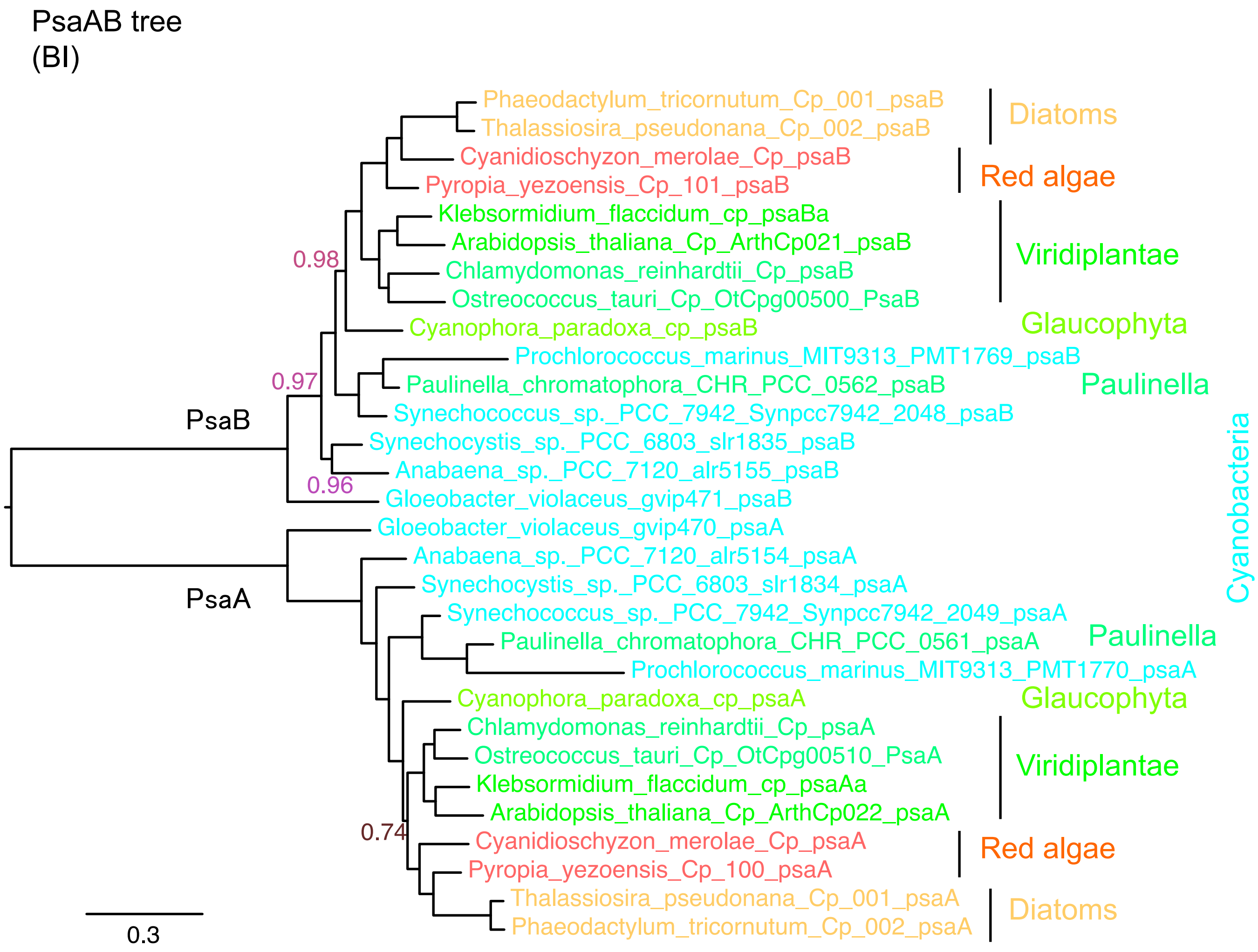
PsaAB tree
(BI)
Phaeodactylum_tricornutum_Cp_001_psaB
Thalassiosira_pseudonana_Cp_002_psaB
Cyanidioschyzon_merolae_Cp_psaB
Pyropia_yezoensis_Cp_101_psaB
Klebsormidium_flaccidum_cp_psaBa
Arabidopsis_thaliana_Cp_ArthCp021_psaB
Chlamydomonas_reinhardtii_Cp_psaB
Ostreococcus_tauri_Cp_OtCpg00500_PsaB
Cyanophora_paradoxa_cp_psaB
Prochlorococcus_marinus_MIT9313_PMT1769_psaB
Paulinella_chromatophora_CHR_PCC_0562_psaB
Synechococcus_sp._PCC_7942_Synpcc7942_2048_psaB
Synechocystis_sp._PCC_6803_slr1835_psaB
Anabaena_sp._PCC_7120_alr5155_psaB
Gloeobacter_violaceus_gvip471_psaB
Gloeobacter_violaceus_gvip470_psaA
Anabaena_sp._PCC_7120_alr5154_psaA
Synechocystis_sp._PCC_6803_slr1834_psaA
Synechococcus_sp._PCC_7942_Synpcc7942_2049_psaA
Paulinella_chromatophora_CHR_PCC_0561_psaA
Prochlorococcus_marinus_MIT9313_PMT1770_psaA
Cyanophora_paradoxa_cp_psaA
Chlamydomonas_reinhardtii_Cp_psaA
Ostreococcus_tauri_Cp_OtCpg00510_PsaA
Klebsormidium_flaccidum_cp_psaAa
Arabidopsis_thaliana_Cp_ArthCp022_psaA
Cyanidioschyzon_merolae_Cp_psaA
Pyropia_yezoensis_Cp_100_psaA
Thalassiosira_pseudonana_Cp_001_psaA
Phaeodactylum_tricornutum_Cp_002_psaA
0.98
0.97
0.96
0.74
PsaB
PsaA
0.3
Diatoms
Red algae
Viridiplantae
Glaucophyta
Paulinella
Cyanobacteria
Paulinella
Glaucophyta
Viridiplantae
Red algae
Diatoms

PsaA
BI

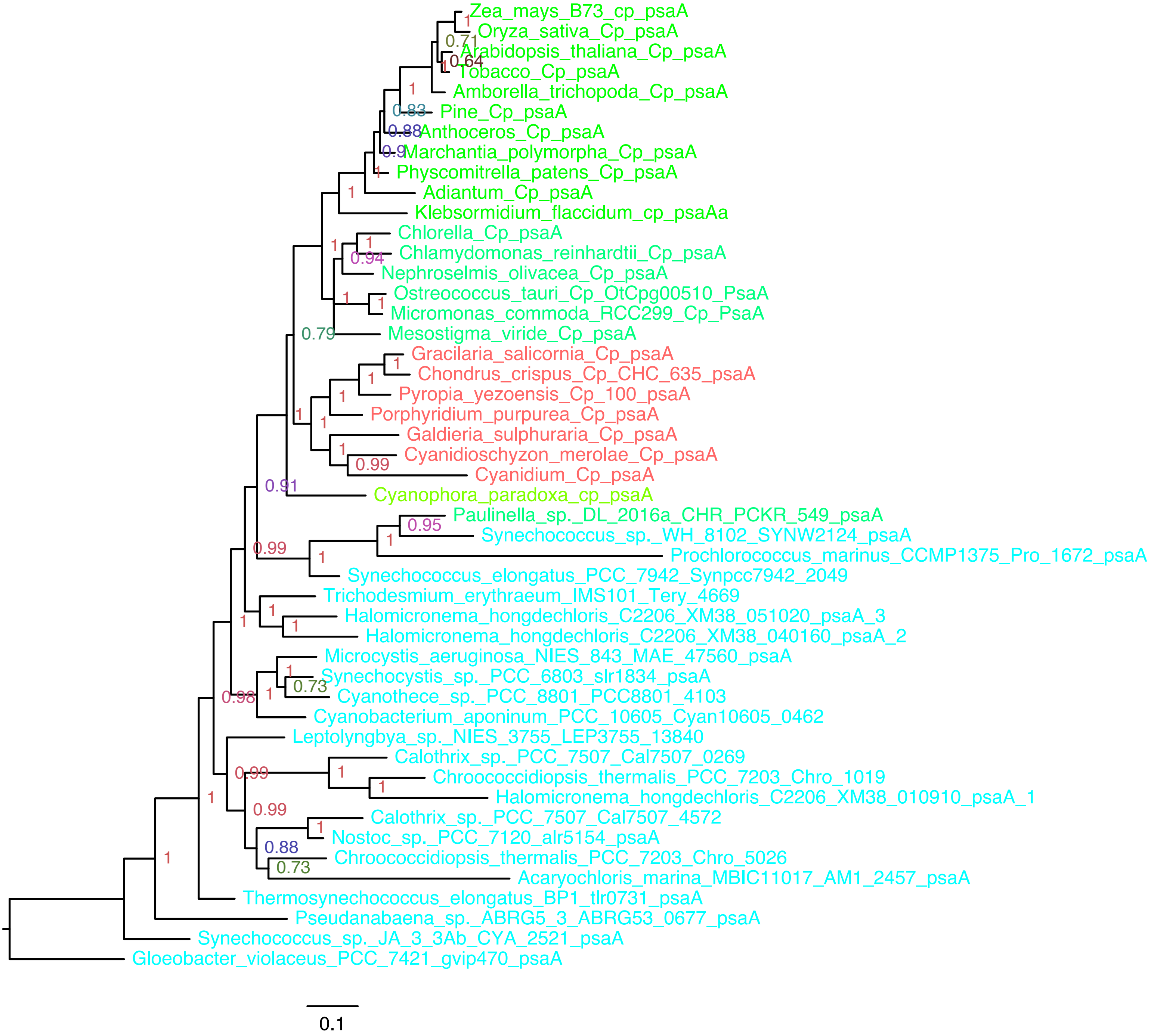
Zea_mays_B73_cp_psaA
Oryza_sativa_Cp_psaA
Arabidopsis_thaliana_Cp_psaA
Tobacco_Cp_psaA
Amborella_trichopoda_Cp_psaA
Pine_Cp_psaA
Anthoceros_Cp_psaA
Marchantia_polymorpha_Cp_psaA
Physcomitrella_patens_Cp_psaA
Adiantum_Cp_psaA
Klebsormidium_flaccidum_cp_psaAa
Chlorella_Cp_psaA
Chlamydomonas_reinhardtii_Cp_psaA
Nephroselmis_olivacea_Cp_psaA
Ostreococcus_tauri_Cp_OtCpg00510_PsaA
Micromonas_commoda_RCC299_Cp_PsaA
Mesostigma_viride_Cp_psaA
Gracilaria_salicornia_Cp_psaA
Chondrus_crispus_Cp_CHC_635_psaA
Pyropia_yezoensis_Cp_100_psaA
Porphyridium_purpurea_Cp_psaA
Galdieria_sulphuraria_Cp_psaA
Cyanidioschyzon_merolae_Cp_psaA
Cyanidium_Cp_psaA
Cyanophora_paradoxa_cp_psaA
Paulinella_sp._DL_2016a_CHR_PCKR_549_psaA
Synechococcus_sp._WH_8102_SYNW2124_psaA
Prochlorococcus_marinus_CCMP1375_Pro_1672_psaA
Synechococcus_elongatus_PCC_7942_Synpcc7942_2049
Trichodesmium_erythraeum_IMS101_Tery_4669
Halomicronema_hongdechloris_C2206_XM38_051020_psaA_3
Halomicronema_hongdechloris_C2206_XM38_040160_psaA_2
Microcystis_aeruginosa_NIES_843_MAE_47560_psaA
Synechocystis_sp._PCC_6803_slr1834_psaA
Cyanothece_sp._PCC_8801_PCC8801_4103
Cyanobacterium_aponinum_PCC_10605_Cyan10605_0462
Leptolyngbya_sp._NIES_3755_LEP3755_13840
Calothrix_sp._PCC_7507_Cal7507_0269
Chroococcidiopsis_thermalis_PCC_7203_Chro_1019
Halomicronema_hongdechloris_C2206_XM38_010910_psaA_1
Calothrix_sp._PCC_7507_Cal7507_4572
Nostoc_sp._PCC_7120_alr5154_psaA
Chroococcidiopsis_thermalis_PCC_7203_Chro_5026
Acaryochloris_marina_MBIC11017_AM1_2457_psaA
Thermosynechococcus_elongatus_BP1_tlr0731_psaA
Pseudanabaena_sp._ABRG5_3_ABRG53_0677_psaA
Synechococcus_sp._JA_3_3Ab_CYA_2521_psaA
Gloeobacter_violaceus_PCC_7421_gvip470_psaA
1
0.71
1
0.64
1
0.83
0.88
0.9
1
1
1
1
0.94
1
1
0.79
1
1
1
1
1
1
0.99
0.91
0.95
1
0.99
1
1
1
1
1
0.73
0.98
1
0.99
1
1
0.99
1
0.88
0.73
1
1
0.1

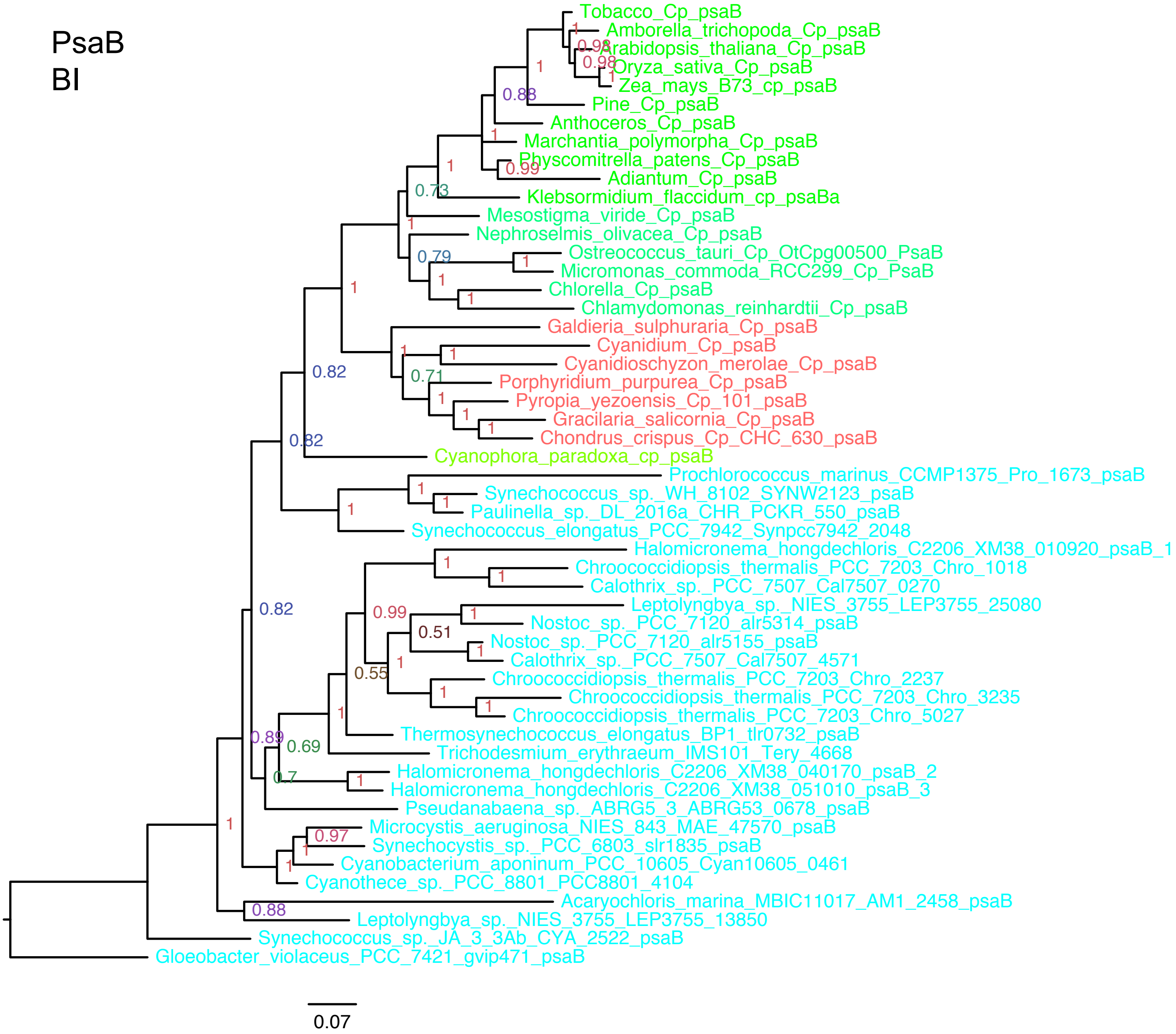
PsaB
BI
Tobacco_Cp_psaB
Amborella_trichopoda_Cp_psaB
Arabidopsis_thaliana_Cp_psaB
Oryza_sativa_Cp_psaB
Zea_mays_B73_cp_psaB
Pine_Cp_psaB
Anthoceros_Cp_psaB
Marchantia_polymorpha_Cp_psaB
Physcomitrella_patens_Cp_psaB
Adiantum_Cp_psaB
Klebsormidium_flaccidum_cp_psaBa
Mesostigma_viride_Cp_psaB
Nephroselmis_olivacea_Cp_psaB
Ostreococcus_tauri_Cp_OtCpg00500_PsaB
Micromonas_commoda_RCC299_Cp_PsaB
Chlorella_Cp_psaB
Chlamydomonas_reinhardtii_Cp_psaB
Galdieria_sulphuraria_Cp_psaB
Cyanidium_Cp_psaB
Cyanidioschyzon_merolae_Cp_psaB
Porphyridium_purpurea_Cp_psaB
Pyropia_yezoensis_Cp_101_psaB
Gracilaria_salicornia_Cp_psaB
Chondrus_crispus_Cp_CHC_630_psaB
Cyanophora_paradoxa_cp_psaB
Prochlorococcus_marinus_CCMP1375_Pro_1673_psaB
Synechococcus_sp._WH_8102_SYNW2123_psaB
Paulinella_sp._DL_2016a_CHR_PCKR_550_psaB
Synechococcus_elongatus_PCC_7942_Synpcc7942_2048
Halomicronema_hongdechloris_C2206_XM38_010920_psaB_1
Chroococcidiopsis_thermalis_PCC_7203_Chro_1018
Calothrix_sp._PCC_7507_Cal7507_0270
Leptolyngbya_sp._NIES_3755_LEP3755_25080
Nostoc_sp._PCC_7120_alr5314_psaB
Nostoc_sp._PCC_7120_alr5155_psaB
Calothrix_sp._PCC_7507_Cal7507_4571
Chroococcidiopsis_thermalis_PCC_7203_Chro_2237
Chroococcidiopsis_thermalis_PCC_7203_Chro_3235
Chroococcidiopsis_thermalis_PCC_7203_Chro_5027
Thermosynechococcus_elongatus_BP1_tlr0732_psaB
Trichodesmium_erythraeum_IMS101_Tery_4668
Halomicronema_hongdechloris_C2206_XM38_040170_psaB_2
Halomicronema_hongdechloris_C2206_XM38_051010_psaB_3
Pseudanabaena_sp._ABRG5_3_ABRG53_0678_psaB
Microcystis_aeruginosa_NIES_843_MAE_47570_psaB
Synechocystis_sp._PCC_6803_slr1835_psaB
Cyanobacterium_aponinum_PCC_10605_Cyan10605_0461
Cyanothece_sp._PCC_8801_PCC8801_4104
Acaryochloris_marina_MBIC11017_AM1_2458_psaB
Leptolyngbya_sp._NIES_3755_LEP3755_13850
Synechococcus_sp._JA_3_3Ab_CYA_2522_psaB
Gloeobacter_violaceus_PCC_7421_gvip471_psaB
0.07

Set B37
(24 proteins conserved in
red algae)
(BI)

AcsF, AtpG, ChlI, ChlD, ClpC, OdpA, OdpB, PsaD, PetF, PsaE, PsaF, PsaK, PsbW/Psb28, RpoZ, SufC, ThiS, TrpG, Ycf19, Ycf23, Ycf24, Ycf39, Ycf44,Ycf52, Ycf60,

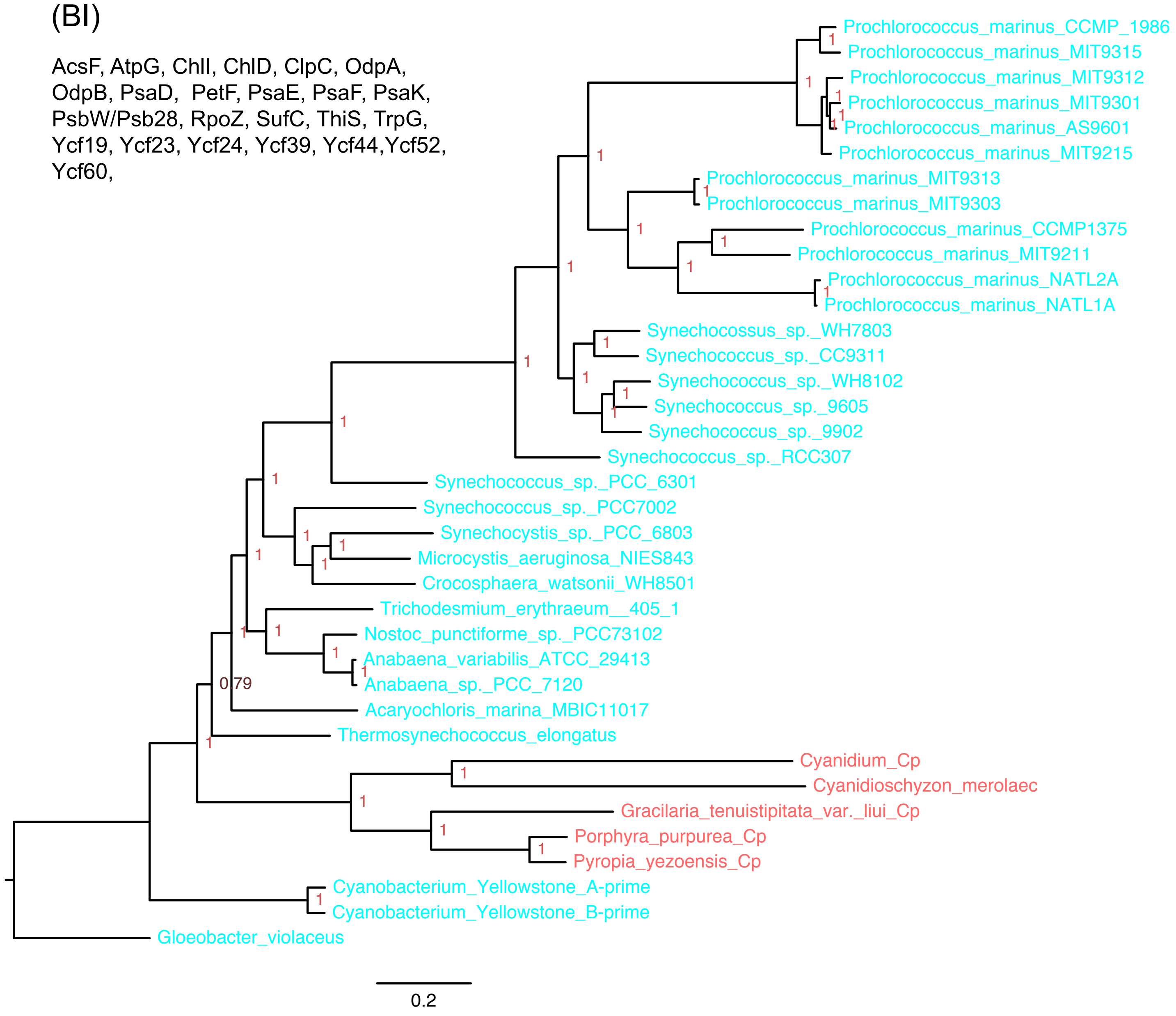

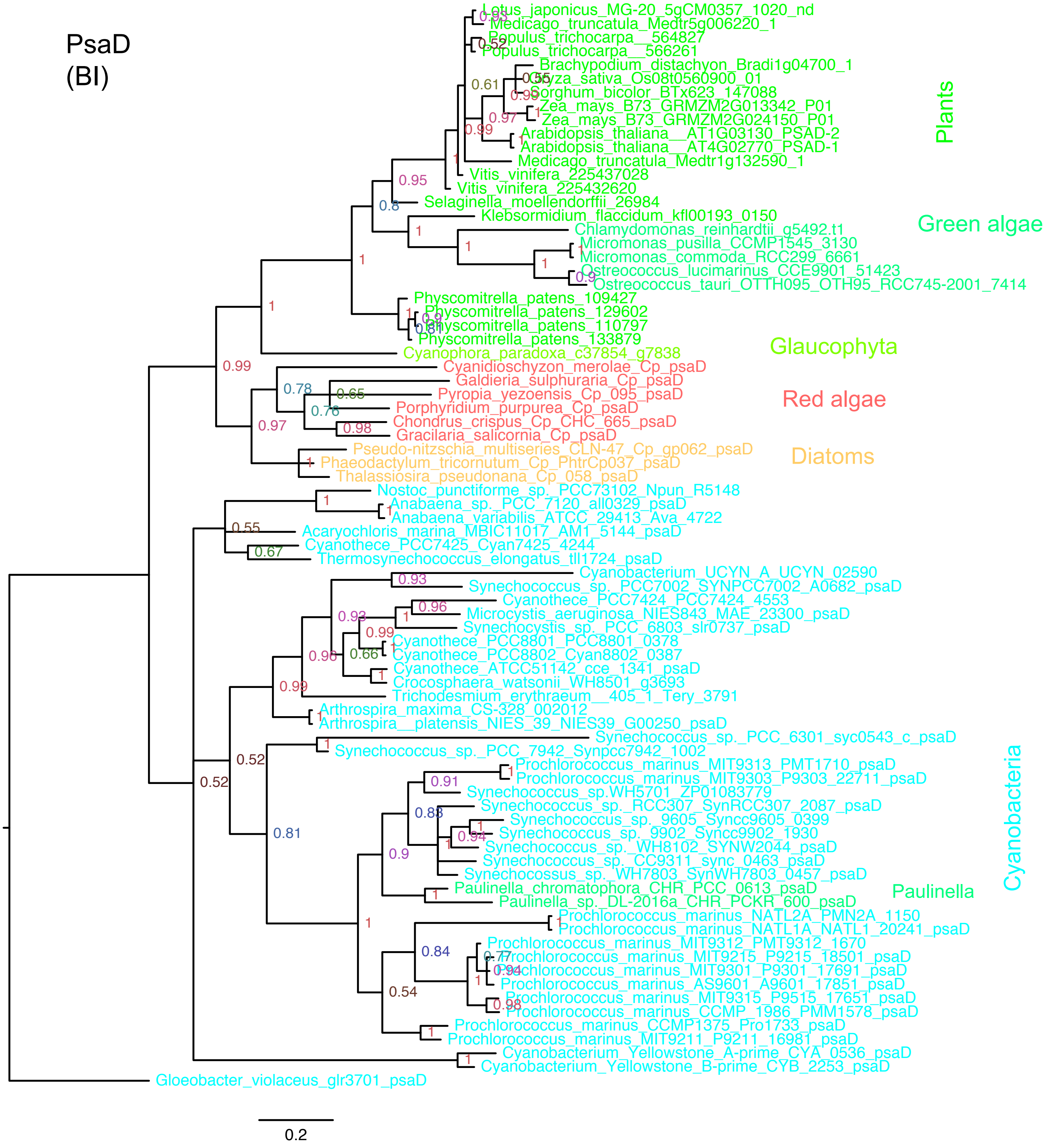
PsaD
(BI)
Lotus_japonicus_MG-20_5gCM0357_1020_nd
Medicago_truncatula_Medtr5g006220_1
Populus_trichocarpa__564827
Populus_trichocarpa__566261
Brachypodium_distachyon_Bradi1g04700_1
Oryza_sativa_Os08t0560900_01
Sorghum_bicolor_BTx623_147088
Zea_mays_B73_GRMZM2G013342_P01
Zea_mays_B73_GRMZM2G024150_P01
Arabidopsis_thaliana__AT1G03130_PSAD-2
Arabidopsis_thaliana__AT4G02770_PSAD-1
Medicago_truncatula_Medtr1g132590_1
Vitis_vinifera_225437028
Vitis_vinifera_225432620
Selaginella_moellendorffii_26984
Klebsormidium_flaccidum_kfl00193_0150
Chlamydomonas_reinhardtii_g5492.t1
Micromonas_pusilla_CCMP1545_3130
Micromonas_commoda_RCC299_6661
Ostreococcus_lucimarinus_CCE9901_51423
Ostreococcus_tauri_OTTH095_OTH95_RCC745-2001_7414
Physcomitrella_patens_109427
Physcomitrella_patens_129602
Physcomitrella_patens_110797
Physcomitrella_patens_133879
Cyanophora_paradoxa_c37854_g7838
Cyanidioschyzon_merolae_Cp_psaD
Galdieria_sulphuraria_Cp_psaD
Pyropia_yezoensis_Cp_095_psaD
Porphyridium_purpurea_Cp_psaD
Chondrus_crispus_Cp_CHC_665_psaD
Gracilaria_salicornia_Cp_psaD
Pseudo-nitzschia_multiseries_CLN-47_Cp_gp062_psaD
Phaeodactylum_tricornutum_Cp_PhtrCp037_psaD
Thalassiosira_pseudonana_Cp_058_psaD
Nostoc_punctiforme_sp._PCC73102_Npun_R5148
Anabaena_sp._PCC_7120_all0329_psaD
Anabaena_variabilis_ATCC_29413_Ava_4722
Acaryochloris_marina_MBIC11017_AM1_5144_psaD
Cyanothece_PCC7425_Cyan7425_4244
Thermosynechococcus_elongatus_tll1724_psaD
Cyanobacterium_UCYN_A_UCYN_02590
Synechococcus_sp._PCC7002_SYNPCC7002_A0682_psaD
Cyanothece_PCC7424_PCC7424_4553
Microcystis_aeruginosa_NIES843_MAE_23300_psaD
Synechocystis_sp._PCC_6803_slr0737_psaD
Cyanothece_PCC8801_PCC8801_0378
Cyanothece_PCC8802_Cyan8802_0387
Cyanothece_ATCC51142_cce_1341_psaD
Crocosphaera_watsonii_WH8501_g3693
Trichodesmium_erythraeum__405_1_Tery_3791
Arthrospira_maxima_CS-328_002012
Arthrospira__platensis_NIES_39_NIES39_G00250_psaD
Synechococcus_sp._PCC_6301_syc0543_c_psaD
Synechococcus_sp._PCC_7942_Synpcc7942_1002
Prochlorococcus_marinus_MIT9313_PMT1710_psaD
Prochlorococcus_marinus_MIT9303_P9303_22711_psaD
Synechococcus_sp.WH5701_ZP01083779
Synechococcus_sp._RCC307_SynRCC307_2087_psaD
Synechococcus_sp._9605_Syncc9605_0399
Synechococcus_sp._9902_Syncc9902_1930
Synechococcus_sp._WH8102_SYNW2044_psaD
Synechococcus_sp._CC9311_sync_0463_psaD
Synechocossus_sp._WH7803_SynWH7803_0457_psaD
Paulinella_chromatophora_CHR_PCC_0613_psaD
Paulinella_sp._DL-2016a_CHR_PCKR_600_psaD
Prochlorococcus_marinus_NATL2A_PMN2A_1150
Prochlorococcus_marinus_NATL1A_NATL1_20241_psaD
Prochlorococcus_marinus_MIT9312_PMT9312_1670
Prochlorococcus_marinus_MIT9215_P9215_18501_psaD
Prochlorococcus_marinus_MIT9301_P9301_17691_psaD
Prochlorococcus_marinus_AS9601_A9601_17851_psaD
Prochlorococcus_marinus_MIT9515_P9515_17651_psaD
Prochlorococcus_marinus_CCMP_1986_PMM1578_psaD
Prochlorococcus_marinus_CCMP1375_Pro1733_psaD
Prochlorococcus_marinus_MIT9211_P9211_16981_psaD
Cyanobacterium_Yellowstone_A-prime_CYA_0536_psaD
Cyanobacterium_Yellowstone_B-prime_CYB_2253_psaD
Gloeobacter_violaceus_glr3701_psaD
Plants
Green algae
Glaucophyta
Red algae
Diatoms
Paulinella
Cyanobacteria
0.2

PsaE
(BI)

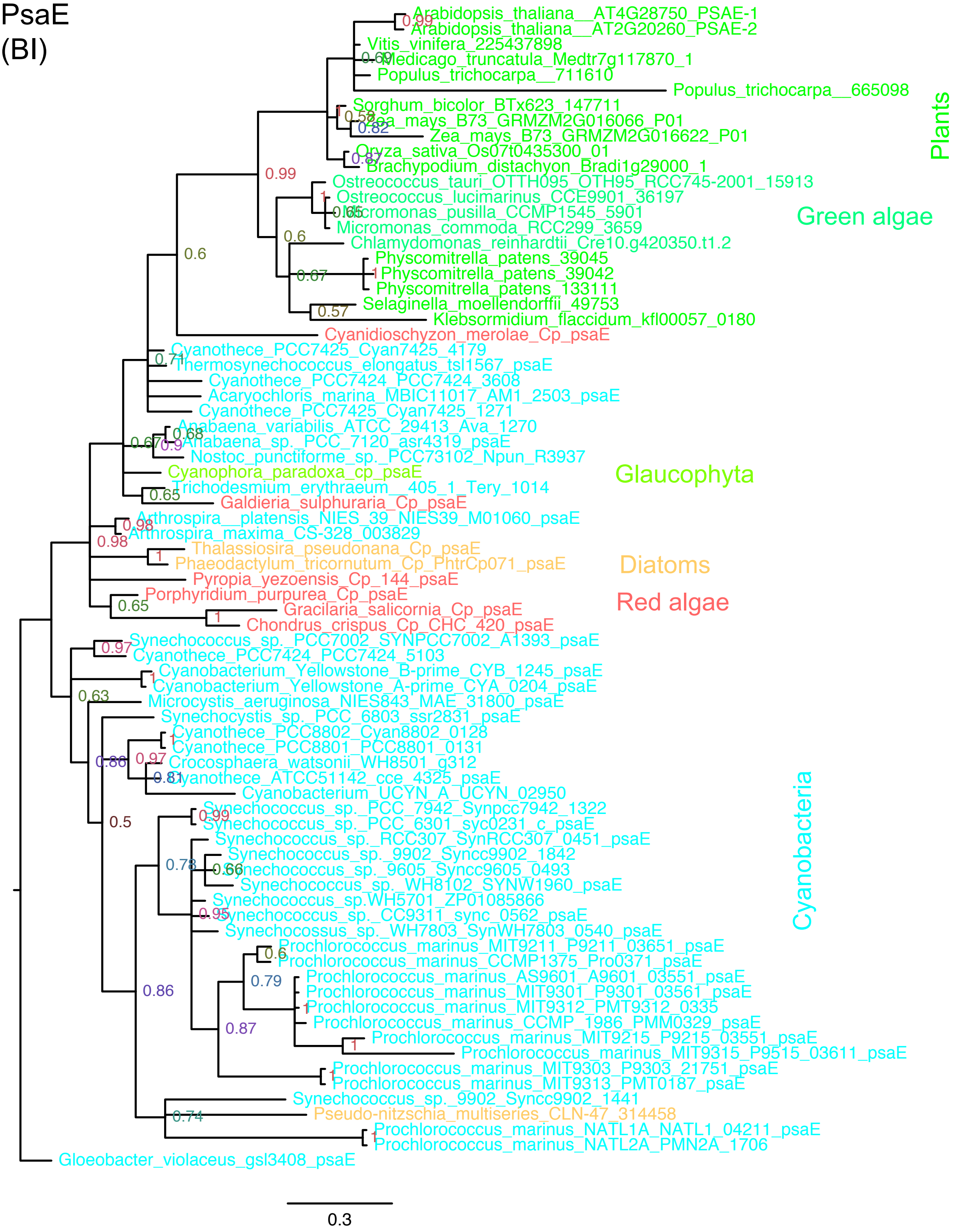
Arabidopsis_thaliana__AT4G28750_PSAE-1
Arabidopsis_thaliana__AT2G20260_PSAE-2
Vitis_vinifera_225437898
Medicago_truncatula_Medtr7g117870_1
Populus_trichocarpa__711610
Populus_trichocarpa__665098
Sorghum_bicolor_BTx623_147711
Zea_mays_B73_GRMZM2G016066_P01
Zea_mays_B73_GRMZM2G016622_P01
Oryza_sativa_Os07t0435300_01
Brachypodium_distachyon_Bradi1g29000_1
Ostreococcus_tauri_OTTH095_OTH95_RCC745-2001_15913
Ostreococcus_lucimarinus_CCE9901_36197
Micromonas_pusilla_CCMP1545_5901
Micromonas_commoda_RCC299_3659
Chlamydomonas_reinhardtii_Cre10.g420350.t1.2
Physcomitrella_patens_39045
Physcomitrella_patens_39042
Physcomitrella_patens_133111
Selaginella_moellendorffii_49753
Klebsormidium_flaccidum_kfl00057_0180
Cyanidioschyzon_merolae_Cp_psaE
Cyanothece_PCC7425_Cyan7425_4179
Thermosynechococcus_elongatus_tsl1567_psaE
Cyanothece_PCC7424_PCC7424_3608
Acaryochloris_marina_MBIC11017_AM1_2503_psaE
Cyanothece_PCC7425_Cyan7425_1271
Anabaena_variabilis_ATCC_29413_Ava_1270
Anabaena_sp._PCC_7120_asr4319_psaE
Nostoc_punctiforme_sp._PCC73102_Npun_R3937
Cyanophora_paradoxa_cp_psaE
Trichodesmium_erythraeum__405_1_Tery_1014
Galdieria_sulphuraria_Cp_psaE
Arthrospira__platensis_NIES_39_NIES39_M01060_psaE
Arthrospira_maxima_CS-328_003829
Thalassiosira_pseudonana_Cp_psaE
Phaeodactylum_tricornutum_Cp_PhtrCp071_psaE
Pyropia_yezoensis_Cp_144_psaE
Porphyridium_purpurea_Cp_psaE
Gracilaria_salicornia_Cp_psaE
Chondrus_crispus_Cp_CHC_420_psaE
Synechococcus_sp._PCC7002_SYNPCC7002_A1393_psaE
Cyanothece_PCC7424_PCC7424_5103
Cyanobacterium_Yellowstone_B-prime_CYB_1245_psaE
Cyanobacterium_Yellowstone_A-prime_CYA_0204_psaE
Microcystis_aeruginosa_NIES843_MAE_31800_psaE
Synechocystis_sp._PCC_6803_ssr2831_psaE
Cyanothece_PCC8802_Cyan8802_0128
Cyanothece_PCC8801_PCC8801_0131
Crocosphaera_watsonii_WH8501_g312
Cyanothece_ATCC51142_cce_4325_psaE
Cyanobacterium_UCYN_A_UCYN_02950
Synechococcus_sp._PCC_7942_Synpcc7942_1322
Synechococcus_sp._PCC_6301_syc0231_c_psaE
Synechococcus_sp._RCC307_SynRCC307_0451_psaE
Synechococcus_sp._9902_Syncc9902_1842
Synechococcus_sp._9605_Syncc9605_0493
Synechococcus_sp._WH8102_SYNW1960_psaE
Synechococcus_sp.WH5701_ZP01085866
Synechococcus_sp._CC9311_sync_0562_psaE
Synechocossus_sp._WH7803_SynWH7803_0540_psaE
Prochlorococcus_marinus_MIT9211_P9211_03651_psaE
Prochlorococcus_marinus_CCMP1375_Pro0371_psaE
Prochlorococcus_marinus_AS9601_A9601_03551_psaE
Prochlorococcus_marinus_MIT9301_P9301_03561_psaE
Prochlorococcus_marinus_MIT9312_PMT9312_0335
Prochlorococcus_marinus_CCMP_1986_PMM0329_psaE
Prochlorococcus_marinus_MIT9215_P9215_03551_psaE
Prochlorococcus_marinus_MIT9315_P9515_03611_psaE
Prochlorococcus_marinus_MIT9303_P9303_21751_psaE
Prochlorococcus_marinus_MIT9313_PMT0187_psaE
Synechococcus_sp._9902_Syncc9902_1441
Pseudo-nitzschia_multiseries_CLN-47_314458
Prochlorococcus_marinus_NATL1A_NATL1_04211_psaE
Prochlorococcus_marinus_NATL2A_PMN2A_1706
Gloeobacter_violaceus_gsl3408_psaE
Plants
Green algae
Glaucophyta
Diatoms
Red algae
Cyanobacteria
0.3

Set A53
(23 proteins of house-keeping proteins conserved in 53 organisms)
(BI)

AtpA, AtpB, AtpE, AtpH, AtpI, Rps2, Rps3, Rps4, Rps7, Rps8, Rps11, Rps12, Rps14, Rps18, Rps19, rpl2, Rpl14, Rpl16, Rpl20, Rpl23, rpoA, RpoB, RpoC (C1+C2)

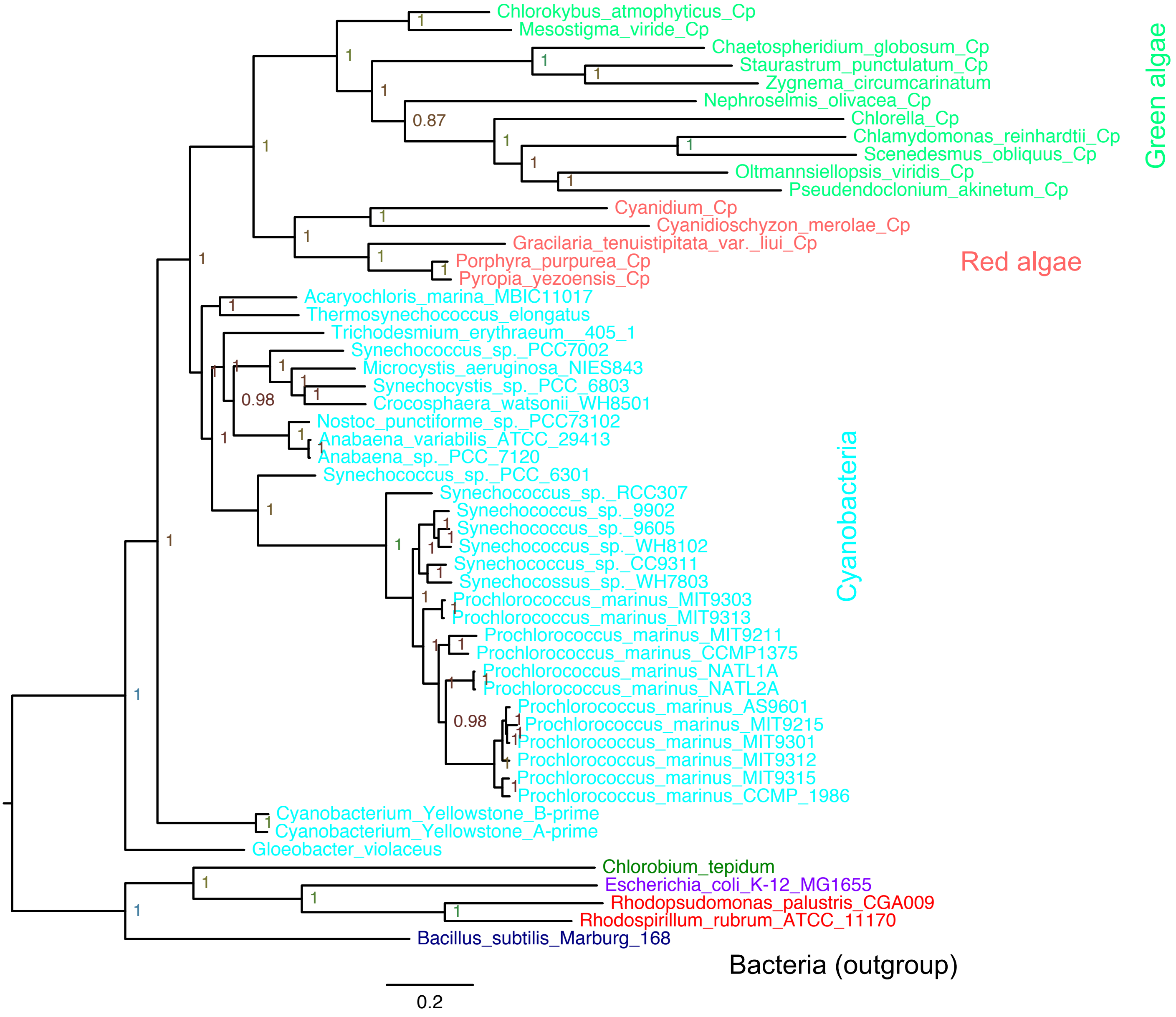

Set B42
(53 proteins conserved in
red algae and bacteria)
(BI)

Rps5, Rps6, Rps9, Rps10, Rps13, Rps16, Rps17, Rps20, Rpl1, Rpl3, Rpl4, Rpl5, Rpl6, Rpl11, Rpl12, Rpl13, Rpl18, Rpl19, Rpl21, Rpl22, Rpl24, Rpl27, Rpl28, Rpl29, Rpl31, Rpl32, Rpl33, Rpl34, Rpl35, AccA, AccB, AccD, AcpP, ArgB, AtpH, CarA, DnaB, DnaK, GltB, GroEL, IlvB, IlvH, InfC, Sds, SecA, SecY, TatC, ThiF, ThiG, TrpA, TrxA, Tsf, TufA

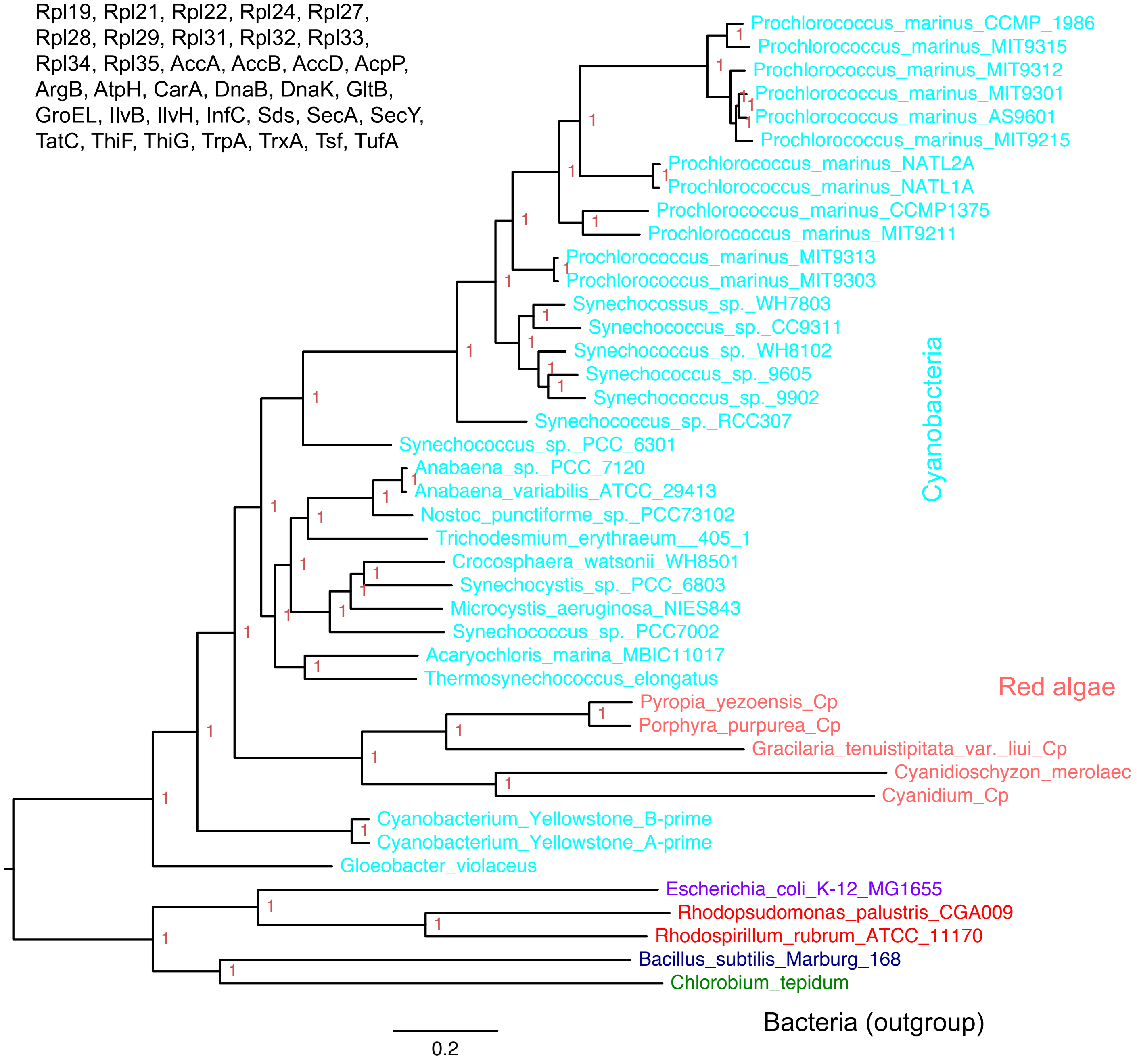

Set F35
(20 proteins conserved in
35 red macrophytes)
(BI)

Rpl9, Rps1, InfB, Rne, CcdA, ElaA, FabH, Ho1, NblS, PgmA, PheT, SecG, Upp, Ycf33, Ycf34, Ycf35, Ycf37, Ycf38, Ycf46, Ycf63

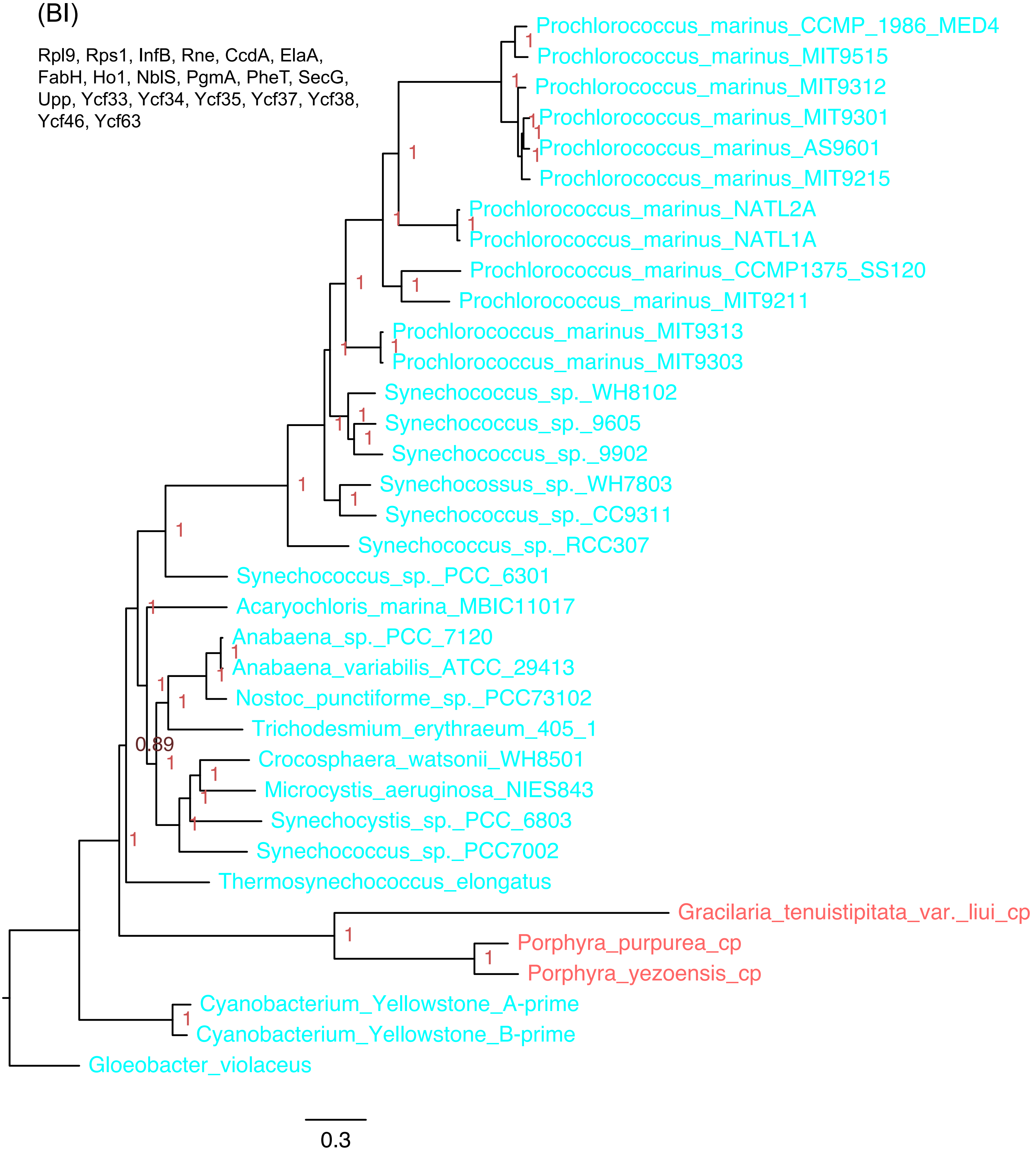

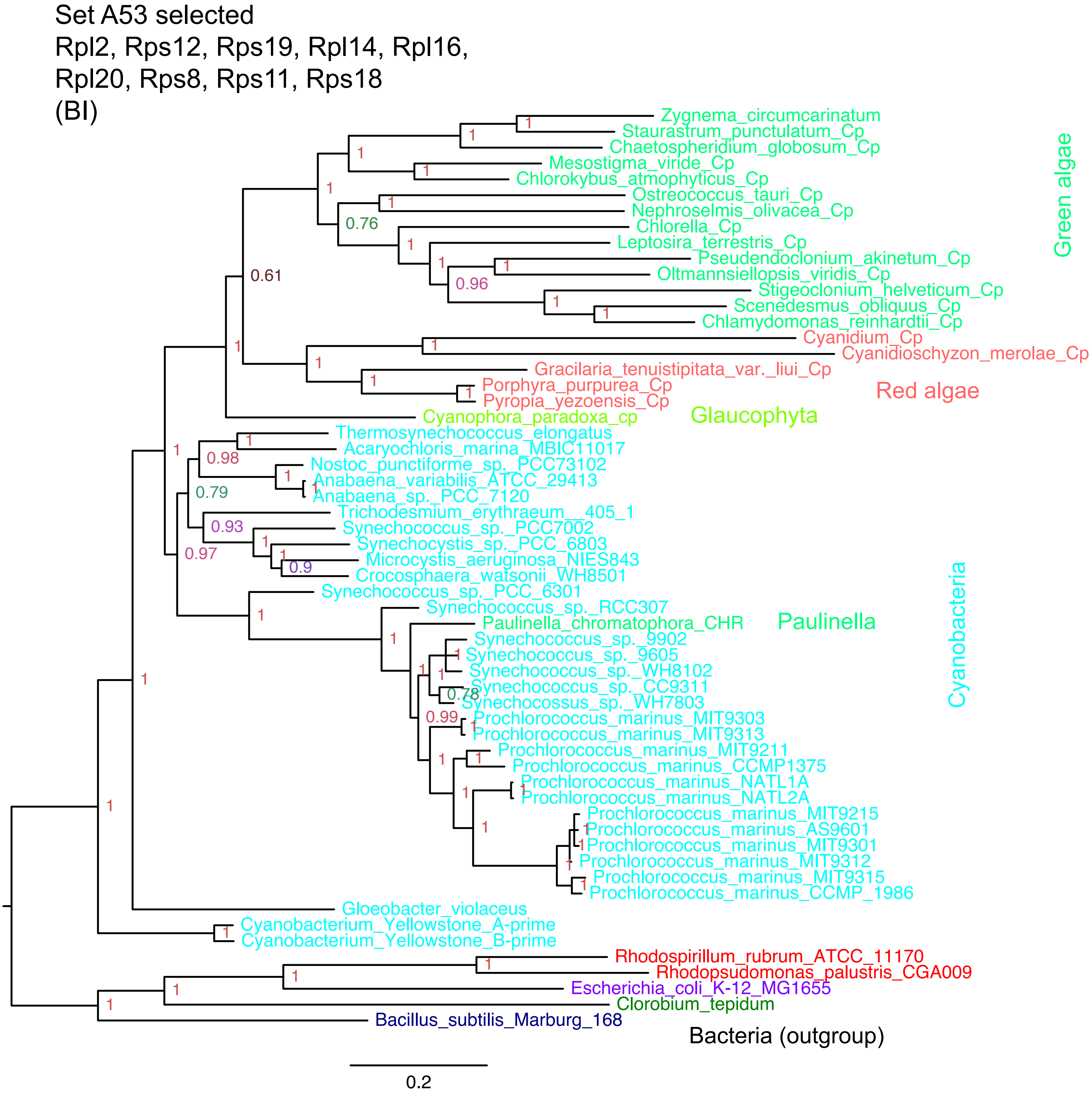
Set A53 selected
Rpl2, Rps12, Rps19, Rpl14, Rpl16,
Rpl20, Rps8, Rps11, Rps18
(BI)
Zygnema_circumcarinatum
Staurastrum_punctulatum_Cp
Chaetospheridium_globosum_Cp
Mesostigma_viride_Cp
Chlorokybus_atmophyticus_Cp
Ostreococcus_tauri_Cp
Nephroselmis_olivacea_Cp
Chlorella_Cp
Leptosira_terrestris_Cp
Pseudendoclonium_akinetum_Cp
Oltmannsiellopsis_viridis_Cp
Stigeoclonium_helveticum_Cp
Scenedesmus_obliquus_Cp
Chlamydomonas_reinhardtii_Cp
Green algae
Cyanidium_Cp
Cyanidioschyzon_merolae_Cp
Gracilaria_tenuistipitata_var._liui_Cp
Porphyra_purpurea_Cp
Pyropia_yezoensis_Cp
Red algae
Cyanophora_paradoxa_cp
Glaucophyta
Thermosynechococcus_elongatus
Acaryochloris_marina_MBIC11017
Nostoc_punctiforme_sp._PCC73102
Anabaena_variabilis_ATCC_29413
Anabaena_sp._PCC_7120
Trichodesmium_erythraeum__405_1
Synechococcus_sp._PCC7002
Synechocystis_sp._PCC_6803
Microcystis_aeruginosa_NIES843
Crocosphaera_watsonii_WH8501
Synechococcus_sp._PCC_6301
Synechococcus_sp._RCC307
Paulinella_chromatophora_CHR
Paulinella
Synechococcus_sp._9902
Synechococcus_sp._9605
Synechococcus_sp._WH8102
Synechococcus_sp._CC9311
Synechocossus_sp._WH7803
Prochlorococcus_marinus_MIT9303
Prochlorococcus_marinus_MIT9313
Prochlorococcus_marinus_MIT9211
Prochlorococcus_marinus_CCMP1375
Prochlorococcus_marinus_NATL1A
Prochlorococcus_marinus_NATL2A
Prochlorococcus_marinus_MIT9215
Prochlorococcus_marinus_AS9601
Prochlorococcus_marinus_MIT9301
Prochlorococcus_marinus_MIT9312
Prochlorococcus_marinus_MIT9315
Prochlorococcus_marinus_CCMP_1986
Cyanobacteria
Gloeobacter_violaceus
Cyanobacterium_Yellowstone_A-prime
Cyanobacterium_Yellowstone_B-prime
Rhodospirillum_rubrum_ATCC_11170
Rhodopsudomonas_palustris_CGA009
Escherichia_coli_K-12_MG1655
Clorobium_tepidum
Bacillus_subtilis_Marburg_168
Bacteria (outgroup)
0.2

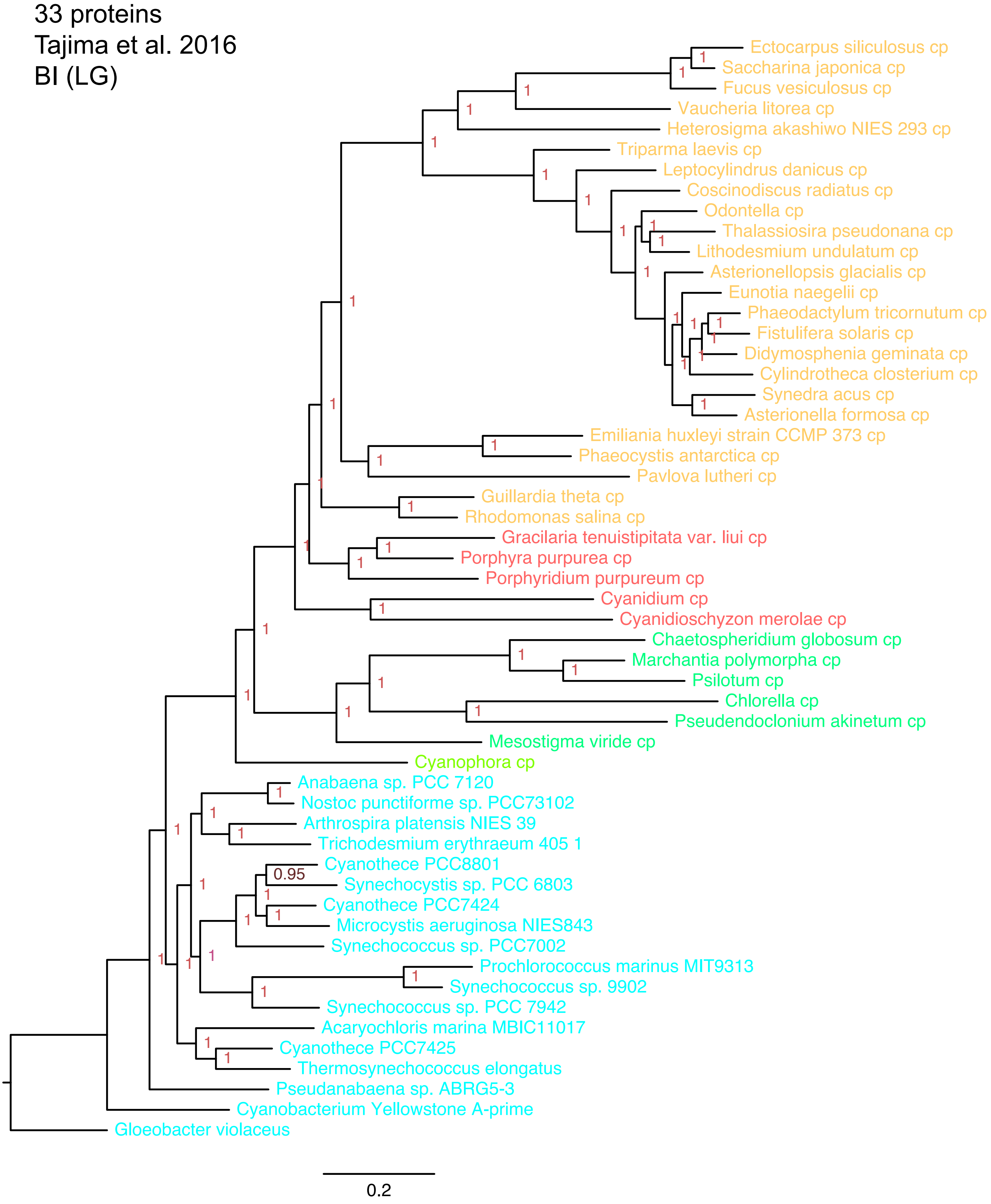
33 proteins
Tajima et al. 2016
BI (LG)
Ectocarpus siliculosus cp
Saccharina japonica cp
Fucus vesiculosus cp
Vaucheria litorea cp
Heterosigma akashiwo NIES 293 cp
Triparma laevis cp
Leptocylindrus danicus cp
Coscinodiscus radiatus cp
Odontella cp
Thalassiosira pseudonana cp
Lithodesmium undulatum cp
Asterionellopsis glacialis cp
Eunotia naegelii cp
Phaeodactylum tricornutum cp
Fistulifera solaris cp
Didymosphenia geminata cp
Cylindrotheca closterium cp
Synedra acus cp
Asterionella formosa cp
Emiliania huxleyi strain CCMP 373 cp
Phaeocystis antarctica cp
Pavlova lutheri cp
Guillardia theta cp
Rhodomonas salina cp
Gracilaria tenuistipitata var. liui cp
Porphyra purpurea cp
Porphyridium purpureum cp
Cyanidium cp
Cyanidioschyzon merolae cp
Chaetospheridium globosum cp
Marchantia polymorpha cp
Psilotum cp
Chlorella cp
Pseudendoclonium akinetum cp
Mesostigma viride cp
Cyanophora cp
Anabaena sp. PCC 7120
Nostoc punctiforme sp. PCC73102
Arthrospira platensis NIES 39
Trichodesmium erythraeum 405 1
Cyanothece PCC8801
0.95
Synechocystis sp. PCC 6803
Cyanothece PCC7424
Microcystis aeruginosa NIES843
Synechococcus sp. PCC7002
Prochlorococcus marinus MIT9313
Synechococcus sp. 9902
Synechococcus sp. PCC 7942
Acaryochloris marina MBIC11017
Cyanothece PCC7425
Thermosynechococcus elongatus
Pseudanabaena sp. ABRG5-3
Cyanobacterium Yellowstone A-prime
Gloeobacter violaceus
0.2

Set E48
ChlB, ChlL and ChlN
(BI/ML)

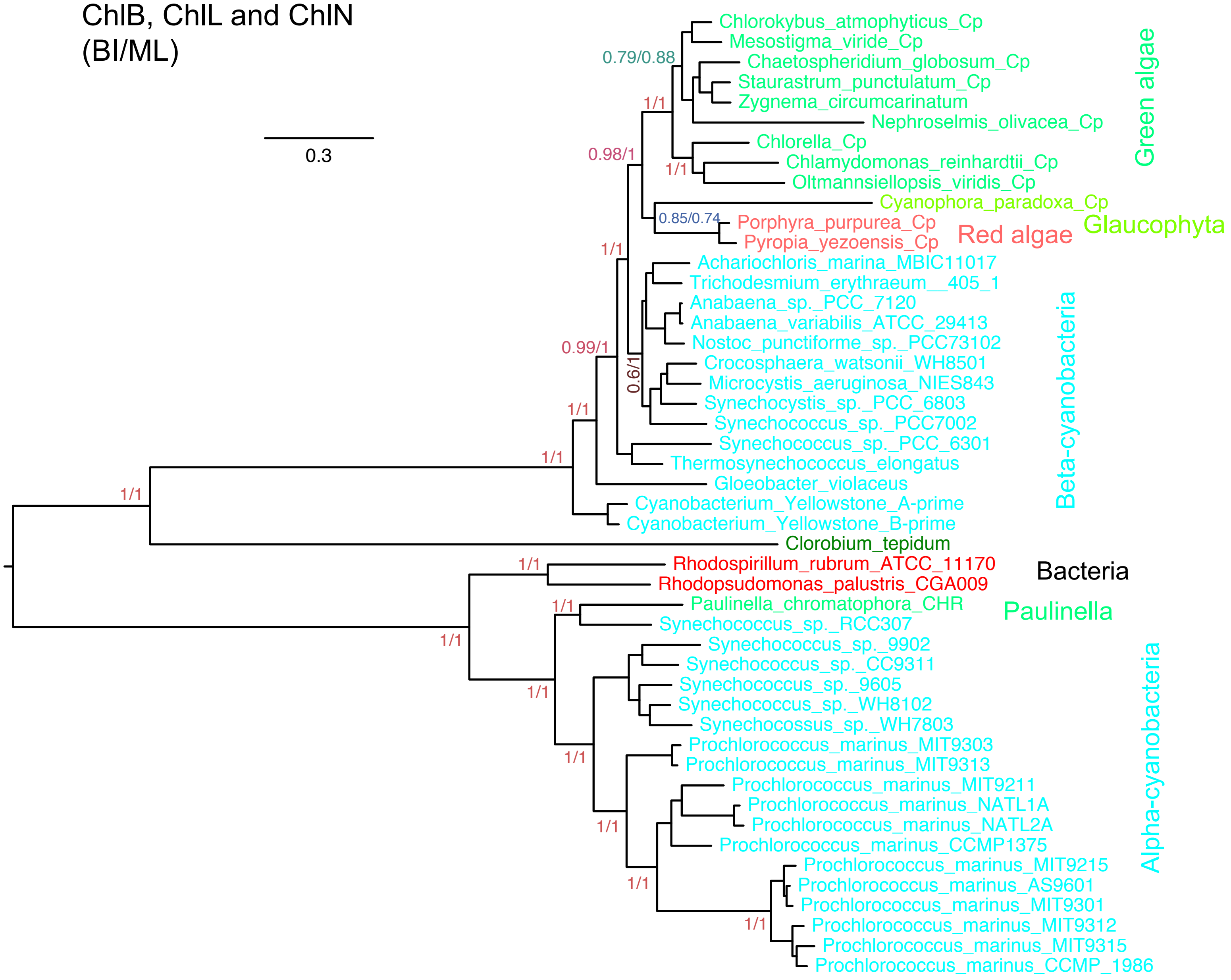

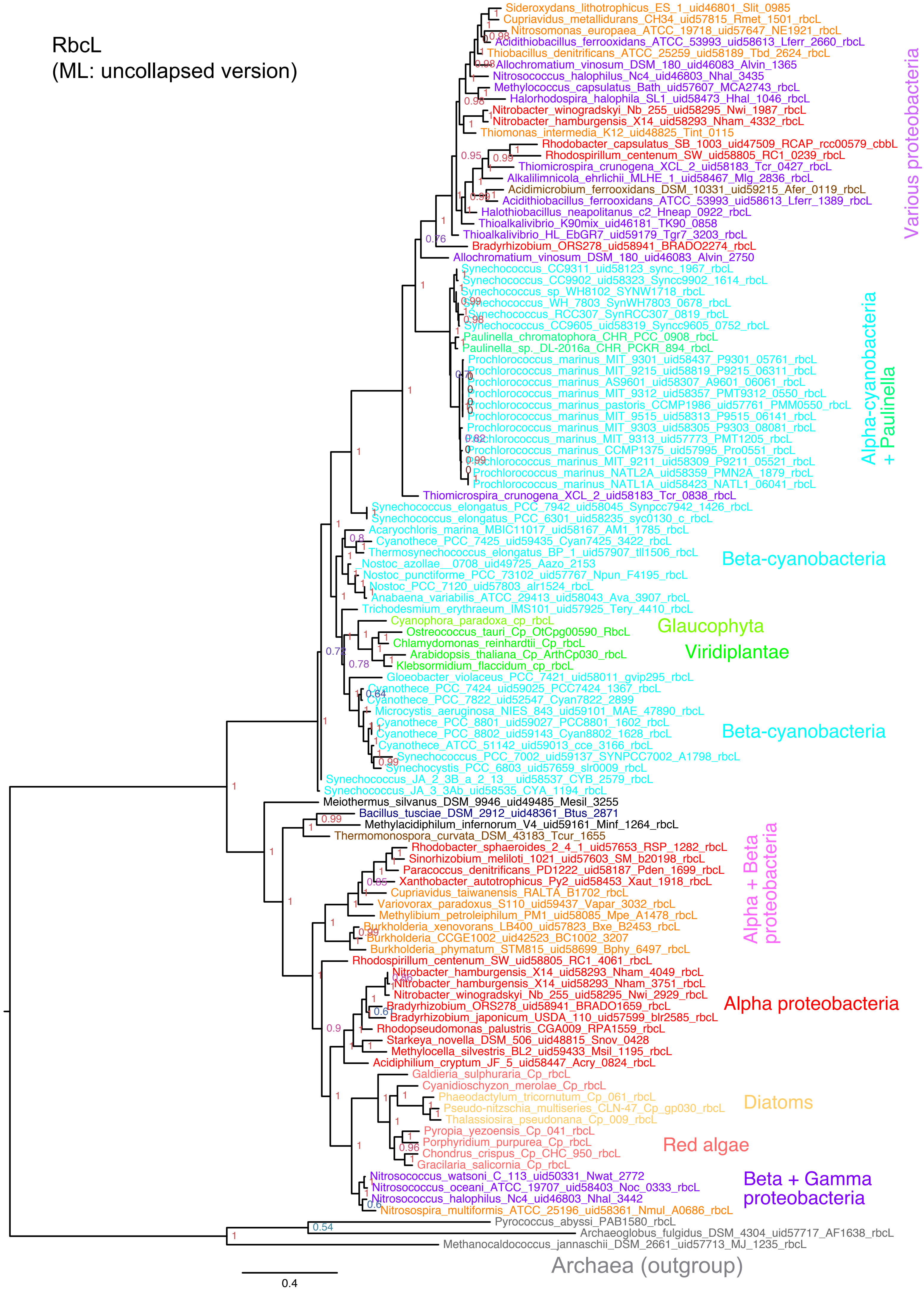
RbcL
(ML: uncollapsed version)
Sideroxydans_lithotrophicus_ES_1_uid46801_Slit_0985
Cupriavidus_metallidurans_CH34_uid57815_Rmet_1501_rbcL
Nitrosomonas_europaea_ATCC_19718_uid57647_NE1921_rbcL
Acidithiobacillus_ferrooxidans_ATCC_53993_uid58613_Lferr_2660_rbcL
Thiobacillus_denitrificans_ATCC_25259_uid58189_Tbd_2624_rbcL
Allochromatium_vinosum_DSM_180_uid46083_Alvin_1365
Nitrosococcus_halophilus_Nc4_uid46803_Nhal_3435
Methylococcus_capsulatus_Bath_uid57607_MCA2743_rbcL
Halorhodospira_halophila_SL1_uid58473_Hhal_1046_rbcL
Nitrobacter_winogradskyi_Nb_255_uid58295_Nwi_1987_rbcL
Nitrobacter_hamburgensis_X14_uid58293_Nham_4332_rbcL
Thiomonas_intermedia_K12_uid48825_Tint_0115
Rhodobacter_capsulatus_SB_1003_uid47509_RCAP_rcc00579_cbbL
Rhodospirillum_centenum_SW_uid58805_RC1_0239_rbcL
Thiomicrospira_crunogena_XCL_2_uid58183_Tcr_0427_rbcL
Alkalilimnicola_ehrlichii_MLHE_1_uid58467_Mlg_2836_rbcL
Acidimicrobium_ferrooxidans_DSM_10331_uid59215_Afer_0119_rbcL
Acidithiobacillus_ferrooxidans_ATCC_53993_uid58613_Lferr_1389_rbcL
Halothiobacillus_neapolitanus_c2_Hneap_0922_rbcL
Thioalkalivibrio_K90mix_uid46181_TK90_0858
Thioalkalivibrio_HL_EbGR7_uid59179_Tgr7_3203_rbcL
Bradyrhizobium_ORS278_uid58941_BRADO2274_rbcL
Allochromatium_vinosum_DSM_180_uid46083_Alvin_2750
Various proteobacteria
Synechococcus_CC9311_uid58123_sync_1967_rbcL
Synechococcus_CC9902_uid58323_Syncc9902_1614_rbcL
Synechococcus_sp_WH8102_SYNW1718_rbcL
Synechococcus_WH_7803_SynWH7803_0678_rbcL
Synechococcus_RCC307_SynRCC307_0819_rbcL
Synechococcus_CC9605_uid58319_Syncc9605_0752_rbcL
Paulinella_chromatophora_CHR_PCC_0908_rbcL
Paulinella_sp._DL-2016a_CHR_PCKR_894_rbcL
Prochlorococcus_marinus_MIT_9301_uid58437_P9301_05761_rbcL
Prochlorococcus_marinus_MIT_9215_uid58819_P9215_06311_rbcL
Prochlorococcus_marinus_AS9601_uid58307_A9601_06061_rbcL
Prochlorococcus_marinus_MIT_9312_uid58357_PMT9312_0550_rbcL
Prochlorococcus_marinus_pastoris_CCMP1986_uid57761_PMM0550_rbcL
Prochlorococcus_marinus_MIT_9515_uid58313_P9515_06141_rbcL
Prochlorococcus_marinus_MIT_9303_uid58305_P9303_08081_rbcL
Prochlorococcus_marinus_MIT_9313_uid57773_PMT1205_rbcL
Prochlorococcus_marinus_CCMP1375_uid57995_Pro0551_rbcL
Prochlorococcus_marinus_MIT_9211_uid58309_P9211_05521_rbcL
Prochlorococcus_marinus_NATL2A_uid58359_PMN2A_1879_rbcL
Prochlorococcus_marinus_NATL1A_uid58423_NATL1_06041_rbcL
Alpha-cyanobacteria
+ Paulinella
Thiomicrospira_crunogena_XCL_2_uid58183_Tcr_0838_rbcL
Synechococcus_elongatus_PCC_7942_uid58045_Synpcc7942_1426_rbcL
Synechococcus_elongatus_PCC_6301_uid58235_syc0130_c_rbcL
Acaryochloris_marina_MBIC11017_uid58167_AM1_1785_rbcL
Cyanothece_PCC_7425_uid59435_Cyan7425_3422_rbcL
Thermosynechococcus_elongatus_BP_1_uid57907_tll1506_rbcL
Nostoc_azollae__0708_uid49725_Aazo_2153
Nostoc_punctiforme_PCC_73102_uid57767_Npun_F4195_rbcL
Nostoc_PCC_7120_uid57803_alr1524_rbcL
Anabaena_variabilis_ATCC_29413_uid58043_Ava_3907_rbcL
Trichodesmium_erythraeum_IMS101_uid57925_Tery_4410_rbcL
Beta-cyanobacteria
Cyanophora_paradoxa_cp_rbcL
Glaucophyta
Ostreococcus_tauri_Cp_OtCpg00590_RbcL
Chlamydomonas_reinhardtii_Cp_rbcL
Arabidopsis_thaliana_Cp_ArthCp030_rbcL
Klebsormidium_flaccidum_cp_rbcL
Viridiplantae
Gloeobacter_violaceus_PCC_7421_uid58011_gvip295_rbcL
Cyanothece_PCC_7424_uid59025_PCC7424_1367_rbcL
Cyanothece_PCC_7822_uid52547_Cyan7822_2899
Microcystis_aeruginosa_NIES_843_uid59101_MAE_47890_rbcL
Cyanothece_PCC_8801_uid59027_PCC8801_1602_rbcL
Cyanothece_PCC_8802_uid59143_Cyan8802_1628_rbcL
Cyanothece_ATCC_51142_uid59013_cce_3166_rbcL
Synechococcus_PCC_7002_uid59137_SYNPCC7002_A1798_rbcL
Synechocystis_PCC_6803_uid57659_slr0009_rbcL
Beta-cyanobacteria
Synechococcus_JA_2_3B_a_2_13__uid58537_CYB_2579_rbcL
Synechococcus_JA_3_3Ab_uid58535_CYA_1194_rbcL
Meiothermus_silvanus_DSM_9946_uid49485_Mesil_3255
Bacillus_tusciae_DSM_2912_uid48361_Btus_2871
Methylacidiphilum_infernorum_V4_uid59161_Minf_1264_rbcL
Thermomonospora_curvata_DSM_43183_Tcur_1655
Rhodobacter_sphaeroides_2_4_1_uid57653_RSP_1282_rbcL
Sinorhizobium_meliloti_1021_uid57603_SM_b20198_rbcL
Paracoccus_denitrificans_PD1222_uid58187_Pden_1699_rbcL
Xanthobacter_autotrophicus_Py2_uid58453_Xaut_1918_rbcL
Cupriavidus_taiwanensis_RALTA_B1702_rbcL
Variovorax_paradoxus_S110_uid59437_Vapar_3032_rbcL
Methylibium_petroleiphilum_PM1_uid58085_Mpe_A1478_rbcL
Burkholderia_xenovorans_LB400_uid57823_Bxe_B2453_rbcL
Burkholderia_CCGE1002_uid42523_BC1002_3207
Burkholderia_phymatum_STM815_uid58699_Bphy_6497_rbcL
Alpha + Beta
proteobacteria
Rhodospirillum_centenum_SW_uid58805_RC1_4061_rbcL
Nitrobacter_hamburgensis_X14_uid58293_Nham_4049_rbcL
Nitrobacter_hamburgensis_X14_uid58293_Nham_3751_rbcL
Nitrobacter_winogradskyi_Nb_255_uid58295_Nwi_2929_rbcL
Bradyrhizobium_ORS278_uid58941_BRADO1659_rbcL
Bradyrhizobium_japonicum_USDA_110_uid57599_blr2585_rbcL
Rhodopseudomonas_palustris_CGA009_RPA1559_rbcL
Starkeya_novella_DSM_506_uid48815_Snov_0428
Methylocella_silvestris_BL2_uid59433_Msil_1195_rbcL
Acidiphilium_cryptum_JF_5_uid58447_Acry_0824_rbcL
Alpha proteobacteria
Galdieria_sulphuraria_Cp_rbcL
Cyanidioschyzon_merolae_Cp_rbcL
Phaeodactylum_tricornutum_Cp_061_rbcL
Pseudo-nitzschia_multiseries_CLN-47_Cp_gp030_rbcL
Thalassiosira_pseudonana_Cp_009_rbcL
Diatoms
Pyropia_yezoensis_Cp_041_rbcL
Porphyridium_purpurea_Cp_rbcL
Chondrus_crispus_Cp_CHC_950_rbcL
Gracilaria_salicornia_Cp_rbcL
Red algae
Nitrosococcus_watsoni_C_113_uid50331_Nwat_2772
Nitrosococcus_oceani_ATCC_19707_uid58403_Noc_0333_rbcL
Nitrosococcus_halophilus_Nc4_uid46803_Nhal_3442
Nitrosospira_multiformis_ATCC_25196_uid58361_Nmul_A0686_rbcL
Beta + Gamma
proteobacteria
Pyrococcus_abyssi_PAB1580_rbcL
Archaeoglobus_fulgidus_DSM_4304_uid57717_AF1638_rbcL
Methanocaldococcus_jannaschii_DSM_2661_uid57713_MJ_1235_rbcL
Archaea (outgroup)
0.4

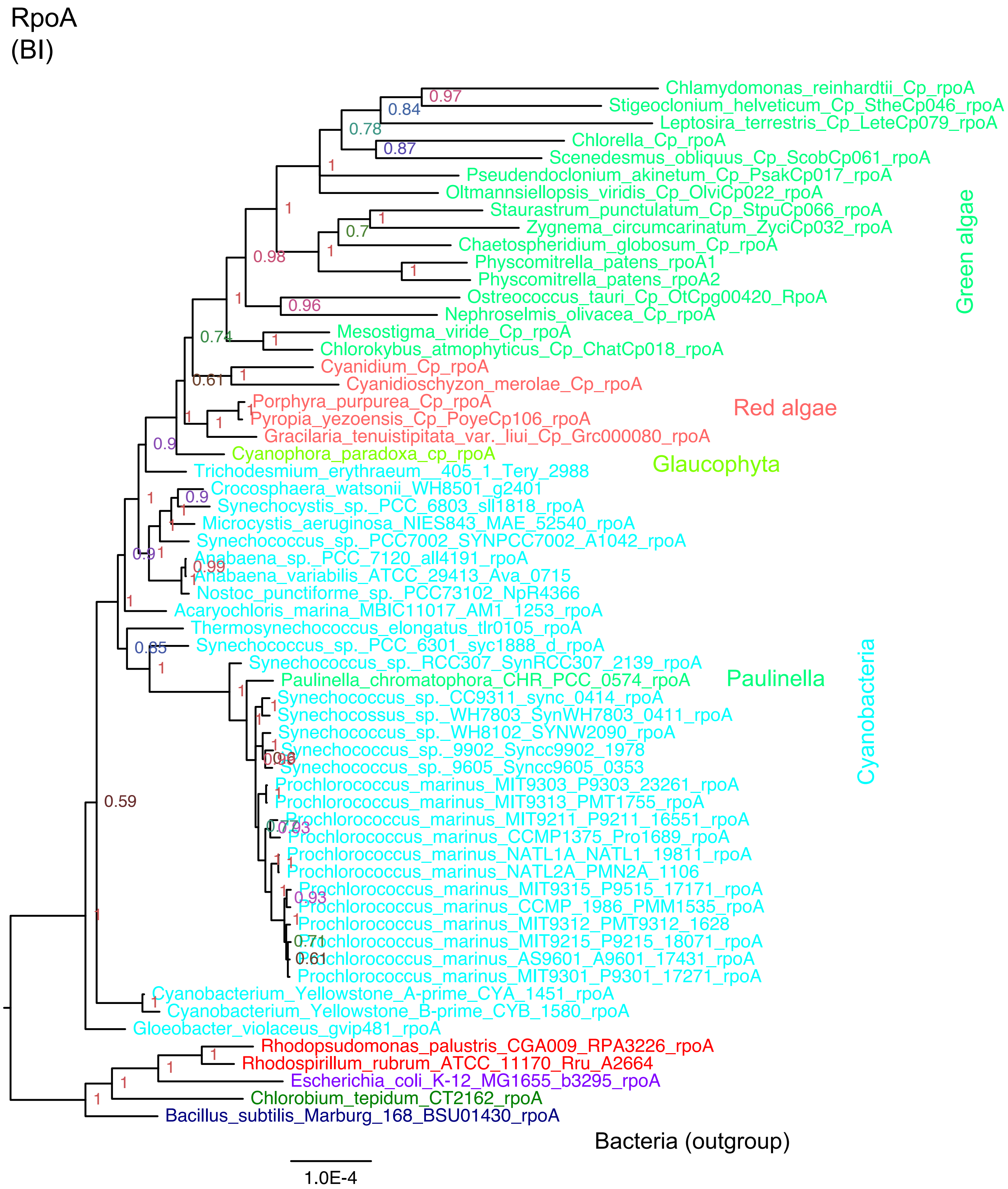

RpoA
(BI)
Chlamydomonas_reinhardtii_Cp_rpoA
Stigeoclonium_helveticum_Cp_StheCp046_rpoA
Leptosira_terrestris_Cp_LeteCp079_rpoA
Chlorella_Cp_rpoA
Scenedesmus_obliquus_Cp_ScobCp061_rpoA
Pseudendoclonium_akinetum_Cp_PsakCp017_rpoA
Oltmannsiellopsis_viridis_Cp_OlviCp022_rpoA
Staurastrum_punctulatum_Cp_StpuCp066_rpoA
Zygnema_circumcarinatum_ZyciCp032_rpoA
Chaetospheridium_globosum_Cp_rpoA
Physcomitrella_patens_rpoA1
Physcomitrella_patens_rpoA2
Ostreococcus_tauri_Cp_OtCpg00420_RpoA
Nephroselmis_olivacea_Cp_rpoA
Mesostigma_viride_Cp_rpoA
Chlorokybus_atmophyticus_Cp_ChatCp018_rpoA
Cyanidium_Cp_rpoA
Cyanidioschyzon_merolae_Cp_rpoA
Porphyra_purpurea_Cp_rpoA
Pyropia_yezoensis_Cp_PoyeCp106_rpoA
Gracilaria_tenuistipitata_var._liui_Cp_Grc000080_rpoA
Cyanophora_paradoxa_cp_rpoA
Trichodesmium_erythraeum__405_1_Tery_2988
Crocosphaera_watsonii_WH8501_g2401
Synechocystis_sp._PCC_6803_sll1818_rpoA
Microcystis_aeruginosa_NIES843_MAE_52540_rpoA
Synechococcus_sp._PCC7002_SYNPCC7002_A1042_rpoA
Anabaena_sp._PCC_7120_all4191_rpoA
Anabaena_variabilis_ATCC_29413_Ava_0715
Nostoc_punctiforme_sp._PCC73102_NpR4366
Acaryochloris_marina_MBIC11017_AM1_1253_rpoA
Thermosynechococcus_elongatus_tlr0105_rpoA
Synechococcus_sp._PCC_6301_syc1888_d_rpoA
Synechococcus_sp._RCC307_SynRCC307_2139_rpoA
Paulinella_chromatophora_CHR_PCC_0574_rpoA
Synechococcus_sp._CC9311_sync_0414_rpoA
Synechocossus_sp._WH7803_SynWH7803_0411_rpoA
Synechococcus_sp._WH8102_SYNW2090_rpoA
Synechococcus_sp._9902_Syncc9902_1978
Synechococcus_sp._9605_Syncc9605_0353
Prochlorococcus_marinus_MIT9303_P9303_23261_rpoA
Prochlorococcus_marinus_MIT9313_PMT1755_rpoA
Prochlorococcus_marinus_MIT9211_P9211_16551_rpoA
Prochlorococcus_marinus_CCMP1375_Pro1689_rpoA
Prochlorococcus_marinus_NATL1A_NATL1_19811_rpoA
Prochlorococcus_marinus_NATL2A_PMN2A_1106
Prochlorococcus_marinus_MIT9315_P9515_17171_rpoA
Prochlorococcus_marinus_CCMP_1986_PMM1535_rpoA
Prochlorococcus_marinus_MIT9312_PMT9312_1628
Prochlorococcus_marinus_MIT9215_P9215_18071_rpoA
Prochlorococcus_marinus_AS9601_A9601_17431_rpoA
Prochlorococcus_marinus_MIT9301_P9301_17271_rpoA
Cyanobacterium_Yellowstone_A-prime_CYA_1451_rpoA
Cyanobacterium_Yellowstone_B-prime_CYB_1580_rpoA
Gloeobacter_violaceus_gvip481_rpoA
Rhodopsudomonas_palustris_CGA009_RPA3226_rpoA
Rhodospirillum_rubrum_ATCC_11170_Rru_A2664
Escherichia_coli_K-12_MG1655_b3295_rpoA
Chlorobium_tepidum_CT2162_rpoA
Bacillus_subtilis_Marburg_168_BSU01430_rpoA
Green algae
Red algae
Glaucophyta
Paulinella
Cyanobacteria
Bacteria (outgroup)
1.0E-4

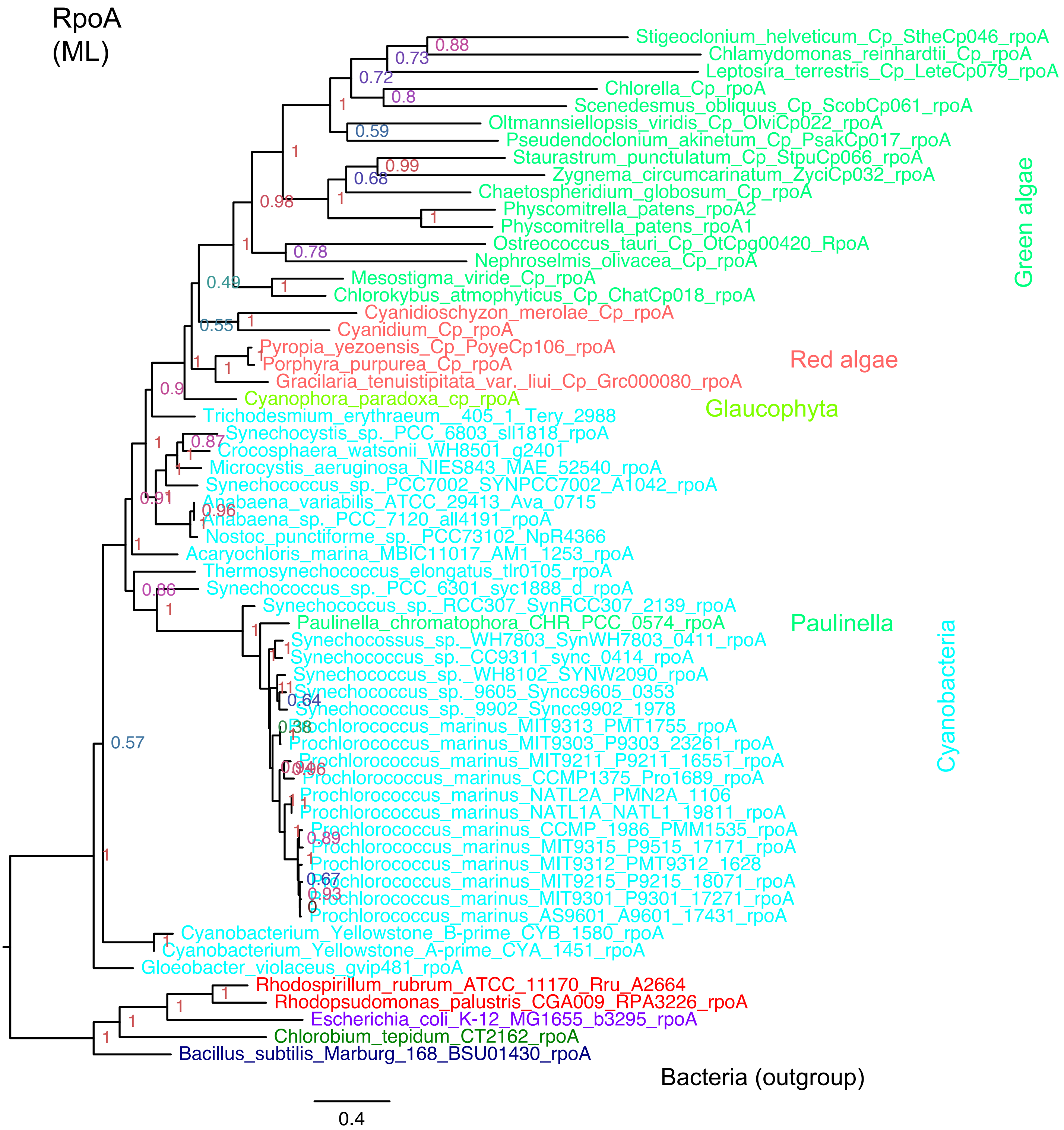
RpoA
(ML)
Stigeoclonium_helveticum_Cp_StheCp046_rpoA
Chlamydomonas_reinhardtii_Cp_rpoA
Leptosira_terrestris_Cp_LeteCp079_rpoA
Chlorella_Cp_rpoA
Scenedesmus_obliquus_Cp_ScobCp061_rpoA
Oltmannsiellopsis_viridis_Cp_OlviCp022_rpoA
Pseudendoclonium_akinetum_Cp_PsakCp017_rpoA
Staurastrum_punctulatum_Cp_StpuCp066_rpoA
Zygnema_circumcarinatum_ZyciCp032_rpoA
Chaetosphaeridium_globosum_Cp_rpoA
Physcomitrella_patens_rpoA2
Physcomitrella_patens_rpoA1
Ostreococcus_tauri_Cp_OtCpg00420_RpoA
Nephroselmis_olivacea_Cp_rpoA
Mesostigma_viride_Cp_rpoA
Chlorokybus_atmophyticus_Cp_ChatCp018_rpoA
Green algae
Cyanidioschyzon_merolae_Cp_rpoA
Cyanidium_Cp_rpoA
Pyropia_yezoensis_Cp_PoyeCp106_rpoA
Porphyra_purpurea_Cp_rpoA
Gracilaria_tenuistipitata_var._liui_Cp_Grc000080_rpoA
Red algae
Cyanophora_paradoxa_cp_rpoA
Glaucophyta
Trichodesmium_erythraeum__405_1_Tery_2988
Synechocystis_sp._PCC_6803_sll1818_rpoA
Crocosphaera_watsonii_WH8501_g2401
Microcystis_aeruginosa_NIES843_MAE_52540_rpoA
Synechococcus_sp._PCC7002_SYNPCC7002_A1042_rpoA
Anabaena_variabilis_ATCC_29413_Ava_0715
Anabaena_sp._PCC_7120_all4191_rpoA
Nostoc_punctiforme_sp._PCC73102_NpR4366
Acaryochloris_marina_MBIC11017_AM1_1253_rpoA
Thermosynechococcus_elongatus_tlr0105_rpoA
Synechococcus_sp._PCC_6301_syc1888_d_rpoA
Synechococcus_sp._RCC307_SynRCC307_2139_rpoA
Paulinella_chromatophora_CHR_PCC_0574_rpoA
Paulinella
Synechocossus_sp._WH7803_SynWH7803_0411_rpoA
Synechococcus_sp._CC9311_sync_0414_rpoA
Synechococcus_sp._WH8102_SYNW2090_rpoA
Synechococcus_sp._9605_Syncc9605_0353
Synechococcus_sp._9902_Syncc9902_1978
Prochlorococcus_marinus_MIT9313_PMT1755_rpoA
Prochlorococcus_marinus_MIT9303_P9303_23261_rpoA
Prochlorococcus_marinus_MIT9211_P9211_16551_rpoA
Prochlorococcus_marinus_CCMP1375_Pro1689_rpoA
Prochlorococcus_marinus_NATL2A_PMN2A_1106
Prochlorococcus_marinus_NATL1A_NATL1_19811_rpoA
Prochlorococcus_marinus_CCMP_1986_PMM1535_rpoA
Prochlorococcus_marinus_MIT9315_P9515_17171_rpoA
Prochlorococcus_marinus_MIT9312_PMT9312_1628
Prochlorococcus_marinus_MIT9215_P9215_18071_rpoA
Prochlorococcus_marinus_MIT9301_P9301_17271_rpoA
Prochlorococcus_marinus_AS9601_A9601_17431_rpoA
Cyanobacterium_Yellowstone_B-prime_CYB_1580_rpoA
Cyanobacterium_Yellowstone_A-prime_CYA_1451_rpoA
Gloeobacter_violaceus_gvip481_rpoA
Cyanobacteria
Rhodospirillum_rubrum_ATCC_11170_Rru_A2664
Rhodopsudomonas_palustris_CGA009_RPA3226_rpoA
Escherichia_coli_K-12_MG1655_b3295_rpoA
Chlorobium_tepidum_CT2162_rpoA
Bacillus_subtilis_Marburg_168_BSU01430_rpoA
Bacteria (outgroup)
0.4

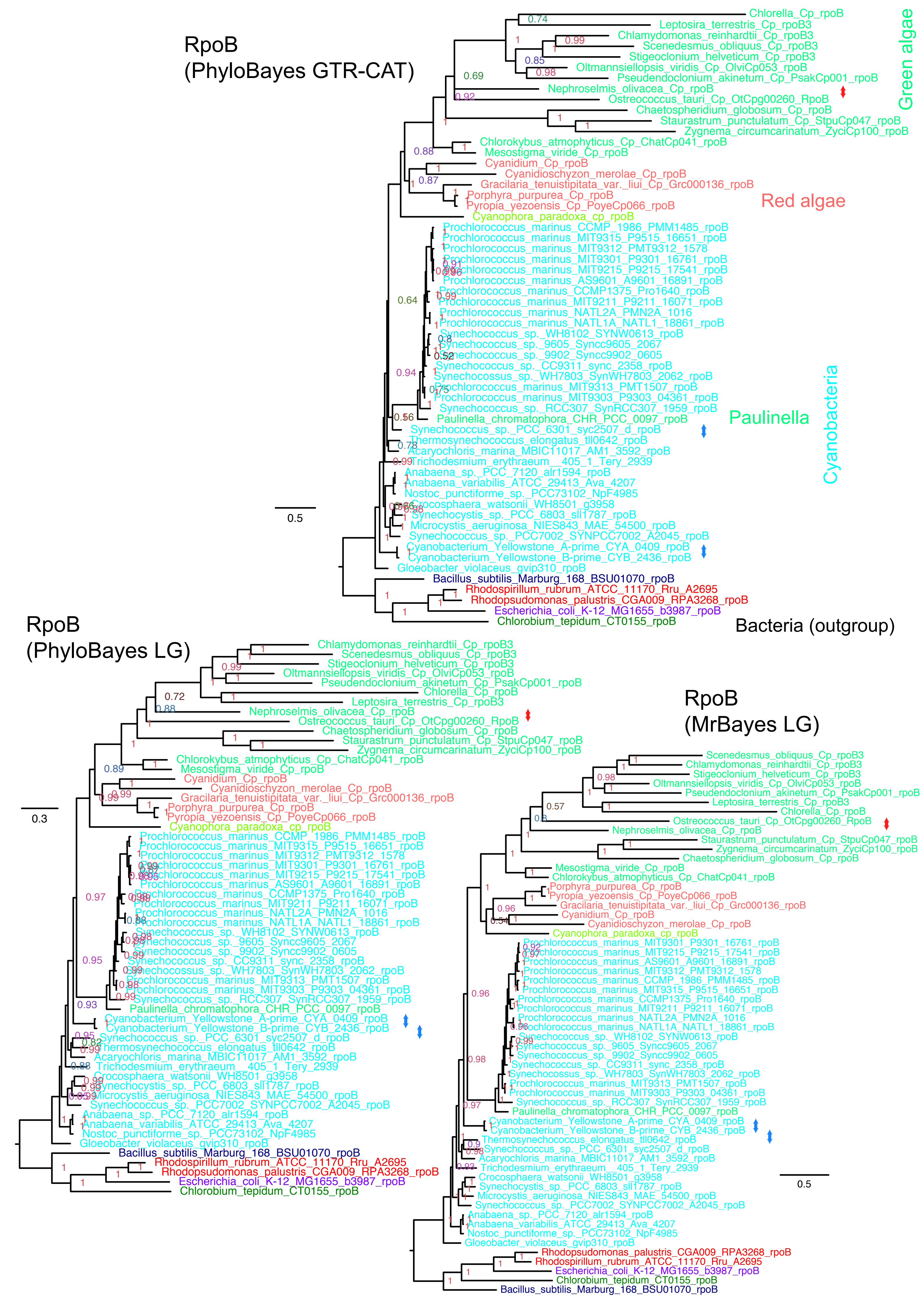
RpoB
(PhyloBayes GTR-CAT)
Chlorella_Cp_rpoB
Leptosira_terrestris_Cp_rpoB3
Chlamydomonas_reinhardtii_Cp_rpoB3
Scenedesmus_obliquus_Cp_rpoB3
Stigeoclonium_helveticum_Cp_rpoB3
Oltmannsiellopsis_viridis_Cp_OlviCp053_rpoB
Pseudendoclonium_akinetum_Cp_PsakCp001_rpoB
Nephroselmis_olivacea_Cp_rpoB
Ostreococcus_tauri_Cp_OtCpg00260_RpoB
Chaetospheridium_globosum_Cp_rpoB
Staurastrum_punctulatum_Cp_StpuCp047_rpoB
Zygnema_circumcarinatum_ZyciCp100_rpoB
Chlorokybus_atmophyticus_Cp_ChatCp041_rpoB
Mesostigma_viride_Cp_rpoB
Cyanidium_Cp_rpoB
Cyanidioschyzon_merolae_Cp_rpoB
Gracilaria_tenuistipitata_var._liui_Cp_Grc000136_rpoB
Porphyra_purpurea_Cp_rpoB
Pyropia_yezoensis_Cp_PoyeCp066_rpoB
Cyanophora_paradoxa_cp_rpoB
Green algae
Red algae
Prochlorococcus_marinus_CCMP_1986_PMM1485_rpoB
Prochlorococcus_marinus_MIT9315_P9515_16651_rpoB
Prochlorococcus_marinus_MIT9312_PMT9312_1578
Prochlorococcus_marinus_MIT9301_P9301_16761_rpoB
Prochlorococcus_marinus_MIT9215_P9215_17541_rpoB
Prochlorococcus_marinus_AS9601_A9601_16891_rpoB
Prochlorococcus_marinus_CCMP1375_Pro1640_rpoB
Prochlorococcus_marinus_MIT9211_P9211_16071_rpoB
Prochlorococcus_marinus_NATL2A_PMN2A_1016
Prochlorococcus_marinus_NATL1A_NATL1_18861_rpoB
Synechococcus_sp._WH8102_SYNW0613_rpoB
Synechococcus_sp._9605_Syncc9605_2067
Synechococcus_sp._9902_Syncc9902_0605
Synechococcus_sp._CC9311_sync_2358_rpoB
Synechocossus_sp._WH7803_SynWH7803_2062_rpoB
Prochlorococcus_marinus_MIT9313_PMT1507_rpoB
Prochlorococcus_marinus_MIT9303_P9303_04361_rpoB
Synechococcus_sp._RCC307_SynRCC307_1959_rpoB
Paulinella_chromatophora_CHR_PCC_0097_rpoB
Synechococcus_sp._PCC_6301_syc2507_d_rpoB
Thermosynechococcus_elongatus_tll0642_rpoB
Acaryochloris_marina_MBIC11017_AM1_3592_rpoB
Trichodesmium_erythraeum__405_1_Tery_2939
Anabaena_sp._PCC_7120_alr1594_rpoB
Anabaena_variabilis_ATCC_29413_Ava_4207
Nostoc_punctiforme_sp._PCC73102_NpF4985
Crocosphaera_watsonii_WH8501_g3958
Synechocystis_sp._PCC_6803_sll1787_rpoB
Microcystis_aeruginosa_NIES843_MAE_54500_rpoB
Synechococcus_sp._PCC7002_SYNPCC7002_A2045_rpoB
Cyanobacterium_Yellowstone_A-prime_CYA_0409_rpoB
Cyanobacterium_Yellowstone_B-prime_CYB_2436_rpoB
Gloeobacter_violaceus_gvip310_rpoB
Paulinella
Cyanobacteria
0.5
Bacillus_subtilis_Marburg_168_BSU01070_rpoB
Rhodospirillum_rubrum_ATCC_11170_Rru_A2695
Rhodopsudomonas_palustris_CGA009_RPA3268_rpoB
Escherichia_coli_K-12_MG1655_b3987_rpoB
Chlorobium_tepidum_CT0155_rpoB
Bacteria (outgroup)
RpoB
(PhyloBayes LG)
0.3
RpoB
(MrBayes LG)
0.5

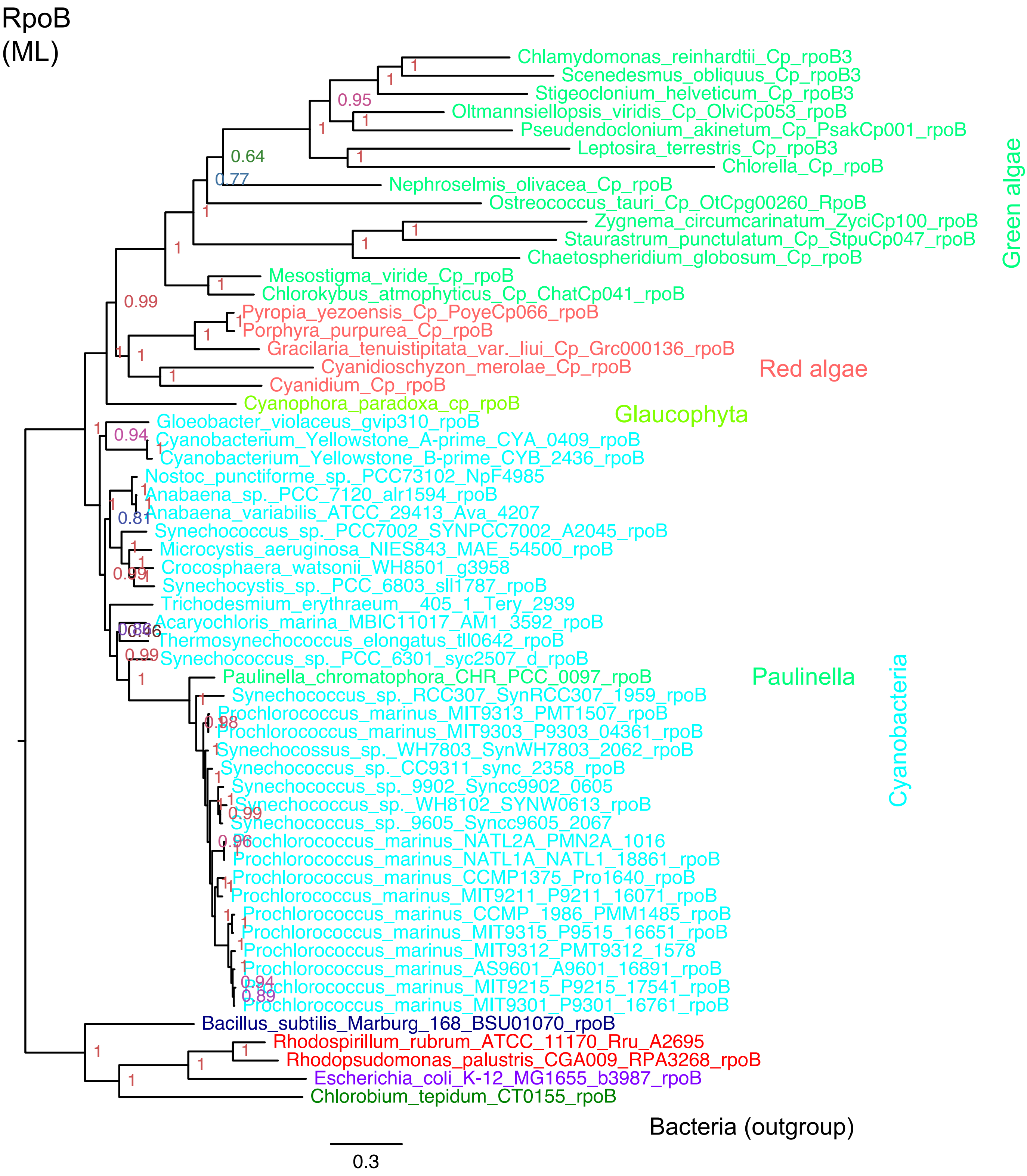

RpoB
(ML)
Chlamydomonas_reinhardtii_Cp_rpoB3
Scenedesmus_obliquus_Cp_rpoB3
Stigeoclonium_helveticum_Cp_rpoB3
Oltmannsiellopsis_viridis_Cp_OlviCp053_rpoB
Pseudendoclonium_akinetum_Cp_PsakCp001_rpoB
Leptosira_terrestris_Cp_rpoB3
Chlorella_Cp_rpoB
Nephroselmis_olivacea_Cp_rpoB
Ostreococcus_tauri_Cp_OtCpg00260_RpoB
Zygnema_circumcarinatum_ZyciCp100_rpoB
Staurastrum_punctulatum_Cp_StpuCp047_rpoB
Chaetosphaeridium_globosum_Cp_rpoB
Mesostigma_viride_Cp_rpoB
Chlorokybus_atmophyticus_Cp_ChatCp041_rpoB
Green algae
Pyropia_yezoensis_Cp_PoyeCp066_rpoB
Porphyra_purpurea_Cp_rpoB
Gracilaria_tenuistipitata_var._liui_Cp_Grc000136_rpoB
Cyanidioschyzon_merolae_Cp_rpoB
Cyanidium_Cp_rpoB
Red algae
Cyanophora_paradoxa_cp_rpoB
Glaucophyta
Gloeobacter_violaceus_gvip310_rpoB
Cyanobacterium_Yellowstone_A-prime_CYA_0409_rpoB
Cyanobacterium_Yellowstone_B-prime_CYB_2436_rpoB
Nostoc_punctiforme_sp._PCC73102_NpF4985
Anabaena_sp._PCC_7120_alr1594_rpoB
Anabaena_variabilis_ATCC_29413_Ava_4207
Synechococcus_sp._PCC7002_SYNPCC7002_A2045_rpoB
Microcystis_aeruginosa_NIES843_MAE_54500_rpoB
Crocosphaera_watsonii_WH8501_g3958
Synechocystis_sp._PCC_6803_sll1787_rpoB
Trichodesmium_erythraeum__405_1_Tery_2939
Acaryochloris_marina_MBIC11017_AM1_3592_rpoB
Thermosynechococcus_elongatus_tll0642_rpoB
Synechococcus_sp._PCC_6301_syc2507_d_rpoB
Paulinella_chromatophora_CHR_PCC_0097_rpoB
Paulinella
Synechococcus_sp._RCC307_SynRCC307_1959_rpoB
Prochlorococcus_marinus_MIT9313_PMT1507_rpoB
Prochlorococcus_marinus_MIT9303_P9303_04361_rpoB
Synechocossus_sp._WH7803_SynWH7803_2062_rpoB
Synechococcus_sp._CC9311_sync_2358_rpoB
Synechococcus_sp._9902_Syncc9902_0605
Synechococcus_sp._WH8102_SYNW0613_rpoB
Synechococcus_sp._9605_Syncc9605_2067
Prochlorococcus_marinus_NATL2A_PMN2A_1016
Prochlorococcus_marinus_NATL1A_NATL1_18861_rpoB
Prochlorococcus_marinus_CCMP1375_Pro1640_rpoB
Prochlorococcus_marinus_MIT9211_P9211_16071_rpoB
Prochlorococcus_marinus_CCMP_1986_PMM1485_rpoB
Prochlorococcus_marinus_MIT9315_P9515_16651_rpoB
Prochlorococcus_marinus_MIT9312_PMT9312_1578
Prochlorococcus_marinus_AS9601_A9601_16891_rpoB
Prochlorococcus_marinus_MIT9215_P9215_17541_rpoB
Prochlorococcus_marinus_MIT9301_P9301_16761_rpoB
Cyanobacteria
Bacillus_subtilis_Marburg_168_BSU01070_rpoB
Rhodospirillum_rubrum_ATCC_11170_Rru_A2695
Rhodopsudomonas_palustris_CGA009_RPA3268_rpoB
Escherichia_coli_K-12_MG1655_b3987_rpoB
Chlorobium_tepidum_CT0155_rpoB
Bacteria (outgroup)
0.3

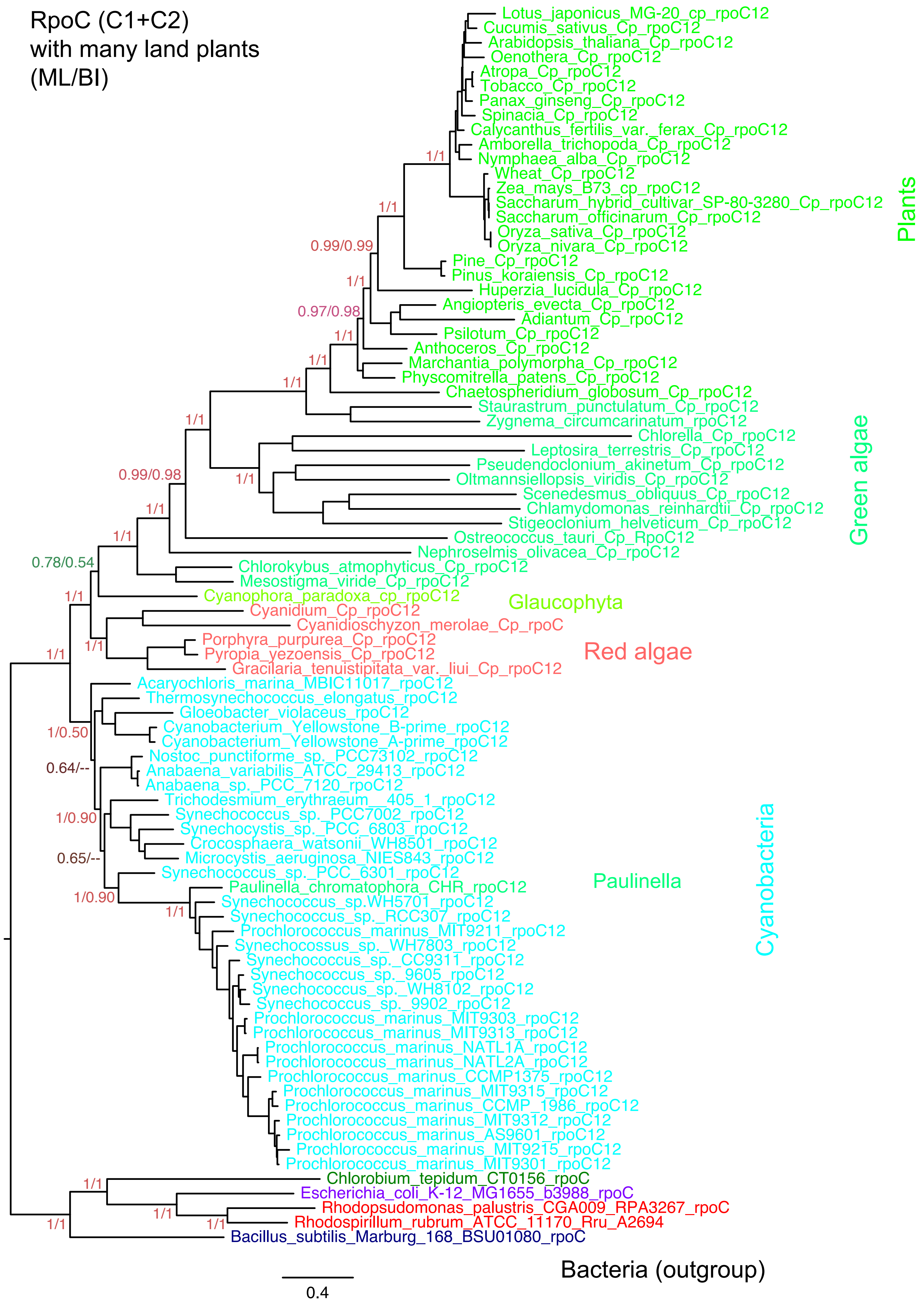

RpoC (C1+C2)
with many land plants
(ML/BI)
Lotus_japonicus_MG-20_cp_rpoC12
Cucumis_sativus_Cp_rpoC12
Arabidopsis_thaliana_Cp_rpoC12
Oenothera_Cp_rpoC12
Atropa_Cp_rpoC12
Tobacco_Cp_rpoC12
Panax_ginseng_Cp_rpoC12
Spinacia_Cp_rpoC12
Calycanthus_fertilis_var._ferax_Cp_rpoC12
Amborella_trichopoda_Cp_rpoC12
Nymphaea_alba_Cp_rpoC12
Wheat_Cp_rpoC12
Zea_mays_B73_cp_rpoC12
Saccharum_hybrid_cultivar_SP-80-3280_Cp_rpoC12
Saccharum_officinarum_Cp_rpoC12
Oryza_sativa_Cp_rpoC12
Oryza_nivara_Cp_rpoC12
Pine_Cp_rpoC12
Pinus_koraiensis_Cp_rpoC12
Huperzia_lucidula_Cp_rpoC12
Angiopteris_evecta_Cp_rpoC12
Adiantum_Cp_rpoC12
Psilotum_Cp_rpoC12
Anthoceros_Cp_rpoC12
Marchantia_polymorpha_Cp_rpoC12
Physcomitrella_patens_Cp_rpoC12
Chaetospheridium_globosum_Cp_rpoC12
Staurastrum_punctulatum_Cp_rpoC12
Zygnema_circumcarinatum_rpoC12
Chlorella_Cp_rpoC12
Leptosira_terrestris_Cp_rpoC12
Pseudendoclonium_akinetum_Cp_rpoC12
Oltmannsiellopsis_viridis_Cp_rpoC12
Scenedesmus_obliquus_Cp_rpoC12
Chlamydomonas_reinhardtii_Cp_rpoC12
Stigeoclonium_helveticum_Cp_rpoC12
Ostreococcus_tauri_Cp_RpoC12
Nephroselmis_olivacea_Cp_rpoC12
Chlorokybus_atmophyticus_Cp_rpoC12
Mesostigma_viride_Cp_rpoC12
Cyanophora_paradoxa_cp_rpoC12
Cyanidium_Cp_rpoC12
Cyanidioschyzon_merolae_Cp_rpoC
Porphyra_purpurea_Cp_rpoC12
Pyropia_yezoensis_Cp_rpoC12
Gracilaria_tenuistipitata_var._liui_Cp_rpoC12
Acaryochloris_marina_MBIC11017_rpoC12
Thermosynechococcus_elongatus_rpoC12
Gloeobacter_violaceus_rpoC12
Cyanobacterium_Yellowstone_B-prime_rpoC12
Cyanobacterium_Yellowstone_A-prime_rpoC12
Nostoc_punctiforme_sp._PCC73102_rpoC12
Anabaena_variabilis_ATCC_29413_rpoC12
Anabaena_sp._PCC_7120_rpoC12
Trichodesmium_erythraeum__405_1_rpoC12
Synechococcus_sp._PCC7002_rpoC12
Synechocystis_sp._PCC_6803_rpoC12
Crocosphaera_watsonii_WH8501_rpoC12
Microcystis_aeruginosa_NIES843_rpoC12
Synechococcus_sp._PCC_6301_rpoC12
Paulinella_chromatophora_CHR_rpoC12
Synechococcus_sp.WH5701_rpoC12
Synechococcus_sp._RCC307_rpoC12
Prochlorococcus_marinus_MIT9211_rpoC12
Synechocossus_sp._WH7803_rpoC12
Synechococcus_sp._CC9311_rpoC12
Synechococcus_sp._9605_rpoC12
Synechococcus_sp._WH8102_rpoC12
Synechococcus_sp._9902_rpoC12
Prochlorococcus_marinus_MIT9303_rpoC12
Prochlorococcus_marinus_MIT9313_rpoC12
Prochlorococcus_marinus_NATL1A_rpoC12
Prochlorococcus_marinus_NATL2A_rpoC12
Prochlorococcus_marinus_CCMP1375_rpoC12
Prochlorococcus_marinus_MIT9315_rpoC12
Prochlorococcus_marinus_CCMP_1986_rpoC12
Prochlorococcus_marinus_MIT9312_rpoC12
Prochlorococcus_marinus_AS9601_rpoC12
Prochlorococcus_marinus_MIT9215_rpoC12
Prochlorococcus_marinus_MIT9301_rpoC12
Chlorobium_tepidum_CT0156_rpoC
Escherichia_coli_K-12_MG1655_b3988_rpoC
Rhodopsudomonas_palustris_CGA009_RPA3267_rpoC
Rhodospirillum_rubrum_ATCC_11170_Rru_A2694
Bacillus_subtilis_Marburg_168_BSU01080_rpoC
Plants
Green algae
Glaucophyta
Red algae
Paulinella
Cyanobacteria
Bacteria (outgroup)
0.4
1/1
0.99/0.99
0.97/0.98
0.99/0.98
0.78/0.54
1/0.50
0.64/--
1/0.90
0.65/--

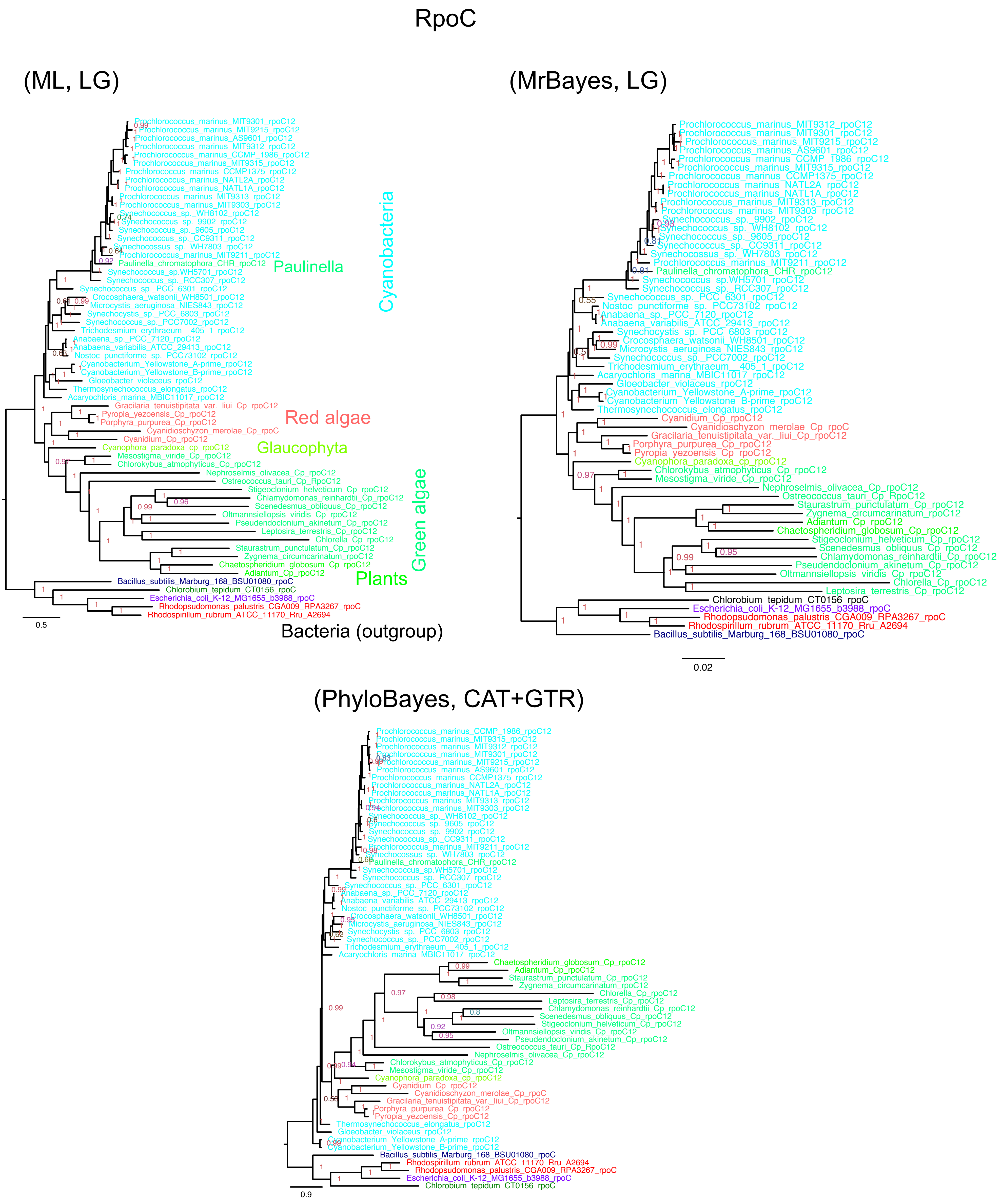
RpoC
(ML, LG)
Prochlorococcus_marinus_MIT9301_rpoC12
Prochlorococcus_marinus_MIT9215_rpoC12
Prochlorococcus_marinus_AS9601_rpoC12
Prochlorococcus_marinus_MIT9312_rpoC12
Prochlorococcus_marinus_CCMP_1986_rpoC12
Prochlorococcus_marinus_MIT9315_rpoC12
Prochlorococcus_marinus_CCMP1375_rpoC12
Prochlorococcus_marinus_NATL2A_rpoC12
Prochlorococcus_marinus_NATL1A_rpoC12
Prochlorococcus_marinus_MIT9313_rpoC12
Prochlorococcus_marinus_MIT9303_rpoC12
Synechococcus_sp._WH8102_rpoC12
Synechococcus_sp._9902_rpoC12
Synechococcus_sp._9605_rpoC12
Synechococcus_sp._CC9311_rpoC12
Synechocossus_sp._WH7803_rpoC12
Prochlorococcus_marinus_MIT9211_rpoC12
Paulinella_chromatophora_CHR_rpoC12
Synechococcus_sp.WH5701_rpoC12
Synechococcus_sp._RCC307_rpoC12
Synechococcus_sp._PCC_6301_rpoC12
Crocosphaera_watsonii_WH8501_rpoC12
Microcystis_aeruginosa_NIES843_rpoC12
Synechocystis_sp._PCC_6803_rpoC12
Synechococcus_sp._PCC7002_rpoC12
Trichodesmium_erythraeum__405_1_rpoC12
Anabaena_sp._PCC_7120_rpoC12
Anabaena_variabilis_ATCC_29413_rpoC12
Nostoc_punctiforme_sp._PCC73102_rpoC12
Cyanobacterium_Yellowstone_A-prime_rpoC12
Cyanobacterium_Yellowstone_B-prime_rpoC12
Gloeobacter_violaceus_rpoC12
Thermosynechococcus_elongatus_rpoC12
Acaryochloris_marina_MBIC11017_rpoC12
Gracilaria_tenuistipitata_var._liui_Cp_rpoC12
Pyropia_yezoensis_Cp_rpoC12
Porphyra_purpurea_Cp_rpoC12
Cyanidioschyzon_merolae_Cp_rpoC
Cyanidium_Cp_rpoC12
Cyanophora_paradoxa_cp_rpoC12
Mesostigma_viride_Cp_rpoC12
Chlorokybus_atmophyticus_Cp_rpoC12
Nephroselmis_olivacea_Cp_rpoC12
Ostreococcus_tauri_Cp_RpoC12
Stigeoclonium_helveticum_Cp_rpoC12
Chlamydomonas_reinhardtii_Cp_rpoC12
Scenedesmus_obliquus_Cp_rpoC12
Oltmannsiellopsis_viridis_Cp_rpoC12
Pseudendoclonium_akinetum_Cp_rpoC12
Leptosira_terrestris_Cp_rpoC12
Chlorella_Cp_rpoC12
Staurastrum_punctulatum_Cp_rpoC12
Zygnema_circumcarinatum_rpoC12
Chaetospheridium_globosum_Cp_rpoC12
Adiantum_Cp_rpoC12
Bacillus_subtilis_Marburg_168_BSU01080_rpoC
Chlorobium_tepidum_CT0156_rpoC
Escherichia_coli_K-12_MG1655_b3988_rpoC
Rhodopsudomonas_palustris_CGA009_RPA3267_rpoC
Rhodospirillum_rubrum_ATCC_11170_Rru_A2694
0.5
Cyanobacteria
Paulinella
Red algae
Glaucophyta
Green algae
Plants
Bacteria (outgroup)
(MrBayes, LG)
Prochlorococcus_marinus_MIT9312_rpoC12
Prochlorococcus_marinus_MIT9301_rpoC12
Prochlorococcus_marinus_MIT9215_rpoC12
Prochlorococcus_marinus_AS9601_rpoC12
Prochlorococcus_marinus_CCMP_1986_rpoC12
Prochlorococcus_marinus_MIT9315_rpoC12
Prochlorococcus_marinus_CCMP1375_rpoC12
Prochlorococcus_marinus_NATL2A_rpoC12
Prochlorococcus_marinus_NATL1A_rpoC12
Prochlorococcus_marinus_MIT9313_rpoC12
Prochlorococcus_marinus_MIT9303_rpoC12
Synechococcus_sp._9902_rpoC12
Synechococcus_sp._WH8102_rpoC12
Synechococcus_sp._9605_rpoC12
Synechococcus_sp._CC9311_rpoC12
Synechocossus_sp._WH7803_rpoC12
Prochlorococcus_marinus_MIT9211_rpoC12
Paulinella_chromatophora_CHR_rpoC12
Synechococcus_sp.WH5701_rpoC12
Synechococcus_sp._RCC307_rpoC12
Synechococcus_sp._PCC_6301_rpoC12
Nostoc_punctiforme_sp._PCC73102_rpoC12
Anabaena_sp._PCC_7120_rpoC12
Anabaena_variabilis_ATCC_29413_rpoC12
Synechocystis_sp._PCC_6803_rpoC12
Crocosphaera_watsonii_WH8501_rpoC12
Microcystis_aeruginosa_NIES843_rpoC12
Synechococcus_sp._PCC7002_rpoC12
Trichodesmium_erythraeum__405_1_rpoC12
Acaryochloris_marina_MBIC11017_rpoC12
Gloeobacter_violaceus_rpoC12
Cyanobacterium_Yellowstone_A-prime_rpoC12
Cyanobacterium_Yellowstone_B-prime_rpoC12
Thermosynechococcus_elongatus_rpoC12
Cyanidium_Cp_rpoC12
Cyanidioschyzon_merolae_Cp_rpoC
Gracilaria_tenuistipitata_var._liui_Cp_rpoC12
Porphyra_purpurea_Cp_rpoC12
Pyropia_yezoensis_Cp_rpoC12
Cyanophora_paradoxa_cp_rpoC12
Chlorokybus_atmophyticus_Cp_rpoC12
Mesostigma_viride_Cp_rpoC12
Nephroselmis_olivacea_Cp_rpoC12
Ostreococcus_tauri_Cp_RpoC12
Staurastrum_punctulatum_Cp_rpoC12
Zygnema_circumcarinatum_rpoC12
Adiantum_Cp_rpoC12
Chaetospheridium_globosum_Cp_rpoC12
Stigeoclonium_helveticum_Cp_rpoC12
Scenedesmus_obliquus_Cp_rpoC12
Chlamydomonas_reinhardtii_Cp_rpoC12
Pseudendoclonium_akinetum_Cp_rpoC12
Oltmannsiellopsis_viridis_Cp_rpoC12
Chlorella_Cp_rpoC12
Leptosira_terrestris_Cp_rpoC12
Chlorobium_tepidum_CT0156_rpoC
Escherichia_coli_K-12_MG1655_b3988_rpoC
Rhodopsudomonas_palustris_CGA009_RPA3267_rpoC
Rhodospirillum_rubrum_ATCC_11170_Rru_A2694
Bacillus_subtilis_Marburg_168_BSU01080_rpoC
0.02
(PhyloBayes, CAT+GTR)
Prochlorococcus_marinus_CCMP_1986_rpoC12
Prochlorococcus_marinus_MIT9315_rpoC12
Prochlorococcus_marinus_MIT9312_rpoC12
Prochlorococcus_marinus_MIT9301_rpoC12
Prochlorococcus_marinus_MIT9215_rpoC12
Prochlorococcus_marinus_AS9601_rpoC12
Prochlorococcus_marinus_CCMP1375_rpoC12
Prochlorococcus_marinus_NATL2A_rpoC12
Prochlorococcus_marinus_NATL1A_rpoC12
Prochlorococcus_marinus_MIT9313_rpoC12
Prochlorococcus_marinus_MIT9303_rpoC12
Synechococcus_sp._WH8102_rpoC12
Synechococcus_sp._9605_rpoC12
Synechococcus_sp._9902_rpoC12
Synechococcus_sp._CC9311_rpoC12
Prochlorococcus_marinus_MIT9211_rpoC12
Synechocossus_sp._WH7803_rpoC12
Paulinella_chromatophora_CHR_rpoC12
Synechococcus_sp.WH5701_rpoC12
Synechococcus_sp._RCC307_rpoC12
Synechococcus_sp._PCC_6301_rpoC12
Anabaena_sp._PCC_7120_rpoC12
Anabaena_variabilis_ATCC_29413_rpoC12
Nostoc_punctiforme_sp._PCC73102_rpoC12
Crocosphaera_watsonii_WH8501_rpoC12
Microcystis_aeruginosa_NIES843_rpoC12
Synechocystis_sp._PCC_6803_rpoC12
Synechococcus_sp._PCC7002_rpoC12
Trichodesmium_erythraeum__405_1_rpoC12
Acaryochloris_marina_MBIC11017_rpoC12
Chaetospheridium_globosum_Cp_rpoC12
Adiantum_Cp_rpoC12
Staurastrum_punctulatum_Cp_rpoC12
Zygnema_circumcarinatum_rpoC12
Chlorella_Cp_rpoC12
Leptosira_terrestris_Cp_rpoC12
Chlamydomonas_reinhardtii_Cp_rpoC12
Scenedesmus_obliquus_Cp_rpoC12
Stigeoclonium_helveticum_Cp_rpoC12
Oltmannsiellopsis_viridis_Cp_rpoC12
Pseudendoclonium_akinetum_Cp_rpoC12
Ostreococcus_tauri_Cp_RpoC12
Nephroselmis_olivacea_Cp_rpoC12
Chlorokybus_atmophyticus_Cp_rpoC12
Mesostigma_viride_Cp_rpoC12
Cyanophora_paradoxa_cp_rpoC12
Cyanidium_Cp_rpoC12
Cyanidioschyzon_merolae_Cp_rpoC
Gracilaria_tenuistipitata_var._liui_Cp_rpoC12
Porphyra_purpurea_Cp_rpoC12
Pyropia_yezoensis_Cp_rpoC12
Thermosynechococcus_elongatus_rpoC12
Gloeobacter_violaceus_rpoC12
Cyanobacterium_Yellowstone_A-prime_rpoC12
Cyanobacterium_Yellowstone_B-prime_rpoC12
Bacillus_subtilis_Marburg_168_BSU01080_rpoC
Rhodospirillum_rubrum_ATCC_11170_Rru_A2694
Rhodopsudomonas_palustris_CGA009_RPA3267_rpoC
Escherichia_coli_K-12_MG1655_b3988_rpoC
Chlorobium_tepidum_CT0156_rpoC
0.9

PBP (Cluster 705 in Gclust2012_42)

(BI/ML)

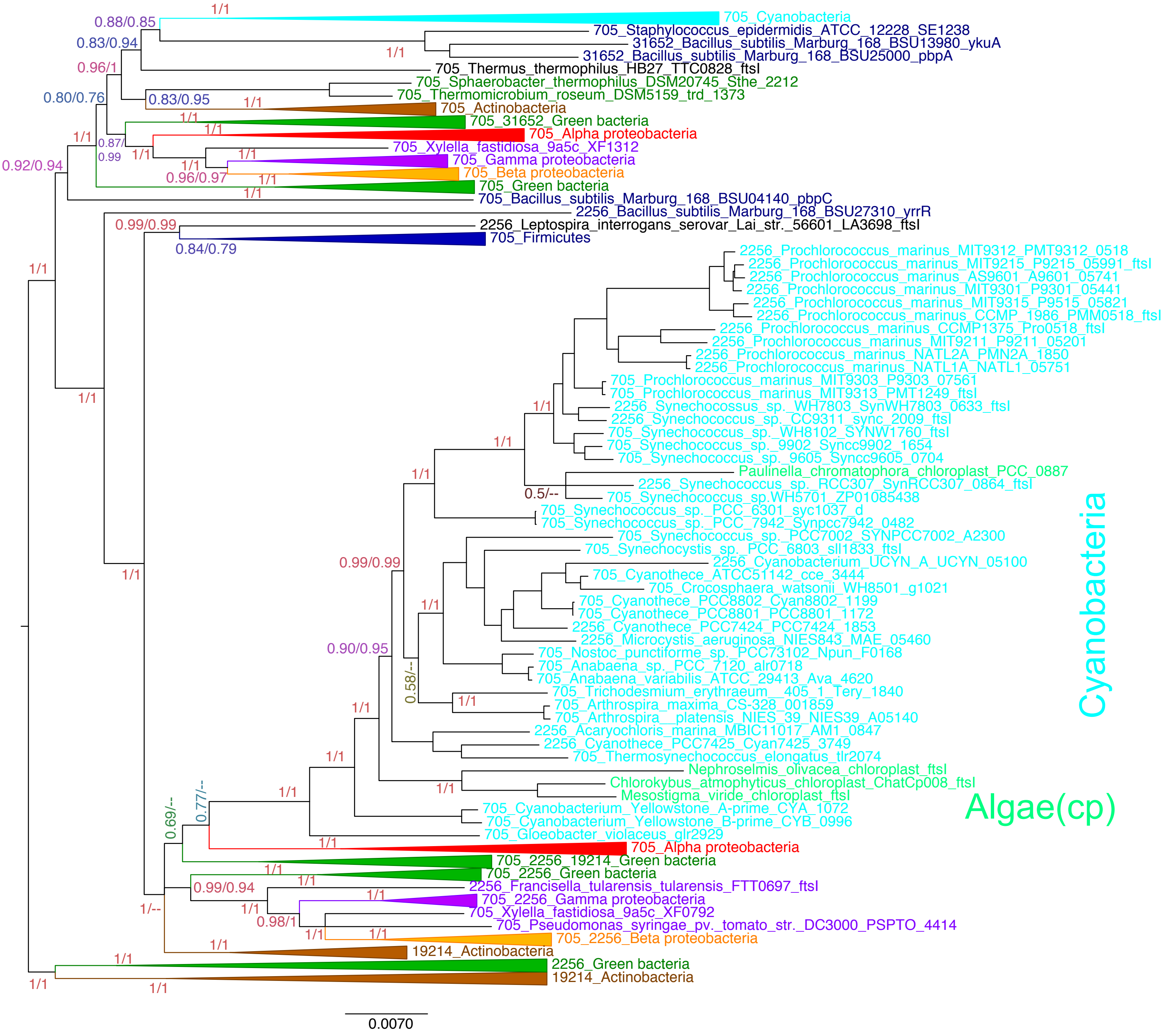

Supplement: Supplementary file 4 — Supplementary material 4 (PDF 3118 kb) [file 10265_2019_1157_MOESM4_ESM.pdf]
