# Supplementary material for: Complex origins of chloroplast membranes with photosynthetic machineries: multiple transfers of genes from divergent organisms at different times or a single endosymbiotic event?
Source: J Plant Res. 2019 Dec 6;133(1):15–33. doi: 10.1007/s10265-019-01157-z (PMC6946739; doi:10.1007/s10265-019-01157-z)
Supplement: Supplementary file 5 — Supplementary material 5 (PDF 186 kb) [file 10265_2019_1157_MOESM5_ESM.pdf]

Journal of Plant Research
Online Resources

Complex origins of chloroplast membranes with photosynthetic machineries: Multiple transfers of genes from divergent organisms at different times or a single endosymbiotic event?

Naoki Sato
University of Tokyo
naokisat@bio.c.u-tokyo.ac.jp

Online Resource 5. Phylogenetic trees of Ndh proteins.

Nine components of the chloroplast Ndh complex, A, B, C, E, F, G, H, I, and K are analyzed, either individually or as a catenated sequences. The sequences were retrieved from Gclust dataset CPBACT10. Phylogeny of NdhD was complicated because of many paralogs, whereas NdhJ was not present in *Nephroselmis*. These two subunits were not analyzed with other subunits, but individual trees were constructed based on Gclust dataset CyanoClust 4.

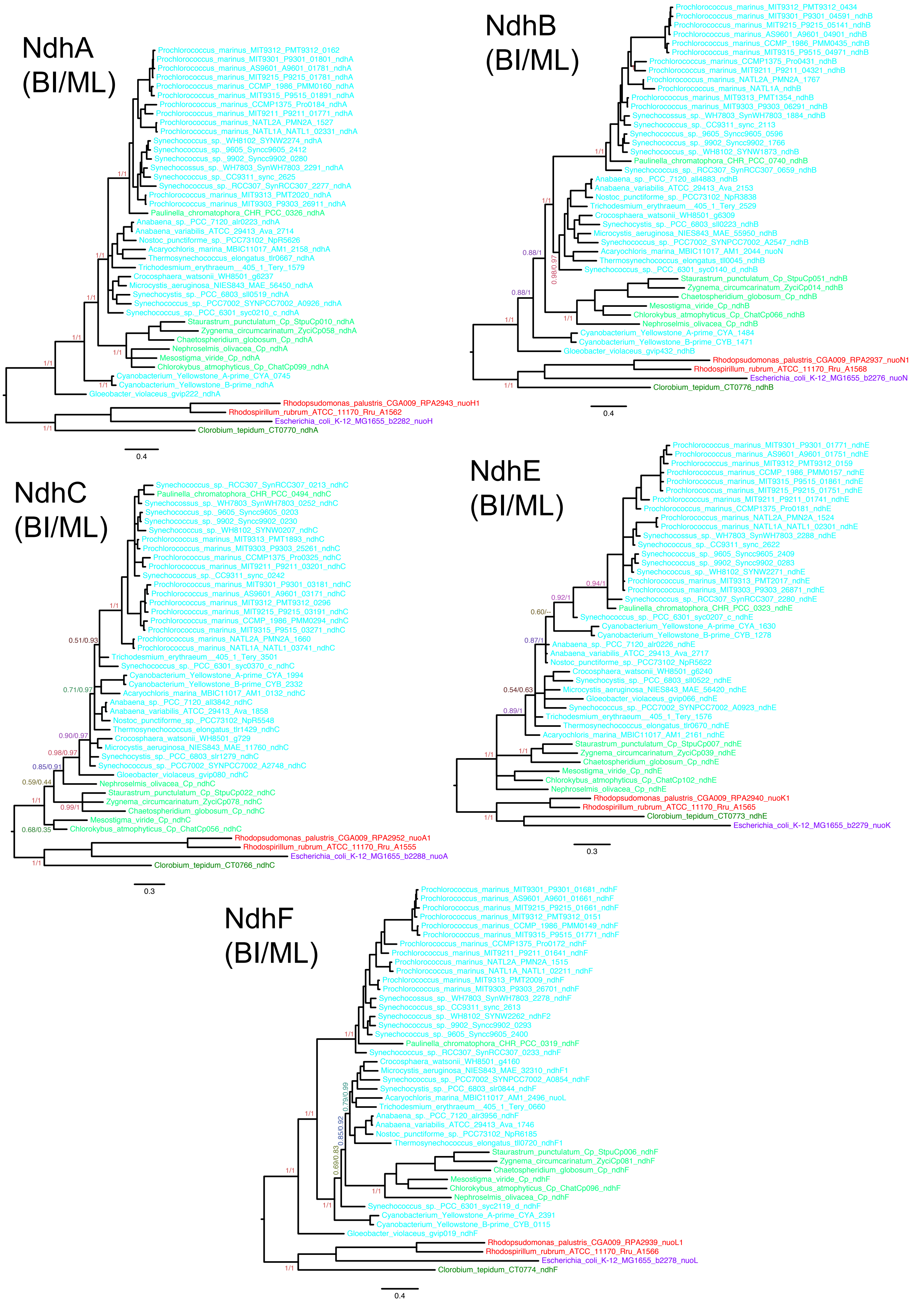
NdhA (BI/ML)
Prochlorococcus_marinus_MIT9312_PMT9312_0162
Prochlorococcus_marinus_MIT9301_P9301_01801_ndhA
Prochlorococcus_marinus_AS9601_A9601_01781_ndhA
Prochlorococcus_marinus_MIT9215_P9215_01781_ndhA
Prochlorococcus_marinus_CCMP_1986_PMM0160_ndhA
Prochlorococcus_marinus_MIT9315_P9515_01891_ndhA
Prochlorococcus_marinus_CCMP1375_Pro0184_ndhA
Prochlorococcus_marinus_MIT9211_P9211_01771_ndhA
Prochlorococcus_marinus_NATL2A_PMN2A_1527
Prochlorococcus_marinus_NATL1A_NATL1_02331_ndhA
Synechococcus_sp._WH8102_SYNW2274_ndhA
Synechococcus_sp._9605_Syncc9605_2412
Synechococcus_sp._9902_Syncc9902_0280
Synechocossus_sp._WH7803_SynWH7803_2291_ndhA
Synechococcus_sp._CC9311_sync_2625
Synechococcus_sp._RCC307_SynRCC307_2277_ndhA
Prochlorococcus_marinus_MIT9313_PMT2020_ndhA
Prochlorococcus_marinus_MIT9303_P9303_26911_ndhA
Paulinella_chromatophora_CHR_PCC_0326_ndhA
Anabaena_sp._PCC_7120_alr0223_ndhA
Anabaena_variabilis_ATCC_29413_Ava_2714
Nostoc_punctiforme_sp._PCC73102_NpR5626
Acaryochloris_marina_MBIC11017_AM1_2158_ndhA
Thermosynechococcus_elongatus_tlr0667_ndhA
Trichodesmium_erythraeum__405_1_Tery_1579
Crocosphaera_watsonii_WH8501_g6237
Microcystis_aeruginosa_NIES843_MAE_56450_ndhA
Synechocystis_sp._PCC_6803_sll0519_ndhA
Synechococcus_sp._PCC7002_SYNPCC7002_A0926_ndhA
Synechococcus_sp._PCC_6301_syc0210_c_ndhA
Staurastrum_punctulatum_Cp_StpuCp010_ndhA
Zygnema_circumcarinatum_ZyciCp058_ndhA
Chaetospheridium_globosum_Cp_ndhA
Nephroselmis_olivacea_Cp_ndhA
Mesostigma_viride_Cp_ndhA
Chlorokybus_atmophyticus_Cp_ChatCp099_ndhA
Cyanobacterium_Yellowstone_A-prime_CYA_0745
Cyanobacterium_Yellowstone_B-prime_ndhA
Gloeobacter_violaceus_gvip222_ndhA
Rhodopsudomonas_palustris_CGA009_RPA2943_nuoH1
Rhodospirillum_rubrum_ATCC_11170_Rru_A1562
Escherichia_coli_K-12_MG1655_b2282_nuoH
Clorobium_tepidum_CT0770_ndhA
1/1
0.4
NdhB (BI/ML)
Prochlorococcus_marinus_MIT9312_PMT9312_0434
Prochlorococcus_marinus_MIT9301_P9301_04591_ndhB
Prochlorococcus_marinus_MIT9215_P9215_05141_ndhB
Prochlorococcus_marinus_AS9601_A9601_04901_ndhB
Prochlorococcus_marinus_CCMP_1986_PMM0435_ndhB
Prochlorococcus_marinus_MIT9315_P9515_04971_ndhB
Prochlorococcus_marinus_CCMP1375_Pro0431_ndhB
Prochlorococcus_marinus_MIT9211_P9211_04321_ndhB
Prochlorococcus_marinus_NATL2A_PMN2A_1767
Prochlorococcus_marinus_NATL1A_ndhB
Prochlorococcus_marinus_MIT9313_PMT1354_ndhB
Prochlorococcus_marinus_MIT9303_P9303_06291_ndhB
Synechocossus_sp._WH7803_SynWH7803_1884_ndhB
Synechococcus_sp._CC9311_sync_2113
Synechococcus_sp._9605_Syncc9605_0596
Synechococcus_sp._9902_Syncc9902_1766
Synechococcus_sp._WH8102_SYNW1873_ndhB
Paulinella_chromatophora_CHR_PCC_0740_ndhB
Synechococcus_sp._RCC307_SynRCC307_0659_ndhB
Anabaena_sp._PCC_7120_all4883_ndhB
Anabaena_variabilis_ATCC_29413_Ava_2153
Nostoc_punctiforme_sp._PCC73102_NpR3838
Trichodesmium_erythraeum__405_1_Tery_2529
Crocosphaera_watsonii_WH8501_g6309
Synechocystis_sp._PCC_6803_sll0223_ndhB
Microcystis_aeruginosa_NIES843_MAE_55950_ndhB
Synechococcus_sp._PCC7002_SYNPCC7002_A2547_ndhB
Acaryochloris_marina_MBIC11017_AM1_2044_nuoN
Thermosynechococcus_elongatus_tll0045_ndhB
Synechococcus_sp._PCC_6301_syc0140_d_ndhB
Staurastrum_punctulatum_Cp_StpuCp051_ndhB
Zygnema_circumcarinatum_ZyciCp014_ndhB
Chaetospheridium_globosum_Cp_ndhB
Mesostigma_viride_Cp_ndhB
Chlorokybus_atmophyticus_Cp_ChatCp066_ndhB
Nephroselmis_olivacea_Cp_ndhB
Cyanobacterium_Yellowstone_A-prime_CYA_1484
Cyanobacterium_Yellowstone_B-prime_CYB_1471
Gloeobacter_violaceus_gvip432_ndhB
Rhodopsudomonas_palustris_CGA009_RPA2937_nuoN1
Rhodospirillum_rubrum_ATCC_11170_Rru_A1568
Escherichia_coli_K-12_MG1655_b2276_nuoN
Clorobium_tepidum_CT0776_ndhB
1/1
0.88/1
0.98/0.97
0.4
NdhC (BI/ML)
Synechococcus_sp._RCC307_SynRCC307_0213_ndhC
Paulinella_chromatophora_CHR_PCC_0494_ndhC
Synechocossus_sp._WH7803_SynWH7803_0252_ndhC
Synechococcus_sp._9605_Syncc9605_0203
Synechococcus_sp._9902_Syncc9902_0230
Synechococcus_sp._WH8102_SYNW0207_ndhC
Prochlorococcus_marinus_MIT9313_PMT1893_ndhC
Prochlorococcus_marinus_MIT9303_P9303_25261_ndhC
Prochlorococcus_marinus_CCMP1375_Pro0325_ndhC
Prochlorococcus_marinus_MIT9211_P9211_03201_ndhC
Synechococcus_sp._CC9311_sync_0242
Prochlorococcus_marinus_MIT9301_P9301_03181_ndhC
Prochlorococcus_marinus_AS9601_A9601_03171_ndhC
Prochlorococcus_marinus_MIT9312_PMT9312_0296
Prochlorococcus_marinus_MIT9215_P9215_03191_ndhC
Prochlorococcus_marinus_CCMP_1986_PMM0294_ndhC
Prochlorococcus_marinus_MIT9315_P9515_03271_ndhC
Prochlorococcus_marinus_NATL2A_PMN2A_1660
Prochlorococcus_marinus_NATL1A_NATL1_03741_ndhC
Trichodesmium_erythraeum__405_1_Tery_3501
Synechococcus_sp._PCC_6301_syc0370_c_ndhC
Cyanobacterium_Yellowstone_A-prime_CYA_1994
Cyanobacterium_Yellowstone_B-prime_CYB_2332
Acaryochloris_marina_MBIC11017_AM1_0132_ndhC
Anabaena_sp._PCC_7120_all3842_ndhC
Anabaena_variabilis_ATCC_29413_Ava_1858
Nostoc_punctiforme_sp._PCC73102_NpR5548
Thermosynechococcus_elongatus_tlr1429_ndhC
Crocosphaera_watsonii_WH8501_g729
Microcystis_aeruginosa_NIES843_MAE_11760_ndhC
Synechocystis_sp._PCC_6803_slr1279_ndhC
Synechococcus_sp._PCC7002_SYNPCC7002_A2748_ndhC
Gloeobacter_violaceus_gvip080_ndhC
Nephroselmis_olivacea_Cp_ndhC
Staurastrum_punctulatum_Cp_StpuCp022_ndhC
Zygnema_circumcarinatum_ZyciCp078_ndhC
Chaetospheridium_globosum_Cp_ndhC
Mesostigma_viride_Cp_ndhC
Chlorokybus_atmophyticus_Cp_ChatCp056_ndhC
Rhodopsudomonas_palustris_CGA009_RPA2952_nuoA1
Rhodospirillum_rubrum_ATCC_11170_Rru_A1555
Escherichia_coli_K-12_MG1655_b2288_nuoA
Clorobium_tepidum_CT0766_ndhC
1/1
0.51/0.93
0.71/0.97
0.90/0.97
0.98/0.97
0.85/0.91
0.59/0.44
0.99/1
0.68/0.35
0.3
NdhE (BI/ML)
Prochlorococcus_marinus_MIT9301_P9301_01771_ndhE
Prochlorococcus_marinus_AS9601_A9601_01751_ndhE
Prochlorococcus_marinus_MIT9312_PMT9312_0159
Prochlorococcus_marinus_CCMP_1986_PMM0157_ndhE
Prochlorococcus_marinus_MIT9315_P9515_01861_ndhE
Prochlorococcus_marinus_MIT9215_P9215_01751_ndhE
Prochlorococcus_marinus_MIT9211_P9211_01741_ndhE
Prochlorococcus_marinus_CCMP1375_Pro0181_ndhE
Prochlorococcus_marinus_NATL2A_PMN2A_1524
Prochlorococcus_marinus_NATL1A_NATL1_02301_ndhE
Synechocossus_sp._WH7803_SynWH7803_2288_ndhE
Synechococcus_sp._CC9311_sync_2622
Synechococcus_sp._9605_Syncc9605_2409
Synechococcus_sp._9902_Syncc9902_0283
Synechococcus_sp._WH8102_SYNW2271_ndhE
Prochlorococcus_marinus_MIT9313_PMT2017_ndhE
Prochlorococcus_marinus_MIT9303_P9303_26871_ndhE
Synechococcus_sp._RCC307_SynRCC307_2280_ndhE
Paulinella_chromatophora_CHR_PCC_0323_ndhE
Synechococcus_sp._PCC_6301_syc0207_c_ndhE
Cyanobacterium_Yellowstone_A-prime_CYA_1630
Cyanobacterium_Yellowstone_B-prime_CYB_1278
Anabaena_sp._PCC_7120_alr0226_ndhE
Anabaena_variabilis_ATCC_29413_Ava_2717
Nostoc_punctiforme_sp._PCC73102_NpR5622
Crocosphaera_watsonii_WH8501_g6240
Synechocystis_sp._PCC_6803_sll0522_ndhE
Microcystis_aeruginosa_NIES843_MAE_56420_ndhE
Gloeobacter_violaceus_gvip066_ndhE
Synechococcus_sp._PCC7002_SYNPCC7002_A0923_ndhE
Trichodesmium_erythraeum__405_1_Tery_1576
Thermosynechococcus_elongatus_tlr0670_ndhE
Acaryochloris_marina_MBIC11017_AM1_2161_ndhE
Staurastrum_punctulatum_Cp_StpuCp007_ndhE
Zygnema_circumcarinatum_ZyciCp039_ndhE
Chaetospheridium_globosum_Cp_ndhE
Mesostigma_viride_Cp_ndhE
Chlorokybus_atmophyticus_Cp_ChatCp102_ndhE
Nephroselmis_olivacea_Cp_ndhE
Rhodopsudomonas_palustris_CGA009_RPA2940_nuoK1
Rhodospirillum_rubrum_ATCC_11170_Rru_A1565
Clorobium_tepidum_CT0773_ndhE
Escherichia_coli_K-12_MG1655_b2279_nuoK
0.94/1
0.92/1
0.60/--
0.87/1
0.54/0.63
0.89/1
1/1
0.3
NdhF (BI/ML)
Prochlorococcus_marinus_MIT9301_P9301_01681_ndhF
Prochlorococcus_marinus_AS9601_A9601_01661_ndhF
Prochlorococcus_marinus_MIT9215_P9215_01661_ndhF
Prochlorococcus_marinus_MIT9312_PMT9312_0151
Prochlorococcus_marinus_CCMP_1986_PMM0149_ndhF
Prochlorococcus_marinus_MIT9315_P9515_01771_ndhF
Prochlorococcus_marinus_CCMP1375_Pro0172_ndhF
Prochlorococcus_marinus_MIT9211_P9211_01641_ndhF
Prochlorococcus_marinus_NATL2A_PMN2A_1515
Prochlorococcus_marinus_NATL1A_NATL1_02211_ndhF
Prochlorococcus_marinus_MIT9313_PMT2009_ndhF
Prochlorococcus_marinus_MIT9303_P9303_26701_ndhF
Synechocossus_sp._WH7803_SynWH7803_2278_ndhF
Synechococcus_sp._CC9311_sync_2613
Synechococcus_sp._WH8102_SYNW2262_ndhF2
Synechococcus_sp._9902_Syncc9902_0293
Synechococcus_sp._9605_Syncc9605_2400
Paulinella_chromatophora_CHR_PCC_0319_ndhF
Synechococcus_sp._RCC307_SynRCC307_0233_ndhF
Crocosphaera_watsonii_WH8501_g4160
Microcystis_aeruginosa_NIES843_MAE_32310_ndhF1
Synechococcus_sp._PCC7002_SYNPCC7002_A0854_ndhF
Synechocystis_sp._PCC_6803_slr0844_ndhF
Acaryochloris_marina_MBIC11017_AM1_2496_nuoL
Trichodesmium_erythraeum__405_1_Tery_0660
Anabaena_sp._PCC_7120_alr3956_ndhF
Anabaena_variabilis_ATCC_29413_Ava_1746
Nostoc_punctiforme_sp._PCC73102_NpR6185
Thermosynechococcus_elongatus_tll0720_ndhF1
Staurastrum_punctulatum_Cp_StpuCp006_ndhF
Zygnema_circumcarinatum_ZyciCp081_ndhF
Chaetospheridium_globosum_Cp_ndhF
Mesostigma_viride_Cp_ndhF
Chlorokybus_atmophyticus_Cp_ChatCp096_ndhF
Nephroselmis_olivacea_Cp_ndhF
Synechococcus_sp._PCC_6301_syc2119_d_ndhF
Cyanobacterium_Yellowstone_A-prime_CYA_2391
Cyanobacterium_Yellowstone_B-prime_CYB_0115
Gloeobacter_violaceus_gvip019_ndhF
Rhodopsudomonas_palustris_CGA009_RPA2939_nuoL1
Rhodospirillum_rubrum_ATCC_11170_Rru_A1566
Escherichia_coli_K-12_MG1655_b2278_nuoL
Clorobium_tepidum_CT0774_ndhF
1/1
0.79/0.99
0.85/0.92
0.69/0.83
0.4

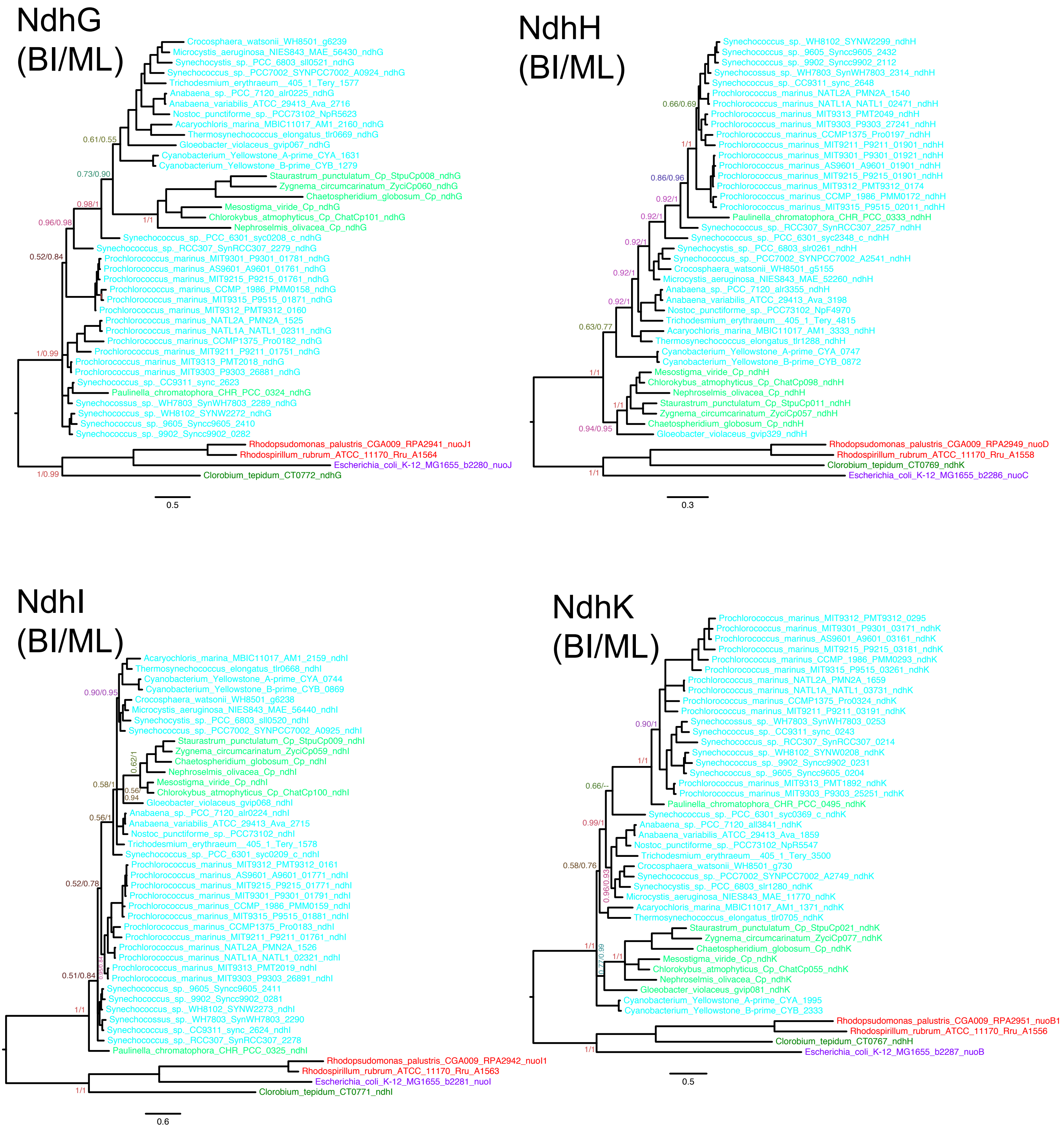
NdhG (BI/ML)
Crocosphaera_watsonii_WH8501_g6239
Microcystis_aeruginosa_NIES843_MAE_56430_ndhG
Synechocystis_sp._PCC_6803_sll0521_ndhG
Synechococcus_sp._PCC7002_SYNPCC7002_A0924_ndhG
Trichodesmium_erythraeum__405_1_Tery_1577
Anabaena_sp._PCC_7120_alr0225_ndhG
Anabaena_variabilis_ATCC_29413_Ava_2716
Nostoc_punctiforme_sp._PCC73102_NpR5623
Acaryochloris_marina_MBIC11017_AM1_2160_ndhG
Thermosynechococcus_elongatus_tlr0669_ndhG
Gloeobacter_violaceus_gvip067_ndhG
Cyanobacterium_Yellowstone_A-prime_CYA_1631
Cyanobacterium_Yellowstone_B-prime_CYB_1279
Staurastrum_punctulatum_Cp_StpuCp008_ndhG
Zygnema_circumcarinatum_ZyciCp060_ndhG
Chaetospheridium_globosum_Cp_ndhG
Mesostigma_viride_Cp_ndhG
Chlorokybus_atmophyticus_Cp_ChatCp101_ndhG
Nephroselmis_olivacea_Cp_ndhG
Synechococcus_sp._PCC_6301_syc0208_c_ndhG
Synechococcus_sp._RCC307_SynRCC307_2279_ndhG
Prochlorococcus_marinus_MIT9301_P9301_01781_ndhG
Prochlorococcus_marinus_AS9601_A9601_01761_ndhG
Prochlorococcus_marinus_MIT9215_P9215_01761_ndhG
Prochlorococcus_marinus_CCMP_1986_PMM0158_ndhG
Prochlorococcus_marinus_MIT9315_P9515_01871_ndhG
Prochlorococcus_marinus_MIT9312_PMT9312_0160
Prochlorococcus_marinus_NATL2A_PMN2A_1525
Prochlorococcus_marinus_NATL1A_NATL1_02311_ndhG
Prochlorococcus_marinus_CCMP1375_Pro0182_ndhG
Prochlorococcus_marinus_MIT9211_P9211_01751_ndhG
Prochlorococcus_marinus_MIT9313_PMT2018_ndhG
Prochlorococcus_marinus_MIT9303_P9303_26881_ndhG
Synechococcus_sp._CC9311_sync_2623
Paulinella_chromatophora_CHR_PCC_0324_ndhG
Synechocossus_sp._WH7803_SynWH7803_2289_ndhG
Synechococcus_sp._WH8102_SYNW2272_ndhG
Synechococcus_sp._9605_Syncc9605_2410
Synechococcus_sp._9902_Syncc9902_0282
Rhodopsudomonas_palustris_CGA009_RPA2941_nuoJ1
Rhodospirillum_rubrum_ATCC_11170_Rru_A1564
Escherichia_coli_K-12_MG1655_b2280_nuoJ
Clorobium_tepidum_CT0772_ndhG
0.61/0.55
0.73/0.90
0.98/1
1/1
0.96/0.98
0.52/0.84
1/0.99
1/0.99
0.5
NdhH (BI/ML)
Synechococcus_sp._WH8102_SYNW2299_ndhH
Synechococcus_sp._9605_Syncc9605_2432
Synechococcus_sp._9902_Syncc9902_2112
Synechocossus_sp._WH7803_SynWH7803_2314_ndhH
Synechococcus_sp._CC9311_sync_2648
Prochlorococcus_marinus_NATL2A_PMN2A_1540
Prochlorococcus_marinus_NATL1A_NATL1_02471_ndhH
Prochlorococcus_marinus_MIT9313_PMT2049_ndhH
Prochlorococcus_marinus_MIT9303_P9303_27241_ndhH
Prochlorococcus_marinus_CCMP1375_Pro0197_ndhH
Prochlorococcus_marinus_MIT9211_P9211_01901_ndhH
Prochlorococcus_marinus_MIT9301_P9301_01921_ndhH
Prochlorococcus_marinus_AS9601_A9601_01901_ndhH
Prochlorococcus_marinus_MIT9215_P9215_01901_ndhH
Prochlorococcus_marinus_MIT9312_PMT9312_0174
Prochlorococcus_marinus_CCMP_1986_PMM0172_ndhH
Prochlorococcus_marinus_MIT9315_P9515_02011_ndhH
Paulinella_chromatophora_CHR_PCC_0333_ndhH
Synechococcus_sp._RCC307_SynRCC307_2257_ndhH
Synechococcus_sp._PCC_6301_syc2348_c_ndhH
Synechocystis_sp._PCC_6803_slr0261_ndhH
Synechococcus_sp._PCC7002_SYNPCC7002_A2541_ndhH
Crocosphaera_watsonii_WH8501_g5155
Microcystis_aeruginosa_NIES843_MAE_52260_ndhH
Anabaena_sp._PCC_7120_alr3355_ndhH
Anabaena_variabilis_ATCC_29413_Ava_3198
Nostoc_punctiforme_sp._PCC73102_NpF4970
Trichodesmium_erythraeum__405_1_Tery_4815
Acaryochloris_marina_MBIC11017_AM1_3333_ndhH
Thermosynechococcus_elongatus_tlr1288_ndhH
Cyanobacterium_Yellowstone_A-prime_CYA_0747
Cyanobacterium_Yellowstone_B-prime_CYB_0872
Mesostigma_viride_Cp_ndhH
Chlorokybus_atmophyticus_Cp_ChatCp098_ndhH
Nephroselmis_olivacea_Cp_ndhH
Staurastrum_punctulatum_Cp_StpuCp011_ndhH
Zygnema_circumcarinatum_ZyciCp057_ndhH
Chaetospheridium_globosum_Cp_ndhH
Gloeobacter_violaceus_gvip329_ndhH
Rhodopsudomonas_palustris_CGA009_RPA2949_nuoD
Rhodospirillum_rubrum_ATCC_11170_Rru_A1558
Clorobium_tepidum_CT0769_ndhK
Escherichia_coli_K-12_MG1655_b2286_nuoC
0.66/0.69
1/1
0.86/0.96
0.92/1
0.92/1
0.92/1
0.92/1
0.92/1
0.63/0.77
1/1
1/1
0.94/0.95
1/1
0.3
NdhI (BI/ML)
Acaryochloris_marina_MBIC11017_AM1_2159_ndhI
Thermosynechococcus_elongatus_tlr0668_ndhI
Cyanobacterium_Yellowstone_A-prime_CYA_0744
Cyanobacterium_Yellowstone_B-prime_CYB_0869
Crocosphaera_watsonii_WH8501_g6238
Microcystis_aeruginosa_NIES843_MAE_56440_ndhI
Synechocystis_sp._PCC_6803_sll0520_ndhI
Synechococcus_sp._PCC7002_SYNPCC7002_A0925_ndhI
Staurastrum_punctulatum_Cp_StpuCp009_ndhI
Zygnema_circumcarinatum_ZyciCp059_ndhI
Chaetospheridium_globosum_Cp_ndhI
Nephroselmis_olivacea_Cp_ndhI
Mesostigma_viride_Cp_ndhI
Chlorokybus_atmophyticus_Cp_ChatCp100_ndhI
Gloeobacter_violaceus_gvip068_ndhI
Anabaena_sp._PCC_7120_alr0224_ndhI
Anabaena_variabilis_ATCC_29413_Ava_2715
Nostoc_punctiforme_sp._PCC73102_ndhI
Trichodesmium_erythraeum__405_1_Tery_1578
Synechococcus_sp._PCC_6301_syc0209_c_ndhI
Prochlorococcus_marinus_MIT9312_PMT9312_0161
Prochlorococcus_marinus_AS9601_A9601_01771_ndhI
Prochlorococcus_marinus_MIT9215_P9215_01771_ndhI
Prochlorococcus_marinus_MIT9301_P9301_01791_ndhI
Prochlorococcus_marinus_CCMP_1986_PMM0159_ndhI
Prochlorococcus_marinus_MIT9315_P9515_01881_ndhI
Prochlorococcus_marinus_CCMP1375_Pro0183_ndhI
Prochlorococcus_marinus_MIT9211_P9211_01761_ndhI
Prochlorococcus_marinus_NATL2A_PMN2A_1526
Prochlorococcus_marinus_NATL1A_NATL1_02321_ndhI
Prochlorococcus_marinus_MIT9313_PMT2019_ndhI
Prochlorococcus_marinus_MIT9303_P9303_26891_ndhI
Synechococcus_sp._9605_Syncc9605_2411
Synechococcus_sp._9902_Syncc9902_0281
Synechococcus_sp._WH8102_SYNW2273_ndhI
Synechocossus_sp._WH7803_SynWH7803_2290
Synechococcus_sp._CC9311_sync_2624_ndhI
Synechococcus_sp._RCC307_SynRCC307_2278
Paulinella_chromatophora_CHR_PCC_0325_ndhI
Rhodopsudomonas_palustris_CGA009_RPA2942_nuoI1
Rhodospirillum_rubrum_ATCC_11170_Rru_A1563
Escherichia_coli_K-12_MG1655_b2281_nuoI
Clorobium_tepidum_CT0771_ndhI
0.90/0.95
0.62/1
0.58/1
0.56/0.94
0.56/1
0.52/0.78
0.51/0.84
1/1
1/1
0.6
NdhK (BI/ML)
Prochlorococcus_marinus_MIT9312_PMT9312_0295
Prochlorococcus_marinus_MIT9301_P9301_03171_ndhK
Prochlorococcus_marinus_AS9601_A9601_03161_ndhK
Prochlorococcus_marinus_MIT9215_P9215_03181_ndhK
Prochlorococcus_marinus_CCMP_1986_PMM0293_ndhK
Prochlorococcus_marinus_MIT9315_P9515_03261_ndhK
Prochlorococcus_marinus_NATL2A_PMN2A_1659
Prochlorococcus_marinus_NATL1A_NATL1_03731_ndhK
Prochlorococcus_marinus_CCMP1375_Pro0324_ndhK
Prochlorococcus_marinus_MIT9211_P9211_03191_ndhK
Synechocossus_sp._WH7803_SynWH7803_0253
Synechococcus_sp._CC9311_sync_0243
Synechococcus_sp._RCC307_SynRCC307_0214
Synechococcus_sp._WH8102_SYNW0208_ndhK
Synechococcus_sp._9902_Syncc9902_0231
Synechococcus_sp._9605_Syncc9605_0204
Prochlorococcus_marinus_MIT9313_PMT1892_ndhK
Prochlorococcus_marinus_MIT9303_P9303_25251_ndhK
Paulinella_chromatophora_CHR_PCC_0495_ndhK
Synechococcus_sp._PCC_6301_syc0369_c_ndhK
Anabaena_sp._PCC_7120_all3841_ndhK
Anabaena_variabilis_ATCC_29413_Ava_1859
Nostoc_punctiforme_sp._PCC73102_NpR5547
Trichodesmium_erythraeum__405_1_Tery_3500
Crocosphaera_watsonii_WH8501_g730
Synechococcus_sp._PCC7002_SYNPCC7002_A2749_ndhK
Synechocystis_sp._PCC_6803_slr1280_ndhK
Microcystis_aeruginosa_NIES843_MAE_11770_ndhK
Acaryochloris_marina_MBIC11017_AM1_1371_ndhK
Thermosynechococcus_elongatus_tlr0705_ndhK
Staurastrum_punctulatum_Cp_StpuCp021_ndhK
Zygnema_circumcarinatum_ZyciCp077_ndhK
Chaetospheridium_globosum_Cp_ndhK
Mesostigma_viride_Cp_ndhK
Chlorokybus_atmophyticus_Cp_ChatCp055_ndhK
Nephroselmis_olivacea_Cp_ndhK
Gloeobacter_violaceus_gvip081_ndhK
Cyanobacterium_Yellowstone_A-prime_CYA_1995
Cyanobacterium_Yellowstone_B-prime_CYB_2333
Rhodopsudomonas_palustris_CGA009_RPA2951_nuoB1
Rhodospirillum_rubrum_ATCC_11170_Rru_A1556
Clorobium_tepidum_CT0767_ndhH
Escherichia_coli_K-12_MG1655_b2287_nuoB
0.90/1
1/1
0.66/--
0.99/1
0.58/0.76
0.96/0.93
1/1
1/1
0.77/0.99
1/1
0.5

NDH all subunits
(BI/ML)

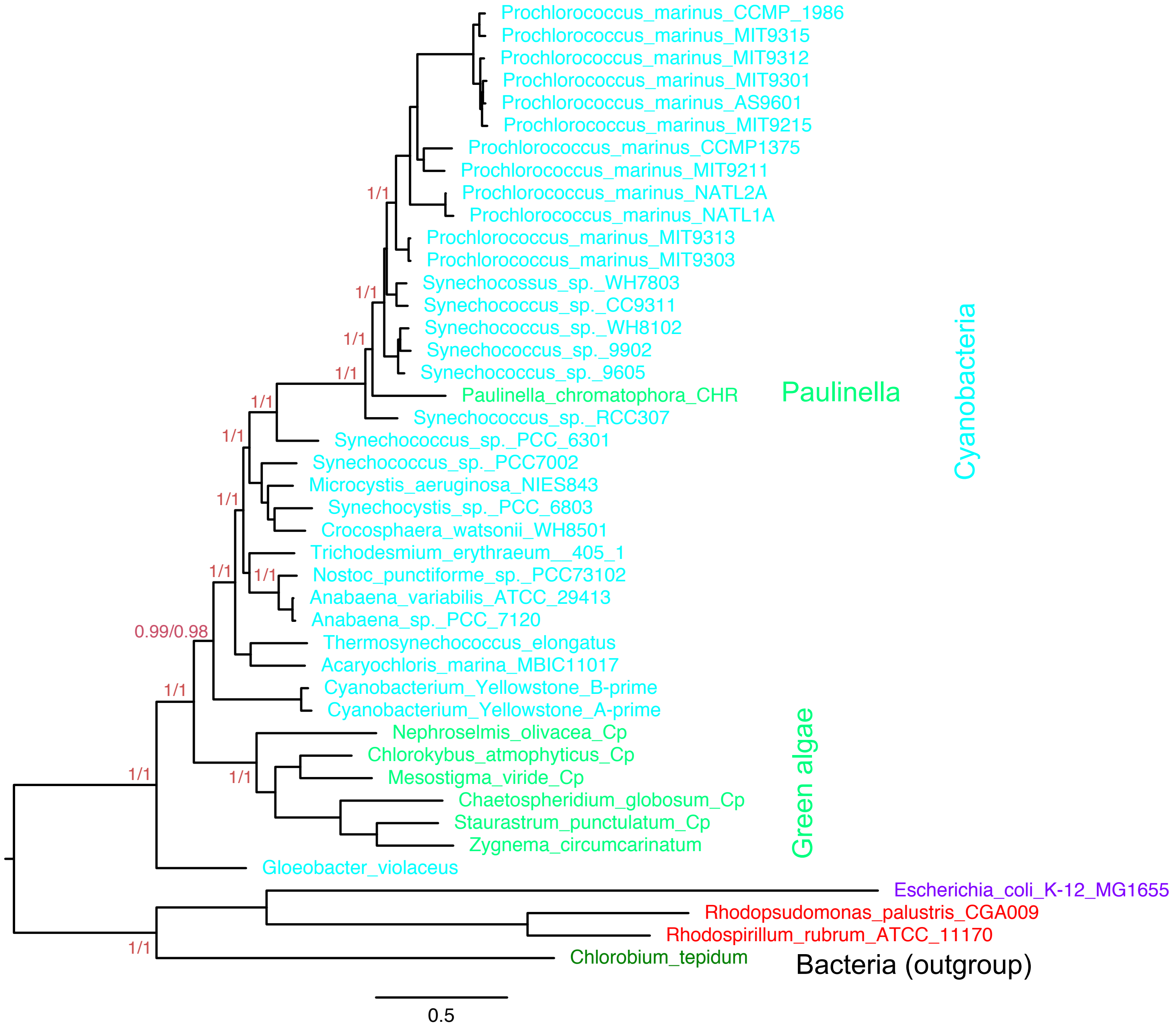

NdhD
(BI/ML)

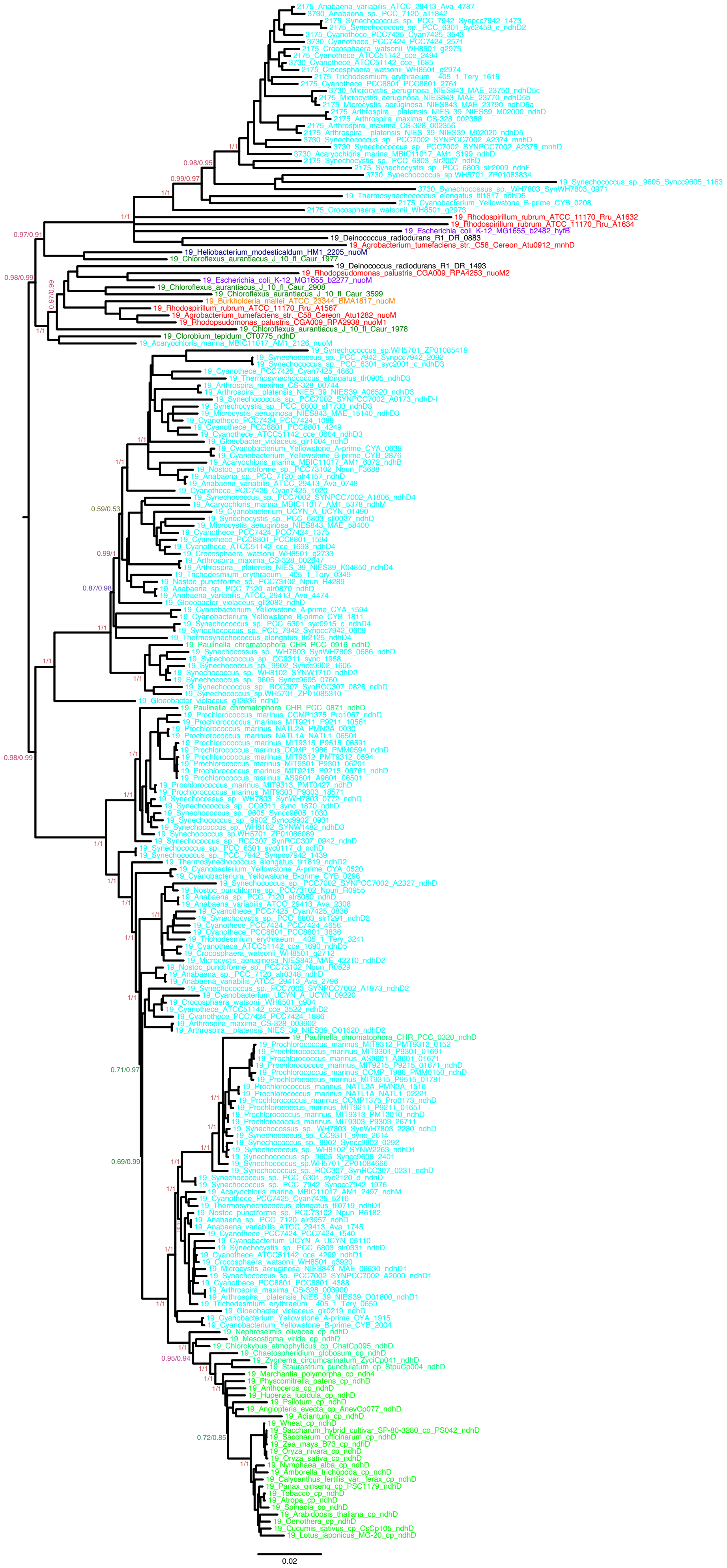

NdhJ
(BI/ML)

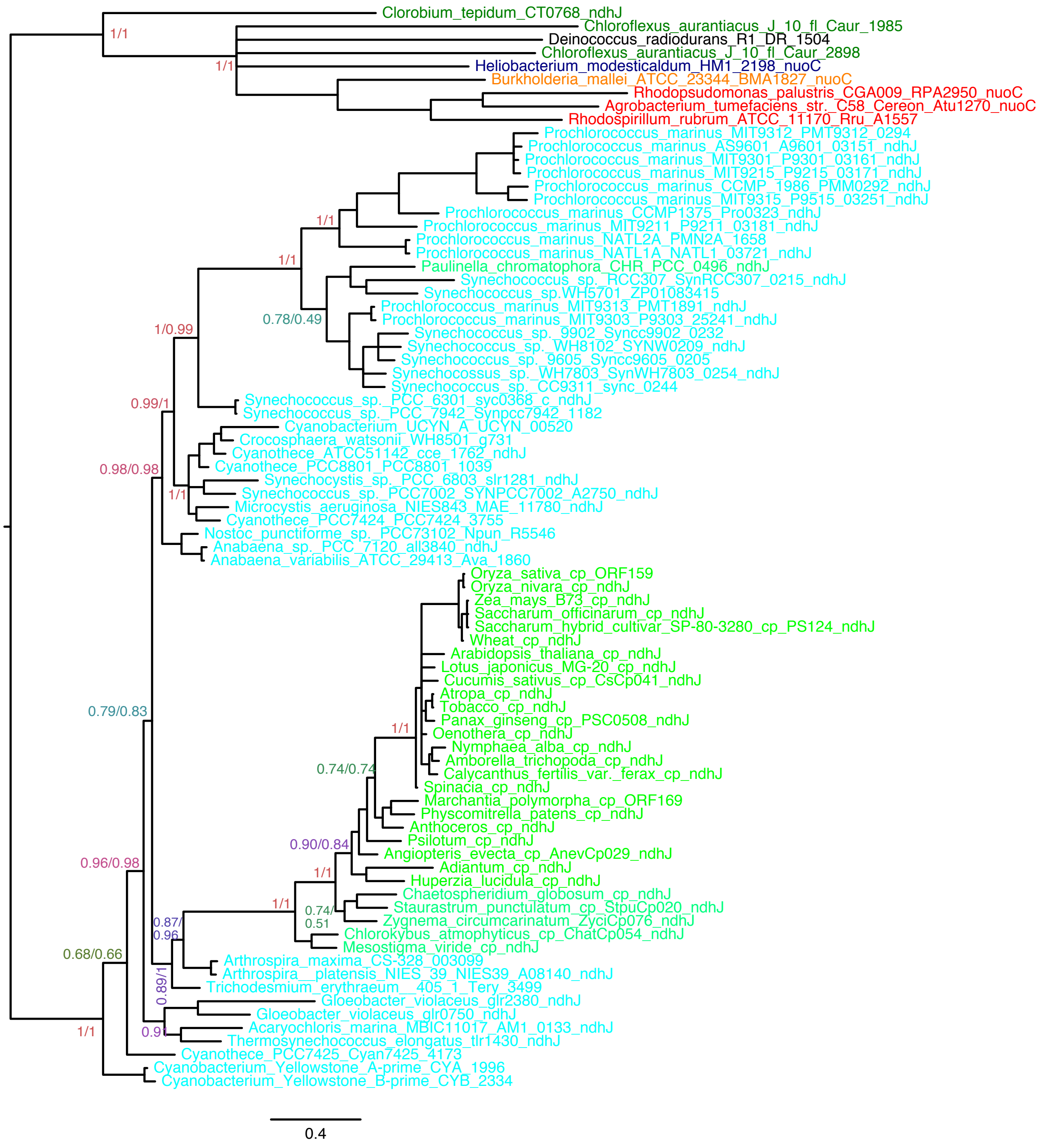

Clorobium_tepidum_CT0768_ndhJ
Chloroflexus_aurantiacus_J_10_fl_Caur_1985
Deinococcus_radiodurans_R1_DR_1504
Chloroflexus_aurantiacus_J_10_fl_Caur_2898
Heliobacterium_modesticaldum_HM1_2198_nuoC
Burkholderia_mallei_ATCC_23344_BMA1827_nuoC
Rhodopsudomonas_palustris_CGA009_RPA2950_nuoC
Agrobacterium_tumefaciens_str._C58_Cereon_Atu1270_nuoC
Rhodospirillum_rubrum_ATCC_11170_Rru_A1557
Prochlorococcus_marinus_MIT9312_PMT9312_0294
Prochlorococcus_marinus_AS9601_A9601_03151_ndhJ
Prochlorococcus_marinus_MIT9301_P9301_03161_ndhJ
Prochlorococcus_marinus_MIT9215_P9215_03171_ndhJ
Prochlorococcus_marinus_CCMP_1986_PMM0292_ndhJ
Prochlorococcus_marinus_MIT9315_P9515_03251_ndhJ
Prochlorococcus_marinus_CCMP1375_Pro0323_ndhJ
Prochlorococcus_marinus_MIT9211_P9211_03181_ndhJ
Prochlorococcus_marinus_NATL2A_PMN2A_1658
Prochlorococcus_marinus_NATL1A_NATL1_03721_ndhJ
Paulinella_chromatophora_CHR_PCC_0496_ndhJ
Synechococcus_sp._RCC307_SynRCC307_0215_ndhJ
Synechococcus_sp.WH5701_ZP01083415
Prochlorococcus_marinus_MIT9313_PMT1891_ndhJ
Prochlorococcus_marinus_MIT9303_P9303_25241_ndhJ
Synechococcus_sp._9902_Syncc9902_0232
Synechococcus_sp._WH8102_SYNW0209_ndhJ
Synechococcus_sp._9605_Syncc9605_0205
Synechocossus_sp._WH7803_SynWH7803_0254_ndhJ
Synechococcus_sp._CC9311_sync_0244
Synechococcus_sp._PCC_6301_syc0368_c_ndhJ
Synechococcus_sp._PCC_7942_Synpcc7942_1182
Cyanobacterium_UCYN_A_UCYN_00520
Crocosphaera_watsonii_WH8501_g731
Cyanothece_ATCC51142_cce_1762_ndhJ
Cyanothece_PCC8801_PCC8801_1039
Synechocystis_sp._PCC_6803_slr1281_ndhJ
Synechococcus_sp._PCC7002_SYNPCC7002_A2750_ndhJ
Microcystis_aeruginosa_NIES843_MAE_11780_ndhJ
Cyanothece_PCC7424_PCC7424_3755
Nostoc_punctiforme_sp._PCC73102_Npun_R5546
Anabaena_sp._PCC_7120_all3840_ndhJ
Anabaena_variabilis_ATCC_29413_Ava_1860
Oryza_sativa_cp_ORF159
Oryza_nivara_cp_ndhJ
Zea_mays_B73_cp_ndhJ
Saccharum_officinarum_cp_ndhJ
Saccharum_hybrid_cultivar_SP-80-3280_cp_PS124_ndhJ
Wheat_cp_ndhJ
Arabidopsis_thaliana_cp_ndhJ
Lotus_japonicus_MG-20_cp_ndhJ
Cucumis_sativus_cp_CsCp041_ndhJ
Atropa_cp_ndhJ
Tobacco_cp_ndhJ
Panax_ginseng_cp_PSC0508_ndhJ
Oenothera_cp_ndhJ
Nymphaea_alba_cp_ndhJ
Amborella_trichopoda_cp_ndhJ
Calycanthus_fertilis_var._ferax_cp_ndhJ
Spinacia_cp_ndhJ
Marchantia_polymorpha_cp_ORF169
Physcomitrella_patens_cp_ndhJ
Anthoceros_cp_ndhJ
Psilotum_cp_ndhJ
Angiopteris_evecta_cp_AnevCp029_ndhJ
Adiantum_cp_ndhJ
Huperzia_lucidula_cp_ndhJ
Chaetospheridium_globosum_cp_ndhJ
Staurastrum_punctulatum_cp_StpuCp020_ndhJ
Zygnema_circumcarinatum_ZyciCp076_ndhJ
Chlorokybus_atmophyticus_cp_ChatCp054_ndhJ
Mesostigma_viride_cp_ndhJ
Arthrospira_maxima_CS-328_003099
Arthrospira__platensis_NIES_39_NIES39_A08140_ndhJ
Trichodesmium_erythraeum__405_1_Tery_3499
Gloeobacter_violaceus_glr2380_ndhJ
Gloeobacter_violaceus_glr0750_ndhJ
Acaryochloris_marina_MBIC11017_AM1_0133_ndhJ
Thermosynechococcus_elongatus_tlr1430_ndhJ
Cyanothece_PCC7425_Cyan7425_4173
Cyanobacterium_Yellowstone_A-prime_CYA_1996
Cyanobacterium_Yellowstone_B-prime_CYB_2334
1/1
1/1
1/1
1/1
1/0.99
0.78/0.49
0.99/1
0.98/0.98
1/1
0.79/0.83
1/1
0.74/0.74
0.90/0.84
1/1
0.96/0.98
1/1
0.74/
0.51
0.87/
0.96
0.68/0.66
0.89/1
0.91
1/1
0.4
