# Supplementary material for: Complex origins of chloroplast membranes with photosynthetic machineries: multiple transfers of genes from divergent organisms at different times or a single endosymbiotic event?
Source: J Plant Res. 2019 Dec 6;133(1):15–33. doi: 10.1007/s10265-019-01157-z (PMC6946739; doi:10.1007/s10265-019-01157-z)
Supplement: Supplementary file 6 — Supplementary material 6 (PDF 1105 kb) [file 10265_2019_1157_MOESM6_ESM.pdf]

Journal of Plant Research

Online Resources

Complex origins of chloroplast membranes with photosynthetic machineries: Multiple transfers of genes from divergent organisms at different times or a single endosymbiotic event?

Naoki Sato
University of Tokyo
naokisat@bio.c.u-tokyo.ac.jp

Online Resource 6. Phylogenetic trees of Calvin-Benson Cycle enzymes.

GAPDH, glyceraldehyde 3-phosphate dehydrogenase; PGK, phosphoglycerate kinase; PRK, phosphoribulokinase; RPE, ribose phosphate epimerase; TKL, transketolase. Locate the enzymes in the metabolic pathway in Fig. 3. Other chloroplast enzymes of Calvin-Benson Cycle are originated from eukaryotes, but not prokaryotes (Matsuzaki et al. 2004), and are not analyzed in the present study.

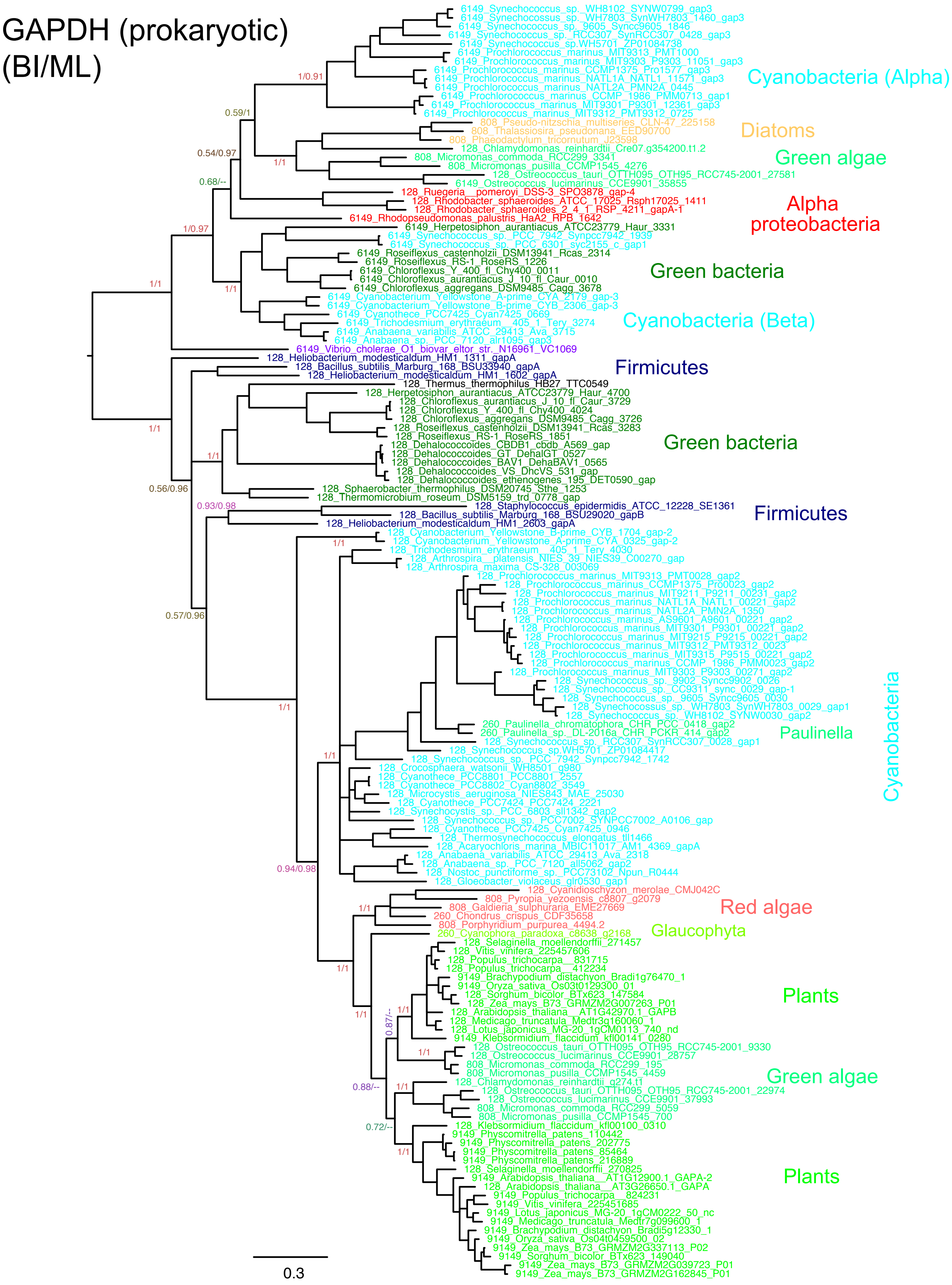

GAPDH (prokaryotic)
(BI/ML)
6149_Synechococcus_sp._WH8102_SYNW0799_gap3
6149_Synechocossus_sp._WH7803_SynWH7803_1460_gap3
6149_Synechococcus_sp._9605_Syncc9605_1846
6149_Synechococcus_sp._RCC307_SynRCC307_0428_gap3
6149_Synechococcus_sp.WH5701_ZP01084738
6149_Prochlorococcus_marinus_MIT9313_PMT1000
6149_Prochlorococcus_marinus_MIT9303_P9303_11051_gap3
6149_Prochlorococcus_marinus_CCMP1375_Pro1577_gap3
6149_Prochlorococcus_marinus_NATL1A_NATL1_11571_gap3
6149_Prochlorococcus_marinus_NATL2A_PMN2A_0445
6149_Prochlorococcus_marinus_CCMP_1986_PMM0713_gap1
6149_Prochlorococcus_marinus_MIT9301_P9301_12361_gap3
6149_Prochlorococcus_marinus_MIT9312_PMT9312_0725
Cyanobacteria (Alpha)
808_Pseudo-nitzschia_multiseries_CLN-47_225158
808_Thalassiosira_pseudonana_EED90700
808_Phaeodactylum_tricornutum_J23598
Diatoms
128_Chlamydomonas_reinhardtii_Cre07.g354200.t1.2
808_Micromonas_commoda_RCC299_3341
808_Micromonas_pusilla_CCMP1545_4276
128_Ostreococcus_tauri_OTTH095_OTH95_RCC745-2001_27581
6149_Ostreococcus_lucimarinus_CCE9901_35855
Green algae
128_Ruegeria_pomeroyi_DSS-3_SPO3878_gap-4
128_Rhodobacter_sphaeroides_ATCC_17025_Rsph17025_1411
128_Rhodobacter_sphaeroides_2_4_1_RSP_4211_gapA-1
6149_Rhodopseudomonas_palustris_HaA2_RPB_1642
Alpha proteobacteria
6149_Herpetosiphon_aurantiacus_ATCC23779_Haur_3331
6149_Synechococcus_sp._PCC_7942_Synpcc7942_1939
6149_Synechococcus_sp._PCC_6301_syc2155_c_gap1
6149_Roseiflexus_castenholzii_DSM13941_Rcas_2314
6149_Roseiflexus_RS-1_RoseRS_1226
6149_Chloroflexus_Y_400_fl_Chy400_0011
6149_Chloroflexus_aurantiacus_J_10_fl_Caur_0010
6149_Chloroflexus_aggregans_DSM9485_Cagg_3678
Green bacteria
6149_Cyanobacterium_Yellowstone_A-prime_CYA_2179_gap-3
6149_Cyanobacterium_Yellowstone_B-prime_CYB_2306_gap-3
6149_Cyanothece_PCC7425_Cyan7425_0669
6149_Trichodesmium_erythraeum_405_1_Tery_3274
6149_Anabaena_variabilis_ATCC_29413_Ava_3715
6149_Anabaena_sp._PCC_7120_alr1095_gap3
Cyanobacteria (Beta)
6149_Vibrio_cholerae_O1_biovar_eltor_str._N16961_VC1069
128_Heliobacterium_modesticaldum_HM1_1311_gapA
128_Bacillus_subtilis_Marburg_168_BSU33940_gapA
128_Heliobacterium_modesticaldum_HM1_1602_gapA
Firmicutes
128_Thermus_thermophilus_HB27_TTC0549
128_Herpetosiphon_aurantiacus_ATCC23779_Haur_4700
128_Chloroflexus_aurantiacus_J_10_fl_Caur_3729
128_Chloroflexus_Y_400_fl_Chy400_4024
128_Chloroflexus_aggregans_DSM9485_Cagg_3726
128_Roseiflexus_castenholzii_DSM13941_Rcas_3283
128_Roseiflexus_RS-1_RoseRS_1851
128_Dehalococcoides_CBDB1_cbdb_A569_gap
128_Dehalococcoides_GT_DehalGT_0527
128_Dehalococcoides_BAV1_DehaBAV1_0565
128_Dehalococcoides_VS_DhcVS_531_gap
128_Dehalococcoides_ethenogenes_195_DET0590_gap
Green bacteria
128_Sphaerobacter_thermophilus_DSM20745_Sthe_1253
128_Thermomicrobium_roseum_DSM5159_trd_0778_gap
128_Staphylococcus_epidermidis_ATCC_12228_SE1361
128_Bacillus_subtilis_Marburg_168_BSU29020_gapB
128_Heliobacterium_modesticaldum_HM1_2603_gapA
Firmicutes
128_Cyanobacterium_Yellowstone_B-prime_CYB_1704_gap-2
128_Cyanobacterium_Yellowstone_A-prime_CYA_0325_gap-2
128_Trichodesmium_erythraeum_405_1_Tery_4030
128_Arthrospira_platensis_NIES_39_NIES39_C00270_gap
128_Arthrospira_maxima_CS-328_003069
128_Prochlorococcus_marinus_MIT9313_PMT0028_gap2
128_Prochlorococcus_marinus_CCMP1375_Pro0023_gap2
128_Prochlorococcus_marinus_MIT9211_P9211_00231_gap2
128_Prochlorococcus_marinus_NATL1A_NATL1_00221_gap2
128_Prochlorococcus_marinus_NATL2A_PMN2A_1350
128_Prochlorococcus_marinus_AS9601_A9601_00221_gap2
128_Prochlorococcus_marinus_MIT9301_P9301_00221_gap2
128_Prochlorococcus_marinus_MIT9215_P9215_00221_gap2
128_Prochlorococcus_marinus_MIT9312_PMT9312_0023
128_Prochlorococcus_marinus_MIT9315_P9515_00221_gap2
128_Prochlorococcus_marinus_CCMP_1986_PMM0023_gap2
128_Prochlorococcus_marinus_MIT9303_P9303_00271_gap2
128_Synechococcus_sp._9902_Syncc9902_0026
128_Synechococcus_sp._CC9311_sync_0029_gap-1
128_Synechococcus_sp._9605_Syncc9605_0030
128_Synechocossus_sp._WH7803_SynWH7803_0029_gap1
128_Synechococcus_sp._WH8102_SYNW0030_gap2
260_Paulinella_chromatophora_CHR_PCC_0418_gap2
260_Paulinella_sp._DL-2016a_CHR_PCKR_414_gap2
Paulinella
128_Synechococcus_sp._RCC307_SynRCC307_0028_gap1
128_Synechococcus_sp.WH5701_ZP01084417
128_Synechococcus_sp._PCC_7942_Synpcc7942_1742
128_Crocosphaera_watsonii_WH8501_g980
128_Cyanothece_PCC8801_PCC8801_2557
128_Cyanothece_PCC8802_Cyan8802_3549
128_Microcystis_aeruginosa_NIES843_MAE_25030
128_Cyanothece_PCC7424_PCC7424_2221
128_Synechocystis_sp._PCC_6803_sll1342_gap2
128_Synechococcus_sp._PCC7002_SYNPCC7002_A0106_gap
128_Cyanothece_PCC7425_Cyan7425_0946
128_Thermosynechococcus_elongatus_tll1466
128_Acaryochloris_marina_MBIC11017_AM1_4369_gapA
128_Anabaena_variabilis_ATCC_29413_Ava_2318
128_Anabaena_sp._PCC_7120_all5062_gap2
128_Nostoc_punctiforme_sp._PCC73102_Npun_R0444
128_Gloeobacter_violaceus_glr0530_gap1
Cyanobacteria
128_Cyanidioschyzon_merolae_CMJ042C
808_Pyropia_yezoensis_c8807_g2079
808_Galdieria_sulphuraria_EME27669
260_Chondrus_crispus_CDF35658
808_Porphyridium_purpurea_4494.2
Red algae
260_Cyanophora_paradoxa_c8638_g2168
Glaucophyta
128_Selaginella_moellendorffii_271457
128_Vitis_vinifera_225457606
128_Populus_trichocarpa_831715
128_Populus_trichocarpa_412234
9149_Brachypodium_distachyon_Bradi1g76470_1
9149_Oryza_sativa_Os03t0129300_01
128_Sorghum_bicolor_BTx623_147584
128_Zea_mays_B73_GRMZM2G007263_P01
128_Arabidopsis_thaliana_AT1G42970.1_GAPB
128_Medicago_truncatula_Medtr3g160060_1
128_Lotus_japonicus_MG-20_1gCM0113_740_nd
9149_Klebsormidium_flaccidum_kfl00141_0280
Plants
128_Ostreococcus_tauri_OTTH095_OTH95_RCC745-2001_9330
128_Ostreococcus_lucimarinus_CCE9901_28757
808_Micromonas_commoda_RCC299_195
808_Micromonas_pusilla_CCMP1545_4459
Green algae
128_Chlamydomonas_reinhardtii_g274.t1
128_Ostreococcus_tauri_OTTH095_OTH95_RCC745-2001_22974
128_Ostreococcus_lucimarinus_CCE9901_37993
808_Micromonas_commoda_RCC299_5059
808_Micromonas_pusilla_CCMP1545_700
128_Klebsormidium_flaccidum_kfl00100_0310
9149_Physcomitrella_patens_110442
9149_Physcomitrella_patens_202775
9149_Physcomitrella_patens_85464
9149_Physcomitrella_patens_216889
128_Selaginella_moellendorffii_270825
9149_Arabidopsis_thaliana_AT1G12900.1_GAPA-2
128_Arabidopsis_thaliana_AT3G26650.1_GAPA
9149_Populus_trichocarpa_824231
9149_Vitis_vinifera_225451685
9149_Lotus_japonicus_MG-20_1gCM0222_50_nc
9149_Medicago_truncatula_Medtr7g099600_1
9149_Brachypodium_distachyon_Bradi5g12330_1
9149_Oryza_sativa_Os04t0459500_02
9149_Zea_mays_B73_GRMZM2G337113_P02
9149_Sorghum_bicolor_BTx623_149040
9149_Zea_mays_B73_GRMZM2G039723_P01
9149_Zea_mays_B73_GRMZM2G162845_P01
Plants
0.3

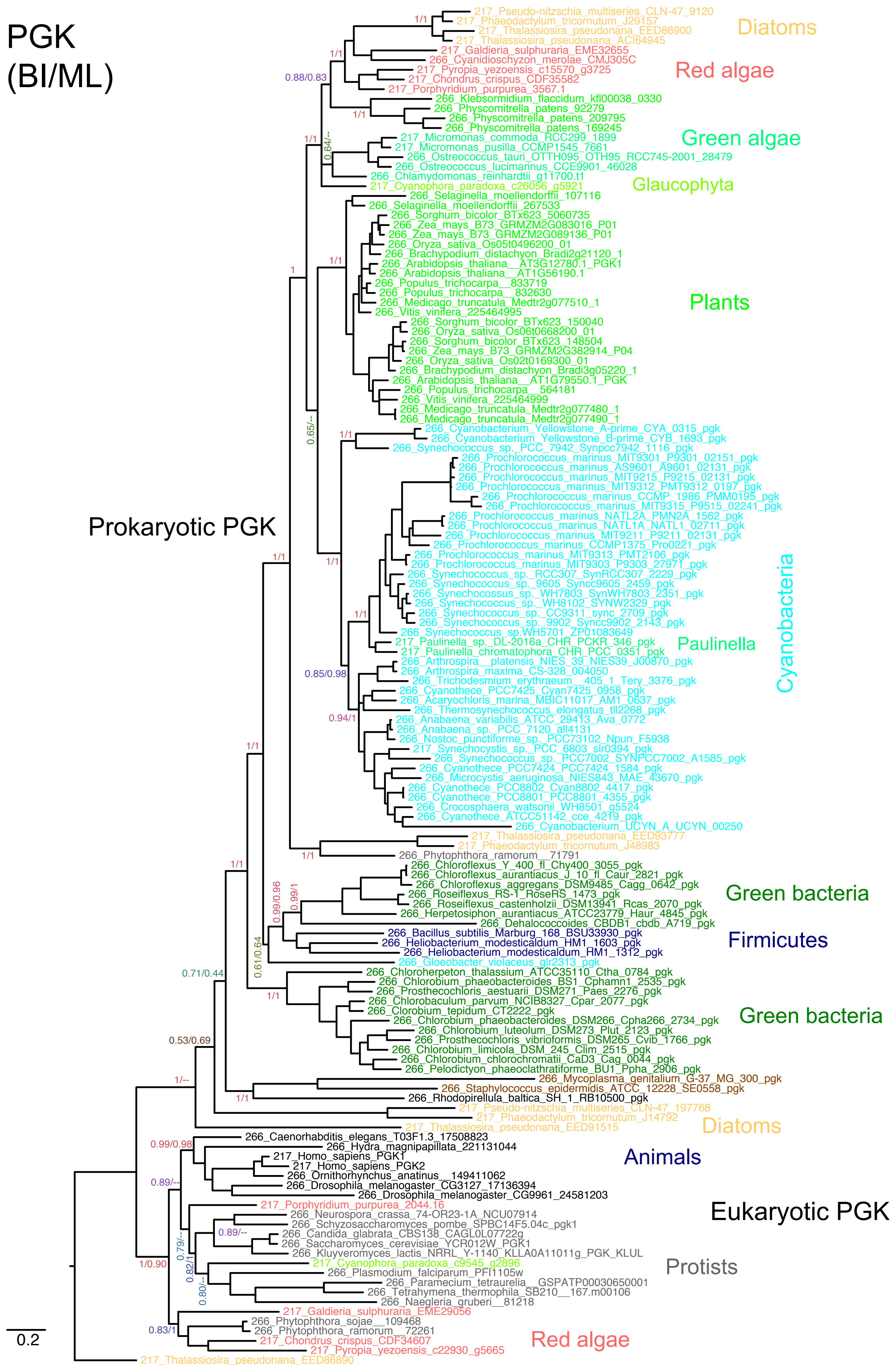
PGK
(BI/ML)
217_Pseudo-nitzschia_multiseries_CLN-47_9120
217_Phaeodactylum_tricornutum_J29157
217_Thalassiosira_pseudonana_EED86900
217_Thalassiosira_pseudonana_ACI64945
217_Galdieria_sulphuraria_EME32655
266_Cyanidioschyzon_merolae_CMJ305C
217_Pyropia_yezoensis_c15570_g3725
217_Chondrus_crispus_CDF35582
217_Porphyridium_purpurea_3567.1
266_Klebsormidium_flaccidum_kfl00038_0330
266_Physcomitrella_patens_92279
266_Physcomitrella_patens_209795
266_Physcomitrella_patens_169245
217_Micromonas_commoda_RCC299_1899
217_Micromonas_pusilla_CCMP1545_7661
266_Ostreococcus_tauri_OTTH095_OTH95_RCC745-2001_28479
266_Ostreococcus_lucimarinus_CCE9901_46028
266_Chlamydomonas_reinhardtii_g11700.t1
217_Cyanophora_paradoxa_c26056_g5921
266_Selaginella_moellendorffii_107116
266_Selaginella_moellendorffii_267533
266_Sorghum_bicolor_BTx623_5060735
266_Zea_mays_B73_GRMZM2G083016_P01
266_Zea_mays_B73_GRMZM2G089136_P01
266_Oryza_sativa_Os05t0496200_01
266_Brachypodium_distachyon_Bradi2g21120_1
266_Arabidopsis_thaliana__AT3G12780.1_PGK1
266_Arabidopsis_thaliana__AT1G56190.1
266_Populus_trichocarpa__833719
266_Populus_trichocarpa__832630
266_Medicago_truncatula_Medtr2g077510_1
266_Vitis_vinifera_225464995
266_Sorghum_bicolor_BTx623_150040
266_Oryza_sativa_Os06t0668200_01
266_Sorghum_bicolor_BTx623_148504
266_Zea_mays_B73_GRMZM2G382914_P04
266_Oryza_sativa_Os02t0169300_01
266_Brachypodium_distachyon_Bradi3g05220_1
266_Arabidopsis_thaliana__AT1G79550.1_PGK
266_Populus_trichocarpa__564181
266_Vitis_vinifera_225464999
266_Medicago_truncatula_Medtr2g077480_1
266_Medicago_truncatula_Medtr2g077490_1
266_Cyanobacterium_Yellowstone_A-prime_CYA_0315_pgk
266_Cyanobacterium_Yellowstone_B-prime_CYB_1693_pgk
266_Synechococcus_sp._PCC_7942_Synpcc7942_1116_pgk
266_Prochlorococcus_marinus_MIT9301_P9301_02151_pgk
266_Prochlorococcus_marinus_AS9601_A9601_02131_pgk
266_Prochlorococcus_marinus_MIT9215_P9215_02131_pgk
266_Prochlorococcus_marinus_MIT9312_PMT9312_0197_pgk
266_Prochlorococcus_marinus_CCMP_1986_PMM0195_pgk
266_Prochlorococcus_marinus_MIT9315_P9515_02241_pgk
266_Prochlorococcus_marinus_NATL2A_PMN2A_1562_pgk
266_Prochlorococcus_marinus_NATL1A_NATL1_02711_pgk
266_Prochlorococcus_marinus_MIT9211_P9211_02131_pgk
266_Prochlorococcus_marinus_CCMP1375_Pro0221_pgk
266_Prochlorococcus_marinus_MIT9313_PMT2106_pgk
266_Prochlorococcus_marinus_MIT9303_P9303_27971_pgk
266_Synechococcus_sp._RCC307_SynRCC307_2229_pgk
266_Synechococcus_sp._9605_Syncc9605_2459_pgk
266_Synechocossus_sp._WH7803_SynWH7803_2351_pgk
266_Synechococcus_sp._WH8102_SYNW2329_pgk
266_Synechococcus_sp._CC9311_sync_2709_pgk
266_Synechococcus_sp._9902_Syncc9902_2143_pgk
266_Synechococcus_sp.WH5701_ZP01083649
217_Paulinella_sp._DL-2016a_CHR_PCKR_346_pgk
217_Paulinella_chromatophora_CHR_PCC_0351_pgk
266_Arthrospira__platensis_NIES_39_NIES39_J00870_pgk
266_Arthrospira_maxima_CS-328_004050
266_Trichodesmium_erythraeum__405_1_Tery_3376_pgk
266_Cyanothece_PCC7425_Cyan7425_0958_pgk
266_Acaryochloris_marina_MBIC11017_AM1_0637_pgk
266_Thermosynechococcus_elongatus_tll2268_pgk
266_Anabaena_variabilis_ATCC_29413_Ava_0772
266_Anabaena_sp._PCC_7120_all4131
266_Nostoc_punctiforme_sp._PCC73102_Npun_F5938
217_Synechocystis_sp._PCC_6803_slr0394_pgk
266_Synechococcus_sp._PCC7002_SYNPCC7002_A1585_pgk
266_Cyanothece_PCC7424_PCC7424_1584_pgk
266_Microcystis_aeruginosa_NIES843_MAE_43670_pgk
266_Cyanothece_PCC8802_Cyan8802_4417_pgk
266_Cyanothece_PCC8801_PCC8801_4355_pgk
266_Crocosphaera_watsonii_WH8501_g5524
266_Cyanothece_ATCC51142_cce_4219_pgk
266_Cyanobacterium_UCYN_A_UCYN_00250
217_Thalassiosira_pseudonana_EED93777
217_Phaeodactylum_tricornutum_J48983
266_Phytophthora_ramorum__71791
266_Chloroflexus_Y_400_fl_Chy400_3055_pgk
266_Chloroflexus_aurantiacus_J_10_fl_Caur_2821_pgk
266_Chloroflexus_aggregans_DSM9485_Cagg_0642_pgk
266_Roseiflexus_RS-1_RoseRS_1473_pgk
266_Roseiflexus_castenholzii_DSM13941_Rcas_2070_pgk
266_Herpetosiphon_aurantiacus_ATCC23779_Haur_4845_pgk
266_Dehalococcoides_CBDB1_cbdb_A719_pgk
266_Bacillus_subtilis_Marburg_168_BSU33930_pgk
266_Heliobacterium_modesticaldum_HM1_1603_pgk
266_Heliobacterium_modesticaldum_HM1_1312_pgk
266_Gloeobacter_violaceus_glr2313_pgk
266_Chloroherpeton_thalassium_ATCC35110_Ctha_0784_pgk
266_Chlorobium_phaeobacteroides_BS1_Cphamn1_2535_pgk
266_Prosthecochloris_aestuarii_DSM271_Paes_2276_pgk
266_Chlorobaculum_parvum_NCIB8327_Cpar_2077_pgk
266_Clorobium_tepidum_CT2222_pgk
266_Chlorobium_phaeobacteroides_DSM266_Cpha266_2734_pgk
266_Chlorobium_luteolum_DSM273_Plut_2123_pgk
266_Prosthecochloris_vibrioformis_DSM265_Cvib_1766_pgk
266_Chlorobium_limicola_DSM_245_Clim_2515_pgk
266_Chlorobium_chlorochromatii_CaD3_Cag_0044_pgk
266_Pelodictyon_phaeoclathratiforme_BU1_Ppha_2906_pgk
266_Mycoplasma_genitalium_G-37_MG_300_pgk
266_Staphylococcus_epidermidis_ATCC_12228_SE0558_pgk
266_Rhodopirellula_baltica_SH_1_RB10500_pgk
217_Pseudo-nitzschia_multiseries_CLN-47_197768
217_Phaeodactylum_tricornutum_J14792
217_Thalassiosira_pseudonana_EED91515
266_Caenorhabditis_elegans_T03F1.3_17508823
266_Hydra_magnipapillata_221131044
217_Homo_sapiens_PGK1
217_Homo_sapiens_PGK2
266_Ornithorhynchus_anatinus__149411062
266_Drosophila_melanogaster_CG3127_17136394
266_Drosophila_melanogaster_CG9961_24581203
217_Porphyridium_purpurea_2044.16
266_Neurospora_crassa_74-OR23-1A_NCU07914
266_Schyzosaccharomyces_pombe_SPBC14F5.04c_pgk1
266_Candida_glabrata_CBS138_CAGL0L07722g
266_Saccharomyces_cerevisiae_YCR012W_PGK1
266_Kluyveromyces_lactis_NRRL_Y-1140_KLLA0A11011g_PGK_KLUL
217_Cyanophora_paradoxa_c9545_g2896
266_Plasmodium_falciparum_PFI1105w
266_Paramecium_tetraurelia__GSPATP00030650001
266_Tetrahymena_thermophila_SB210__167.m00106
266_Naegleria_gruberi__81218
217_Galdieria_sulphuraria_EME29056
266_Phytophthora_sojae__109468
266_Phytophthora_ramorum__72261
217_Chondrus_crispus_CDF34607
217_Pyropia_yezoensis_c22930_g5665
217_Thalassiosira_pseudonana_EED86890
Diatoms
Red algae
Green algae
Glaucophyta
Plants
Prokaryotic PGK
Cyanobacteria
Paulinella
Green bacteria
Firmicutes
Green bacteria
Diatoms
Animals
Eukaryotic PGK
Protists
Red algae
1/1
1/1
0.88/0.83
1/1
1/1
0.64/--
1
1/1
0.65/--
1/1
1/1
1/1
1/1
0.85/0.98
0.94/1
1/1
1/1
1/1
0.99/0.96
0.99/1
0.61/0.64
1/1
0.71/0.44
0.53/0.69
1/--
1/1
0.99/0.98
0.89/--
0.89/--
0.79/--
0.82/1
0.80/--
1/0.90
0.83/1
0.2

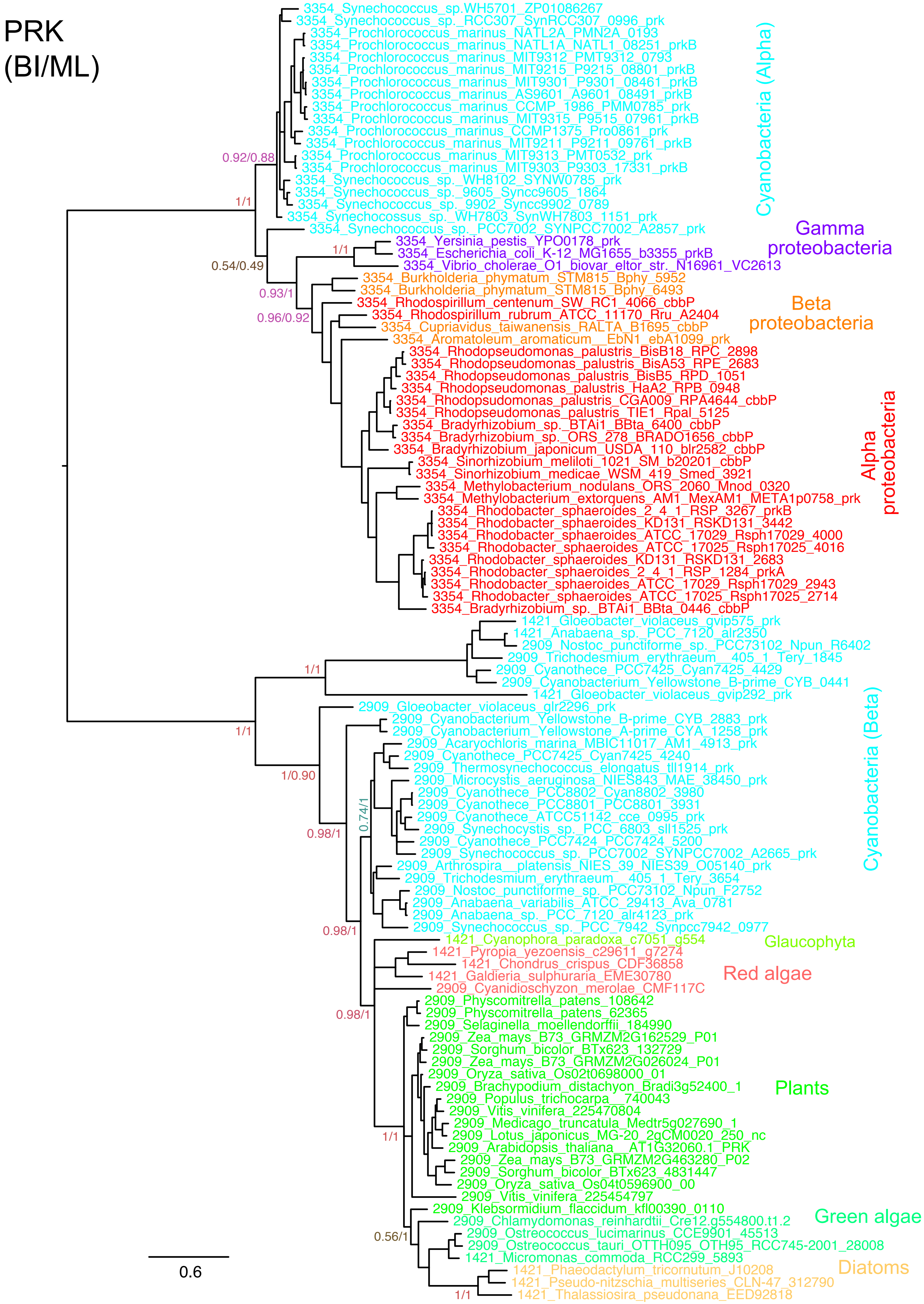
PRK
(BI/ML)
3354_Synechococcus_sp.WH5701_ZP01086267
3354_Synechococcus_sp._RCC307_SynRCC307_0996_prk
3354_Prochlorococcus_marinus_NATL2A_PMN2A_0193
3354_Prochlorococcus_marinus_NATL1A_NATL1_08251_prkB
3354_Prochlorococcus_marinus_MIT9312_PMT9312_0793
3354_Prochlorococcus_marinus_MIT9215_P9215_08801_prkB
3354_Prochlorococcus_marinus_MIT9301_P9301_08461_prkB
3354_Prochlorococcus_marinus_AS9601_A9601_08491_prkB
3354_Prochlorococcus_marinus_CCMP_1986_PMM0785_prk
3354_Prochlorococcus_marinus_MIT9315_P9515_07961_prkB
3354_Prochlorococcus_marinus_CCMP1375_Pro0861_prk
3354_Prochlorococcus_marinus_MIT9211_P9211_09761_prkB
3354_Prochlorococcus_marinus_MIT9313_PMT0532_prk
3354_Prochlorococcus_marinus_MIT9303_P9303_17331_prkB
3354_Synechococcus_sp._WH8102_SYNW0785_prk
3354_Synechococcus_sp._9605_Syncc9605_1864
3354_Synechococcus_sp._9902_Syncc9902_0789
3354_Synechocossus_sp._WH7803_SynWH7803_1151_prk
3354_Synechococcus_sp._PCC7002_SYNPCC7002_A2857_prk
0.92/0.88
1/1
0.54/0.49
Cyanobacteria (Alpha)
3354_Yersinia_pestis_YPO0178_prk
3354_Escherichia_coli_K-12_MG1655_b3355_prkB
3354_Vibrio_cholerae_O1_biovar_eltor_str._N16961_VC2613
1/1
Gamma
proteobacteria
3354_Burkholderia_phymatum_STM815_Bphy_5952
3354_Burkholderia_phymatum_STM815_Bphy_6493
3354_Rhodospirillum_centenum_SW_RC1_4066_cbbP
3354_Rhodospirillum_rubrum_ATCC_11170_Rru_A2404
3354_Cupriavidus_taiwanensis_RALTA_B1695_cbbP
3354_Aromatoleum_aromaticum__EbN1_ebA1099_prk
0.93/1
0.96/0.92
Beta
proteobacteria
3354_Rhodopseudomonas_palustris_BisB18_RPC_2898
3354_Rhodopseudomonas_palustris_BisA53_RPE_2683
3354_Rhodopseudomonas_palustris_BisB5_RPD_1051
3354_Rhodopseudomonas_palustris_HaA2_RPB_0948
3354_Rhodopsudomonas_palustris_CGA009_RPA4644_cbbP
3354_Rhodopseudomonas_palustris_TIE1_Rpal_5125
3354_Bradyrhizobium_sp._BTAi1_BBta_6400_cbbP
3354_Bradyrhizobium_sp._ORS_278_BRADO1656_cbbP
3354_Bradyrhizobium_japonicum_USDA_110_blr2582_cbbP
3354_Sinorhizobium_meliloti_1021_SM_b20201_cbbP
3354_Sinorhizobium_medicae_WSM_419_Smed_3921
3354_Methylobacterium_nodulans_ORS_2060_Mnod_0320
3354_Methylobacterium_extorquens_AM1_MexAM1_META1p0758_prk
3354_Rhodobacter_sphaeroides_2_4_1_RSP_3267_prkB
3354_Rhodobacter_sphaeroides_KD131_RSKD131_3442
3354_Rhodobacter_sphaeroides_ATCC_17029_Rsph17029_4000
3354_Rhodobacter_sphaeroides_ATCC_17025_Rsph17025_4016
3354_Rhodobacter_sphaeroides_KD131_RSKD131_2683
3354_Rhodobacter_sphaeroides_2_4_1_RSP_1284_prkA
3354_Rhodobacter_sphaeroides_ATCC_17029_Rsph17029_2943
3354_Rhodobacter_sphaeroides_ATCC_17025_Rsph17025_2714
3354_Bradyrhizobium_sp._BTAi1_BBta_0446_cbbP
Alpha
proteobacteria
1421_Gloeobacter_violaceus_gvip575_prk
1421_Anabaena_sp._PCC_7120_alr2350
2909_Nostoc_punctiforme_sp._PCC73102_Npun_R6402
2909_Trichodesmium_erythraeum__405_1_Tery_1845
2909_Cyanothece_PCC7425_Cyan7425_4429
2909_Cyanobacterium_Yellowstone_B-prime_CYB_0441
1421_Gloeobacter_violaceus_gvip292_prk
1/1
1/1
2909_Gloeobacter_violaceus_glr2296_prk
2909_Cyanobacterium_Yellowstone_B-prime_CYB_2883_prk
2909_Cyanobacterium_Yellowstone_A-prime_CYA_1258_prk
2909_Acaryochloris_marina_MBIC11017_AM1_4913_prk
2909_Cyanothece_PCC7425_Cyan7425_4240
2909_Thermosynechococcus_elongatus_tll1914_prk
2909_Microcystis_aeruginosa_NIES843_MAE_38450_prk
2909_Cyanothece_PCC8802_Cyan8802_3980
2909_Cyanothece_PCC8801_PCC8801_3931
2909_Cyanothece_ATCC51142_cce_0995_prk
2909_Synechocystis_sp._PCC_6803_sll1525_prk
2909_Cyanothece_PCC7424_PCC7424_5200
2909_Synechococcus_sp._PCC7002_SYNPCC7002_A2665_prk
2909_Arthrospira__platensis_NIES_39_NIES39_O05140_prk
2909_Trichodesmium_erythraeum__405_1_Tery_3654
2909_Nostoc_punctiforme_sp._PCC73102_Npun_F2752
2909_Anabaena_variabilis_ATCC_29413_Ava_0781
2909_Anabaena_sp._PCC_7120_alr4123_prk
2909_Synechococcus_sp._PCC_7942_Synpcc7942_0977
1/0.90
0.98/1
0.74/1
0.98/1
Cyanobacteria (Beta)
1421_Cyanophora_paradoxa_c7051_g554
Glaucophyta
1421_Pyropia_yezoensis_c29611_g7274
1421_Chondrus_crispus_CDF36858
1421_Galdieria_sulphuraria_EME30780
2909_Cyanidioschyzon_merolae_CMF117C
Red algae
2909_Physcomitrella_patens_108642
2909_Physcomitrella_patens_62365
2909_Selaginella_moellendorffii_184990
2909_Zea_mays_B73_GRMZM2G162529_P01
2909_Sorghum_bicolor_BTx623_132729
2909_Zea_mays_B73_GRMZM2G026024_P01
2909_Oryza_sativa_Os02t0698000_01
2909_Brachypodium_distachyon_Bradi3g52400_1
2909_Populus_trichocarpa__740043
2909_Vitis_vinifera_225470804
2909_Medicago_truncatula_Medtr5g027690_1
2909_Lotus_japonicus_MG-20_2gCM0020_250_nc
2909_Arabidopsis_thaliana__AT1G32060.1_PRK
2909_Zea_mays_B73_GRMZM2G463280_P02
2909_Sorghum_bicolor_BTx623_4831447
2909_Oryza_sativa_Os04t0596900_00
2909_Vitis_vinifera_225454797
0.98/1
1/1
Plants
2909_Klebsormidium_flaccidum_kfl00390_0110
2909_Chlamydomonas_reinhardtii_Cre12.g554800.t1.2
2909_Ostreococcus_lucimarinus_CCE9901_45513
2909_Ostreococcus_tauri_OTTH095_OTH95_RCC745-2001_28008
1421_Micromonas_commoda_RCC299_5893
0.56/1
Green algae
1421_Phaeodactylum_tricornutum_J10208
1421_Pseudo-nitzschia_multiseries_CLN-47_312790
1421_Thalassiosira_pseudonana_EED92818
1/1
Diatoms
0.6

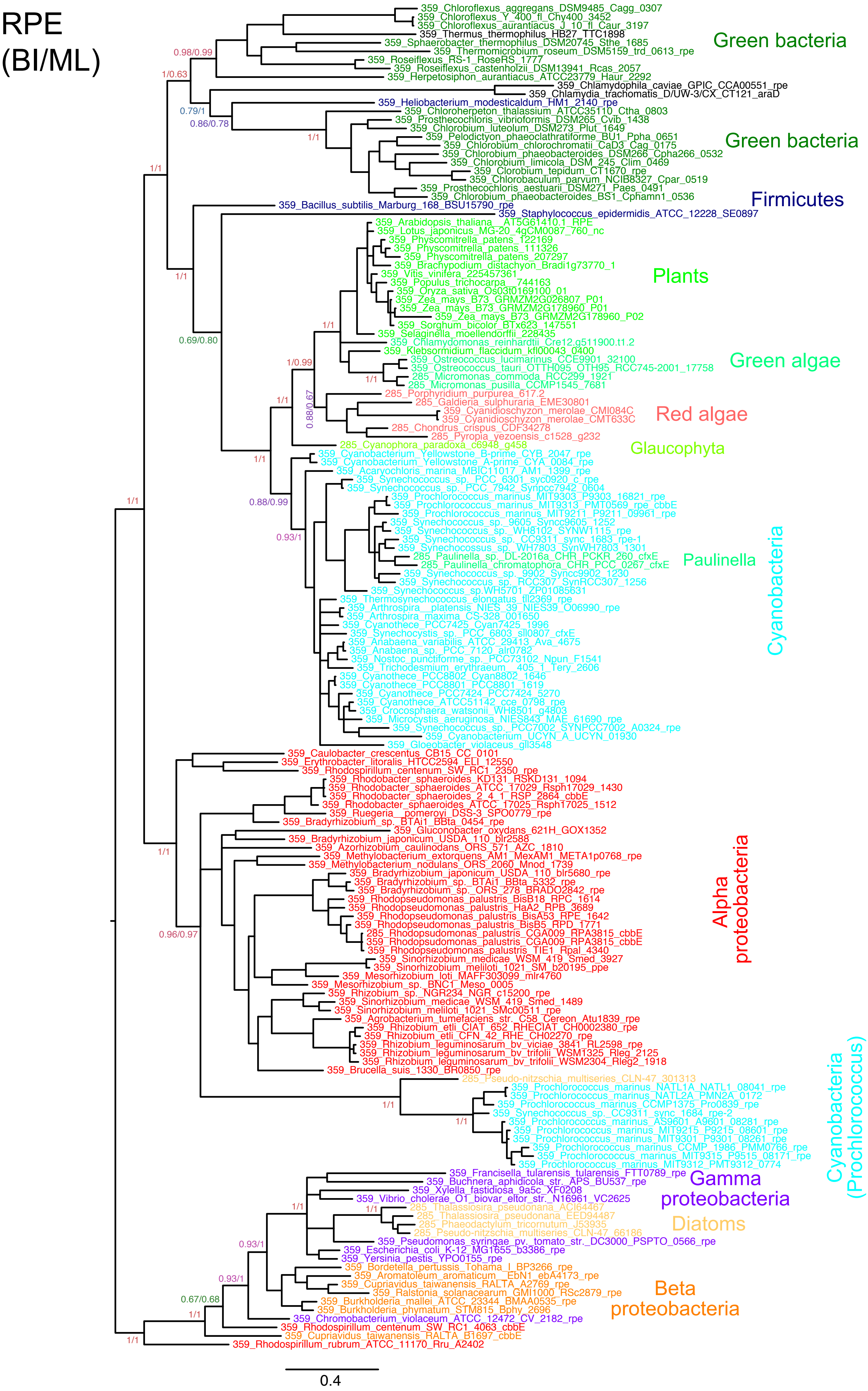
RPE
(BI/ML)
359_Chloroflexus_aggregans_DSM9485_Cagg_0307
359_Chloroflexus_Y_400_fl_Chy400_3452
359_Chloroflexus_aurantiacus_J_10_fl_Caur_3197
359_Thermus_thermophilus_HB27_TTC1898
359_Sphaerobacter_thermophilus_DSM20745_Sthe_1685
359_Thermomicrobium_roseum_DSM5159_trd_0613_rpe
359_Roseiflexus_RS-1_RoseRS_1777
359_Roseiflexus_castenholzii_DSM13941_Rcas_2057
359_Herpetosiphon_aurantiacus_ATCC23779_Haur_2292
Green bacteria
359_Chlamydophila_caviae_GPIC_CCA00551_rpe
359_Chlamydia_trachomatis_D/UW-3/CX_CT121_araD
359_Heliobacterium_modesticaldum_HM1_2140_rpe
359_Chloroherpeton_thalassium_ATCC35110_Ctha_0803
359_Prosthecochloris_vibrioformis_DSM265_Cvib_1438
359_Chlorobium_luteolum_DSM273_Plut_1649
359_Pelodictyon_phaeoclathratiforme_BU1_Ppha_0651
359_Chlorobium_chlorochromatii_CaD3_Cag_0175
359_Chlorobium_phaeobacteroides_DSM266_Cpha266_0532
359_Chlorobium_limicola_DSM_245_Clim_0469
359_Clorobium_tepidum_CT1670_rpe
359_Chlorobaculum_parvum_NCIB8327_Cpar_0519
359_Prosthecochloris_aestuarii_DSM271_Paes_0491
359_Chlorobium_phaeobacteroides_BS1_Cphamn1_0536
Green bacteria
359_Bacillus_subtilis_Marburg_168_BSU15790_rpe
359_Staphylococcus_epidermidis_ATCC_12228_SE0897
Firmicutes
359_Arabidopsis_thaliana__AT5G61410.1_RPE
359_Lotus_japonicus_MG-20_4gCM0087_760_nc
359_Physcomitrella_patens_122169
359_Physcomitrella_patens_111326
359_Physcomitrella_patens_207297
359_Brachypodium_distachyon_Bradi1g73770_1
359_Vitis_vinifera_225457361
359_Populus_trichocarpa__744163
359_Oryza_sativa_Os03t0169100_01
359_Zea_mays_B73_GRMZM2G026807_P01
359_Zea_mays_B73_GRMZM2G178960_P01
359_Zea_mays_B73_GRMZM2G178960_P02
359_Sorghum_bicolor_BTx623_147551
359_Selaginella_moellendorffii_228435
Plants
359_Chlamydomonas_reinhardtii_Cre12.g511900.t1.2
359_Klebsormidium_flaccidum_kfl00043_0400
359_Ostreococcus_lucimarinus_CCE9901_32100
359_Ostreococcus_tauri_OTTH095_OTH95_RCC745-2001_17758
285_Micromonas_commoda_RCC299_1921
285_Micromonas_pusilla_CCMP1545_7681
Green algae
285_Porphyridium_purpurea_617.2
285_Galdieria_sulphuraria_EME30801
359_Cyanidioschyzon_merolae_CMI084C
359_Cyanidioschyzon_merolae_CMT633C
285_Chondrus_crispus_CDF34278
285_Pyropia_yezoensis_c1528_g232
Red algae
285_Cyanophora_paradoxa_c6948_g458
Glaucophyta
359_Cyanobacterium_Yellowstone_B-prime_CYB_2047_rpe
359_Cyanobacterium_Yellowstone_A-prime_CYA_0084_rpe
359_Acaryochloris_marina_MBIC11017_AM1_1399_rpe
359_Synechococcus_sp._PCC_6301_syc0920_c_rpe
359_Synechococcus_sp._PCC_7942_Synpcc7942_0604
359_Prochlorococcus_marinus_MIT9303_P9303_16821_rpe
359_Prochlorococcus_marinus_MIT9313_PMT0569_rpe_cbbE
359_Prochlorococcus_marinus_MIT9211_P9211_09961_rpe
359_Synechococcus_sp._9605_Syncc9605_1252
359_Synechococcus_sp._WH8102_SYNW1115_rpe
359_Synechococcus_sp._CC9311_sync_1683_rpe-1
359_Synechocossus_sp._WH7803_SynWH7803_1301
285_Paulinella_sp._DL-2016a_CHR_PCKR_260_cfxE
285_Paulinella_chromatophora_CHR_PCC_0267_cfxE
359_Synechococcus_sp._9902_Syncc9902_1230
359_Synechococcus_sp._RCC307_SynRCC307_1256
359_Synechococcus_sp.WH5701_ZP01085631
Paulinella
359_Thermosynechococcus_elongatus_tll2369_rpe
359_Arthrospira__platensis_NIES_39_NIES39_O06990_rpe
359_Arthrospira_maxima_CS-328_001650
359_Cyanothece_PCC7425_Cyan7425_1996
359_Synechocystis_sp._PCC_6803_sll0807_cfxE
359_Anabaena_variabilis_ATCC_29413_Ava_4675
359_Anabaena_sp._PCC_7120_alr0782
359_Nostoc_punctiforme_sp._PCC73102_Npun_F1541
359_Trichodesmium_erythraeum__405_1_Tery_2606
359_Cyanothece_PCC8802_Cyan8802_1646
359_Cyanothece_PCC8801_PCC8801_1619
359_Cyanothece_PCC7424_PCC7424_5270
359_Cyanothece_ATCC51142_cce_0798_rpe
359_Crocosphaera_watsonii_WH8501_g4803
359_Microcystis_aeruginosa_NIES843_MAE_61690_rpe
359_Synechococcus_sp._PCC7002_SYNPCC7002_A0324_rpe
359_Cyanobacterium_UCYN_A_UCYN_01930
359_Gloeobacter_violaceus_gll3548
Cyanobacteria
359_Caulobacter_crescentus_CB15_CC_0101
359_Erythrobacter_litoralis_HTCC2594_ELI_12550
359_Rhodospirillum_centenum_SW_RC1_2350_rpe
359_Rhodobacter_sphaeroides_KD131_RSKD131_1094
359_Rhodobacter_sphaeroides_ATCC_17029_Rsph17029_1430
359_Rhodobacter_sphaeroides_2_4_1_RSP_2864_cbbE
359_Rhodobacter_sphaeroides_ATCC_17025_Rsph17025_1512
359_Ruegeria__pomeroyi_DSS-3_SPO0779_rpe
359_Bradyrhizobium_sp._BTAi1_BBta_0454_rpe
359_Gluconobacter_oxydans_621H_GOX1352
359_Bradyrhizobium_japonicum_USDA_110_blr2588
359_Azorhizobium_caulinodans_ORS_571_AZC_1810
359_Methylobacterium_extorquens_AM1_MexAM1_META1p0768_rpe
359_Methylobacterium_nodulans_ORS_2060_Mnod_1739
359_Bradyrhizobium_japonicum_USDA_110_blr5680_rpe
359_Bradyrhizobium_sp._BTAi1_BBta_5332_rpe
359_Bradyrhizobium_sp._ORS_278_BRADO2842_rpe
359_Rhodopseudomonas_palustris_BisB18_RPC_1614
359_Rhodopseudomonas_palustris_HaA2_RPB_3689
359_Rhodopseudomonas_palustris_BisA53_RPE_1642
359_Rhodopseudomonas_palustris_BisB5_RPD_1771
285_Rhodopsudomonas_palustris_CGA009_RPA3815_cbbE
359_Rhodopseudomonas_palustris_CGA009_RPA3815_cbbE
359_Rhodopseudomonas_palustris_TIE1_Rpal_4340
359_Sinorhizobium_medicae_WSM_419_Smed_3927
359_Sinorhizobium_meliloti_1021_SM_b20195_ppe
359_Mesorhizobium_loti_MAFF303099_mlr4760
359_Mesorhizobium_sp._BNC1_Meso_0005
359_Rhizobium_sp._NGR234_NGR_c15200_rpe
359_Sinorhizobium_medicae_WSM_419_Smed_1489
359_Sinorhizobium_meliloti_1021_SMc00511_rpe
359_Agrobacterium_tumefaciens_str._C58_Cereon_Atu1839_rpe
359_Rhizobium_etli_CIAT_652_RHECIAT_CH0002380_rpe
359_Rhizobium_etli_CFN_42_RHE_CH02270_rpe
359_Rhizobium_leguminosarum_bv_viciae_3841_RL2598_rpe
359_Rhizobium_leguminosarum_bv_trifolii_WSM1325_Rleg_2125
359_Rhizobium_leguminosarum_bv_trifolii_WSM2304_Rleg2_1918
359_Brucella_suis_1330_BR0850_rpe
Alpha
proteobacteria
285_Pseudo-nitzschia_multiseries_CLN-47_301313
359_Prochlorococcus_marinus_NATL1A_NATL1_08041_rpe
359_Prochlorococcus_marinus_NATL2A_PMN2A_0172
359_Prochlorococcus_marinus_CCMP1375_Pro0839_rpe
359_Synechococcus_sp._CC9311_sync_1684_rpe-2
359_Prochlorococcus_marinus_AS9601_A9601_08281_rpe
359_Prochlorococcus_marinus_MIT9215_P9215_08601_rpe
359_Prochlorococcus_marinus_MIT9301_P9301_08261_rpe
359_Prochlorococcus_marinus_CCMP_1986_PMM0766_rpe
359_Prochlorococcus_marinus_MIT9515_P9515_08171_rpe
359_Prochlorococcus_marinus_MIT9312_PMT9312_0774
Cyanobacteria
(Prochlorococcus)
359_Francisella_tularensis_tularensis_FTT0789_rpe
359_Buchnera_aphidicola_str._APS_BU537_rpe
359_Xylella_fastidiosa_9a5c_XF0208
359_Vibrio_cholerae_O1_biovar_eltor_str._N16961_VC2625
Gamma
proteobacteria
285_Thalassiosira_pseudonana_ACI64467
285_Thalassiosira_pseudonana_EED94487
285_Phaeodactylum_tricornutum_J53935
285_Pseudo-nitzschia_multiseries_CLN-47_66186
Diatoms
359_Pseudomonas_syringae_pv._tomato_str._DC3000_PSPTO_0566_rpe
359_Escherichia_coli_K-12_MG1655_b3386_rpe
359_Yersinia_pestis_YPO0155_rpe
359_Bordetella_pertussis_Tohama_I_BP3266_rpe
359_Aromatoleum_aromaticum__EbN1_ebA4173_rpe
359_Cupriavidus_taiwanensis_RALTA_A2769_rpe
359_Ralstonia_solanacearum_GMI1000_RSc2879_rpe
359_Burkholderia_mallei_ATCC_23344_BMAA0535_rpe
359_Burkholderia_phymatum_STM815_Bphy_2696
359_Chromobacterium_violaceum_ATCC_12472_CV_2182_rpe
359_Rhodospirillum_centenum_SW_RC1_4063_cbbE
359_Cupriavidus_taiwanensis_RALTA_B1697_cbbE
359_Rhodospirillum_rubrum_ATCC_11170_Rru_A2402
Beta
proteobacteria
0.98/0.99
1/0.63
0.79/1
0.86/0.78
1/1
0.69/0.80
1/0.99
0.88/0.67
0.88/0.99
0.93/1
0.96/0.97
0.93/1
0.67/0.68
0.4

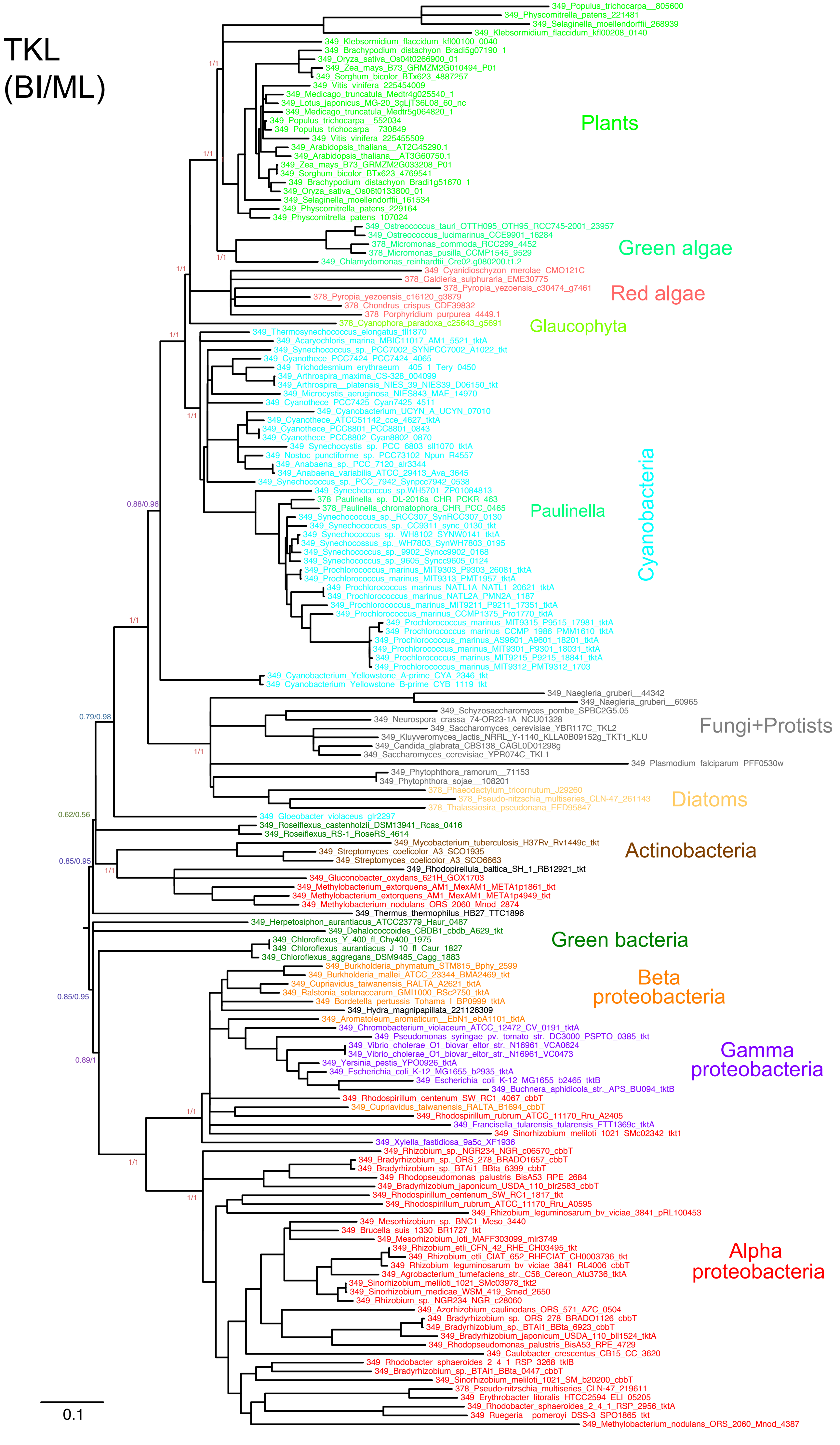

TKL (BI/ML)
349_Populus_trichocarpa__805600
349_Physcomitrella_patens_221481
349_Selaginella_moellendorffii_268939
349_Klebsormidium_flaccidum_kfl00208_0140
349_Klebsormidium_flaccidum_kfl00100_0040
349_Brachypodium_distachyon_Bradi5g07190_1
349_Oryza_sativa_Os04t0266900_01
349_Zea_mays_B73_GRMZM2G010494_P01
349_Sorghum_bicolor_BTx623_4887257
349_Vitis_vinifera_225454009
349_Medicago_truncatula_Medtr4g025540_1
349_Lotus_japonicus_MG-20_3gLjT36L08_60_nc
349_Medicago_truncatula_Medtr5g064820_1
349_Populus_trichocarpa__552034
349_Populus_trichocarpa__730849
349_Vitis_vinifera_225455509
349_Arabidopsis_thaliana__AT2G45290.1
349_Arabidopsis_thaliana__AT3G60750.1
349_Zea_mays_B73_GRMZM2G033208_P01
349_Sorghum_bicolor_BTx623_4769541
349_Brachypodium_distachyon_Bradi1g51670_1
349_Oryza_sativa_Os06t0133800_01
349_Selaginella_moellendorffii_161534
349_Physcomitrella_patens_229164
349_Physcomitrella_patens_107024
Plants
349_Ostreococcus_tauri_OTTH095_OTH95_RCC745-2001_23957
349_Ostreococcus_lucimarinus_CCE9901_16284
378_Micromonas_commoda_RCC299_4452
378_Micromonas_pusilla_CCMP1545_9529
349_Chlamydomonas_reinhardtii_Cre02.g080200.t1.2
Green algae
349_Cyanidioschyzon_merolae_CMO121C
378_Galdieria_sulphuraria_EME30775
378_Pyropia_yezoensis_c30474_g7461
378_Pyropia_yezoensis_c16120_g3879
378_Chondrus_crispus_CDF39832
378_Porphyridium_purpurea_4449.1
Red algae
378_Cyanophora_paradoxa_c25643_g5691
Glaucophyta
349_Thermosynechococcus_elongatus_tll1870
349_Acaryochloris_marina_MBIC11017_AM1_5521_tktA
349_Synechococcus_sp._PCC7002_SYNPCC7002_A1022_tkt
349_Cyanothece_PCC7424_PCC7424_4065
349_Trichodesmium_erythraeum__405_1_Tery_0450
349_Arthrospira_maxima_CS-328_004099
349_Arthrospira__platensis_NIES_39_NIES39_D06150_tkt
349_Microcystis_aeruginosa_NIES843_MAE_14970
349_Cyanothece_PCC7425_Cyan7425_4511
349_Cyanobacterium_UCYN_A_UCYN_07010
349_Cyanothece_ATCC51142_cce_4627_tktA
349_Cyanothece_PCC8801_PCC8801_0843
349_Cyanothece_PCC8802_Cyan8802_0870
349_Synechocystis_sp._PCC_6803_sll1070_tktA
349_Nostoc_punctiforme_sp._PCC73102_Npun_R4557
349_Anabaena_sp._PCC_7120_alr3344
349_Anabaena_variabilis_ATCC_29413_Ava_3645
349_Synechococcus_sp._PCC_7942_Synpcc7942_0538
349_Synechococcus_sp.WH5701_ZP01084813
378_Paulinella_sp._DL-2016a_CHR_PCKR_463
378_Paulinella_chromatophora_CHR_PCC_0465
Paulinella
349_Synechococcus_sp._RCC307_SynRCC307_0130
349_Synechococcus_sp._CC9311_sync_0130_tkt
349_Synechococcus_sp._WH8102_SYNW0141_tktA
349_Synechocossus_sp._WH7803_SynWH7803_0195
349_Synechococcus_sp._9902_Syncc9902_0168
349_Synechococcus_sp._9605_Syncc9605_0124
349_Prochlorococcus_marinus_MIT9303_P9303_26081_tktA
349_Prochlorococcus_marinus_MIT9313_PMT1957_tktA
349_Prochlorococcus_marinus_NATL1A_NATL1_20621_tktA
349_Prochlorococcus_marinus_NATL2A_PMN2A_1187
349_Prochlorococcus_marinus_MIT9211_P9211_17351_tktA
349_Prochlorococcus_marinus_CCMP1375_Pro1770_tktA
349_Prochlorococcus_marinus_MIT9315_P9515_17981_tktA
349_Prochlorococcus_marinus_CCMP_1986_PMM1610_tktA
349_Prochlorococcus_marinus_AS9601_A9601_18201_tktA
349_Prochlorococcus_marinus_MIT9301_P9301_18031_tktA
349_Prochlorococcus_marinus_MIT9215_P9215_18841_tktA
349_Prochlorococcus_marinus_MIT9312_PMT9312_1703
349_Cyanobacterium_Yellowstone_A-prime_CYA_2346_tkt
349_Cyanobacterium_Yellowstone_B-prime_CYB_1119_tkt
Cyanobacteria
349_Naegleria_gruberi__44342
349_Naegleria_gruberi__60965
349_Schyzosaccharomyces_pombe_SPBC2G5.05
349_Neurospora_crassa_74-OR23-1A_NCU01328
349_Saccharomyces_cerevisiae_YBR117C_TKL2
349_Kluyveromyces_lactis_NRRL_Y-1140_KLLA0B09152g_TKT1_KLU
349_Candida_glabrata_CBS138_CAGL0D01298g
349_Saccharomyces_cerevisiae_YPR074C_TKL1
Fungi+Protists
349_Plasmodium_falciparum_PFF0530w
349_Phytophthora_ramorum__71153
349_Phytophthora_sojae__108201
378_Phaeodactylum_tricornutum_J29260
378_Pseudo-nitzschia_multiseries_CLN-47_261143
378_Thalassiosira_pseudonana_EED95847
Diatoms
349_Gloeobacter_violaceus_glr2297
349_Roseiflexus_castenholzii_DSM13941_Rcas_0416
349_Roseiflexus_RS-1_RoseRS_4614
349_Mycobacterium_tuberculosis_H37Rv_Rv1449c_tkt
349_Streptomyces_coelicolor_A3_SCO1935
349_Streptomyces_coelicolor_A3_SCO6663
Actinobacteria
349_Rhodopirellula_baltica_SH_1_RB12921_tkt
349_Gluconobacter_oxydans_621H_GOX1703
349_Methylobacterium_extorquens_AM1_MexAM1_META1p1861_tkt
349_Methylobacterium_extorquens_AM1_MexAM1_META1p4949_tkt
349_Methylobacterium_nodulans_ORS_2060_Mnod_2874
349_Thermus_thermophilus_HB27_TTC1896
349_Herpetosiphon_aurantiacus_ATCC23779_Haur_0487
349_Dehalococcoides_CBDB1_cbdb_A629_tkt
349_Chloroflexus_Y_400_fl_Chy400_1975
349_Chloroflexus_aurantiacus_J_10_fl_Caur_1827
349_Chloroflexus_aggregans_DSM9485_Cagg_1883
Green bacteria
349_Burkholderia_phymatum_STM815_Bphy_2599
349_Burkholderia_mallei_ATCC_23344_BMA2469_tkt
349_Cupriavidus_taiwanensis_RALTA_A2621_tktA
349_Ralstonia_solanacearum_GMI1000_RSc2750_tktA
349_Bordetella_pertussis_Tohama_I_BP0999_tktA
Beta proteobacteria
349_Hydra_magnipapillata_221126309
349_Aromatoleum_aromaticum__EbN1_ebA1101_tktA
349_Chromobacterium_violaceum_ATCC_12472_CV_0191_tktA
349_Pseudomonas_syringae_pv._tomato_str._DC3000_PSPTO_0385_tkt
349_Vibrio_cholerae_O1_biovar_eltor_str._N16961_VCA0624
349_Vibrio_cholerae_O1_biovar_eltor_str._N16961_VC0473
349_Yersinia_pestis_YPO0926_tktA
349_Escherichia_coli_K-12_MG1655_b2935_tktA
349_Escherichia_coli_K-12_MG1655_b2465_tktB
349_Buchnera_aphidicola_str._APS_BU094_tktB
Gamma proteobacteria
349_Rhodospirillum_centenum_SW_RC1_4067_cbbT
349_Cupriavidus_taiwanensis_RALTA_B1694_cbbT
349_Rhodospirillum_rubrum_ATCC_11170_Rru_A2405
349_Francisella_tularensis_tularensis_FTT1369c_tktA
349_Sinorhizobium_meliloti_1021_SMc02342_tkt1
349_Xylella_fastidiosa_9a5c_XF1936
349_Rhizobium_sp._NGR234_NGR_c06570_cbbT
349_Bradyrhizobium_sp._ORS_278_BRADO1657_cbbT
349_Bradyrhizobium_sp._BTAi1_BBta_6399_cbbT
349_Rhodopseudomonas_palustris_BisA53_RPE_2684
349_Bradyrhizobium_japonicum_USDA_110_blr2583_cbbT
349_Rhodospirillum_centenum_SW_RC1_1817_tkt
349_Rhodospirillum_rubrum_ATCC_11170_Rru_A0595
349_Rhizobium_leguminosarum_bv_viciae_3841_pRL100453
349_Mesorhizobium_sp._BNC1_Meso_3440
349_Brucella_suis_1330_BR1727_tkt
349_Mesorhizobium_loti_MAFF303099_mlr3749
349_Rhizobium_etli_CFN_42_RHE_CH03495_tkt
349_Rhizobium_etli_CIAT_652_RHECIAT_CH0003736_tkt
349_Rhizobium_leguminosarum_bv_viciae_3841_RL4006_cbbT
349_Agrobacterium_tumefaciens_str._C58_Cereon_Atu3736_tktA
349_Sinorhizobium_meliloti_1021_SMc03978_tkt2
349_Sinorhizobium_medicae_WSM_419_Smed_2650
349_Rhizobium_sp._NGR234_NGR_c28060
349_Azorhizobium_caulinodans_ORS_571_AZC_0504
349_Bradyrhizobium_sp._ORS_278_BRADO1126_cbbT
349_Bradyrhizobium_sp._BTAi1_BBta_6923_cbbT
349_Bradyrhizobium_japonicum_USDA_110_bll1524_tktA
349_Rhodopseudomonas_palustris_BisA53_RPE_4729
349_Caulobacter_crescentus_CB15_CC_3620
349_Rhodobacter_sphaeroides_2_4_1_RSP_3268_tklB
349_Bradyrhizobium_sp._BTAi1_BBta_0447_cbbT
349_Sinorhizobium_meliloti_1021_SM_b20200_cbbT
378_Pseudo-nitzschia_multiseries_CLN-47_219611
349_Erythrobacter_litoralis_HTCC2594_ELI_05205
349_Rhodobacter_sphaeroides_2_4_1_RSP_2956_tktA
349_Ruegeria__pomeroyi_DSS-3_SPO1865_tkt
349_Methylobacterium_nodulans_ORS_2060_Mnod_4387
Alpha proteobacteria
1/1
0.88/0.96
0.79/0.98
0.62/0.56
0.85/0.95
0.89/1
0.1
