# Supplementary material for: Complex origins of chloroplast membranes with photosynthetic machineries: multiple transfers of genes from divergent organisms at different times or a single endosymbiotic event?
Source: J Plant Res. 2019 Dec 6;133(1):15–33. doi: 10.1007/s10265-019-01157-z (PMC6946739; doi:10.1007/s10265-019-01157-z)
Supplement: Supplementary file 7 — Supplementary material 7 (PDF 324 kb) [file 10265_2019_1157_MOESM7_ESM.pdf]

Journal of Plant Research

Online Resources

Complex origins of chloroplast membranes with photosynthetic machineries: Multiple transfers of genes from divergent organisms at different times or a single endosymbiotic event?

Naoki Sato
University of Tokyo
naokisat@bio.c.u-tokyo.ac.jp

Online Resource 7. Phylogenetic trees of proteins involved in other functions.

This PDF includes phylogenetic trees of the proteins involved in chloroplast division, FtsZ, MinD, and MinE, and a translocon component Tic20/Ycf60.

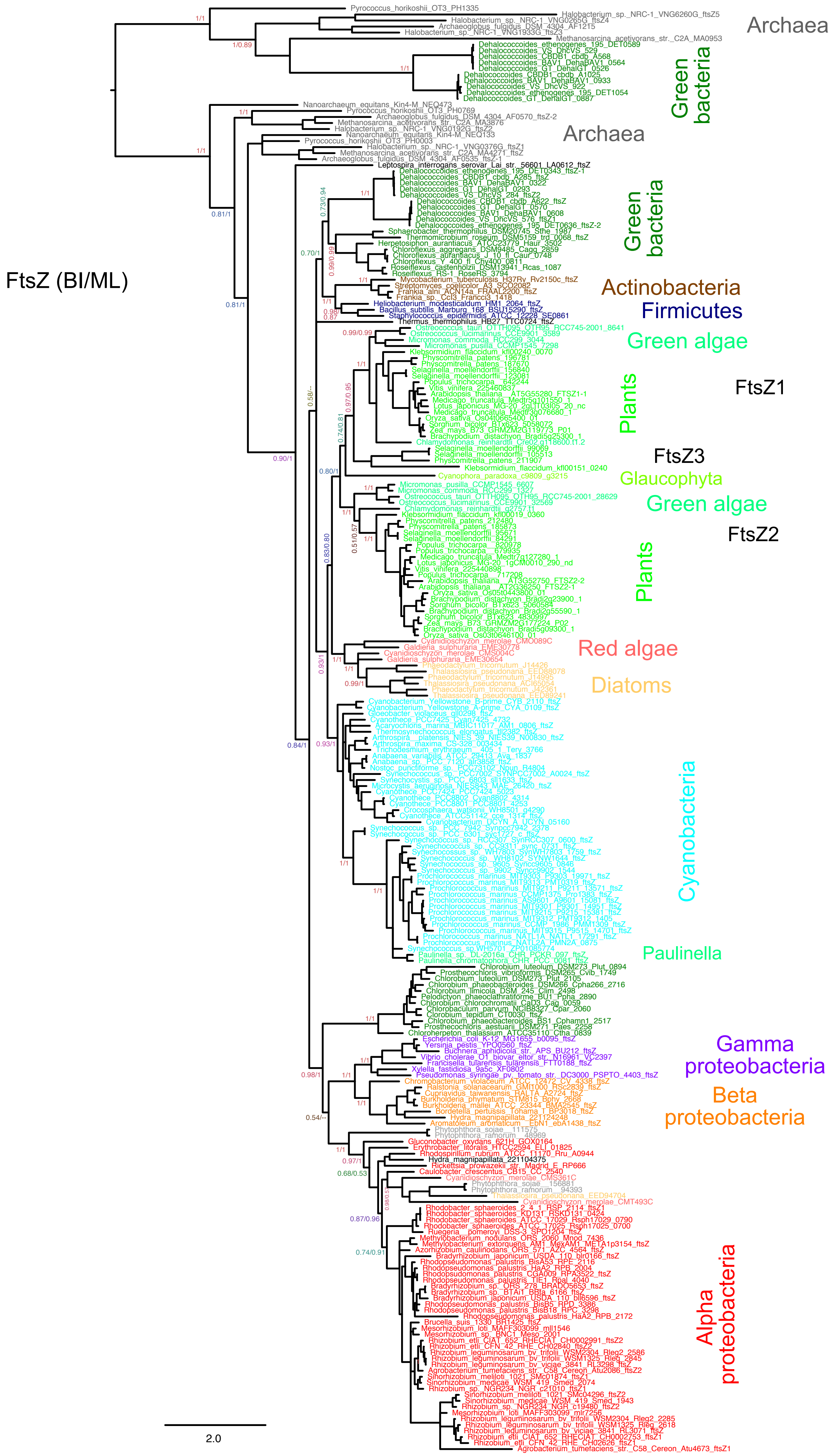
FtsZ (BI/ML)
Archaea
Green bacteria
Archaea
Green bacteria
Actinobacteria
Firmicutes
Green algae
Plants
FtsZ1
FtsZ3
Glaucophyta
Green algae
FtsZ2
Plants
Red algae
Diatoms
Cyanobacteria
Paulinella
Gamma proteobacteria
Beta proteobacteria
Alpha proteobacteria
2.0

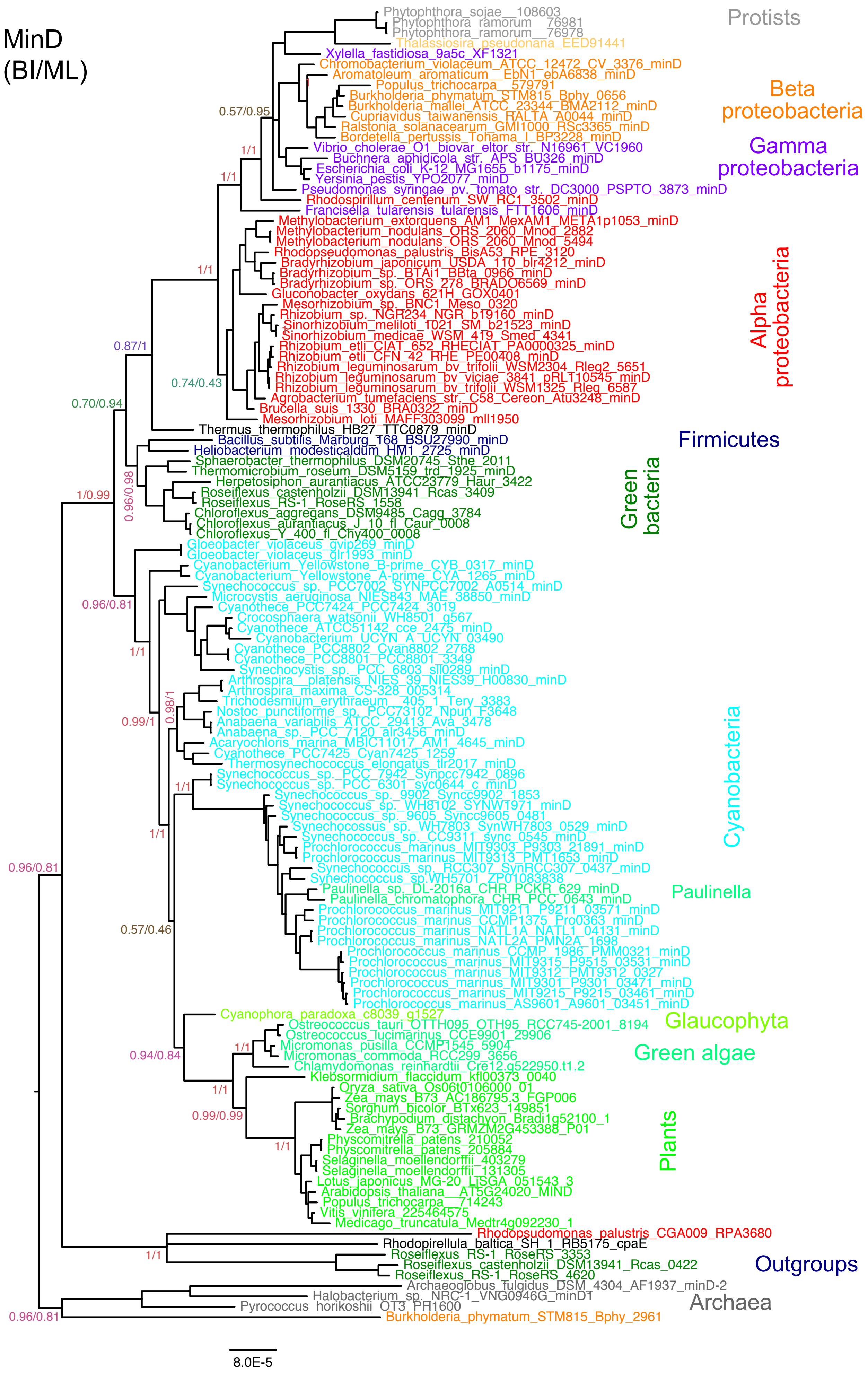
MinD
(BI/ML)
Phytophthora_sojae__108603
Phytophthora_ramorum__76981
Phytophthora_ramorum__76978
Thalassiosira_pseudonana_EED91441
Protists
Xylella_fastidiosa_9a5c_XF1321
Chromobacterium_violaceum_ATCC_12472_CV_3376_minD
Aromatoleum_aromaticum__EbN1_ebA6838_minD
Populus_trichocarpa__579791
Burkholderia_phymatum_STM815_Bphy_0656
Burkholderia_mallei_ATCC_23344_BMA2112_minD
Cupriavidus_taiwanensis_RALTA_A0044_minD
Ralstonia_solanacearum_GMI1000_RSc3365_minD
Bordetella_pertussis_Tohama_I_BP3228_minD
Beta
proteobacteria
0.57/0.95
Vibrio_cholerae_O1_biovar_eltor_str._N16961_VC1960
Buchnera_aphidicola_str._APS_BU326_minD
Escherichia_coli_K-12_MG1655_b1175_minD
Yersinia_pestis_YPO2077_minD
Pseudomonas_syringae_pv._tomato_str._DC3000_PSPTO_3873_minD
Gamma
proteobacteria
1/1
1/1
Rhodospirillum_centenum_SW_RC1_3502_minD
Francisella_tularensis_tularensis_FTT1606_minD
Methylobacterium_extorquens_AM1_MexAM1_META1p1053_minD
Methylobacterium_nodulans_ORS_2060_Mnod_2882
Methylobacterium_nodulans_ORS_2060_Mnod_5494
Rhodopseudomonas_palustris_BisA53_RPE_3120
Bradyrhizobium_japonicum_USDA_110_blr4212_minD
Bradyrhizobium_sp._BTAi1_BBta_0966_minD
Bradyrhizobium_sp._ORS_278_BRADO6569_minD
Gluconobacter_oxydans_621H_GOX0401
Mesorhizobium_sp._BNC1_Meso_0320
Rhizobium_sp._NGR234_NGR_b19160_minD
Sinorhizobium_meliloti_1021_SM_b21523_minD
Sinorhizobium_medicae_WSM_419_Smed_4341
Rhizobium_etli_CIAT_652_RHECIAT_PA0000325_minD
Rhizobium_etli_CFN_42_RHE_PE00408_minD
Rhizobium_leguminosarum_bv_trifolii_WSM2304_Rleg2_5651
Rhizobium_leguminosarum_bv_viciae_3841_pRL110545_minD
Rhizobium_leguminosarum_bv_trifolii_WSM1325_Rleg_6587
Agrobacterium_tumefaciens_str._C58_Cereon_Atu3248_minD
Brucella_suis_1330_BRA0322_minD
Mesorhizobium_loti_MAFF303099_mll1950
Alpha
proteobacteria
1/1
0.87/1
0.74/0.43
0.70/0.94
Thermus_thermophilus_HB27_TTC0879_minD
Bacillus_subtilis_Marburg_168_BSU27990_minD
Heliobacterium_modesticaldum_HM1_2725_minD
Firmicutes
Sphaerobacter_thermophilus_DSM20745_Sthe_2011
Thermomicrobium_roseum_DSM5159_trd_1925_minD
Herpetosiphon_aurantiacus_ATCC23779_Haur_3422
Roseiflexus_castenholzii_DSM13941_Rcas_3409
Roseiflexus_RS-1_RoseRS_1558
Chloroflexus_aggregans_DSM9485_Cagg_3784
Chloroflexus_aurantiacus_J_10_fl_Caur_0008
Chloroflexus_Y_400_fl_Chy400_0008
Green
bacteria
1/0.99
0.96/0.98
Gloeobacter_violaceus_gvip269_minD
Gloeobacter_violaceus_glr1993_minD
Cyanobacterium_Yellowstone_B-prime_CYB_0317_minD
Cyanobacterium_Yellowstone_A-prime_CYA_1265_minD
Synechococcus_sp._PCC7002_SYNPCC7002_A0514_minD
Microcystis_aeruginosa_NIES843_MAE_38850_minD
Cyanothece_PCC7424_PCC7424_3019
Crocosphaera_watsonii_WH8501_g567
Cyanothece_ATCC51142_cce_2475_minD
Cyanobacterium_UCYN_A_UCYN_03490
Cyanothece_PCC8802_Cyan8802_2768
Cyanothece_PCC8801_PCC8801_3349
Synechocystis_sp._PCC_6803_sll0289_minD
0.96/0.81
1/1
Arthrospira__platensis_NIES_39_NIES39_H00830_minD
Arthrospira_maxima_CS-328_005314
Trichodesmium_erythraeum__405_1_Tery_3383
Nostoc_punctiforme_sp._PCC73102_Npun_F3648
Anabaena_variabilis_ATCC_29413_Ava_3478
Anabaena_sp._PCC_7120_alr3456_minD
Acaryochloris_marina_MBIC11017_AM1_4645_minD
Cyanothece_PCC7425_Cyan7425_1259
Thermosynechococcus_elongatus_tlr2017_minD
0.99/1
0.98/1
Synechococcus_sp._PCC_7942_Synpcc7942_0896
Synechococcus_sp._PCC_6301_syc0644_c_minD
Synechococcus_sp._9902_Syncc9902_1853
Synechococcus_sp._WH8102_SYNW1971_minD
Synechococcus_sp._9605_Syncc9605_0481
Synechocossus_sp._WH7803_SynWH7803_0529_minD
Synechococcus_sp._CC9311_sync_0545_minD
Prochlorococcus_marinus_MIT9303_P9303_21891_minD
Prochlorococcus_marinus_MIT9313_PMT1653_minD
Synechococcus_sp._RCC307_SynRCC307_0437_minD
Synechococcus_sp.WH5701_ZP01083838
1/1
1/1
Cyanobacteria
Paulinella_sp._DL-2016a_CHR_PCKR_629_minD
Paulinella_chromatophora_CHR_PCC_0643_minD
Paulinella
Prochlorococcus_marinus_MIT9211_P9211_03571_minD
Prochlorococcus_marinus_CCMP1375_Pro0363_minD
Prochlorococcus_marinus_NATL1A_NATL1_04131_minD
Prochlorococcus_marinus_NATL2A_PMN2A_1698
Prochlorococcus_marinus_CCMP_1986_PMM0321_minD
Prochlorococcus_marinus_MIT9315_P9515_03531_minD
Prochlorococcus_marinus_MIT9312_PMT9312_0327
Prochlorococcus_marinus_MIT9301_P9301_03471_minD
Prochlorococcus_marinus_MIT9215_P9215_03461_minD
Prochlorococcus_marinus_AS9601_A9601_03451_minD
0.96/0.81
0.57/0.46
Cyanophora_paradoxa_c8039_g1527
Glaucophyta
Ostreococcus_tauri_OTTH095_OTH95_RCC745-2001_8194
Ostreococcus_lucimarinus_CCE9901_29906
Micromonas_pusilla_CCMP1545_5904
Micromonas_commoda_RCC299_3656
Chlamydomonas_reinhardtii_Cre12.g522950.t1.2
Green algae
1/1
0.94/0.84
Klebsormidium_flaccidum_kfl00373_0040
Oryza_sativa_Os06t0106000_01
Zea_mays_B73_AC186795.3_FGP006
Sorghum_bicolor_BTx623_149851
Brachypodium_distachyon_Bradi1g52100_1
Zea_mays_B73_GRMZM2G453388_P01
1/1
0.99/0.99
Physcomitrella_patens_210052
Physcomitrella_patens_205884
Selaginella_moellendorffii_403279
Selaginella_moellendorffii_131305
Lotus_japonicus_MG-20_LjSGA_051543_3
Arabidopsis_thaliana__AT5G24020_MIND
Populus_trichocarpa__714243
Vitis_vinifera_225464575
Medicago_truncatula_Medtr4g092230_1
1/1
Plants
Rhodopsudomonas_palustris_CGA009_RPA3680
Rhodopirellula_baltica_SH_1_RB5175_cpaE
Roseiflexus_RS-1_RoseRS_3353
Roseiflexus_castenholzii_DSM13941_Rcas_0422
Roseiflexus_RS-1_RoseRS_4620
1/1
Outgroups
Archaeoglobus_fulgidus_DSM_4304_AF1937_minD-2
Halobacterium_sp._NRC-1_VNG0946G_minD1
Pyrococcus_horikoshii_OT3_PH1600
Archaea
Burkholderia_phymatum_STM815_Bphy_2961
0.96/0.81
8.0E-5

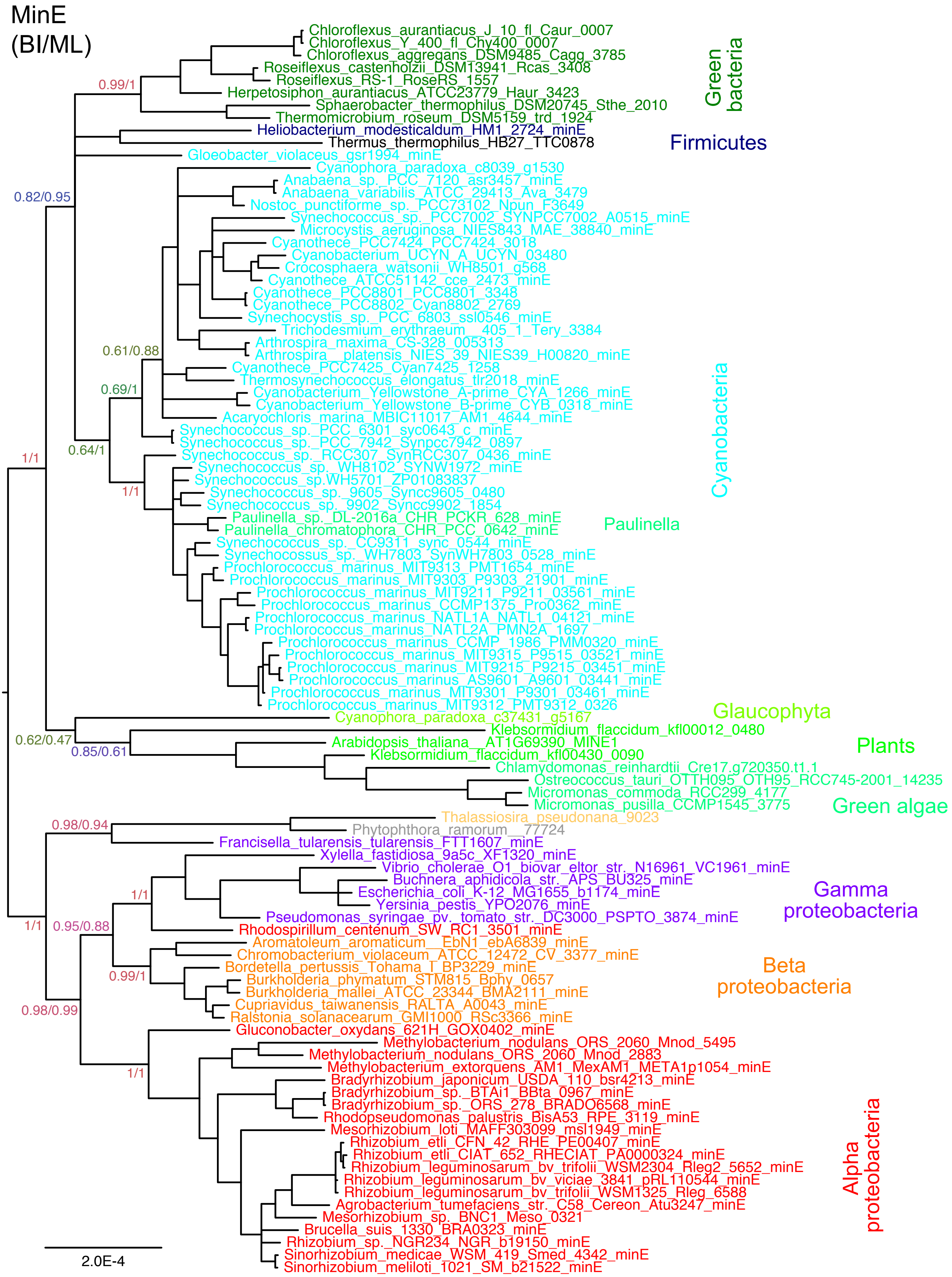
MinE
(BI/ML)
Chloroflexus_aurantiacus_J_10_fl_Caur_0007
Chloroflexus_Y_400_fl_Chy400_0007
Chloroflexus_aggregans_DSM9485_Cagg_3785
Roseiflexus_castenholzii_DSM13941_Rcas_3408
Roseiflexus_RS-1_RoseRS_1557
Herpetosiphon_aurantiacus_ATCC23779_Haur_3423
Sphaerobacter_thermophilus_DSM20745_Sthe_2010
Thermomicrobium_roseum_DSM5159_trd_1924
Green bacteria
0.99/1
Heliobacterium_modesticaldum_HM1_2724_minE
Thermus_thermophilus_HB27_TTC0878
Firmicutes
Gloeobacter_violaceus_gsr1994_minE
Cyanophora_paradoxa_c8039_g1530
Anabaena_sp._PCC_7120_asr3457_minE
Anabaena_variabilis_ATCC_29413_Ava_3479
Nostoc_punctiforme_sp._PCC73102_Npun_F3649
0.82/0.95
Synechococcus_sp._PCC7002_SYNPCC7002_A0515_minE
Microcystis_aeruginosa_NIES843_MAE_38840_minE
Cyanothece_PCC7424_PCC7424_3018
Cyanobacterium_UCYN_A_UCYN_03480
Crocosphaera_watsonii_WH8501_g568
Cyanothece_ATCC51142_cce_2473_minE
Cyanothece_PCC8801_PCC8801_3348
Cyanothece_PCC8802_Cyan8802_2769
Synechocystis_sp._PCC_6803_ssl0546_minE
Trichodesmium_erythraeum__405_1_Tery_3384
Arthrospira_maxima_CS-328_005313
Arthrospira__platensis_NIES_39_NIES39_H00820_minE
0.61/0.88
Cyanothece_PCC7425_Cyan7425_1258
Thermosynechococcus_elongatus_tlr2018_minE
0.69/1
Cyanobacterium_Yellowstone_A-prime_CYA_1266_minE
Cyanobacterium_Yellowstone_B-prime_CYB_0318_minE
Acaryochloris_marina_MBIC11017_AM1_4644_minE
Synechococcus_sp._PCC_6301_syc0643_c_minE
Synechococcus_sp._PCC_7942_Synpcc7942_0897
0.64/1
Synechococcus_sp._RCC307_SynRCC307_0436_minE
1/1
Synechococcus_sp._WH8102_SYNW1972_minE
Synechococcus_sp.WH5701_ZP01083837
1/1
Synechococcus_sp._9605_Syncc9605_0480
Synechococcus_sp._9902_Syncc9902_1854
Cyanobacteria
Paulinella_sp._DL-2016a_CHR_PCKR_628_minE
Paulinella_chromatophora_CHR_PCC_0642_minE
Paulinella
Synechococcus_sp._CC9311_sync_0544_minE
Synechocossus_sp._WH7803_SynWH7803_0528_minE
Prochlorococcus_marinus_MIT9313_PMT1654_minE
Prochlorococcus_marinus_MIT9303_P9303_21901_minE
Prochlorococcus_marinus_MIT9211_P9211_03561_minE
Prochlorococcus_marinus_CCMP1375_Pro0362_minE
Prochlorococcus_marinus_NATL1A_NATL1_04121_minE
Prochlorococcus_marinus_NATL2A_PMN2A_1697
Prochlorococcus_marinus_CCMP_1986_PMM0320_minE
Prochlorococcus_marinus_MIT9315_P9515_03521_minE
Prochlorococcus_marinus_MIT9215_P9215_03451_minE
Prochlorococcus_marinus_AS9601_A9601_03441_minE
Prochlorococcus_marinus_MIT9301_P9301_03461_minE
Prochlorococcus_marinus_MIT9312_PMT9312_0326
Cyanophora_paradoxa_c37431_g5167
Glaucophyta
Klebsormidium_flaccidum_kfl00012_0480
0.62/0.47
Arabidopsis_thaliana__AT1G69390_MINE1
0.85/0.61
Klebsormidium_flaccidum_kfl00430_0090
Plants
Chlamydomonas_reinhardtii_Cre17.g720350.t1.1
Ostreococcus_tauri_OTTH095_OTH95_RCC745-2001_14235
Micromonas_commoda_RCC299_4177
Micromonas_pusilla_CCMP1545_3775
Green algae
Thalassiosira_pseudonana_9023
0.98/0.94
Phytophthora_ramorum__77724
Francisella_tularensis_tularensis_FTT1607_minE
Xylella_fastidiosa_9a5c_XF1320_minE
Vibrio_cholerae_O1_biovar_eltor_str._N16961_VC1961_minE
Buchnera_aphidicola_str._APS_BU325_minE
1/1
Escherichia_coli_K-12_MG1655_b1174_minE
Yersinia_pestis_YPO2076_minE
Gamma proteobacteria
Pseudomonas_syringae_pv._tomato_str._DC3000_PSPTO_3874_minE
1/1
0.95/0.88
Rhodospirillum_centenum_SW_RC1_3501_minE
Aromatoleum_aromaticum__EbN1_ebA6839_minE
Chromobacterium_violaceum_ATCC_12472_CV_3377_minE
Bordetella_pertussis_Tohama_I_BP3229_minE
0.99/1
Burkholderia_phymatum_STM815_Bphy_0657
Burkholderia_mallei_ATCC_23344_BMA2111_minE
Beta proteobacteria
Cupriavidus_taiwanensis_RALTA_A0043_minE
0.98/0.99
Ralstonia_solanacearum_GMI1000_RSc3366_minE
Gluconobacter_oxydans_621H_GOX0402_minE
Methylobacterium_nodulans_ORS_2060_Mnod_5495
Methylobacterium_nodulans_ORS_2060_Mnod_2883
Methylobacterium_extorquens_AM1_MexAM1_META1p1054_minE
1/1
Bradyrhizobium_japonicum_USDA_110_bsr4213_minE
Bradyrhizobium_sp._BTAi1_BBta_0967_minE
Bradyrhizobium_sp._ORS_278_BRADO6568_minE
Rhodopseudomonas_palustris_BisA53_RPE_3119_minE
Mesorhizobium_loti_MAFF303099_msl1949_minE
Rhizobium_etli_CFN_42_RHE_PE00407_minE
Rhizobium_etli_CIAT_652_RHECIAT_PA0000324_minE
Rhizobium_leguminosarum_bv_trifolii_WSM2304_Rleg2_5652_minE
Rhizobium_leguminosarum_bv_viciae_3841_pRL110544_minE
Rhizobium_leguminosarum_bv_trifolii_WSM1325_Rleg_6588
Agrobacterium_tumefaciens_str._C58_Cereon_Atu3247_minE
Mesorhizobium_sp._BNC1_Meso_0321
Alpha proteobacteria
Brucella_suis_1330_BRA0323_minE
Rhizobium_sp._NGR234_NGR_b19150_minE
Sinorhizobium_medicae_WSM_419_Smed_4342_minE
Sinorhizobium_meliloti_1021_SM_b21522_minE
2.0E-4

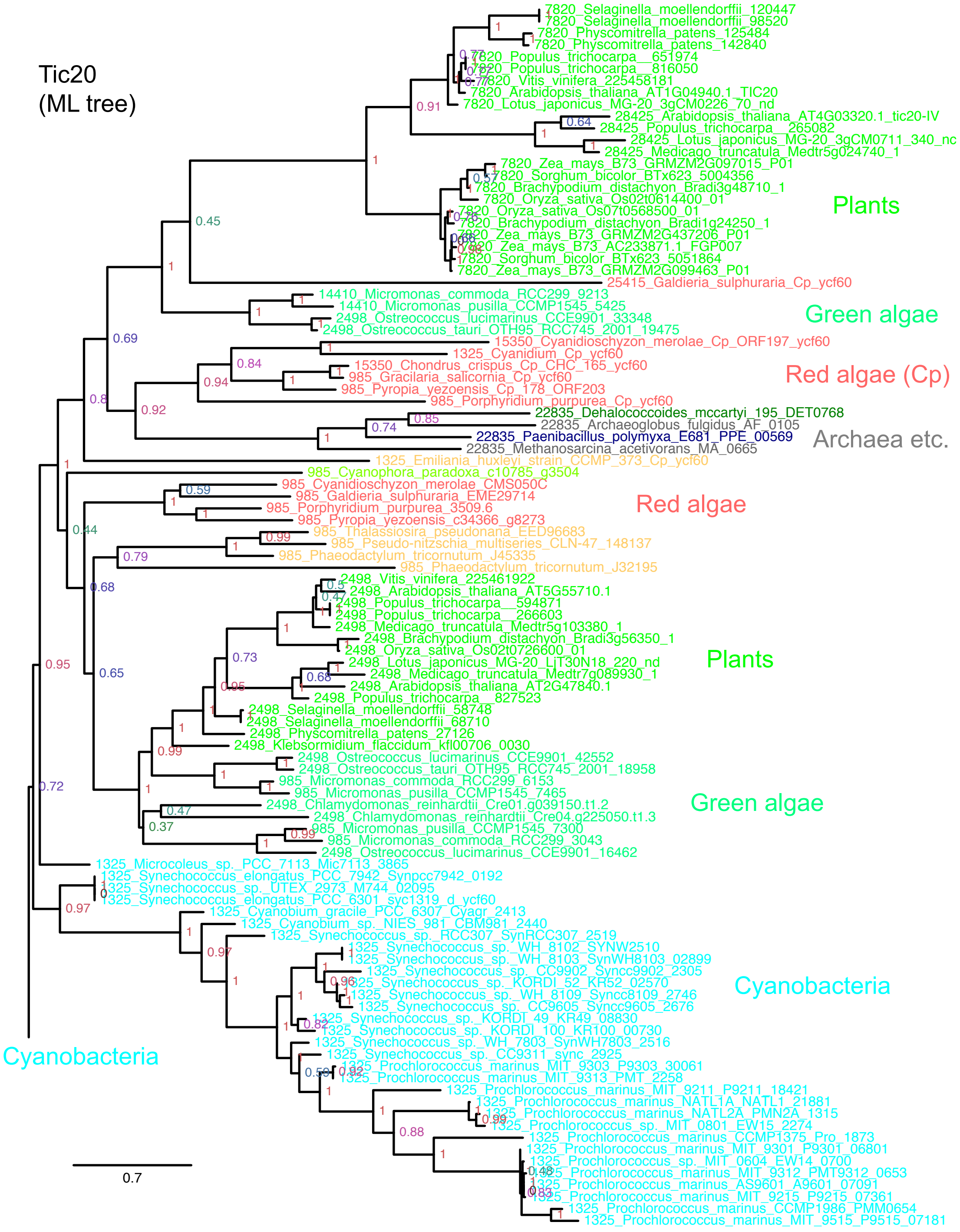

Tic20
(ML tree)
7820_Selaginella_moellendorffii_120447
7820_Selaginella_moellendorffii_98520
7820_Physcomitrella_patens_125484
7820_Physcomitrella_patens_142840
7820_Populus_trichocarpa__651974
7820_Populus_trichocarpa__816050
7820_Vitis_vinifera_225458181
7820_Arabidopsis_thaliana_AT1G04940.1_TIC20
7820_Lotus_japonicus_MG-20_3gCM0226_70_nd
28425_Arabidopsis_thaliana_AT4G03320.1_tic20-IV
28425_Populus_trichocarpa__265082
28425_Lotus_japonicus_MG-20_3gCM0711_340_nc
28425_Medicago_truncatula_Medtr5g024740_1
7820_Zea_mays_B73_GRMZM2G097015_P01
7820_Sorghum_bicolor_BTx623_5004356
7820_Brachypodium_distachyon_Bradi3g48710_1
7820_Oryza_sativa_Os02t0614400_01
7820_Oryza_sativa_Os07t0568500_01
7820_Brachypodium_distachyon_Bradi1g24250_1
7820_Zea_mays_B73_GRMZM2G437206_P01
7820_Zea_mays_B73_AC233871.1_FGP007
7820_Sorghum_bicolor_BTx623_5051864
7820_Zea_mays_B73_GRMZM2G099463_P01
Plants
25415_Galdieria_sulphuraria_Cp_ycf60
14410_Micromonas_commoda_RCC299_9213
14410_Micromonas_pusilla_CCMP1545_5425
2498_Ostreococcus_lucimarinus_CCE9901_33348
2498_Ostreococcus_tauri_OTH95_RCC745_2001_19475
Green algae
15350_Cyanidioschyzon_merolae_Cp_ORF197_ycf60
1325_Cyanidium_Cp_ycf60
15350_Chondrus_crispus_Cp_CHC_165_ycf60
985_Gracilaria_salicornia_Cp_ycf60
985_Pyropia_yezoensis_Cp_178_ORF203
985_Porphyridium_purpurea_Cp_ycf60
Red algae (Cp)
22835_Dehalococcoides_mccartyi_195_DET0768
22835_Archaeoglobus_fulgidus_AF_0105
22835_Paenibacillus_polymyxa_E681_PPE_00569
22835_Methanosarcina_acetivorans_MA_0665
Archaea etc.
1325_Emiliania_huxleyi_strain_CCMP_373_Cp_ycf60
985_Cyanophora_paradoxa_c10785_g3504
985_Cyanidioschyzon_merolae_CMS050C
985_Galdieria_sulphuraria_EME29714
985_Porphyridium_purpurea_3509.6
985_Pyropia_yezoensis_c34366_g8273
Red algae
985_Thalassiosira_pseudonana_EED96683
985_Pseudo-nitzschia_multiseries_CLN-47_148137
985_Phaeodactylum_tricornutum_J45335
985_Phaeodactylum_tricornutum_J32195
2498_Vitis_vinifera_225461922
2498_Arabidopsis_thaliana_AT5G55710.1
2498_Populus_trichocarpa__594871
2498_Populus_trichocarpa__266603
2498_Medicago_truncatula_Medtr5g103380_1
2498_Brachypodium_distachyon_Bradi3g56350_1
2498_Oryza_sativa_Os02t0726600_01
2498_Lotus_japonicus_MG-20_LjT30N18_220_nd
2498_Medicago_truncatula_Medtr7g089930_1
2498_Arabidopsis_thaliana_AT2G47840.1
2498_Populus_trichocarpa__827523
2498_Selaginella_moellendorffii_58748
2498_Selaginella_moellendorffii_68710
2498_Physcomitrella_patens_27126
2498_Klebsormidium_flaccidum_kfl00706_0030
Plants
2498_Ostreococcus_lucimarinus_CCE9901_42552
2498_Ostreococcus_tauri_OTH95_RCC745_2001_18958
985_Micromonas_commoda_RCC299_6153
985_Micromonas_pusilla_CCMP1545_7465
2498_Chlamydomonas_reinhardtii_Cre01.g039150.t1.2
2498_Chlamydomonas_reinhardtii_Cre04.g225050.t1.3
985_Micromonas_pusilla_CCMP1545_7300
985_Micromonas_commoda_RCC299_3043
2498_Ostreococcus_lucimarinus_CCE9901_16462
Green algae
1325_Microcoleus_sp._PCC_7113_Mic7113_3865
1325_Synechococcus_elongatus_PCC_7942_Synpcc7942_0192
1325_Synechococcus_sp._UTEX_2973_M744_02095
1325_Synechococcus_elongatus_PCC_6301_syc1319_d_ycf60
1325_Cyanobium_gracile_PCC_6307_Cyagr_2413
1325_Cyanobium_sp._NIES_981_CBM981_2440
1325_Synechococcus_sp._RCC307_SynRCC307_2519
1325_Synechococcus_sp._WH_8102_SYNW2510
1325_Synechococcus_sp._WH_8103_SynWH8103_02899
1325_Synechococcus_sp._CC9902_Syncc9902_2305
1325_Synechococcus_sp._KORDI_52_KR52_02570
1325_Synechococcus_sp._WH_8109_Syncc8109_2746
1325_Synechococcus_sp._CC9605_Syncc9605_2676
1325_Synechococcus_sp._KORDI_49_KR49_08830
1325_Synechococcus_sp._KORDI_100_KR100_00730
1325_Synechococcus_sp._WH_7803_SynWH7803_2516
1325_Synechococcus_sp._CC9311_sync_2925
1325_Prochlorococcus_marinus_MIT_9303_P9303_30061
1325_Prochlorococcus_marinus_MIT_9313_PMT_2258
1325_Prochlorococcus_marinus_MIT_9211_P9211_18421
1325_Prochlorococcus_marinus_NATL1A_NATL1_21881
1325_Prochlorococcus_marinus_NATL2A_PMN2A_1315
1325_Prochlorococcus_sp._MIT_0801_EW15_2274
1325_Prochlorococcus_marinus_CCMP1375_Pro_1873
1325_Prochlorococcus_marinus_MIT_9301_P9301_06801
1325_Prochlorococcus_sp._MIT_0604_EW14_0700
1325_Prochlorococcus_marinus_MIT_9312_PMT9312_0653
1325_Prochlorococcus_marinus_AS9601_A9601_07091
1325_Prochlorococcus_marinus_MIT_9215_P9215_07361
1325_Prochlorococcus_marinus_CCMP1986_PMM0654
1325_Prochlorococcus_marinus_MIT_9515_P9515_07181
Cyanobacteria
Cyanobacteria
0.7
